# Burden of disease in Brazil, 1990–2016: a systematic subnational analysis for the Global Burden of Disease Study 2016

**DOI:** 10.1016/S0140-6736(18)31221-2

**Published:** 2018-09-01

**Authors:** GBD 2016 Brazil Collaborators, Fatima Marinho, Valéria Maria de Azeredo Passos, Deborah Carvalho Malta, Elizabeth Barboza França, Daisy M X Abreu, Valdelaine E M Araújo, Maria Teresa Bustamante-Teixeira, Paulo A M Camargos, Carolina Cândida da Cunha, Bruce Bartholow Duncan, Mariana Santos Felisbino-Mendes, Maximiliano Ribeiro Guerra, Mark D C Guimaraes, Paulo A Lotufo, Wagner Marcenes, Patricia Pereira Vasconcelos Oliveira, Marcel de Moares Pedroso, Antonio L Ribeiro, Maria Inês Schmidt, Renato Azeredo Teixeira, Ana Maria Nogales Vasconcelos, Mauricio L Barreto, Isabela M Bensenor, Luisa C C Brant, Rafael M Claro, Alexandre Costa Pereira, Ewerton Cousin, Maria Paula Curado, Kadine Priscila Bender dos Santos, André Faro, Cleusa P Ferri, João M Furtado, Julia Gall, Scott D Glenn, Alessandra Carvalho Goulart, Lenice Harumi Ishitani, Christian Kieling, Roberto Marini Ladeira, Isis Eloah Machado, Sheila Cristina Ouriques Martins, Francisco Rogerlândio Martins-Melo, Ana Paula Souto Melo, Molly K Miller-Petrie, Meghan D Mooney, Bruno P Nunes, Marcos Roberto Tovani Palone, Claudia C Pereira, Davide Rasella, Sarah E Ray, Leonardo Roever, Raphael de Freitas Saldanha, Itamar S Santos, Ione J C Schneider, Diego Augusto Santos Silva, Dayane Gabriele Alves Silveira, Adauto Martins Soares Filho, Tatiane Cristina Moraes Sousa, Celia L Szwarcwald, Jefferson Traebert, Gustavo Velasquez-Melendez, Yuan-Pang Wang, Rafael Lozano, Christopher J L Murray, Mohsen Naghavi

## Abstract

**Background:**

Political, economic, and epidemiological changes in Brazil have affected health and the health system. We used the Global Burden of Disease Study 2016 (GBD 2016) results to understand changing health patterns and inform policy responses.

**Methods:**

We analysed GBD 2016 estimates for life expectancy at birth (LE), healthy life expectancy (HALE), all-cause and cause-specific mortality, years of life lost (YLLs), years lived with disability (YLDs), disability-adjusted life-years (DALYs), and risk factors for Brazil, its 26 states, and the Federal District from 1990 to 2016, and compared these with national estimates for ten comparator countries.

**Findings:**

Nationally, LE increased from 68·4 years (95% uncertainty interval [UI] 68·0–68·9) in 1990 to 75·2 years (74·7–75·7) in 2016, and HALE increased from 59·8 years (57·1–62·1) to 65·5 years (62·5–68·0). All-cause age-standardised mortality rates decreased by 34·0% (33·4–34·5), while all-cause age-standardised DALY rates decreased by 30·2% (27·7–32·8); the magnitude of declines varied among states. In 2016, ischaemic heart disease was the leading cause of age-standardised YLLs, followed by interpersonal violence. Low back and neck pain, sense organ diseases, and skin diseases were the main causes of YLDs in 1990 and 2016. Leading risk factors contributing to DALYs in 2016 were alcohol and drug use, high blood pressure, and high body-mass index.

**Interpretation:**

Health improved from 1990 to 2016, but improvements and disease burden varied between states. An epidemiological transition towards non-communicable diseases and related risks occurred nationally, but later in some states, while interpersonal violence grew as a health concern. Policy makers can use these results to address health disparities.

**Funding:**

Bill & Melinda Gates Foundation and the Brazilian Ministry of Health.

## Introduction

Brazil has undergone important structural and economic changes over the past 50 years. 21 years of military dictatorship came to an end in 1985, and in 1988, a Constitutional Assembly edited a new constitution establishing health as a “right for all” and a “duty of the state”, leading to health care reforms to create the universal Brazilian Unified Health System (SUS).^1^, ^2^ National immunisation programmes have provided vaccines to all citizens since 1973,^3^ and since 1994, the Estratégia Saúde da Família^4^ (ESF–Family Health Strategy) has reorganised primary health services to guarantee universal access, improve health education, and increase health promotion. These reforms have taken place within an increasingly urban and globalised national context that has shifted social structures and further affected patterns of disease. Improvements in health outcomes from communicable diseases have been one notable result of the reforms, while the growing burden of non-communicable diseases (NCDs) presents a new challenge.^5^ Industrialisation, urbanisation, economic growth, changes in income inequality, and the introduction of national and community health programmes are among the factors that have spurred declines in mortality and fertility in Brazil,^1^, ^6^, ^7^ and the subsequent expansion of the older population and decreasing population in the labour force demand new policies for health and social security.^8^, ^9^ High levels of violence, fuelled in part by the illegal drug trade, present another important health challenge.^10^, ^11^

Geographically disaggregated data for health outcomes^12^, ^13^, ^14^, ^15^ have allowed policy makers in many countries to better understand and allocate resources towards domestic health problems. Brazil is home to 209·8 million citizens living in 26 states and the Federal District; the population of the state of São Paulo alone, at 45·4 million, is greater than the total population of neighbouring Argentina. The states are grouped into five geographic macro-regions: the north, northeast, central-west, southeast, and south. States in the regions of the south and southeast, which include the major cities of São Paulo and Rio de Janeiro, are generally more urban and industrialised, with better infrastructure, compared with states in the regions of the north and northeast.^1^ Table 1 provides a complete list of states with information on population and Socio-demographic Index (SDI). Demographic and epidemiological changes in Brazil have not been experienced uniformly across states, resulting in subnational disparities in health and corresponding burdens on health systems.

**Table 1:** Population, SDI, and cause of death star rating for Brazil, its 26 states, and the Federal District, both sexes, 1980–2016

		**Population**	**SDI**	**1980–2016**	**1980–84**	**1985–89**	**1990–94**	**1995–99**	**2000–04**	**2005–09**	**2010–16**
Brazil		209 813 840	0·71	★★★★⋆	★★★⋆⋆	★★★⋆⋆	★★★⋆⋆	★★★★⋆	★★★★⋆	★★★★⋆	★★★★⋆
North region											
	Acre	838 127	0·64	★★★⋆⋆	★★★⋆⋆	★★★⋆⋆	★★★⋆⋆	★★★⋆⋆	★★★⋆⋆	★★★★⋆	★★★★⋆
	Amapá	802 040	0·68	★★★★⋆	★★★★⋆	★★★★⋆	★★★★⋆	★★★★⋆	★★★★⋆	★★★★⋆	★★★★⋆
	Amazonas	4 078 537	0·69	★★★⋆⋆	★★★⋆⋆	★★★⋆⋆	★★★⋆⋆	★★★⋆⋆	★★★★⋆	★★★★⋆	★★★★⋆
	Pará	8 465 727	0·63	★★★⋆⋆	★★★⋆⋆	★★★⋆⋆	★★★⋆⋆	★★★⋆⋆	★★★⋆⋆	★★★★⋆	★★★★⋆
	Rondônia	1 828 786	0·67	★★★★⋆	★★★★⋆	★★★★⋆	★★★★⋆	★★★★⋆	★★★★⋆	★★★★⋆	★★★★⋆
	Roraima	534 068	0·68	★★★★⋆	★★★★⋆	★★★★⋆	★★★★⋆	★★★★⋆	★★★★⋆	★★★★⋆	★★★★⋆
	Tocantins	1 580 590	0·67	★★★⋆⋆	⋆⋆⋆⋆⋆	⋆⋆⋆⋆⋆	★★★⋆⋆	★★★⋆⋆	★★★★⋆	★★★★⋆	★★★★★
Northeast region											
	Alagoas	3 418 963	0·61	★★★⋆⋆	★★⋆⋆⋆	★★★⋆⋆	★★★⋆⋆	★★★⋆⋆	★★★⋆⋆	★★★★⋆	★★★★⋆
	Bahia	15 693 986	0·64	★★★⋆⋆	★★★⋆⋆	★★★⋆⋆	★★★⋆⋆	★★★⋆⋆	★★★⋆⋆	★★★★⋆	★★★★⋆
	Ceará	9 151 099	0·64	★★★⋆⋆	★★⋆⋆⋆	★★⋆⋆⋆	★★★⋆⋆	★★★⋆⋆	★★★⋆⋆	★★★★⋆	★★★★⋆
	Maranhão	7 084 284	0·60	★★★⋆⋆	★★⋆⋆⋆	★★⋆⋆⋆	★★⋆⋆⋆	★★⋆⋆⋆	★★★⋆⋆	★★★★⋆	★★★★⋆
	Paraíba	4 025 557	0·63	★★★⋆⋆	★★⋆⋆⋆	★★★⋆⋆	★★★⋆⋆	★★★⋆⋆	★★★⋆⋆	★★★★⋆	★★★★⋆
	Pernambuco	9 635 500	0·64	★★★⋆⋆	★★★⋆⋆	★★★⋆⋆	★★★⋆⋆	★★★⋆⋆	★★★★⋆	★★★★⋆	★★★★⋆
	Piaui	3 278 234	0·60	★★★⋆⋆	★★⋆⋆⋆	★★⋆⋆⋆	★★★⋆⋆	★★★⋆⋆	★★★⋆⋆	★★★★⋆	★★★★⋆
	Rio Grande do Norte	3 520 016	0·66	★★★⋆⋆	★★⋆⋆⋆	★★★⋆⋆	★★★⋆⋆	★★★⋆⋆	★★★★⋆	★★★★⋆	★★★★★
	Sergipe	2 290 601	0·66	★★★⋆⋆	★★⋆⋆⋆	★★⋆⋆⋆	★★★⋆⋆	★★★⋆⋆	★★★★⋆	★★★★⋆	★★★★⋆
Central-west region											
	Distrito Federal	3 006 931	0·83	★★★★★	★★★★⋆	★★★★★	★★★★★	★★★★★	★★★★★	★★★★★	★★★★★
	Goiás	6 890 890	0·69	★★★★⋆	★★★⋆⋆	★★★⋆⋆	★★★★⋆	★★★★⋆	★★★★⋆	★★★★⋆	★★★★★
	Mato Grosso	3 423 154	0·70	★★★★⋆	★★★⋆⋆	★★★⋆⋆	★★★⋆⋆	★★★★⋆	★★★★⋆	★★★★⋆	★★★★⋆
	Mato Grosso do Sul	2 750 887	0·69	★★★★⋆	★★★★⋆	★★★★⋆	★★★★⋆	★★★★⋆	★★★★★	★★★★★	★★★★★
Southeast region											
	Espírito Santo	4 047 161	0·72	★★★★⋆	★★★★⋆	★★★★⋆	★★★★⋆	★★★★⋆	★★★★⋆	★★★★★	★★★★★
	Minas Gerais	21 268 245	0·70	★★★★⋆	★★★★⋆	★★★★⋆	★★★★⋆	★★★★⋆	★★★★⋆	★★★★⋆	★★★★⋆
	Rio de Janeiro	16 929 511	0·75	★★★★⋆	★★★★⋆	★★★★⋆	★★★★⋆	★★★★⋆	★★★★⋆	★★★★⋆	★★★★⋆
	São Paulo	45 400 932	0·76	★★★★⋆	★★★★⋆	★★★★⋆	★★★★⋆	★★★★⋆	★★★★⋆	★★★★⋆	★★★★⋆
South region											
	Paraná	11 419 677	0·72	★★★★⋆	★★★★⋆	★★★★⋆	★★★★⋆	★★★★⋆	★★★★⋆	★★★★★	★★★★★
	Santa Catarina	6 948 258	0·74	★★★★⋆	★★★⋆⋆	★★★★⋆	★★★★⋆	★★★★⋆	★★★★⋆	★★★★⋆	★★★★★
	Rio Grande do Sul	11 502 077	0·73	★★★★⋆	★★★★⋆	★★★★⋆	★★★★⋆	★★★★⋆	★★★★⋆	★★★★★	★★★★★

For the GBD 2016 study, the percentage of well-certified deaths across the time series by location was assessed and assigned from 0 to 5 stars: 5 stars where percentage well certified equaled or exceeded 85%; 4 stars for 65% to less than 85%; 3 stars for 35% to less than 65%; 1 star for greater than 0% to less than 10%; and 0 stars for 0% well certified. SDI=Socio-demographic Index.

**Research in context**

**Evidence before this study**

The Global Burden of Disease Study 2016 (GBD 2016) reported on 333 causes of disease and injury in 195 countries and territories from 1990 to 2016. Previous versions of the GBD were published for 1990, 2010, 2013, and 2015. GBD 2015 provided subnational estimates for Brazil's 26 states and the Federal District for the first time. In May, 2017, a supplement of the journal *Revista Brasileira de Epidemiologia* presented the burden of selected diseases in Brazil based on the results of GBD 2015, thanks to the GBD Brazil Network of researchers, created by the GBD Brazil Project, an agreement between the Ministry of Health, *Universidade Federal de Minas Gerais* (UFMG), and the Institute for Health Metrics and Evaluation (IHME) of the University of Washington (WA, USA).

**Added value of this study**

The GBD 2016 estimation of mortality, years of life lost, years lived with disability, disability-adjusted life-years, healthy life expectancy, and risk factors built on the evidence base published in GBD 2015, including estimates for an additional 18 causes of death and non-fatal disease. Substantial methodological improvements were made to provide the most accurate estimates available. Our study analysed subnational estimates for Brazil's 26 states and the Federal District, providing a detailed and nuanced picture of health in Brazil by geographical region for the first time. We used the GBD summary metric of Socio-demographic Index (SDI), a combined measure of average years of schooling above age 15 years, total fertility rate, and income per capita, to further assess whether health outcomes in Brazil were better or worse than would be expected based on SDI.

**Implications of all the available evidence**

This study provides the most comprehensive assessment to date of the levels and trends of disability and death in Brazil. While health outcomes have improved substantially since 1990, improvements have not been consistent across the geographical regions of the country. While the burden of communicable, maternal, neonatal, and nutritional diseases decreased, most of the disease burden at the national and state level is now due to non-communicable diseases. Road injuries and interpersonal violence are also responsible for a large portion of the national burden of disease. These data provide an opportunity for policy makers to target specific regions and health areas for improvement, as well to learn from those regions where health gains have been made.

Here we provide analyses of patterns of disease, disability, and related risk factors in Brazil, its states, and the Federal District to present the current picture of disease burden contrasted with ten comparator countries. Understanding health patterns over the past 26 years at regional and state levels will allow decision makers to better plan health policies, assess the effect of programmes, and allocate finances.

## Methods

### Overview

The Global Burden of Disease Study 2016 (GBD 2016) estimated disease burden due to 333 diseases and injuries, 2982 unique sequelae, and 84 risk factors for 195 countries and territories from 1990 to 2016. All metrics were estimated separately for Brazil's 26 states and the Federal District and are presented with their 95% uncertainty intervals (UIs). All rates presented are age-standardised per 100 000 population calculated using the world standard population developed for the GBD^16^, ^17^ unless otherwise specified. We applied the GBD star rating system to assess data completeness from 1980 to 2016 at the state level, as shown in table 1. A complete list of data sources used to produce Brazil estimates of cause-specific mortality, morbidity, and risk factors is available in the appendix (p 1).

Additional results can be explored using online data visualisation tools (now available in Portuguese) and downloaded using a query tool. Subnational data will also be available from the Institute for Scientific and Technological Communication and Information on Health (Icict/Fiocruz). This study complies with the Guidelines for Accurate and Transparent Health Estimates Reporting (GATHER; appendix p 60).^18^ Analyses were done with Python (versions 2.5.4 and 2.7.3), Stata (version 13.1), or R (version 3.1.2), and statistical code is available online. A comprehensive description of data sources, quality, and modelling for GBD 2016 has been reported elsewhere.^5^, ^17^, ^19^, ^20^, ^21^, ^22^

A network of Brazilian researchers and health workers collaborated with the Institute for Health Metrics and Evaluation (IHME; Seattle, WA, USA) to provide national and subnational data; assist in scientific literature reviews; analyse results; and translate visualisation tools. Previously, these efforts resulted in the publication of a special article in Portuguese on the burden of selected diseases as estimated in GBD 2015, as a supplement of the journal *Revista Brasileira de Epidemiologia*.^23^

### Mortality, causes of death, and years of life lost

We estimated all-cause mortality rates for each age-sex-location-year using multistage models of adult and under-5 mortality and the GBD model life table system.^17^ Cause-specific mortality rates were computed using the GBD cause-of-death database and, in most cases, the cause-of-death ensemble model (CODEm)^15^ as previously described.^5^, ^17^ The quality of each data source was assessed, and the International Classification of Diseases and Injuries (ICD; version 9 and 10) codes were mapped to the GBD 2016 cause list.^5^ The estimation process is described in greater detail in the appendix (p 69). We estimated years of life lost (YLLs) for each cause by location, age, sex, and year by multiplying each cause-specific death by the normative standard life expectancy at each age.^5^

### YLDs, DALYs, LE, and HALE

We calculated years lived with disability (YLDs) by multiplying the prevalence of each disease sequela by its disability weight, developed using population-based surveys, as described in the appendix (p 69) and elsewhere.^16^, ^19^, ^24^ Disability-adjusted life-years (DALYs), a combined measure of health lost to fatal and non-fatal causes (appendix p 70), are calculated as the sum of YLLs and YLDs for each age-sex-location-year.^20^ The calculations for life expectancy at birth (LE) and maximum life expectancy at each age have been previously reported.^17^ Healthy life expectancy (HALE) summarises overall population health accounting for length of life and level of health loss by age using YLD estimates and the GBD life tables, as previously described.^20^

### Risk factors

Relative risk of mortality and morbidity, exposure to each risk factor, and ultimately attributable deaths or DALYs were estimated for each risk-outcome pair. This process is explained in greater detail in the appendix (p 70) and in prior publications.^21^

### SDI

The SDI is calculated as the geometric mean of rescaled values of income per capita, total fertility rate, and average educational attainment, as described in detail in the appendix (p 70) and elsewhere.^5^ The components of the SDI indicator are strongly correlated with health outcomes.

### GBD star rating system

For GBD 2016, a star rating system from 0 to 5 was developed to assess the quality of cause of death data in each location-year. A higher star rating indicates greater completeness, availability, and detail of mortality data, as well as lower levels of garbage codes and fewer aggregated causes (these terms are described in greater detail in the methods appendix). The percentage of data well-certified indicates the percentage of total deaths for which the detailed cause of death was known, by location-year. Based on these metrics, Brazil was assigned four out of five stars in 2016, with 70·5% well-certified data for the period 2010–2016. Star rankings of data quality in each state are shown in table 1. We have dropped every state-year of data with less than 50% data completeness or more than 50% of garbage codes in level 1 and level 2, which amounted to 27 state-years of data, all prior to 1998. All included sources were ultimately adjusted to 100% completeness, as described elsewhere.^5^

### Benchmarking

We compared outcomes in Brazil (SDI 0·708) to outcomes in the emerging economies that constitute the BRICS group^25^ (Russia [SDI 0·832], India [SDI 0·584], China [SDI 0·727], and South Africa [SDI 0·734]), countries with similar socioeconomic status and/or geographic proximity in Latin America (Mexico [SDI 0·734], Argentina [SDI 0·761], and Colombia [SDI 0·707]), and select high-SDI countries with universal health care (Canada [SDI 0·908], Australia [SDI 0·892], and England [SDI 0·866]).^5^ The subnational analysis compared all metrics among Brazilian states in 1990 and 2016.

### Role of the funding source

The funders had no role in study design, data collection, analysis, interpretation, or writing of the report. All authors had full access to the data in the study and had final responsibility for the decision to submit for publication.

## Results

Between 1990 and 2016, LE in Brazil increased by 6·8 years, from 68·4 years (95% uncertainty interval [UI] 68·0–68·9) to 75·2 years (74·7–75·7; table 2). HALE increased by 5·7 years, from 59·8 (57·1–62·1) to 65·5 (62·5–68·0). In 2016, the three states with the lowest LE were in the northeast region: Alagoas (73·5 years [72·3–74·8]), Pernambuco (73·6 years [72·2–74·8]), and Sergipe (73·8 years [72·7–75·1]) (table 3). However, between 1990 and 2016, Alagoas also had the largest increase in LE (9·5 years), with the smallest increases seen in Piauí (1·6 years) in the northeast and Amapá (1·8 years) in the north. The states with the highest LE in 2016 were the Federal District (77·8 years [76·8–78·9]) in the central-west and Santa Catarina (76·2 years [75·0–77·2]) in the south (table 3).

**Table 2:** Life expectancy, healthy life expectancy (HALE), mortality, years of life lost (YLLs), and years lived with disability (YLDs) in Brazil and comparator countries, both sexes, 1990 and 2016

	**Life expectancy**		**HALE**		**Age-standardised mortality rate (per 100 000)**		**Age-standardised YLL rate (per 100 000)**		**Age-standardised YLD rate (per 100 000)**		**Age-standardised DALY rate (per 100 000)**	
	1990	2016	1990	2016	1990	2016	1990	2016	1990	2016	1990	2016
Brazil	68·4 (68·0–68·9)	75·2 (74·7–75·7)	59·8 (57·1–62·1)	65·5 (62·5–68·0)	1116·6 (1098·9–1134·8)	737 (719·6–756·3)	29 348·3 (28 862·4–29 835·6)	17 412·9 (16 965·3–17 884·9)	11 354·9 (83 93·2–14 744·1)	11 011·7 (8174·7–14 312·0)	40 703·2 (37 686·9–44 088·9)	28 424·7 (25 411·7–31 646·7)
Russia	69·2 (68·1–70·3)	70·9 (67·1–74·5)	60·2 (57·5–62·8)	61·9 (58·1–65·7)	1125·8 (1044·8–1210·0)	1009·4 (785·9–1311·1)	26 988·5 (24 966·6–29 024·0)	23 717·3 (17 945·9–30 954·5)	11 676·4 (8719·7–15 118·6)	11 263·6 (8392·2–14 580·7)	38 664·8 (34 821·1–42 330·4)	34 980·8 (28 197·5–43 102·1)
India	59 (58·5–59·4)	68·5 (67·9–69·1)	50·8 (48·4–52·9)	58·9 (56·1–61·3)	1652·7 (1616·9–1686·2)	1138·7 (1112·4–1165·6)	51 612·7 (50 508·8–52 671·8)	28 642·3 (27 928·9–29 358·5)	13 197·6 (9945·3–17 015·0)	12 720·1 (9574·9–16 399·9)	64 810·3 (61 303·3–68 652·5)	41 362·3 (38 221–44 965·3)
China	67 (66·4–67·4)	76·4 (76·1–76·6)	59·9 (57·8–61·7)	67·9 (65·4–70·0)	1259 (1236·6–1280·6)	730·3 (716·4–744·1)	32 049·1 (31 431·9–32 675·7)	14 809·1 (14 478·7–15 141·2)	9645·1 (7189·4–12 516·5)	9200·9 (6862·1–11 942·7)	41 694·2 (39 004·9–44 612·3)	41 694·2 (39 004·9–44 612·3)
South Africa	64·3 (63·7–65·0)	62·4 (61·2–63·5)	55·8 (53·3–58·0)	53·8 (51·4–56·3)	1130·4 (1091·2–1171·0)	1305·8 (1250·6–1359·9)	39 510·9 (37 720·0–41 312·2)	42 905·2 (40 366·2–45 756·7)	11 932·4 (8923·6–15 475·8)	12 850·6 (9631·5–16574·0)	51 443·3 (48 012·9–55 251·5)	55 755·7 (51 450·4–60 664·5)
Mexico	71·5 (71·2–71·8)	76·4 (76·0–76·8)	62·8 (60·2–65·1)	67·1 (64·3–69·4)	864·5 (854·1–875·1)	666·2 (651·9–680·1)	24 298·8 (23 983·2–24 591·9)	15 888·5 (15 539·0–16 223·1)	10 284·7 (7629·1–13 354·6)	9972·5 (7374·1–12 916·0)	34 583·5 (31 931·4–37 638·2)	25 861 (23 271·2–28 978·9)
Argentina	72·1 (71·7–72·5)	76·7 (76·0–77·5)	63·4 (60·7–65·7)	67·2 (64·4–69·8)	921 (893·8–945·6)	685·3 (642·7–727·7)	22 200 (21 594·2–22 755·5)	14 748·9 (13 847·6–15 722·1)	10 680·6 (7910·9–13 842·3)	10 548·3 (7787·6–13 674·0)	32 880·6 (30 005·8–36 103·3)	25 297·3 (22 363·9–28 425·7)
Colombia	71·3 (70·9–71·7)	78·3 (77·3–79·2)	63·1 (60·5–65·3)	69·1 (66·3–71·6)	904·2 (882·4–927·9)	589·5 (546·2–635·3)	24 336·7 (23 459·5–25 264·8)	13 493·5 (12 470·0–14 623·9)	9925·3 (7346·4–13 046·3)	9494·7 (7012·6–12 401·7)	34 262·1 (31 436·1–37 488·7)	22 988·3 (20 177·6–26 166)
Australia	77 (76·8–77·2)	82·5 (82·0–83·0)	67·1 (64·2–69·6)	71·5 (68·4–74·3)	696·8 (685·2–709·5)	437·2 (416·0–461·0)	13 832·8 (13 591·4–14 094·5)	7879·6 (7469·2–8347·0)	11 106·6 (8226·2–14 375·4)	10 901·2 (8064·2–14 130·5)	25 262·8 (22 297·2–28 494·6)	18 948·7 (16 210·7–22 088·3)
Canada	77·4 (77·2–77·7)	81·9 (81·5–82·2)	67·8 (65·0–70·3)	71·2 (68·1–74·0)	664·9 (651·8–676·6)	457·4 (442·8–470·8)	13 429·8 (13 150·9–13 685·2)	8798·2 (8482·7–9112·6)	10 611·9 (7872·2–13 724·6)	10 598 (7885·9–13 701·3)	24 041·7 (21 293–27 134·3)	19 396·3 (16 597·8–22 491·1)
England	76 (75·9–76·0)	81·2 (81·1–81·3)	66 (63·2–68·6)	70·2 (67·1–73·0)	773·5 (769·0–778·2)	498·2 (493·0–502·8)	14 915·4 (14 823·1–15 010·8)	8941 (8847·4–9028·8)	11 294·9 (8385·2–14 568·6)	11 053·6 (8210·9–14 261·4)	26 210·3 (23 311·7–29 499·3)	19 994·6 (17 149–23 222·1)

DALY=disability-adjusted life-years.

**Table 3:** Life expectancy, HALE, and rates of mortality, YLLs, YLDs, and DALYs in Brazil, its 26 states, and the Federal District, both sexes, 1990 and 2016

		**Life expectancy**		**HALE**		**Age-standardised mortality rate (per 100 000)**		**Age-standardised YLL rate (per 100 000)**		**Age-standardised YLD rate (per 100 000)**		**Age-standardised DALY rate (per 100 000)**	
		1990	2016	1990	2016	1990	2016	1990	2016	1990	2016	1990	2016
Brazil		68·4 (68·0–68·9)	75·2 (74·7–75·7)	59·8 (57·1–62·1)	65·5 (62·5–68·0)	1116·6 (1098·9–1134·8)	737 (719·6–756·3)	29 348·3 (28 862·4–29 835·6)	17 412·9 (16 965·3–17 884·9)	11 354·9 (8393·2–14 744·1)	11 011·7 (8174·7–14 312·0)	40 703·2 (37 686·9–44 088·9)	28 424·7 (25 411·7–31 646·7)
North region													
	Acre	68·8 (67·8–69·6)	74·2 (72·8–75·3)	59·9 (57·2–62·2)	64·4 (61·5–67·1)	1008·9 (955·7–1064·7)	785·2 (720·3–853·5)	29 647 (27 865·6–31 551·9)	19 315 (17 385·8–21 308·3)	11 403·6 (8424·0–14 855·2)	11 190·4 (8286·1–14 563·8)	41 050·6 (37 494·6–44 967·3)	30 505·4 (26 977·7–34 258·6)
	Amapá	73·3 (72·5–74·1)	75·1 (73·9–76·3)	63·7 (60·8–66·3)	65·5 (62·5–68·3)	815 (769·9–861·5)	734·6 (670·0–803·0)	21 001·5 (19 745·9–22 222·8)	17 832·4 (16 264·7–19 673·6)	11 339·8 (8361·9–14 734·4)	10 866·7 (8069·1–14 158·5)	32 341·3 (29 027·8–35 996·7)	28 699·1 (25 204–32 444·5)
	Amazonas	71·3 (70·5–72·1)	75 (73·7–76·1)	62·2 (59·4–64·7)	65·5 (62·6–68·2)	895·9 (849·8–946·6)	763·4 (700·8–832·9)	24 603·3 (23 082·2–26 322·2)	17 726·1 (16 090·2–19 504·1)	11 160 (8228·7–14 503·2)	10 770·1 (7959·8–13 972·3)	35 763·3 (32 602·9–39 343·7)	28 496·2 (25 175·3–32 205·4)
	Para	71·3 (70·5–72·1)	75·2 (74·0–76·5)	62·1 (59·3–64·6)	65·5 (62·6–68·4)	864·1 (817·3–910·3)	729·7 (667·1–793·2)	25 082·4 (23 480·6–26 809·1)	17 588·5 (15 977·7–19 409·7)	11 180·8 (8267·2–14 554·0)	10 905·9 (8123·3–14 177·8)	36 263·2 (32 894·3–40 016·1)	28 494·4 (24 854·4–32 206)
	Rondônia	67·6 (66·7–68·4)	75·2 (74·0–76·3)	59·2 (56·6–61·5)	65·7 (62·9–68·4)	1197·6 (1127·6–1268·5)	771·8 (706·8–845·2)	31 021 (29 218·3–32 809·5)	17 063·5 (15 426·3–18 843·6)	11 338·9 (8370·4–14 796·3)	10 782·2 (7929·7–13 986·4)	42 359·9 (38 916·7–46 095·8)	27 845·7 (24 500·2–31 255·8)
	Roraima	68·4 (67·6–69·3)	74·1 (73·0–75·1)	59·8 (57·0–62·2)	64·7 (61·8–67·3)	1051·3 (1000·3–1107·7)	812·6 (755·3–874·4)	29 900·2 (27 978·6–31 897·7)	19 199·9 (17 806·7–20 732·6)	11 274·4 (8353·9–14 698·3)	10 946·3 (8027·4–14 276·0)	41 174·6 (37 694·3–44 847·5)	30 146·3 (27 030·2–33 792·6)
	Tocantins	72·3 (71·1–73·7)	75·3 (74·2–76·6)	62·9 (59·8–65·6)	65·5 (62·5–68·3)	811·5 (752·6–876·1)	726·7 (663·0–790·2)	23 645·2 (21 545·6–26 028·5)	17 488·7 (15 801·1–19 336·0)	11 125·8 (8201·5–14 462·0)	11 056·8 (8236·7–14 399·9)	34 771 (31 013·2–38 749·7)	28 545·5 (24 985·2–32 246·9)
Northeast region													
	Alagoas	64 (63·0–65·2)	73·5 (72·3–74·8)	55·9 (53·2–58·3)	64·1 (61·3–66·7)	1222·7 (1163·3–1286·0)	808·2 (741·8–879·8)	39 653·1 (37 165·4–42 360·3)	20 078·2 (18 238·9–22 126·1)	11 501·2 (8431·2–14 948·9)	11 089·2 (8188·1–14 448·5)	51 154·3 (47 234·9–55 410·3)	31 167·5 (27 783·4–34 962·8)
	Bahia	69·7 (68·8–70·6)	75·3 (74·0–76·6)	60·7 (57·8–63·2)	65·4 (62·3–68·2)	930·5 (886·9–973·4)	705·5 (645·1–774·9)	28 275·2 (26 633·3–29 999·6)	18 053·3 (16 187·8–20 233·0)	11 268·9 (8356·2–14 647·3)	11 123 (8234·8–14 464·0)	39 544·1 (36 101·8–43 279·5)	29 176·3 (25 703·4–33 049·1)
	Ceará	70·6 (69·7–71·6)	74·8 (73·6–75·9)	61·3 (58·3–63·8)	65·1 (62·1–67·8)	809·5 (769·9–851·5)	743 (684·4–803·3)	28 252·7 (26 266·3–30 373·9)	18 372·1 (16 638·3–20 335·0)	11 147·7 (8239·6–14 501·2)	10 998·7 (8178·8–14 309·0)	39 400·4 (35 933·8–43 203·5)	29 370·8 (25 881·7–33 280·2)
	Maranhão	69·8 (68·8–70·8)	75·1 (73·8–76·3)	60·5 (57·7–63·1)	65·2 (62·1–68·1)	876·4 (832·7–922·9)	710·2 (649·1–770·5)	29 092·1 (27 205·7–31 145·6)	18 395·9 (16 512·9–20 307·4)	11 401·3 (8447·8–14 893·1)	11 191·4 (8279·0–14 626·3)	40 493·4 (36 989·8–44 837·7)	29 587·3 (25 959·2–33 597·6)
	Paraíba	70·3 (69·5–71·1)	74·9 (73·7–76·0)	61·3 (58·5–63·8)	65·3 (62·2–67·9)	943·4 (890·0–994·2)	761·2 (702·4–826·3)	26 678·9 (25 058·3–28 418·3)	17 940 (16 354·1–19 732·7)	11 148·4 (8263·5–14 522·3)	10 955·6 (8087·9–14 176·5)	36 263·2 (32 894·3–40 016·1)	28 895·6 (25 255·1–32 715·8)
	Pernambuco	65·5 (64·3–66·6)	73·6 (72·2–74·8)	57·1 (54·4–59·5)	64·1 (61·1–67·0)	1215·8 (1153·3–1282·5)	826·2 (761·6–904·2)	35 993·4 (33 608·0–38 431·9)	19 883·7 (17 933·7–22 236·3)	11 555·8 (8605·0–14 999·9)	11 130·3 (8207·9–14 513·3)	47 549·2 (43 741·8–51 481·7)	31 014 (27 261·1–35 026)
	Piauí	73·2 (72·4–74·0)	74·8 (73·7–76·0)	63·6 (60·6–66·2)	65·1 (62·1–67·9)	743 (708·9–781·4)	753·9 (690·3–821·2)	22 750·5 (21 449·2–24 273·7)	18 175 (16 465·7–20 112·2)	11 157·3 (8215·2–14 535·2)	11 121·9 (8243·6–14 431·9)	33 907·8 (30 635·5–37 704·2)	29 296·9 (25 870·3–33 058·8)
	Rio Grande do Norte	71·9 (70·8–72·9)	76 (74·9–77·1)	62·4 (59·5–65·1)	66·1 (63·0–68·8)	811·7 (770·8–857·3)	688·5 (632·8–745·5)	24 815·9 (23 029·7–26 848·2)	16 521·3 (14 861·0–18 170·2)	11 168·9 (8258·2–14 499·0)	11 029·5 (8172·6–14 429·4)	35 984·8 (32 557·3–39 872·6)	27 550·8 (24 162·8–31 392·3)
	Sergipe	68·6 (67·7–69·5)	73·8 (72·7–75·1)	59·7 (57·0–62·2)	64·3 (61·4–66·9)	1041 (992·7–1095·1)	810·8 (747·1–875·7)	29 688·6 (27 991·0–31 561·8)	19 629·2 (17 730·4–21 562·9)	11 518·1 (8500·4–14 977·7)	11 165·3 (8254·0–14 503·7)	41 206·7 (37 682·6–44 918)	30 794·6 (27 322·9–34 662·1)
Central-west region													
	Federal District	70·6 (69·9–71·3)	77·8 (76·8–78·9)	61·7 (59·1–64·3)	67·6 (64·4–70·4)	1078·9 (1026·4–1137·1)	598·2 (548·3–652·7)	24 492 (23 256·3–25 924·3)	14 192·4 (12 937·9–15 388·2)	11 296·9 (8359·1–14 662·2)	10 847·8 (8064·9–14 148·8)	35 788·9 (32 396·9–39 363)	25 040·1 (21 834·4–28 577·1)
	Goiás	68·3 (67·6–69·1)	74·2 (73·0–75·3)	59·8 (57·2–62·1)	64·6 (61·7–67·3)	1208·8 (1143·1–1270·9)	801·7 (740·8–865·1)	29 019·4 (27 419·1–30 571·0)	18 891·7 (17 205·1–20 619·0)	11 432·3 (8500·3–14 848·3)	11 073 (8238·4–14 378·6)	40 451·8 (37 045·3–44 042·5)	29 964·7 (26 778·8–33 735·1)
	Mato Grosso	70·6 (69·7–71·4)	74·6 (73·3–75·9)	61·7 (59·0–64·0)	65 (62·1–67·7)	969·3 (917·0–1031·0)	785·2 (712·3–855·3)	25 371·5 (24 009·7–27 033·1)	18 134·3 (16 331·4–20 018·3)	11 136·7 (8254·1–14 467·5)	10 994·9 (8133·1–14 250·5)	36 508·2 (33 267·5–40 043)	29 129·2 (25 814·5–32 827·6)
	Mato Grosso do Sul	70·1 (69·4–71·0)	75·1 (74·0–76·3)	61·4 (58·8–63·9)	65·5 (62·5–68·2)	1071·7 (1011·2–1129·1)	766·9 (704·9–833·3)	25 559·1 (24 143·5–27 002·9)	17 158·7 (15 644·6–18 781·1)	11 210·7 (8271·8–14 602·5)	10 980·1 (8146·7–14 296·9)	36 769·8 (33 288·7–40 224)	28 138·7 (24 755·8–31 658·7)
Southeast region													
	Espírito Santo	69·1 (68·3–69·9)	75·8 (74·6–77·0)	60·5 (58·0–62·8)	65·9 (62·9–68·8)	1114·7 (1058·0–1175·3)	694·3 (638·1–753·8)	27 716·8 (26 176·6–29 392·6)	17 063·8 (15 352·8–18 860·1)	11 254·6 (8294·2–14 659·2)	10 936·5 (8076·4–14 213·3)	38 971·3 (35 592–42 573·1)	28 000·2 (24 640·1–31 662·6)
	Minas Gerais	68·8 (68·1–69·5)	76 (74·9–77·0)	60·2 (57·6–62·7)	66·2 (63·2–68·8)	1154·9 (1100·7–1209·4)	692·4 (638·7–748·1)	28 203·5 (26 822·9–29 689·9)	16 537·5 (14 992·2–18 184·3)	11 341 (8435·7–14 706·8)	10 861·1 (8082·8–14 106·8)	39 544·5 (36 119–43 356)	27 398·6 (24 308·1–30 756·4)
	Rio de Janeiro	66·1 (65·4–66·9)	74 (73·0–75·0)	58·1 (55·6–60·3)	64·8 (62·0–67·3)	1333 (1265·5–1399·1)	805·8 (744·9–862·8)	33 665·4 (31 953·5–35 422·8)	19 121·8 (17 586·1–20 560·5)	11 251·7 (8330·0–14 573·3)	10 694·4 (7960·8–13 912·1)	44 917·1 (41 437·8–48 649)	29 816·3 (26 508·9–33 253·1)
	São Paulo	68·5 (67·8–69·2)	76·1 (75·2–77·0)	59·9 (57·3–62·2)	66 (63·1–68·9)	1206 (1148·6–1264·6)	713 (662·8–768·3)	28 654 (27 285·1–30 020·9)	15 717·4 (14 542·5–16 931·0)	11 527·3 (8569·4–14 911·5)	11 163·5 (8290·9–14 475·9)	40 181·3 (36 768·6–43 968·7)	26 880·8 (23 638·3–30 416·8)
South region													
	Paraná	69·2 (68·5–69·8)	75·2 (74·1–76·3)	60·7 (58·1–63·0)	65·5 (62·5–68·2)	1204·7 (1147·7–1268·0)	756·7 (696·6–821·5)	27 069·6 (25 777·5–28 600·6)	17 167·9 (15 596·9–18 808·6)	11 257·8 (8283·8–14 611·5)	10 994·5 (8147·6–14 288·7)	38 327·4 (35 136·3–41 749·6)	28 162·4 (24 630·9–31 773·6)
	Rio Grande do Sul	70·2 (69·5–70·9)	75·7 (74·7–76·9)	61·5 (58·8–63·9)	66 (63·1–68·6)	1136·5 (1076·1–1198·2)	724·1 (661·3–784·8)	25 032·5 (23 684·9–26 300·0)	16 299·3 (14 861·0–17 735·8)	11 289·4 (8327·2–14 610·6)	10 978·8 (8073·1–14 259·3)	36 321·9 (33 036·3–39 881·6)	27 278·1 (24 115–30 620·1)
	Santa Catarina	69·7 (69·0–70·4)	76·2 (75·0–77·2)	61·2 (58·5–63·5)	66·4 (63·4–69·1)	1172·5 (1111·9–1238·6)	702·4 (648·0–766·1)	26 195 (24 785·6–27 603·7)	15 935·3 (14 449·5–17 606·8)	11 112·5 (8277·4–14 410·3)	10 772·3 (7980·1–14 012·9)	37 307·5 (33 937·7–41 025·6)	26 707·6 (23 436·2–30 318·8)

HALE=healthy life expectancy. YLLs=years of life lost. YLDs=years lived with disability. DALY=disability-adjusted life-years.

Nationally, all-cause age-standardised mortality rates decreased by 34·0% (95% UI 33·4 to 34·5) between 1990 and 2016, from 1116·6 deaths per 100 000 (1098·9 to 1134·8) to 737·0 deaths per 100 000 (719·6 to 756·3; table 2). The mortality rate decreased in all but one state (Piauí had a small but non-significant increase), while the magnitude of the declines differed between and within regions (table 3). The largest decreases were recorded among states in the south (from 36·3% [29·4 to 42·2] in Rio Grande do Sul to 40·1% [33·9 to 45·7] in Santa Catarina) and southeast (from 37·7% [31·3 to 43·4] in Espírito Santo to 40·9% [36·1 to 45·8] in São Paulo), while the smallest decreases were in states in the north (from 9·9% [0·3 to 19·2] in Amapá to 35·6% [28·7 to 41·8] in Rondônia) and northeast (from −1·5% [–11·4 to 8·3] in Piauí to 33·9% [27·1 to 40·2] in Alagoas).

**Figure 1:**
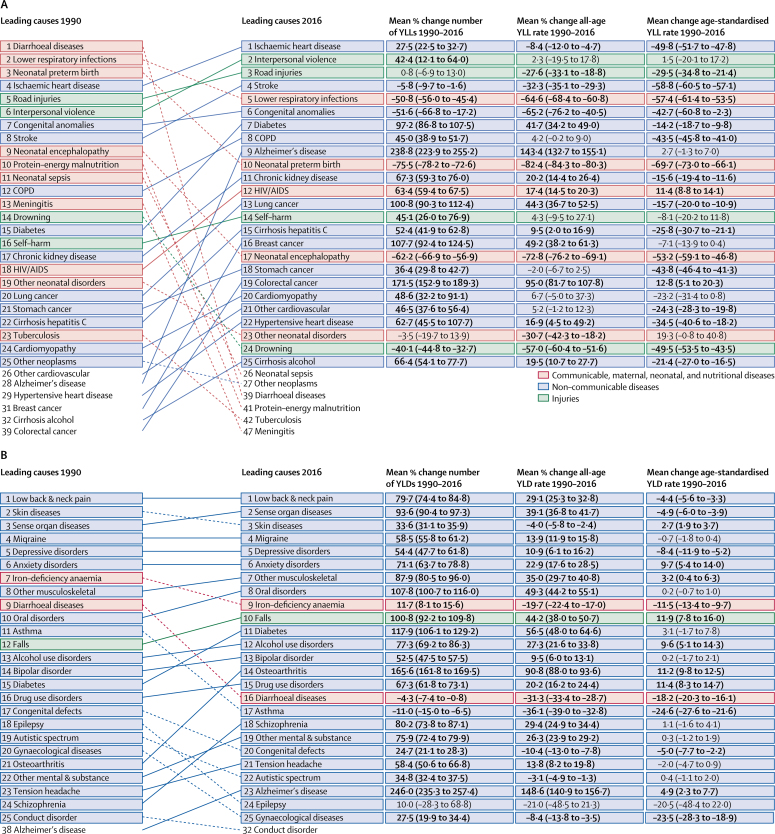
Leading 25 GBD Level 3 causes of YLLs (A) and YLDs (B) for 1990 and 2016, Brazil, both sexes YLLs=years of life lost. YLDs=years lived with disability.

Between 1990 and 2016, the age-standardised rate of YLLs decreased by 40·7% (95% UI 38·7 to 42·5) from 29 348·3 YLLs per 100 000 (28   862·4 to 29 835·6) to 17 412·9 YLLs per 100 000 (16 965·3 to 17 884·9; table 2). The leading cause of YLLs in 2016 was ischaemic heart disease, for which YLLs increased by 27·5% (22·5 to 32·7) from 1990, when it was the fourth leading cause (figure 1A). In 1990, the leading three causes of YLLs were diarrhoeal diseases, lower respiratory infections (LRIs), and neonatal preterm birth complications, which dropped to 39th, fifth, and tenth, respectively in 2016. Total YLLs due to interpersonal violence increased by 42·4% (12·1 to 64·0), rising from the sixth leading cause in 1990 to the second in 2016, while total YLLs for road injuries rose from the fifth leading cause to the third leading cause. Total YLLs also increased for HIV/AIDS and diabetes, among others. The age-standardised YLL rate of the ten leading causes in 2016 decreased over the period, with the exception of small and non-significant increases for interpersonal violence (1·5% [–20·1 to 17·2]) and Alzheimer's disease (2·7% [–1·3 to 7·0]). When all causes were aggregated one level further, the leading cause of YLLs in 2016 was cardiovascular diseases, followed by neoplasms (cancers).

**Figure 2:**
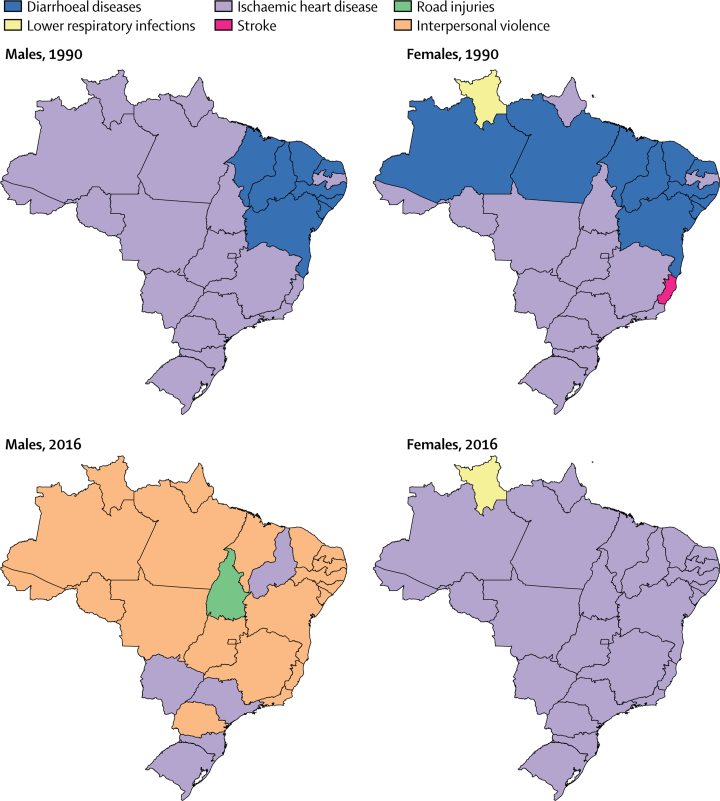
Leading causes of age-standardised YLLs in the states of Brazil, males and females, 1990 and 2016 YLLs=years of life lost.

For males, the leading causes of age-standardised YLLs in 1990 were diarrhoeal diseases in the northeast and ischaemic heart disease in the rest of the country (figure 2). This pattern was similar for females in 1990, although diarrhoeal disease was the leading cause in additional states in the north (Amazonas and Pará), and stroke and LRIs were the leading causes in Espírito Santo and Roraima, respectively. In 2016, interpersonal violence was the top cause of YLLs for males in most states, in addition to ischaemic heart disease in five states and road injuries in Toncantins. For females in 2016, ischaemic heart disease was the leading cause in all states except Roraima, where LRIs remained the leading cause.

The age-standardised rate of YLDs decreased slightly from 1990 to 2016, by 3·0% (95% UI 2·4 to 3·8), from 11 354·9 YLDs per 100 000 (8393·2 to 14 744·1) to 11 011·7 YLDs per 100 000 (8174·7 to 14 312·0; table 2). The morbidity profile has not changed substantially in Brazil since 1990: many leading causes of YLDs are non-communicable and chronic diseases (figure 1B). Low back and neck pain, skin diseases, and sense organ diseases such as hearing and vision loss were the main causes of YLDs in both 1990 and in 2016. Many diseases experienced an increasing percentage change in total number of YLDs but a decreasing percentage change in age-standardised YLD rates: low back and neck pain increased by 79·7% (74·4 to 84·8) in total YLDs and decreased by 4·4% (3·3 to 5·6) in age-standardised YLD rate. For diabetes there was a 117·9% (106·1 to 129·2) increase in number of YLDs but a small and non-significant increase of 3·1% (−1·7 to 7·8) in age-standardised YLD rate. Decreases in age-standardised YLD rates occurred for iron-deficiency anaemia (−11·5% [95% UI −9·7 to −13·4]), diarrhoeal diseases (−18·2% [–16·1 to −20·3]), and asthma (−24·6% [–21·6 to −27·6]).

At the state level, the Federal District had the lowest age-standardised YLL rate (14 192·4 YLLs [95% UI 12 937·9–15 388·2] per 100 000) in 2016, and the fifth lowest YLD rate (10 847·8 YLDs [8064·9–14 148·8] per 100 000), while São Paulo presented the second lowest age-standardised YLL rate (15 717·4 YLLs [14 542·5–16 931·0] per 100 000), but was among the states with the highest YLD rates (11 163·5 YLDs [8290·0–14 475·9] per 100 000; table 3). The highest YLD and YLL rates occurred in states in the northeast: the highest YLD rate was in Pernambuco in 1990 and Maranhão in 2016, while Alagoas had the highest YLL rate in both 1990 and 2016.

**Figure 3:**
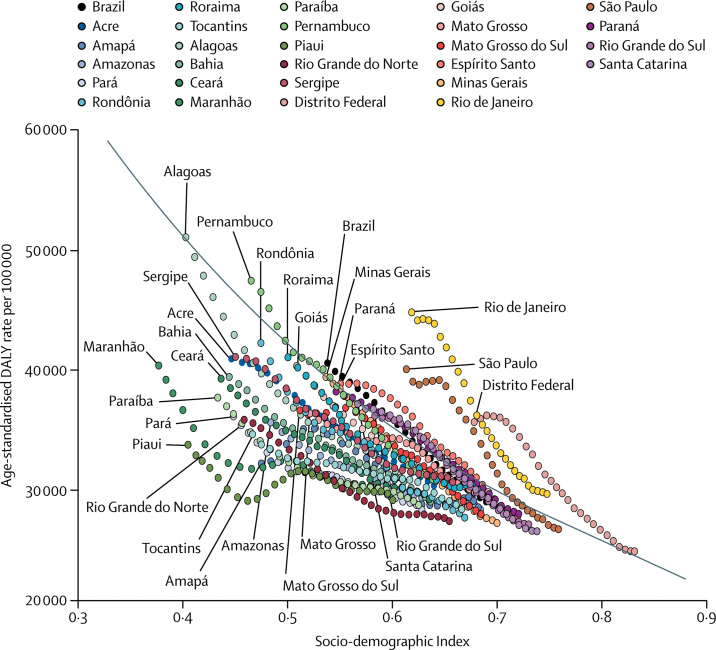
Scatter of all-cause age-standardised disability-adjusted life-year rates and Socio-demographic Index in Brazil, its states, and the Federal District, both sexes, 1990–2016 Each point represents DALY rates in a single location-year by that location's SDI in the given year, coloured by location. SDI in locations has increased year on year, so points from earlier years are associated with lower SDI in most cases. The black line indicates expected values based on SDI. DALY=disability-adjusted life-year.

During the same period, the age-standardised rate of DALYs for all causes decreased by 30·2% (95% UI 27·7–32·8), from 40 703·2 (37 686·9–44 088·9) per 100 000 to 28 424·7 (25 411·7–31 646·7) per 100 000 (table 2). Declines in all-cause age-standardised DALY rates occurred alongside rising SDI in Brazil, its states, and the Federal District (figure 3), and although states such as Piauí and Maranhão saw increases in DALY rates in some years in spite of rising SDI, many states had lower DALY rates than would be expected on the basis of SDI. The appendix (p 64) provides age-standardised DALY rates for leading GBD causes for Brazil and by state in 1990 and 2016.

**Figure 4:**
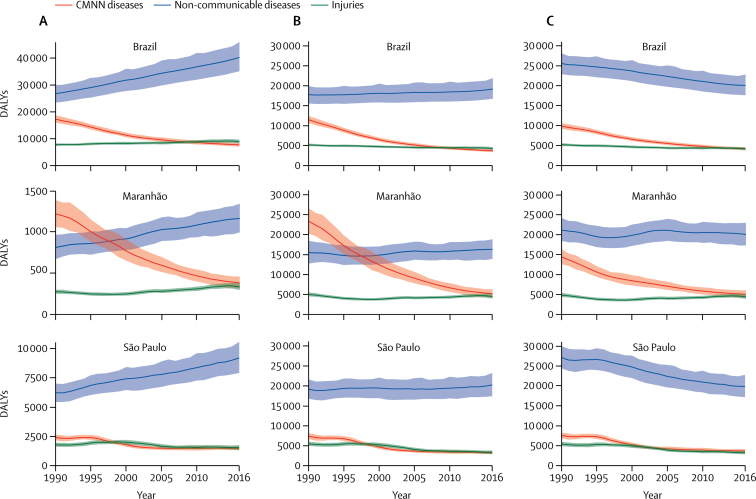
Total DALYs (A, in thousands), crude rates (B, per 100 000 people), and age-standardised rates (C, per 100 000 people), in Brazil, Maranhão, and São Paulo, both sexes, 1990–2016 DALYs=disability-adjusted life-years.

Temporal changes in DALYs for NCDs, communicable, maternal, neonatal, and nutritional (CMNN) diseases, and injuries differed within Brazil. This is evident in the comparison between the states of Maranhão and São Paulo, which have the lowest and the highest SDI scores (SDI 0·60 and 0·76, respectively), excluding the Federal District (figure 4). Between 1990 and 2016, DALYs due to NCDs increasingly predominated in all settings, with the total number of DALYs increasing and age-standardised rates decreasing, especially in São Paulo. With respect to CMNN diseases, total DALYs and crude DALY rates decreased sharply in Maranhão from 1990 to 2016, and NCDs overtook CMNN diseases as the leading contributor to total and crude DALY rates in 1998. By 2016, all DALY measures for CMNN diseases in Brazil, Maranhão, and São Paulo had declined to similar levels as for injuries, which were relatively lower and stable throughout the period.

**Figure 5:**
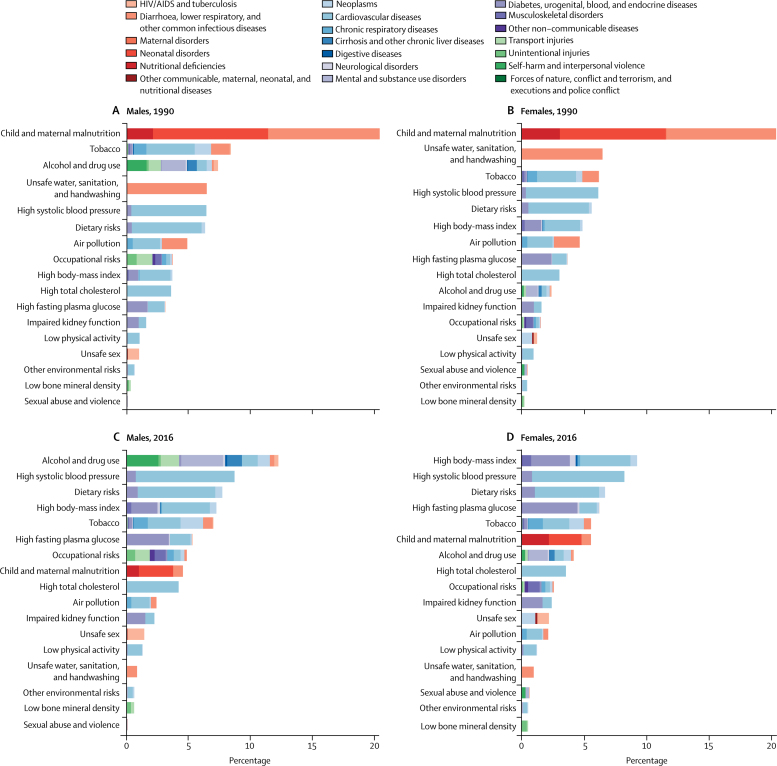
Percent contribution to DALYs by risk, all ages, Brazil, males (A), females (B) in 1990, and males (C), females (D) in 2016 DALYs=disability-adjusted life-years.

In 1990, many leading risk factors in Brazil contributed to DALYs for CMNN diseases. Child and maternal malnutrition contributed the greatest percentage of DALYs for males and females in 1990 (20·7% [95% UI 18·4–22·8] and 21·2% [18·7–24·0] respectively), and unsafe water, sanitation, and handwashing (WASH) also contributed a large percentage (6·5% [5·1–7·7] for males and 6·7% [5·3–8·2] for females) (figure 5A and 5B). These risks contributed to DALYs from diarrhoeal diseases, lower respiratory and other common infectious diseases, and maternal and neonatal disorders. By 2016, these risk factors contributed a much smaller percentage of DALYs (4·5% [4·1–4·9] for males and 5·5% [5·0–6·0] for females for child and maternal malnutrition, 0·8% [0·6–1·0] for males and 0·9% [0·9–1·1] for females for unsafe WASH; figures 5C and 5D).

In 2016, many leading risk factors were metabolic, reflecting the increasing predominance of NCDs as the cause of the disease burden. In 2016 for females, the top four risk factors were high body-mass index (BMI; 9·2% [95% UI 6·8–11·8] contribution to DALYs), high systolic blood pressure (8·2% [7·1–9·4]), dietary risks (6·7% [5·1–8·4]), and high fasting plasma glucose (6·2% [5·3–7·2]). For males, high systolic blood pressure was the second greatest contributor (8·6% [7·7–9·6]), followed by dietary risks (7·7% [6·0–9·4]), and high BMI (7·2% [4·9–9·6]). These risk factors contributed primarily to DALYs from cardiovascular diseases and diabetes, and urogenital, blood, and endocrine diseases, among others.

The behavioural risk factor of alcohol and drug use contributed to an increasing percentage of DALYs, especially for males, rising from 7·4% (95% UI 6·4–8·5) in 1990 to 12·2% (10·3–14·0) in 2016, when it was the leading risk factor for males. The percentage of DALYs contributed by tobacco decreased for both sexes, from 8·5% (7·7–9·3) for males and 6·4% (5·7–7·2) for females in 1990 to 6·9% (6·2–7·7) and 5·5% (4·8–6·3), respectively, in 2016. Alcohol and drug use contributed primarily to NCD DALYs from self-harm and interpersonal violence and mental and substance use disorders, as well as transport injuries, while tobacco mainly contributed to NCD DALYs from neoplasms, cardiovascular disease, and chronic respiratory diseases (figures 5C and 5D).

Compared with the high-income and Latin American comparator countries, Brazil had higher age-standardised rates of mortality, YLLs, YLDs, and DALYs in 1990 and 2016 (with the exception of YLDs in England in 2016) and lower LE and HALE (table 2). Of the BRICS countries, Brazil had lower rates of mortality, YLLs, YLDs, and DALYs and higher LE and HALE than Russia, India, and South Africa in 1990 and 2016, with the exception of Russia in 1990, which had lower levels of YLLs and DALYs and higher HALE and LE in that year. China had lower LE and higher mortality, YLLs, and DALYs than Brazil in 1990, but had surpassed Brazil on all health measures except DALYs by 2016. The gap between health outcomes in Brazil and Mexico and between Brazil and Argentina narrowed from 1990 to 2016, as health outcomes improved in Brazil. The appendix shows the rank of top GBD causes of age-standardised DALYs (p 66) and the ratio of observed to expected age-standardised DALY rates for leading GBD causes (p 67) in Brazil and comparator countries.

## Discussion

Although health outcomes in Brazil improved overall between 1990 and 2016, these improvements were not sufficient to eliminate health inequities: the burden of disease is generally higher in the states of the north and northeast regions than in the south and southeast. Brazil has undergone an epidemiological transition, with increasing life expectancy and decreasing mortality—largely attributable to declines in CMNN diseases—taking place alongside an increasing NCD burden. States in the south and southeast seem to be in later stages of the epidemiological transition towards NCDs compared with states in the north and northeast, and some states in earlier stages of the transition are facing a double burden of CMNN diseases and NCDs, alongside a growing burden due to injuries across the country. Leading risk factors now contribute primarily to the NCD burden, and Brazil is also contending with increased burdens of violence and road traffic injuries. Although these growing burdens have not yet negatively affected trends in life expectancy, they may shape future patterns if not addressed.

Although health outcomes improved in states in the north and northeast, higher rates of YLLs and YLDs persist in many states in those regions compared with states in the south and southeast, and LE and HALE remain generally higher in the south and southeast. These results are likely to reflect overall socioeconomic improvements, as reflected in increasing SDIs across all states, that have not mitigated socioeconomic disparities in access to and quality of health care. Of note, the comparatively good health indicators observed in the Federal District may be partially explained by the fact that poorer communities and slums surrounding Brasilia are not included in the official district population counts. The significant gains in some northern states in LE and HALE led to greater convergence between regions; however, these gains primarily reflect a reduction in the burden of CMNN diseases rather than NCDs. Access to health care in Brazil has become more equal since 1998,^26^ which may have contributed to this convergence.

Access to health care improved with the creation of the tax-financed national unified health system (SUS).^1^ Under the SUS, coverage of public health interventions rapidly expanded, including immunisation, family planning, and prenatal and maternal care.^1^ The Family Health Strategy has been associated with reduced neonatal and child mortality even after accounting for the *Bolsa Familia* cash transfer programme, as well as with reduced mortality from stroke and heart disease.^27^, ^28^ Exclusive breastfeeding rates in children under 4 months also increased from 4% to 48% from the mid-1980s to the mid-2000s.^29^ However, the SUS is not the sole explanation for improved health outcomes. Over the same period, Brazil experienced dramatic economic growth, accompanied by increases in the value of the minimum wage and increasing wealth distribution.^7^, ^30^ The government also invested in education, social protection, and sanitation, all of which affect health and wellbeing. At the same time, the private health sector has played and continues to play a major role for those that can afford to use it, likely dictating some of the variation in health across populations.^30^

In 2016, more than 95% of children in Brazil received most scheduled childhood vaccinations, including vaccines for measles, rubella, and rotavirus.^31^, ^32^ The successful expansion of coverage from 1990 has likely contributed greatly to the declines in vaccine preventable CMNN diseases and child mortality.^33^ However, high levels of preterm births in Brazil may be contributing to the large burden of YLLs from neonatal conditions.^34^, ^35^ Improvements to newborn care services may have partially offset this increasing burden, but more remains to be done.^36^, ^37^, ^38^ Maintaining gains in infectious disease control will also be a challenge in the face of endemic and emerging vector-borne diseases such as dengue, chikungunya, and Zika virus.^39^ In addition, although Brazil has implemented numerous measures to combat HIV,^40^ including free universal access to essential medicines and antiretroviral treatment,^41^, ^42^ the increasing number of YLLs due to HIV indicate a need for increased efforts.

Ischaemic heart disease and stroke have been the leading causes of death in Brazil since the end of the 1960s, due in part to lifestyle changes related to urbanisation and globalisation.^43^ The shift toward NCDs occurred earlier in the south and southeast, as highlighted by the distinct time trends in disease burden between Maranhão and São Paulo. However, many states in the north and northeast are moving towards similar trends for NCDs, and had the highest rates for important NCDs in 2016.^44^ This indicates that states in the south and southeast have been able to improve health efforts to address the NCD burden, while states in the north and northeast have not yet been able to achieve the same success. Indeed, variation in NCD burden can occur within states and cities,^45^ and policies will need to address this diversity. Successful efforts to prevent and control NCDs might enable states in the north and northeast to avoid the pitfalls of the epidemiological transition towards chronic disease.

The increasing life expectancy in Brazil brings additional health challenges: as individuals live longer with some form of disability, rates of non-fatal health loss increase, resulting in an absolute expansion of morbidity.^46^ São Paulo is a particular example: the state had very low rates of YLLs in 2016 compared with others, but one of the highest rates of YLDs. Population ageing also partially explains the increases in total YLDs (due to a growing elderly population) experienced alongside decreases in age-standardised YLD rates for some diseases (due to declining disease incidence or prevalence). Overall, the burden of chronic conditions, such as diabetes and hypertension, is growing.^44^, ^47^ Interventions targeting risk factors for NCDs and other chronic diseases might help address this concern.

The risk landscape also shifted from CMNN diseases towards NCDs and, to a lesser extent, injuries. Controlling the growing NCD burden will require attention to diet and physical activity.^47^ The prevalence of obesity has increased by 60% among those aged 25 to 34 years since 2006,^48^ and was 17% in 2016,^49^ even as some populations continue to suffer from undernutrition and malnourishment. The Brazilian Government has enacted some policies to combat these risks, including the Plano de Enfrentamento das DCNTs, the Nutritional Guidelines for the Brazilian Population, and the WHO Global Strategy on Diet, Physical Activity and Health, aiming to prevent obesity, tobacco use, and NCDs.^43^, ^50^, ^51^ Since 2006, a national system has monitored risk factors for NCDs through telephone interviews.^49^ In 2013, the National Health Survey showed that older age, less schooling, and the male sex were associated with less physical activity, prevalence of daily smoking, and low consumption of fruits and vegetables—highlighting the increased potential risk for this demographic.^52^, ^53^

Many efforts have contributed to the decline in DALY rates from 1990 to 2016 and should be considered a priority, including improved access to primary care and care for related risk factors, such as hypertension; access to free or subsidised drugs for hypertension, diabetes, and asthma; and access to and prioritisation of care for acute cardiovascular events.^4^, ^54^, ^55^, ^56^ Tackling ischaemic heart disease poses a double challenge to health systems: prevention must take place alongside improvements in health-care delivery, particularly in public hospitals.^54^ Policies restricting salt could further reduce rates of cardiovascular disease and stroke.^57^ The potential impact of new technologies to detect and treat NCDs is also substantial.

Since 2006, the National Health Promotion Policy has introduced initiatives to reduce behavioural risk factors such as smoking.^58^, ^59^ Despite being the second greatest producer of tobacco worldwide, Brazil's “National Tobacco Control Program” has successfully reduced tobacco consumption,^54^ distinguishing Brazil from many other low-income and middle-income countries, which have seen increasing trends.^60^ However, rates declined more slowly for males and in lower socioeconomic populations,^54^ and rates of smoking are still much higher in males; overall, close to 4 million more males than females smoke in Brazil.^61^ Tobacco control efforts in Brazil have included national and state level smoke-free air laws; packaging, marketing, and age restrictions; minimum pricing and taxation; cessation treatment; and behaviour change campaigns.^62^, ^63^ The success of these programmes, even when confronted with industry interests, indicates that Brazil could be well positioned to introduce similar fiscal policies and restrictions to reduce consumption of sugar, unhealthy foods, and alcohol.

Alcohol consumption, economic growth, and urbanisation are among the factors that have contributed to the high DALY burden of road injuries in Brazil, particularly among young males and their main victims, pedestrians.^64^, ^65^ The increasing use of motorcycles has also contributed.^66^, ^67^ Careless riding, speeding, and increased vulnerability of the driver and passenger have led to an increase in the number of motorcycle accidents, especially among young males.^66^ Motorcycle drivers are a priority for current injury prevention programmes.^10^, ^66^ High DALY rates are likely due in part to rapid urbanisation, low quality of roads, and lack of access to and quality of health-care services, particularly in rural areas,^68^ potentially influencing the disparate rates between regions. Traffic safety laws, including car safety requirements and zero tolerance on alcohol consumption, have been introduced, but many individuals continue to self-report consumption levels above legal limits while driving.^68^

Alcohol also contributes to the DALY burden due to interpersonal violence, one of the leading causes of DALYs in 2016. Violence primarily affects younger individuals,^10^ thereby contributing to a higher number of YLLs and YLDs. Much of the interpersonal violence in Brazil is due to homicides with firearms related in part to drug trafficking, availability and circulation of illegal firearms, and consumption of alcohol and drugs.^10^, ^11^ Mortality rates are especially high for young males, as is the case in other countries.^17^ Although rates remain high in Rio de Janeiro, they represent an impressive decline from 1990, in part due to national policies introduced in 2003 that restricted firearm ownership, carrying, and importation, and increased punishments for violations, alongside disarmament efforts.^69^, ^70^ However, increases in the burden of interpersonal violence in the majority of states indicate a potential disparity in policy enforcement, among other factors. The historic relationship between police, crime, and communities in Brazil is complex,^10^ and focusing on the top risk factors contributing to interpersonal violence—alcohol and drug use—could be a more productive way to address the problem than increased policing, particularly among males.^71^ However, efforts will also need to take into account the role of the drug trade, which has led to increased rates of violence across a belt of countries in Latin America, including comparators Mexico and Colombia.^17^, ^72^

To continue to positively affect the changing health landscape, financing in Brazil must reflect the geographic-specific burden of disease and focus on “best buys” for NCD control.^73^ Public financing of the health system has been a constitutional right since 1988. Brazil's investment in health has increased over the past 20 years, and it currently invests US$947 per capita and 8·3% of GDP in health.^74^ This is a much lower sum than countries with public health systems (eg, Australia and England) invest, but similar to investments made in Argentina, Colombia, and Mexico in recent years.^74^

The challenge ahead is to ensure that financial resources in Brazil are strategically allocated according to disease priorities in each state, region, sex, and age group. For example, although the burden of ischaemic heart disease is highest in the northeast region, inequalities exist in public financing of hospital admissions for heart disease.^54^, ^75^ In 2012, the median per capita outlay for adults aged 40 years or older in the northern and northeastern regions was $6·07 and $10·28, respectively; considerably less than the median per capita outlay in states of the southern region of $20·32.^2^

Finally, while increased funds are required to maintain gains and further improve health and equality, congress approved Constitutional Amendment 95 in December, 2016, restricting funds allocated to the health sector and providing no real increase in health funding for the next 20 years—until 2037.^76^ This austerity has also extended to other sectors affecting health and wellbeing, including education and public utilities such as sanitation. These policies could stall the important progress that has been made over the past 26 years in Brazil, while the ultimate cost-saving potential of reducing deaths and injuries from violence, road injuries, and other diseases is considerable.^10^

### Limitations and future directions

This study is subject to all the limitations of the GBD 2016 study, which have been previously described.^5^, ^17^, ^19^, ^20^, ^21^, ^22^ Cause of death data completeness at the state level in Brazil is quite high, with all states receiving at least four stars in the period 2010 to 2016, and at least one state in each region receiving five stars (table 1). This represents a substantial improvement from 1980, when many states in the northeast had a rating of just two stars. Because our first cause of death data point in Tocantins is from 1993, Tocantins received zero stars for the periods 1980–84 and 1985–89. Despite GBD methods to correct under-registered deaths, we dropped 27 years of state data prior to 1998 due to low completeness or low quality.

Data on morbidity in some northern states in 1990 may not be reliable due to small population sizes (about 300 000) and poor information quality. Data from recent years are likely to be more reliable, but data sparsity and under-reporting or incorrect reporting of morbidity can present major challenges to the production of accurate estimates, particularly at the state level. For some causes of morbidity, we did not have data at the state level, and results for these causes were based on country or regional level results. Sparsity of survey data at the state level is the biggest challenge for risk factor and morbidity estimation.

The lack of a harmonised system to connect different data sources has impeded the comprehensive comparison of temporal trends and inter-region variability. Further research and increased data collection is needed to complete the picture of health in Brazil, particularly for conditions with very limited data, such as mental health. Improving the quality of registered data will also improve the validity of both national and GBD results, which have not been extensively compared. Further research in this area can fill knowledge gaps and guide health system planning at the state level.

## Conclusion

Brazil has improved population health over the past 26 years despite an ageing population, increasing NCD burden, and rising costs of care. The public health system expanded, the economy grew, and policies were introduced focusing on risk factors for chronic diseases, prevention, and health promotion. The remaining challenge will be to address the growing NCD burden and remaining regional health inequalities. The current distribution of health resources must be realigned to efficiently address changing geographic-specific burdens. This study presents a thorough analysis of health inequalities and shows that overall, health outcomes are better in the south and southeast compared with the north and northeast. These findings can help to identify challenges, successes, and research gaps that policy makers can use to improve the health of all Brazilians and preserve gains in the face of rising poverty in the current recession.^77^

Correspondence to: Prof Mohsen Naghavi, Institute for Health Metrics and Evaluation, University of Washington, Seattle, WA 98121, USA **nagham@uw.edu**

## References

[1] Paim J, Travassos C, Almeida C, Bahia L, Macinko J (2011). The Brazilian health system: history, advances, and challenges. Lancet.

[2] Paim JS (2013). A Constituição Cidadã e os 25 anos do Sistema Único de Saúde (SUS). Cad Saúde Pública.

[3] Homma A, Tanuri A, Duarte AJS (2013). Vaccine research, development, and innovation in Brazil: a translational science perspective. Vaccine.

[4] Escorel S, Giovanella L, Magalhães de Mendonça MH, de Castro Maia Senna M (2007). The Family Health Program and the construction of a new model for primary care in Brazil. Rev Panam Salud Publica.

[5] Naghavi M, Abajobir AA, Abbafati C (2017). Global, regional, and national age-sex specific mortality for 264 causes of death, 1980–2016: a systematic analysis for the Global Burden of Disease Study 2016. Lancet.

[6] Vincens N, Stafström M (2015). Income inequality, economic growth and stroke mortality in brazil: longitudinal and regional analysis 2002–2009. PLoS One.

[7] Carvalho L, Rugitsky F Growth and distribution in Brazil in the 21st century: revisiting the wage-led versus profit-led debate. São Paulo: University of São Paulo. https://www.boeckler.de/pdf/v_2015_10_23_carvalho.pdf.

[8] Wong LLR, Carvalho JA (2006). The rapid process of aging in Brazil: serious challenges for public policies. Rev Bras Estud Popul.

[9] Instituto Brasileiro de Geografia e Estatística https://ww2.ibge.gov.br/english/.

[10] Reichenheim ME, de Souza ER, Moraes CL, de Mello Jorge MH, da Silva CM, de Souza Minayo MC (2011). Violence and injuries in Brazil: the effect, progress made, and challenges ahead. Lancet.

[11] Malta DC, de Souza Minayo MC, Soares Filho AM (2017). Mortality and years of life lost by interpersonal violence and self-harm: in Brazil and Brazilian states: analysis of the estimates of the Global Burden of Disease Study, 1990 and 2015. Rev Bras Epidemiol.

[12] Nomura S, Sakamoto H, Glenn S (2017). Population health and regional variations of disease burden in Japan, 1990–2015: a systematic subnational analysis for the Global Burden of Disease Study 2015. Lancet.

[13] Gómez-Dantés H, Fullman N, Lamadrid-Figueroa H (2016). Dissonant health transition in the states of Mexico, 1990–2013: a systematic analysis for the Global Burden of Disease Study 2013. Lancet.

[14] Newton JN, Briggs ADM, Murray CJL (2015). Changes in health in England, with analysis by English regions and areas of deprivation, 1990–2013: a systematic analysis for the Global Burden of Disease Study 2013. Lancet.

[15] Zhou M, Wang H, Zhu J (2016). Cause-specific mortality for 240 causes in China during 1990–2013: a systematic subnational analysis for the Global Burden of Disease Study 2013. Lancet.

[16] Murray CJ, Ezzati M, Flaxman AD (2012). GBD 2010: design, definitions, and metrics. Lancet.

[17] Wang H, Abajobir AA, Abate KH (2017). Global, regional, and national under-5 mortality, adult mortality, age-specific mortality, and life expectancy, 1970–2016: a systematic analysis for the Global Burden of Disease Study 2016. Lancet.

[18] Stevens GA, Alkema L, Black RE (2016). Guidelines for Accurate and Transparent Health Estimates Reporting: the GATHER statement. Lancet.

[19] Vos T, Abajobir AA, Abate KH (2017). Global, regional, and national incidence, prevalence, and years lived with disability for 328 diseases and injuries for 195 countries, 1990–2016: a systematic analysis for the Global Burden of Disease Study 2016. Lancet.

[20] Hay SI, Abajobir AA, Abate KH (2017). Global, regional, and national disability-adjusted life-years (DALYs) for 333 diseases and injuries and healthy life expectancy (HALE) for 195 countries and territories, 1990–2016: a systematic analysis for the Global Burden of Disease Study 2016. Lancet.

[21] Gakidou E, Afshin A, Abajobir AA (2017). Global, regional, and national comparative risk assessment of 84 behavioural, environmental and occupational, and metabolic risks or clusters of risks, 1990–2016: a systematic analysis for the Global Burden of Disease Study 2016. Lancet.

[22] Fullman N, Barber RM, Abajobir AA (2017). Measuring progress and projecting attainment on the basis of past trends of the health-related Sustainable Development Goals in 188 countries: an analysis from the Global Burden of Disease Study 2016. Lancet.

[23] (2017). Global Burden of Disease 2015 study: summary of methods used. Rev Bras Epidemiol.

[24] Salomon JA, Haagsma JA, Davis A (2015). Disability weights for the Global Burden of Disease 2013 study. Lancet Glob Health.

[25] BRICS Building responsive, inclusive & collective solutions. http://brics2016.gov.in/content/innerpage/about-usphp.php.

[26] Macinko J, Lima-Costa MF (2012). Horizontal equity in health care utilization in Brazil, 1998–2008. Int J Equity Health.

[27] Bastos ML, Menzies D, Hone T, Dehghani K, Trajman A (2017). The impact of the Brazilian family health on selected primary care sensitive conditions: a systematic review. PLoS One.

[28] Rasella D, Harhay MO, Pamponet ML, Aquino R, Barreto ML (2014). Impact of primary health care on mortality from heart and cerebrovascular diseases in Brazil: a nationwide analysis of longitudinal data. BMJ.

[29] Victora CG, Aquino EM, do Carmo Leal M, Monteiro CA, Barros FC, Szwarcwald CL (2011). Maternal and child health in Brazil: progress and challenges. Lancet.

[30] Victora CG, Barreto ML, do Carmo Leal M (2011). Health conditions and health-policy innovations in Brazil: the way forward. Lancet.

[31] WHO, UNICEF (2017). Brazil: WHO and UNICEF estimates of immunization coverage: 2016 revision. https://data.unicef.org/wp-content/uploads/country_profiles/Brazil/immunization_country_profiles/immunization_bra.pdf.

[32] WHO (April, 2017). From warehouse to remote indigenous communities: the journey of vaccines in Brazil. http://www.who.int/features/2017/vaccines-brazil-communities/en/.

[33] Lanzieri TM, Linhares AC, Costa I (2011). Impact of rotavirus vaccination on childhood deaths from diarrhea in Brazil. Int J Infect Dis.

[34] Leal M do C, Esteves-Pereira AP, Nakamura-Pereira M (2016). Prevalence and risk factors related to preterm birth in Brazil. Reprod Health.

[35] Blencowe H, Cousens S, Oestergaard MZ (2012). National, regional, and worldwide estimates of preterm birth rates in the year 2010 with time trends since 1990 for selected countries: a systematic analysis and implications. Lancet.

[36] do Carmo Leal M, Esteves-Pereira AP, Nakamura-Pereira M (2016). Provider-initiated late preterm births in Brazil: differences between public and private health services. PLoS One.

[37] Barros FC, Matijasevich A, Requejo JH (2010). Recent trends in maternal, newborn, and child health in Brazil: progress toward Millennium Development Goals 4 and 5. Am J Public Health.

[38] Aquino R, de Oliveira NF, Barreto ML (2009). Impact of the family health program on infant mortality in Brazilian municipalities. Am J Public Health.

[39] Ali S, Gugliemini O, Harber S (2017). Environmental and Social Change Drive the Explosive Emergence of Zika Virus in the Americas. PLoS Negl Trop Dis.

[40] Carneiro-Proietti AB, Sabino EC, Sampaio D (2010). Demographic profile of blood donors at three major Brazilian blood centers: results from the International REDS-II study, 2007 to 2008. Transfusion (Paris).

[41] Galvão J (2002). Access to antiretroviral drugs in Brazil. Lancet.

[42] Ministério da Saúde (October 30, 1998). Portfolio no. 3,916 of. http://bvsms.saude.gov.br/bvs/saudelegis/gm/1998/prt3916_30_10_1998.html.

[43] Barreto SM, de Oliveira Pinheiro AR, Sichieri R (2005). Análise da estratégia global para alimentação, atividade física e saúde, da Organização Mundial da Saúde. Epidemiol E Serviços Saúde.

[44] Duncan BB, Schmidt MI, Ewerton Cousin (2017). The burden of diabetes and hyperglycemia in Brazil-past and present: findings from the Global Burden of Disease Study 2015. Diabetol Metab Syndr.

[45] Brant LCC, Nascimento BR, Passos VMA (2017). Variations and particularities in cardiovascular disease mortality in Brazil and Brazilian states in 1990 and 2015: estimates from the Global Burden of Disease. Rev Bras Epidemiol.

[46] Fries JF (1980). Aging, natural death, and the compression of morbidity. N Engl J Med.

[47] Malta DC, da Silva JB (2012). Policies to promote physical activity in Brazil. Lancet.

[48] Ministério da Saúde (2007). Vigitel Brasil 2006: vigilância de fatores de risco e proteção Pará doenças crônicas por inquérito telefônico. http://bvsms.saude.gov.br/bvs/publicacoes/vigitel_brasil_2006.pdf.

[49] Ministério da Saúde (2017). Vigitel Brazil 2016 Private Health Insurance and Plans Beneficiaries: protective and risk factors for chronic diseases by telephone survey.

[50] Monteiro CA, Cannon G, Moubarac J-C (2015). Dietary guidelines to nourish humanity and the planet in the twenty-first century. A blueprint from Brazil. Public Health Nutr.

[51] Malta DC, Morais Neto OL, Silva JB (2011). Presentation of the strategic action plan for coping with chronic diseases in Brazil from 2011 to 2022. Epidemiol Serv Saúde.

[52] de Azevedo Barros MB, Lima MG, de Paula Barbosa Medina L, Szwarcwald CL, Malta DC (2016). Social inequalities in health behaviors among Brazilian adults: National Health Survey, 2013. Int J Equity Health.

[53] Malta DC, Bernal RTI, de Souza MF, Szwarcwald CL, Lima MG, Barros MB de A (2016). Social inequalities in the prevalence of self-reported chronic non-communicable diseases in Brazil: national health survey 2013. Int J Equity Health.

[54] Ribeiro ALP, Duncan BB, Brant LCC, Lotufo PA, Mill JG, Barreto SM (2016). Cardiovascular health in Brazil: trends and perspectives. Circulation.

[55] Emmerick ICM, do Nascimento JM, Pereira MA, Luiza VL, Ross-Degnan D (2015). Farmácia Popular Program: changes in geographic accessibility of medicines during ten years of a medicine subsidy policy in Brazil. J Pharm Policy Pract.

[56] Costa KS, Francisco PMSB, Malta DC, de Azevedo Barros MB (2016). Sources of medicines for hypertension and diabetes in Brazil: telephone survey results from Brazilian state capitals and the Federal District, 2011. Cad Saúde Pública.

[57] Nilson EAF, Spaniol AM, Gonçalves VSS (2017). Sodium reduction in processed foods in Brazil: analysis of food categories and voluntary targets from 2011 to 2017. Nutrients.

[58] PNUD, Brazilian Cooperation Agency Promoção da saúde: um novo modelo de atenção. http://pesquisa.bvsalud.org/bvsms/resource/pt/mis-3561.

[59] Ministério da Saúde (2006). National Health Promotion Policy: PNPS/Brazil: revision of the MS/GM ordinance no 687, of March 30th. http://bvsms.saude.gov.br/bvs/publicacoes/pnps_revisao_portaria_687.pdf.

[60] Cavalcante TM, Marques de Pinho MC, de Abreu Perez C (2017). Brazil: balance of the National Tobacco Control Policy in the last decade and dilemmas. Cad Saude Publica.

[61] Reitsma MB, Fullman N, Ng M (2017). Smoking prevalence and attributable disease burden in 195 countries and territories, 1990–2015: a systematic analysis from the Global Burden of Disease Study 2015. Lancet.

[62] Levy D, de Almeida LM, Szklo A (2012). The Brazil simsmoke policy simulation model: the effect of strong tobacco control policies on smoking prevalence and smoking-attributable deaths in a middle income nation. PLoS Med.

[63] WHO (2017). WHO report on the global tobacco epidemic, 2017: Country profile, Brazil. http://www.who.int/tobacco/surveillance/policy/country_profile/bra.pdf?ua=1.

[64] Malta DC, Bernal RTI, Mascarenhas MDM, da Silva MMA, Szwarcwald CL, de Morais Neto OL (2015). Alcohol consumption and driving in Brazilian capitals and Federal District according to two national health surveys. Rev Bras Epidemiol.

[65] Ladeira RM, Malta DC, de Morais Neto OL (2017). Road traffic accidents: Global Burden of Disease study, Brazil and federated units, 1990 and 2015. Rev Bras Epidemiol.

[66] Bacchieri G, Barros AJD (2011). Traffic accidents in Brazil from 1998 to 2010: many changes and few effects. Rev Saúde Pública.

[67] Instituto Brasileiro de Geografia e Estatística (2016). Frota municipal de veículos no Brasil. https://cidades.ibge.gov.br/painel/frota.php.

[68] Blumenberg C, Martins RC, Calu Costa J, Ricardo LIC (2017). Is Brazil going to achieve the road traffic deaths target? An analysis about the sustainable development goals. Inj Prev.

[69] Murray J, de Castro Cerqueira DR, Kahn T (2013). Crime and violence in Brazil: systematic review of time trends, prevalence rates and risk factors. Aggress Violent Behav.

[70] de Fátima Marinho de Souza M, Macinko J, Alencar AP, Malta DC, de Morais Neto OL (2007). Reductions in firearm-related mortality and hospitalizations in Brazil after gun control. Health Aff (Millwood).

[71] Malta DC, Campos MO, de Oliveira MM (2015). Noncommunicable chronic disease risk and protective factor prevalence among adults in Brazilian state capital cities, 2013. Epidemiol E Serviços Saúde.

[72] Lotto Persio S Drug war turned Mexico into world's deadliest conflict zone after only Syria: survey. Newsweek. http://www.newsweek.com/mexicos-drugs-war-created-worlds-deadliest-conflict-zone-after-syria-survey-606558.

[73] World Economic Forum, World Health Organization (2011). From burden to ‘best buys’: reducing the economic impact of non-communicable diseases in low- and middle-income countries.

[74] WHO (June 19, 2018). Global Health Expenditure Database. http://apps.who.int/nha/database.

[75] Lotufo PA, Benseñor IM (2009). Stroke mortality in Brazil: one example of delayed epidemiological cardiovascular transition. Int J Stroke.

[76] (2016). Brasil. Legislação Federal do Brasil. http://legislacao.planalto.gov.br/legisla/legislacao.nsf/Viw_Identificacao/emc%2095-2016?OpenDocument.

[77] Skoufias E (Feb 24, 2017). Protecting the “new poor” during Brazil's economic crisis. World Bank Latin America & Caribbean Opportunities For All. http://blogs.worldbank.org/latinamerica/protecting-new-poor-during-brazil-s-economic-crisis.

