# Supplementary material for: Burden of disease in Brazil, 1990–2016: a systematic subnational analysis for the Global Burden of Disease Study 2016
Source: Lancet. 2018 Sep 1;392(10149):760–75. doi: 10.1016/S0140-6736(18)31221-2 (PMC6123514; doi:10.1016/S0140-6736(18)31221-2)
Supplement: Supplementary appendix [file mmc1.pdf]

THE LANCET

Supplementary appendix

This appendix formed part of the original submission and has been peer reviewed. We post it as supplied by the authors.

Supplement to: GBD 2016 Brazil Collaborators. Burden of disease in Brazil, 1990–2016: a systematic subnational analysis for the Global Burden of Disease Study 2016. *Lancet* 2018; published online July 20. http://dx.doi.org/10.1016/S0140-6736(18)31221-2.

Appendix Table 1: Total number of site years by cause and source type, cause of death estimates, 1990-2016, Brazil								
Level	Cause	Vital Registration	Verbal Autopsy	Surveillance	Sibling History	Survey/Census	Cancer Registry	Police Records
0	All causes	683	2		196		252	
1	Communicable, maternal, neonatal, and nutritional diseases	683	2		196			
2	HIV/AIDS and tuberculosis	683						
3	Tuberculosis	683						
4	Drug-susceptible tuberculosis	683						
4	Multidrug-resistant tuberculosis without extensive drug resistance	545						
4	Extensively drug-resistant tuberculosis							
3	HIV/AIDS	683						
4	Drug-susceptible HIV/AIDS - Tuberculosis	546						
4	Multidrug-resistant HIV/AIDS - Tuberculosis without extensive drug resistance							
4	Extensively drug-resistant HIV/AIDS - Tuberculosis							
4	HIV/AIDS resulting in other diseases	683						
2	Diarrhea, lower respiratory, and other common infectious diseases	683	2					
3	Diarrheal diseases	683	2					
3	Intestinal infectious diseases	682						
4	Typhoid fever	682						
4	Paratyphoid fever	682						
4	Other intestinal infectious diseases	682						
3	Lower respiratory infections	683	2					
3	Upper respiratory infections	682						
3	Otitis media	680						
3	Meningitis	683	1					
4	Pneumococcal meningitis	683						
4	H influenzae type B meningitis	683						
4	Meningococcal meningitis	683						
4	Other meningitis	683						
3	Encephalitis	683						
3	Diphtheria	672						
3	Whooping cough	677						
3	Tetanus	682	1					
3	Measles	683						
3	Varicella and herpes zoster	683						
2	Neglected tropical diseases and malaria	683						
3	Malaria	680						
3	Chagas disease	683						
3	Leishmaniasis	682						
4	Visceral leishmaniasis	682						
3	African trypanosomiasis	679						
3	Schistosomiasis	680						
3	Cysticercosis	680						
3	Cystic echinococcosis	680						
3	Dengue	680						
3	Yellow fever	680						
3	Rabies	680						
3	Intestinal nematode infections	682						
4	Ascariasis	682						
3	Ebola	3						
3	Zika virus	545						
3	Other neglected tropical diseases	680						
2	Maternal disorders	683			196			

3	Maternal hemorrhage	680						
3	Maternal sepsis and other maternal infections	681						
3	Maternal hypertensive disorders	678						
3	Maternal obstructed labor and uterine rupture	680						
3	Maternal abortion, miscarriage, and ectopic pregnancy	682						
3	Indirect maternal deaths	649						
3	Late maternal deaths	504						
3	Maternal deaths aggravated by HIV/AIDS	683			196			
3	Other maternal disorders	681						
2	Neonatal disorders	683	2					
3	Neonatal preterm birth complications	683	1					
3	Neonatal encephalopathy due to birth asphyxia and trauma	683	1					
3	Neonatal sepsis and other neonatal infections	682						
3	Hemolytic disease and other neonatal jaundice	683						
3	Other neonatal disorders	683						
2	Nutritional deficiencies	683	1					
3	Protein-energy malnutrition	683						
3	Iodine deficiency	127						
3	Iron-deficiency anemia	683						
3	Other nutritional deficiencies	683						
2	Other communicable, maternal, neonatal, and nutritional diseases	683	1					
3	Sexually transmitted diseases excluding HIV	683						
4	Syphilis	680						
4	Chlamydial infection	683						
4	Gonococcal infection	683						
4	Other sexually transmitted diseases	683						
3	Hepatitis	682	1					
4	Acute hepatitis A	682						
4	Hepatitis B	681						
4	Hepatitis C	679						
4	Acute hepatitis E	679						
3	Other infectious diseases	683						
1	Non-communicable diseases	683	2				252	
2	Neoplasms	683					252	
3	Lip and oral cavity cancer	683					245	
3	Nasopharynx cancer	683					223	
3	Other pharynx cancer	683					242	
3	Esophageal cancer	683					245	
3	Stomach cancer	683					247	
3	Colon and rectum cancer	683					247	
3	Liver cancer	683					246	
4	Liver cancer due to hepatitis B							
4	Liver cancer due to hepatitis C							
4	Liver cancer due to alcohol use							
4	Liver cancer due to other causes							
3	Gallbladder and biliary tract cancer	683					242	
3	Pancreatic cancer	683					244	
3	Larynx cancer	683					244	
3	Tracheal, bronchus, and lung cancer	683					247	

3	Malignant skin melanoma	683					247	
3	Non-melanoma skin cancer	683						
4	Non-melanoma skin cancer (squamous-cell carcinoma)	683						
3	Breast cancer	683					247	
3	Cervical cancer	683					247	
3	Uterine cancer	683					246	
3	Ovarian cancer	683					247	
3	Prostate cancer	683					247	
3	Testicular cancer	683					233	
3	Kidney cancer	683					244	
3	Bladder cancer	683					244	
3	Brain and nervous system cancer	683					246	
3	Thyroid cancer	683					244	
3	Mesothelioma	546						
3	Hodgkin lymphoma	683					241	
3	Non-Hodgkin lymphoma	683					246	
3	Multiple myeloma	683					239	
3	Leukemia	683					252	
4	Acute lymphoid leukemia	683					243	
4	Chronic lymphoid leukemia	683					230	
4	Acute myeloid leukemia	683					239	
4	Chronic myeloid leukemia	683					242	
4	Other leukemia	683					241	
3	Other neoplasms	683					252	
2	Cardiovascular diseases	683						
3	Rheumatic heart disease	683						
3	Ischemic heart disease	683						
3	Cerebrovascular disease	683						
4	Ischemic stroke	683						
4	Hemorrhagic stroke	683						
3	Hypertensive heart disease	683						
3	Cardiomyopathy and myocarditis	683						
4	Myocarditis	683						
4	Alcoholic cardiomyopathy	683						
4	Other cardiomyopathy	683						
3	Atrial fibrillation and flutter	680						
3	Aortic aneurysm	683						
3	Peripheral artery disease	675						
3	Endocarditis	683						
3	Other cardiovascular and circulatory diseases	683						
2	Chronic respiratory diseases	683						
3	Chronic obstructive pulmonary disease	683						
3	Pneumoconiosis	682						
4	Silicosis	681						
4	Asbestosis	682						
4	Coal workers pneumoconiosis	681						
4	Other pneumoconiosis	681						
3	Asthma	682						
3	Interstitial lung disease and pulmonary sarcoidosis	682						
3	Other chronic respiratory diseases	683						
2	Cirrhosis and other chronic liver diseases	683						
3	Cirrhosis and other chronic liver diseases due to hepatitis B							
3	Cirrhosis and other chronic liver diseases due to hepatitis C							
3	Cirrhosis and other chronic liver diseases due to alcohol use							

3	Cirrhosis and other chronic liver diseases due to other causes								
2	Digestive diseases	683							
3	Peptic ulcer disease	683							
3	Gastritis and duodenitis	683							
3	Appendicitis	683							
3	Paralytic ileus and intestinal obstruction	683							
3	Inguinal, femoral, and abdominal hernia	683							
3	Inflammatory bowel disease	683							
3	Vascular intestinal disorders	682							
3	Gallbladder and biliary diseases	683							
3	Pancreatitis	683							
3	Other digestive diseases	683							
2	Neurological disorders	683							
3	Alzheimer disease and other dementias	678							
3	Parkinson disease	679							
3	Epilepsy	682							
3	Multiple sclerosis	572							
3	Motor neuron disease	681							
3	Other neurological disorders	683							
2	Mental and substance use disorders	683							
3	Alcohol use disorders	683							
3	Drug use disorders	683							
4	Opioid use disorders	683							
4	Cocaine use disorders	682							
4	Amphetamine use disorders	682							
4	Other drug use disorders	682							
3	Eating disorders	678							
4	Anorexia nervosa	678							
4	Bulimia nervosa	665							
2	Diabetes, urogenital, blood, and endocrine diseases	683							
3	Diabetes mellitus	683							
3	Acute glomerulonephritis	683							
3	Chronic kidney disease	683							
4	Chronic kidney disease due to diabetes mellitus	683							
4	Chronic kidney disease due to hypertension	683							
4	Chronic kidney disease due to glomerulonephritis	683							
4	Chronic kidney disease due to other causes	683							
3	Urinary diseases and male infertility	683							
4	Interstitial nephritis and urinary tract infections	683							
4	Urolithiasis	683							
4	Other urinary diseases	683							
3	Gynecological diseases	683							
4	Uterine fibroids	683							
4	Polycystic ovarian syndrome	591							
4	Endometriosis	613							
4	Genital prolapse	663							
4	Other gynecological diseases	683							
3	Hemoglobinopathies and hemolytic anemias	683							
4	Thalassemias	683							
4	Sickle cell disorders	683							
4	G6PD deficiency	59							
4	Other hemoglobinopathies and hemolytic anemias	681							

3	Endocrine, metabolic, blood, and immune disorders	683						
2	Musculoskeletal disorders	683						
3	Rheumatoid arthritis	681						
3	Other musculoskeletal disorders	683						
2	Other non-communicable diseases	683	2					
3	Congenital birth defects	683	2					
4	Neural tube defects	683						
4	Congenital heart anomalies	683						
4	Orofacial clefts	582						
4	Down syndrome	678						
4	Other chromosomal abnormalities	679						
4	Congenital musculoskeletal and limb anomalies	678						
4	Urogenital congenital anomalies	682						
4	Digestive congenital anomalies	683						
4	Other congenital birth defects	683						
3	Skin and subcutaneous diseases	683						
4	Cellulitis	683						
4	Pyoderma	683						
4	Decubitus ulcer	680						
4	Other skin and subcutaneous diseases	680						
3	Sudden infant death syndrome	553						
1	Injuries	683						
2	Transport injuries	683						
3	Road injuries	683						
4	Pedestrian road injuries	683						
4	Cyclist road injuries	683						
4	Motorcyclist road injuries	683						
4	Motor vehicle road injuries	683						
4	Other road injuries	683						
3	Other transport injuries	683						
2	Unintentional injuries	683						
3	Falls	683						
3	Drowning	683						
3	Fire, heat, and hot substances	683						
3	Poisonings	683						
3	Exposure to mechanical forces	683						
4	Unintentional firearm injuries	683						
4	Unintentional suffocation	683						
4	Other exposure to mechanical forces	683						
3	Adverse effects of medical treatment	683						
3	Animal contact	683						
4	Venomous animal contact	683						
4	Non-venomous animal contact	683						
3	Foreign body	683						
4	Pulmonary aspiration and foreign body in airway	683						
4	Foreign body in other body part	682						
3	Environmental heat and cold exposure	683						
3	Other unintentional injuries	683						
2	Self-harm and interpersonal violence	683						

3	Self-harm	683							
4	Self-harm by firearm	683							
4	Self-harm by other specified means	683							
3	Interpersonal violence	683							
4	Physical violence by firearm	683							
4	Physical violence by sharp object	683							
4	Physical violence by other means	683							
2	Forces of nature, conflict and terrorism, and executions and police conflict	683							
3	Exposure to forces of nature	681							
3	Conflict and terrorism	683							
3	Executions and police conflict	668							

Appendix Table 2: Data used in Brazil non-fatal estimates, GBD 2016				
Cause	Citation	Coverage	Years	Data type
HIV/AIDS and tuberculosis	World Health Organization (WHO). WHO Tuberculosis Case Notifications. Geneva, Switzerland: World Health Organization (WHO).	Global	2003-2014	Epi surveillance
Tuberculosis	Institute for Health Metrics and Evaluation (IHME). IHME GBD 2015 DisMod HIV-TB Corrected Cause-specific Mortality Rate Estimates.	Global	1990-2016	Modeled data
Tuberculosis	Institute for Health Metrics and Evaluation (IHME). IHME GBD 2015 DisMod All Causes Excess Mortality Estimates.	Global	2000-2015	Modeled data
Tuberculosis	Camacho LA, Klein CH. [Risk of tubercular infection among schoolchildren with high BCG coverage]. . 1990; 108(2): 100[illegible]2.		1986	Scientific literature
Tuberculosis	Ministry of Health (Brazil). Brazil Information System for Notifiable Diseases 2010.	Country	2010	Epi surveillance
Tuberculosis	Ministry of Health (Brazil). Brazil Information System for Notifiable Diseases 2013.	Country	2013	Epi surveillance
Tuberculosis	Ministry of Health (Brazil). Brazil Information System for Notifiable Diseases 2006.	Country	2006	Epi surveillance
Tuberculosis	World Health Organization (WHO). WHO Tuberculosis Case Notifications. Geneva, Switzerland: World Health Organization (WHO).	Global	2000-2015	Epi surveillance
Tuberculosis	LC. A comparison of dual skin test with mycobacterial antigens and tuberculin skin test alone in estimating prevalence of Mycobacterium tuberculosis infection from population surveys. . 2003; 7(4): 312[illegible]19.		1998	Scientific literature
Multidrug-resistant tuberculosis without extensive drug resistance	World Health Organization (WHO). WHO Global Project on Anti-Tuberculosis Drug Resistance Surveillance Data 1988-2015.	Global	2008	Survey
HIV/AIDS	Ministry of Health (Brazil). Brazil Mortality Information System - Deaths 1988. Rio de Janeiro, Brazil: Ministry of Health (Brazil).	Country	1988	Vital registration
HIV/AIDS	Ministry of Health (Brazil). Brazil Mortality Information System - Deaths 2005. Rio de Janeiro, Brazil: Ministry of Health (Brazil).	Country	2005	Vital registration
HIV/AIDS	Ministry of Health (Brazil). Brazil Mortality Information System - Deaths 1989. Rio de Janeiro, Brazil: Ministry of Health (Brazil).	Country	1989	Vital registration
HIV/AIDS	Ministry of Health (Brazil). Brazil Mortality Information System - Deaths 2004. Rio de Janeiro, Brazil: Ministry of Health (Brazil).	Country	2004	Vital registration
HIV/AIDS	Ministry of Health (Brazil). Brazil Mortality Information System - Deaths 1990. Rio de Janeiro, Brazil: Ministry of Health (Brazil).	Country	1990	Vital registration
HIV/AIDS	Ministry of Health (Brazil). Brazil Mortality Information System - Deaths 2003. Rio de Janeiro, Brazil: Ministry of Health (Brazil).	Country	2003	Vital registration
HIV/AIDS	Ministry of Health (Brazil). Brazil Mortality Information System - Deaths 1991. Rio de Janeiro, Brazil: Ministry of Health (Brazil).	Country	1991	Vital registration
HIV/AIDS	Ministry of Health (Brazil). Brazil Mortality Information System - Deaths 2006. Rio de Janeiro, Brazil: Ministry of Health (Brazil).	Country	2006	Vital registration
HIV/AIDS	Ministry of Health (Brazil). Brazil Mortality Information System - Deaths 1992. Rio de Janeiro, Brazil: Ministry of Health (Brazil).	Country	1992	Vital registration
HIV/AIDS	Ministry of Health (Brazil). Brazil Mortality Information System - Deaths 1987. Rio de Janeiro, Brazil: Ministry of Health (Brazil).	Country	1987	Vital registration
HIV/AIDS	Ministry of Health (Brazil). Brazil Mortality Information System - Deaths 2001. Rio de Janeiro, Brazil: Ministry of Health (Brazil).	Country	2001	Vital registration
HIV/AIDS	Ministry of Health (Brazil). Brazil Mortality Information System - Deaths 1993. Rio de Janeiro, Brazil: Ministry of Health (Brazil).	Country	1993	Vital registration
HIV/AIDS	Ministry of Health (Brazil). Brazil Mortality Information System - Deaths 2000. Rio de Janeiro, Brazil: Ministry of Health (Brazil).	Country	2000	Vital registration
HIV/AIDS	Ministry of Health (Brazil). Brazil Mortality Information System - Deaths 1994. Rio de Janeiro, Brazil: Ministry of Health (Brazil).	Country	1994	Vital registration
HIV/AIDS	Ministry of Health (Brazil). Brazil Mortality Information System - Deaths 1999. Rio de Janeiro, Brazil: Ministry of Health (Brazil).	Country	1999	Vital registration
HIV/AIDS	Ministry of Health (Brazil). Brazil Mortality Information System - Deaths 1995. Rio de Janeiro, Brazil: Ministry of Health (Brazil).	Country	1995	Vital registration
HIV/AIDS	Ministry of Health (Brazil). Brazil Mortality Information System - Deaths 1998. Rio de Janeiro, Brazil: Ministry of Health (Brazil).	Country	1998	Vital registration
HIV/AIDS	Ministry of Health (Brazil). Brazil Mortality Information System - Deaths 2002. Rio de Janeiro, Brazil: Ministry of Health (Brazil).	Country	2002	Vital registration
HIV/AIDS	Ministry of Health (Brazil). Brazil Mortality Information System - Deaths 2007. Rio de Janeiro, Brazil: Ministry of Health (Brazil).	Country	2007	Vital registration
HIV/AIDS	Ministry of Health (Brazil). Brazil Mortality Information System - Deaths 1984. Rio de Janeiro, Brazil: Ministry of Health (Brazil).	Country	1984	Vital registration
HIV/AIDS	Ministry of Health (Brazil). Brazil Mortality Information System - Deaths 2008. Rio de Janeiro, Brazil: Ministry of Health (Brazil).	Country	2008	Vital registration
HIV/AIDS	Ministry of Health (Brazil). Brazil Mortality Information System - Deaths 2015.	Country	2015	Vital registration
HIV/AIDS	Carriquiry G, Fink V, Koethe JR, Giganti MJ, Jayathilake K, Blevins M, Cahn P, Grinsztejn B, Wolff M, Pape JW, Padgett D, Madero JS, Gotuzzo E, McGowan CC, Shepherd BE. Mortality and loss to follow-up among HIV-infected persons on long-term antiretroviral therapy in Latin America and the Caribbean. . 2015; 18: 20016.		2000-2014	Scientific literature
HIV/AIDS	Ministry of Health (Brazil). Brazil Mortality Information System - Deaths 2014.	Country	2014	Vital registration
HIV/AIDS	Tuboi SH, Schechter M, McGowan CC, Cesar C, Krolewiecki A, Cahn P, Wolff M, Pape JW, Padgett D, Madero JS, Gotuzzo E, Masys DR, Shepherd BE. Mortality during the first year of potent antiretroviral therapy in HIV-1-infected patients in 7 sites throughout Latin America and the Caribbean. . 2009; 51(5): 615-23.		1996-2007	Scientific literature
HIV/AIDS	Hunter R, Vasquez-Mora G, Quava-Jones A, Adomakoh N, Peter Figueroa J, Liautaud B, Torres M, Pape JW. Long-term antiretroviral treatment outcomes in seven countries in the Caribbean. . 2012; 59(4): e60-71.		1998-2008	Scientific literature
HIV/AIDS	Keiser O, May M, Sprinz E, Egger M, Anglaret X, ART-LINC, IeDEA. Early loss of HIV-infected patients on potent antiretroviral therapy programmes in lower-income countries. . 2008; 86(7): 559-67.		2000-2004	Scientific literature
HIV/AIDS	Cazanave C, Veloso VG, Dabis F, Grinsztejn B, Chêne G, IPEC/FIOCRUZ Cohort and the Aquitaine ANRS C03 Study Group. AIDS and non-AIDS severe morbidity associated with hospitalizations among HIV-infected patients in two regions with universal access to care and antiretroviral therapy, France and Brazil, 2000-2008: hospital-based cohort studies. . 2014; 278.		2000-2008	Scientific literature
HIV/AIDS	Ministry of Health (Brazil). Brazil Mortality Information System - Deaths 2013. Rio de Janeiro, Brazil: Ministry of Health (Brazil).	Country	2013	Vital registration
HIV/AIDS	Ministry of Health (Brazil). Brazil Mortality Information System - Deaths 1981. Rio de Janeiro, Brazil: Ministry of Health (Brazil).	Country	1981	Vital registration
HIV/AIDS	Ministry of Health (Brazil). Brazil Mortality Information System - Deaths 2012. Rio de Janeiro, Brazil: Ministry of Health (Brazil).	Country	2012	Vital registration
HIV/AIDS	Ministry of Health (Brazil). Brazil Mortality Information System - Deaths 1982. Rio de Janeiro, Brazil: Ministry of Health (Brazil).	Country	1982	Vital registration
HIV/AIDS	Ministry of Health (Brazil). Brazil Mortality Information System - Deaths 2011. Rio de Janeiro, Brazil: Ministry of Health (Brazil).	Country	2011	Vital registration
HIV/AIDS	Ministry of Health (Brazil). Brazil Mortality Information System - Deaths 1983. Rio de Janeiro, Brazil: Ministry of Health (Brazil).	Country	1983	Vital registration
HIV/AIDS	Ministry of Health (Brazil). Brazil Mortality Information System - Deaths 2010. Rio de Janeiro, Brazil: Ministry of Health (Brazil).	Country	2010	Vital registration
HIV/AIDS	Ministry of Health (Brazil). Brazil Mortality Information System - Deaths 1996. Rio de Janeiro, Brazil: Ministry of Health (Brazil).	Country	1996	Vital registration
HIV/AIDS	Ministry of Health (Brazil). Brazil Mortality Information System - Deaths 2009. Rio de Janeiro, Brazil: Ministry of Health (Brazil).	Country	2009	Vital registration
HIV/AIDS	Ministry of Health (Brazil). Brazil Mortality Information System - Deaths 1985. Rio de Janeiro, Brazil: Ministry of Health (Brazil).	Country	1985	Vital registration
HIV/AIDS	Ministry of Health (Brazil). Brazil Mortality Information System - Deaths 1986. Rio de Janeiro, Brazil: Ministry of Health (Brazil).	Country	1986	Vital registration
HIV/AIDS	Ministry of Health (Brazil). Brazil Mortality Information System - Deaths 1997. Rio de Janeiro, Brazil: Ministry of Health (Brazil).	Country	1997	Vital registration
Multidrug-resistant HIV/AIDS - Tuberculosis without extensive drug resistance	World Health Organization (WHO). WHO Global Project on Anti-Tuberculosis Drug Resistance Surveillance Data 1988-2015.	Global	2008	Survey
HIV/AIDS resulting in other diseases	Ministry of Health (Brazil). Brazil Mortality Information System - Deaths 1997. Rio de Janeiro, Brazil: Ministry of Health (Brazil).	Country	1997	Vital registration
HIV/AIDS resulting in other diseases	Ministry of Health (Brazil). Brazil Mortality Information System - Deaths 2008. Rio de Janeiro, Brazil: Ministry of Health (Brazil).	Country	2008	Vital registration
HIV/AIDS resulting in other diseases	Ministry of Health (Brazil). Brazil Mortality Information System - Deaths 1984. Rio de Janeiro, Brazil: Ministry of Health (Brazil).	Country	1984	Vital registration
HIV/AIDS resulting in other diseases	Ministry of Health (Brazil). Brazil Mortality Information System - Deaths 2009. Rio de Janeiro, Brazil: Ministry of Health (Brazil).	Country	2009	Vital registration

HIV/AIDS resulting in other diseases	Ministry of Health (Brazil). Brazil Mortality Information System - Deaths 1983. Rio de Janeiro, Brazil: Ministry of Health (Brazil).	Country	1983	Vital registration
HIV/AIDS resulting in other diseases	Ministry of Health (Brazil). Brazil Mortality Information System - Deaths 1982. Rio de Janeiro, Brazil: Ministry of Health (Brazil).	Country	1982	Vital registration
HIV/AIDS resulting in other diseases	Ministry of Health (Brazil). Brazil Mortality Information System - Deaths 2011. Rio de Janeiro, Brazil: Ministry of Health (Brazil).	Country	2011	Vital registration
HIV/AIDS resulting in other diseases	Ministry of Health (Brazil). Brazil Mortality Information System - Deaths 1981. Rio de Janeiro, Brazil: Ministry of Health (Brazil).	Country	1981	Vital registration
HIV/AIDS resulting in other diseases	Ministry of Health (Brazil). Brazil Mortality Information System - Deaths 2012. Rio de Janeiro, Brazil: Ministry of Health (Brazil).	Country	2012	Vital registration
HIV/AIDS resulting in other diseases	Ministry of Health (Brazil). Brazil Mortality Information System - Deaths 1996. Rio de Janeiro, Brazil: Ministry of Health (Brazil).	Country	1996	Vital registration
HIV/AIDS resulting in other diseases	Ministry of Health (Brazil). Brazil Mortality Information System - Deaths 2013. Rio de Janeiro, Brazil: Ministry of Health (Brazil).	Country	2013	Vital registration
HIV/AIDS resulting in other diseases	Cazanave C, Veloso VG, Dabis F, Grinsztejn B, Chêne G, IPEC/FIOCRUZ Cohort and the Aquitaine ANRS C03 Study Group. AIDS and non-AIDS severe morbidity associated with hospitalizations among HIV-infected patients in two regions with universal access to care and antiretroviral therapy, France and Brazil, 2000-2008: hospital-based cohort studies. . 2014; 278.		2000-2008	Scientific literature
HIV/AIDS resulting in other diseases	Keiser O, May M, Sprinz E, Egger M, Anglaret X, ART-LINC, IeDEA. Early loss of HIV-infected patients on potent antiretroviral therapy programmes in lower-income countries. . 2008; 86(7): 559-67.		2000-2004	Scientific literature
HIV/AIDS resulting in other diseases	Hunter R, Vasquez-Mora G, Quava-Jones A, Adomakoh N, Peter Figueroa J, Liautaud B, Torres M, Pape JW. Long-term antiretroviral treatment outcomes in seven countries in the Caribbean. . 2012; 59(4): e60-71.		1998-2008	Scientific literature
HIV/AIDS resulting in other diseases	Tuboi SH, Schechter M, McGowan CC, Cesar C, Krolewiecki A, Cahn P, Wolff M, Pape JW, Padgett D, Madero JS, Gotuzzo E, Masys DR, Shepherd BE. Mortality during the first year of potent antiretroviral therapy in HIV-1-infected patients in 7 sites throughout Latin America and the Caribbean. . 2009; 51(5): 615-23.		1996-2007	Scientific literature
HIV/AIDS resulting in other diseases	Ministry of Health (Brazil). Brazil Mortality Information System - Deaths 2014.	Country	2014	Vital registration
HIV/AIDS resulting in other diseases	Carriquiry G, Fink V, Koethe JR, Giganti MJ, Jayathilake K, Blevins M, Cahn P, Grinsztejn B, Wolff M, Pape JW, Padgett D, Madero JS, Gotuzzo E, McGowan CC, Shepherd BE. Mortality and loss to follow-up among HIV-infected persons on long-term antiretroviral therapy in Latin America and the Caribbean. . 2015; 18: 20016.		2000-2014	Scientific literature
HIV/AIDS resulting in other diseases	Ministry of Health (Brazil). Brazil Mortality Information System - Deaths 2015.	Country	2015	Vital registration
HIV/AIDS resulting in other diseases	Ministry of Health (Brazil). Brazil Mortality Information System - Deaths 1985. Rio de Janeiro, Brazil: Ministry of Health (Brazil).	Country	1985	Vital registration
HIV/AIDS resulting in other diseases	Ministry of Health (Brazil). Brazil Mortality Information System - Deaths 2007. Rio de Janeiro, Brazil: Ministry of Health (Brazil).	Country	2007	Vital registration
HIV/AIDS resulting in other diseases	Ministry of Health (Brazil). Brazil Mortality Information System - Deaths 2010. Rio de Janeiro, Brazil: Ministry of Health (Brazil).	Country	2010	Vital registration
HIV/AIDS resulting in other diseases	Ministry of Health (Brazil). Brazil Mortality Information System - Deaths 2006. Rio de Janeiro, Brazil: Ministry of Health (Brazil).	Country	2006	Vital registration
HIV/AIDS resulting in other diseases	Ministry of Health (Brazil). Brazil Mortality Information System - Deaths 1994. Rio de Janeiro, Brazil: Ministry of Health (Brazil).	Country	1994	Vital registration
HIV/AIDS resulting in other diseases	Ministry of Health (Brazil). Brazil Mortality Information System - Deaths 1999. Rio de Janeiro, Brazil: Ministry of Health (Brazil).	Country	1999	Vital registration
HIV/AIDS resulting in other diseases	Ministry of Health (Brazil). Brazil Mortality Information System - Deaths 1993. Rio de Janeiro, Brazil: Ministry of Health (Brazil).	Country	1993	Vital registration
HIV/AIDS resulting in other diseases	Ministry of Health (Brazil). Brazil Mortality Information System - Deaths 1986. Rio de Janeiro, Brazil: Ministry of Health (Brazil).	Country	1986	Vital registration
HIV/AIDS resulting in other diseases	Ministry of Health (Brazil). Brazil Mortality Information System - Deaths 2000. Rio de Janeiro, Brazil: Ministry of Health (Brazil).	Country	2000	Vital registration
HIV/AIDS resulting in other diseases	Ministry of Health (Brazil). Brazil Mortality Information System - Deaths 1992. Rio de Janeiro, Brazil: Ministry of Health (Brazil).	Country	1992	Vital registration
HIV/AIDS resulting in other diseases	Ministry of Health (Brazil). Brazil Mortality Information System - Deaths 1995. Rio de Janeiro, Brazil: Ministry of Health (Brazil).	Country	1995	Vital registration
HIV/AIDS resulting in other diseases	Ministry of Health (Brazil). Brazil Mortality Information System - Deaths 1991. Rio de Janeiro, Brazil: Ministry of Health (Brazil).	Country	1991	Vital registration
HIV/AIDS resulting in other diseases	Ministry of Health (Brazil). Brazil Mortality Information System - Deaths 2001. Rio de Janeiro, Brazil: Ministry of Health (Brazil).	Country	2001	Vital registration
HIV/AIDS resulting in other diseases	Ministry of Health (Brazil). Brazil Mortality Information System - Deaths 1990. Rio de Janeiro, Brazil: Ministry of Health (Brazil).	Country	1990	Vital registration
HIV/AIDS resulting in other diseases	Ministry of Health (Brazil). Brazil Mortality Information System - Deaths 2003. Rio de Janeiro, Brazil: Ministry of Health (Brazil).	Country	2003	Vital registration
HIV/AIDS resulting in other diseases	Ministry of Health (Brazil). Brazil Mortality Information System - Deaths 1989. Rio de Janeiro, Brazil: Ministry of Health (Brazil).	Country	1989	Vital registration
HIV/AIDS resulting in other diseases	Ministry of Health (Brazil). Brazil Mortality Information System - Deaths 2004. Rio de Janeiro, Brazil: Ministry of Health (Brazil).	Country	2004	Vital registration
HIV/AIDS resulting in other diseases	Ministry of Health (Brazil). Brazil Mortality Information System - Deaths 1988. Rio de Janeiro, Brazil: Ministry of Health (Brazil).	Country	1988	Vital registration
HIV/AIDS resulting in other diseases	Ministry of Health (Brazil). Brazil Mortality Information System - Deaths 2005. Rio de Janeiro, Brazil: Ministry of Health (Brazil).	Country	2005	Vital registration
HIV/AIDS resulting in other diseases	Ministry of Health (Brazil). Brazil Mortality Information System - Deaths 1987. Rio de Janeiro, Brazil: Ministry of Health (Brazil).	Country	1987	Vital registration
HIV/AIDS resulting in other diseases	Ministry of Health (Brazil). Brazil Mortality Information System - Deaths 2002. Rio de Janeiro, Brazil: Ministry of Health (Brazil).	Country	2002	Vital registration
HIV/AIDS resulting in other diseases	Ministry of Health (Brazil). Brazil Mortality Information System - Deaths 1998. Rio de Janeiro, Brazil: Ministry of Health (Brazil).	Country	1998	Vital registration
Diarrheal diseases	Volotão EM, Soares CC, Maranhão AG, Rocha LN, Hoshino Y, Santos N. Rotavirus surveillance in the city of Rio de Janeiro-Brazil during 2000-2004: detection of unusual strains with G8P4 or G10P9 specificities. . 2006; 78(2): 263-72.		2000-2004	Scientific literature
Diarrheal diseases	Volotão EM, Soares CC, Maranhão AG, Rocha LN, Hoshino Y, Santos N. Rotavirus surveillance in the city of Rio de Janeiro-Brazil during 2000-2004: detection of unusual strains with G8P4 or G10P9 specificities. . 2006; 78(2): 263-72.		2000-2004	Scientific literature
Diarrheal diseases	Sobel J, Gomes TAT, Ramos RTS, Hoekstra M, Rodrigue D, Rassi V, Griffin PM. Pathogen-specific risk factors and protective factors for acute diarrheal illness in children aged 12-59 months in São Paulo, Brazil. . 2004; 38(11): 1545-51.		1989-1990	Scientific literature
Diarrheal diseases	Soares CC, Volotão EM, Albuquerque MCM, da Silva FM, de Carvalho TRB, Nozawa CM, Linhares RE, Santos N. Prevalence of enteric adenoviruses among children with diarrhea in four Brazilian cities. . 2002; 23(3): 171-7.		1998-2000	Scientific literature
Diarrheal diseases	Soares CC, Volotão EM, Albuquerque MCM, da Silva FM, de Carvalho TRB, Nozawa CM, Linhares RE, Santos N. Prevalence of enteric adenoviruses among children with diarrhea in four Brazilian cities. . 2002; 23(3): 171-7.		1998-2000	Scientific literature
Diarrheal diseases	Rosa E Silva ML, Pires De Carvalho I, Gouvea V. 1998-1999 rotavirus seasons in Juiz de Fora, Minas Gerais, Brazil: detection of an unusual G3P4 epidemic strain. . 2002; 40(8): 2837-42.		1998-1999	Scientific literature
Diarrheal diseases	Santos N, Volotão EM, Soares CC, Campos GS, Sardi SI, Hoshino Y. Predominance of rotavirus genotype G9 during the 1999, 2000, and 2002 seasons among hospitalized children in the city of Salvador, Bahia, Brazil: implications for future vaccine strategies. . 2005; 43(8): 4064-9.		1999-2002	Scientific literature
Diarrheal diseases	Santos N, Volotão EM, Soares CC, Campos GS, Sardi SI, Hoshino Y. Predominance of rotavirus genotype G9 during the 1999, 2000, and 2002 seasons among hospitalized children in the city of Salvador, Bahia, Brazil: implications for future vaccine strategies. . 2005; 43(8): 4064-9.		1999-2002	Scientific literature
Diarrheal diseases	Rosa E Silva ML, Pires De Carvalho I, Gouvea V. 1998-1999 rotavirus seasons in Juiz de Fora, Minas Gerais, Brazil: detection of an unusual G3P4 epidemic strain. . 2002; 40(8): 2837-42.		1998-1999	Scientific literature
Diarrheal diseases	Cardoso D das D de P, Soares CMA, Dias e Souza MB de L, de Azevedo M da SP, Martins RMB, Queiróz DA de O, de Brito WMED, Munford V, Rácz ML. Epidemiological features of rotavirus infection in Goiânia, Goiás, Brazil, from 1986 to 2000. . 2003; 98(1): 25-9.		1986-2000	Scientific literature
Diarrheal diseases	Cardoso D das D de P, Soares CMA, Dias e Souza MB de L, de Azevedo M da SP, Martins RMB, Queiróz DA de O, de Brito WMED, Munford V, Rácz ML. Epidemiological features of rotavirus infection in Goiânia, Goiás, Brazil, from 1986 to 2000. . 2003; 98(1): 25-9.		1986-2000	Scientific literature
Diarrheal diseases	Sobel J, Gomes TAT, Ramos RTS, Hoekstra M, Rodrigue D, Rassi V, Griffin PM. Pathogen-specific risk factors and protective factors for acute diarrheal illness in children aged 12-59 months in São Paulo, Brazil. . 2004; 38(11): 1545-51.		1989-1990	Scientific literature
Diarrheal diseases	Soares CC, Volotão EM, Albuquerque MCM, da Silva FM, de Carvalho TRB, Nozawa CM, Linhares RE, Santos N. Prevalence of enteric adenoviruses among children with diarrhea in four Brazilian cities. . 2002; 23(3): 171-7.		1998-2000	Scientific literature

Diarrheal diseases	Sobel J, Gomes TAT, Ramos RTS, Hoekstra M, Rodrigue D, Rassi V, Griffin PM. Pathogen-specific risk factors and protective factors for acute diarrheal illness in children aged 12-59 months in São Paulo, Brazil. . 2004; 38(11): 1545-51.		1989-1990	Scientific literature
Diarrheal diseases	Guerrant RL, Kirchhoff LV, Shields DS, Nations MK, Leslie J, de Sousa MA, Araujo JG, Correia LL, Sauer KT, McClelland KE. Prospective study of diarrheal illnesses in northeastern Brazil: patterns of disease, nutritional impact, etiologies, and risk factors. . 1983; 148(6): 986-97.		1978-1980	Scientific literature
Diarrheal diseases	Sobel J, Gomes TAT, Ramos RTS, Hoekstra M, Rodrigue D, Rassi V, Griffin PM. Pathogen-specific risk factors and protective factors for acute diarrheal illness in children aged 12-59 months in São Paulo, Brazil. . 2004; 38(11): 1545-51.		1989-1990	Scientific literature
Diarrheal diseases	Sobel J, Gomes TAT, Ramos RTS, Hoekstra M, Rodrigue D, Rassi V, Griffin PM. Pathogen-specific risk factors and protective factors for acute diarrheal illness in children aged 12-59 months in São Paulo, Brazil. . 2004; 38(11): 1545-51.		1989-1990	Scientific literature
Diarrheal diseases	Sobel J, Gomes TAT, Ramos RTS, Hoekstra M, Rodrigue D, Rassi V, Griffin PM. Pathogen-specific risk factors and protective factors for acute diarrheal illness in children aged 12-59 months in São Paulo, Brazil. . 2004; 38(11): 1545-51.		1989-1990	Scientific literature
Diarrheal diseases	Sobel J, Gomes TAT, Ramos RTS, Hoekstra M, Rodrigue D, Rassi V, Griffin PM. Pathogen-specific risk factors and protective factors for acute diarrheal illness in children aged 12-59 months in São Paulo, Brazil. . 2004; 38(11): 1545-51.		1989-1990	Scientific literature
Diarrheal diseases	Munford V, Gilio AE, de Souza EC, Cardoso DM, Cardoso D das D de P, Borges AMT, Costa PSS da, Melgaço IAM, Rosa H, Carvalho PRA, Goldani MZ, Moreira ED Jr, Santana C, El Khoury A, Ikedo F, Rácz ML. Rotavirus gastroenteritis in children in 4 regions in Brazil: a hospital-based surveillance study. . 2009; 200(Supp 1): S106-113.		2005-2006	Scientific literature
Diarrheal diseases	Munford V, Gilio AE, de Souza EC, Cardoso DM, Cardoso D das D de P, Borges AMT, Costa PSS da, Melgaço IAM, Rosa H, Carvalho PRA, Goldani MZ, Moreira ED Jr, Santana C, El Khoury A, Ikedo F, Rácz ML. Rotavirus gastroenteritis in children in 4 regions in Brazil: a hospital-based surveillance study. . 2009; 200(Supp 1): S106-113.		2005-2006	Scientific literature
Diarrheal diseases	Stewien KE, da Cunha LC, Alvim A de C, dos Reis Filho SA, Alvim MA, Brandão AA, Neiva MN. Rotavirus associated diarrhoea during infancy in the city of S. Luís (MA), Brazil: a two-year longitudinal study. . 1991; 33(6): 459-64.		1986-1988	Scientific literature
Diarrheal diseases	Stewien KE, da Cunha LC, Alvim A de C, dos Reis Filho SA, Alvim MA, Brandão AA, Neiva MN. Rotavirus associated diarrhoea during infancy in the city of S. Luís (MA), Brazil: a two-year longitudinal study. . 1991; 33(6): 459-64.		1986-1988	Scientific literature
Diarrheal diseases	CHOICE Study Group. Multicenter, randomized, double-blind clinical trial to evaluate the efficacy and safety of a reduced osmolarity oral rehydration salts solution in children with acute watery diarrhea. . 2001; 107(4): 613-8.		1995-1997	Scientific literature
Diarrheal diseases	CHOICE Study Group. Multicenter, randomized, double-blind clinical trial to evaluate the efficacy and safety of a reduced osmolarity oral rehydration salts solution in children with acute watery diarrhea. . 2001; 107(4): 613-8.		1995-1997	Scientific literature
Diarrheal diseases	Coiro JR, Bendati MM, de Almeida Neto AJ, Heuser CF, Vasconcellos VL. Rotavirus infection in Brazilian children with acute enteritis: a seasonal variation study. . 1983; 32(5): 1186-8.	Country	1981-1982	Scientific literature
Diarrheal diseases	Coiro JR, Bendati MM, de Almeida Neto AJ, Heuser CF, Vasconcellos VL. Rotavirus infection in Brazilian children with acute enteritis: a seasonal variation study. . 1983; 32(5): 1186-8.	Country	1981-1982	Scientific literature
Diarrheal diseases	Luz CRNE da, Mascarenhas JDP, Gabbay YB, Motta ARB, Lima TVR, Soares L da S, Linhares AC. Rotavirus serotypes and electropherotypes identified among hospitalised children in São Luís, Maranhão, Brazil. . 2005; 47(5): 287-93.		1997-1999	Scientific literature
Diarrheal diseases	Guerrant RL, Kirchhoff LV, Shields DS, Nations MK, Leslie J, de Sousa MA, Araujo JG, Correia LL, Sauer KT, McClelland KE. Prospective study of diarrheal illnesses in northeastern Brazil: patterns of disease, nutritional impact, etiologies, and risk factors. . 1983; 148(6): 986-97.		1978-1980	Scientific literature
Diarrheal diseases	Sobel J, Gomes TAT, Ramos RTS, Hoekstra M, Rodrigue D, Rassi V, Griffin PM. Pathogen-specific risk factors and protective factors for acute diarrheal illness in children aged 12-59 months in São Paulo, Brazil. . 2004; 38(11): 1545-51.		1989-1990	Scientific literature
Diarrheal diseases	Luz CRNE da, Mascarenhas JDP, Gabbay YB, Motta ARB, Lima TVR, Soares L da S, Linhares AC. Rotavirus serotypes and electropherotypes identified among hospitalised children in São Luís, Maranhão, Brazil. . 2005; 47(5): 287-93.		1997-1999	Scientific literature
Diarrheal diseases	Araújo IT, Fialho AM, de Assis RMS, Rocha M, Galvão M, Cruz CM, Ferreira MSR, Leite JPG. Rotavirus strain diversity in Rio de Janeiro, Brazil: characterization of VP4 and VP7 genotypes in hospitalized children. . 2002; 48(4): 214-8.		1996-1999	Scientific literature
Diarrheal diseases	Lima AA, Guerrant RL. Persistent diarrhea in children: epidemiology, risk factors, pathophysiology, nutritional impact, and management. . 1992; 14: 222-42.		1989-1991	Scientific literature
Diarrheal diseases	Fang GD, Lima AA, Martins CV, Nataro JP, Guerrant RL. Etiology and epidemiology of persistent diarrhea in northeastern Brazil: a hospital-based, prospective, case-control study. . 1995; 21(2): 137-44.		1988-1991	Scientific literature
Diarrheal diseases	Fang GD, Lima AA, Martins CV, Nataro JP, Guerrant RL. Etiology and epidemiology of persistent diarrhea in northeastern Brazil: a hospital-based, prospective, case-control study. . 1995; 21(2): 137-44.		1988-1991	Scientific literature
Diarrheal diseases	Fang GD, Lima AA, Martins CV, Nataro JP, Guerrant RL. Etiology and epidemiology of persistent diarrhea in northeastern Brazil: a hospital-based, prospective, case-control study. . 1995; 21(2): 137-44.		1988-1991	Scientific literature
Diarrheal diseases	Fang GD, Lima AA, Martins CV, Nataro JP, Guerrant RL. Etiology and epidemiology of persistent diarrhea in northeastern Brazil: a hospital-based, prospective, case-control study. . 1995; 21(2): 137-44.		1988-1991	Scientific literature
Diarrheal diseases	Gusmão RH, Mascarenhas JD, Gabbay YB, Lins-Lainson Z, Ramos FL, Monteiro TA, Valente SA, Fagundes-Neto U, Linhares AC. Rotavirus subgroups, G serotypes, and electrophoretypes in cases of nosocomial infantile diarrhoea in Belém, Brazil. . 1999; 45(2): 81-6.		1992-1994	Scientific literature
Diarrheal diseases	Gusmão RH, Mascarenhas JD, Gabbay YB, Lins-Lainson Z, Ramos FL, Monteiro TA, Valente SA, Fagundes-Neto U, Linhares AC. Rotavirus subgroups, G serotypes, and electrophoretypes in cases of nosocomial infantile diarrhoea in Belém, Brazil. . 1999; 45(2): 81-6.		1992-1994	Scientific literature
Diarrheal diseases	Gusmão RH, Mascarenhas JD, Gabbay YB, Lins-Lainson Z, Ramos FL, Monteiro TA, Valente SA, Fagundes-Neto U, Linhares AC. Rotavirus subgroups, G serotypes, and electrophoretypes in cases of nosocomial infantile diarrhoea in Belém, Brazil. . 1999; 45(2): 81-6.		1992-1994	Scientific literature
Diarrheal diseases	Gusmão RH, Mascarenhas JD, Gabbay YB, Lins-Lainson Z, Ramos FL, Monteiro TA, Valente SA, Fagundes-Neto U, Linhares AC. Rotavirus subgroups, G serotypes, and electrophoretypes in cases of nosocomial infantile diarrhoea in Belém, Brazil. . 1999; 45(2): 81-6.		1992-1994	Scientific literature
Diarrheal diseases	Gusmão RH, Mascarenhas JD, Gabbay YB, Lins-Lainson Z, Ramos FL, Monteiro TA, Valente SA, Fagundes-Neto U, Linhares AC. Rotavirus subgroups, G serotypes, and electrophoretypes in cases of nosocomial infantile diarrhoea in Belém, Brazil. . 1999; 45(2): 81-6.		1992-1994	Scientific literature
Diarrheal diseases	Gusmão RH, Mascarenhas JD, Gabbay YB, Lins-Lainson Z, Ramos FL, Monteiro TA, Valente SA, Fagundes-Neto U, Linhares AC. Rotavirus subgroups, G serotypes, and electrophoretypes in cases of nosocomial infantile diarrhoea in Belém, Brazil. . 1999; 45(2): 81-6.		1992-1994	Scientific literature
Diarrheal diseases	Gusmão RH, Mascarenhas JD, Gabbay YB, Lins-Lainson Z, Ramos FL, Monteiro TA, Valente SA, Fagundes-Neto U, Linhares AC. Rotavirus subgroups, G serotypes, and electrophoretypes in cases of nosocomial infantile diarrhoea in Belém, Brazil. . 1999; 45(2): 81-6.		1992-1994	Scientific literature
Diarrheal diseases	Araújo IT, Fialho AM, de Assis RMS, Rocha M, Galvão M, Cruz CM, Ferreira MSR, Leite JPG. Rotavirus strain diversity in Rio de Janeiro, Brazil: characterization of VP4 and VP7 genotypes in hospitalized children. . 2002; 48(4): 214-8.		1996-1999	Scientific literature
Diarrheal diseases	Guerrant RL, Kirchhoff LV, Shields DS, Nations MK, Leslie J, de Sousa MA, Araujo JG, Correia LL, Sauer KT, McClelland KE. Prospective study of diarrheal illnesses in northeastern Brazil: patterns of disease, nutritional impact, etiologies, and risk factors. . 1983; 148(6): 986-97.		1978-1980	Scientific literature
Diarrheal diseases	Bittencourt JA, Arbo E, Malysz AS, Oravec R, Dias C. Seasonal and age distribution of rotavirus infection in Porto Alegre--Brazil. . 2000; 4(6): 279-83.		1996-1998	Scientific literature
Diarrheal diseases	Lima AA, Guerrant RL. Persistent diarrhea in children: epidemiology, risk factors, pathophysiology, nutritional impact, and management. . 1992; 14: 222-42.		1989-1991	Scientific literature
Diarrheal diseases	Bittencourt JA, Arbo E, Malysz AS, Oravec R, Dias C. Seasonal and age distribution of rotavirus infection in Porto Alegre--Brazil. . 2000; 4(6): 279-83.		1996-1998	Scientific literature
Diarrheal diseases	Carmona RCC, Timenetsky M do CST, da Silva FF, Granato CFH. Characterization of rotavirus strains from hospitalized and outpatient children with acute diarrhoea in São Paulo, Brazil. . 2004; 74(1): 166-72.		1994-1995	Scientific literature
Diarrheal diseases	Carmona RCC, Timenetsky M do CST, da Silva FF, Granato CFH. Characterization of rotavirus strains from hospitalized and outpatient children with acute diarrhoea in São Paulo, Brazil. . 2004; 74(1): 166-72.		1994-1995	Scientific literature
Diarrheal diseases	Carmona RCC, Timenetsky M do CST, da Silva FF, Granato CFH. Characterization of rotavirus strains from hospitalized and outpatient children with acute diarrhoea in São Paulo, Brazil. . 2004; 74(1): 166-72.		1994-1995	Scientific literature
Diarrheal diseases	Carmona RCC, Timenetsky M do CST, da Silva FF, Granato CFH. Characterization of rotavirus strains from hospitalized and outpatient children with acute diarrhoea in São Paulo, Brazil. . 2004; 74(1): 166-72.		1994-1995	Scientific literature

Diarrheal diseases	Carmona RCC, Timenetsky M do CST, da Silva FF, Granato CFH. Characterization of rotavirus strains from hospitalized and outpatient children with acute diarrhoea in São Paulo, Brazil. . 2004; 74(1): 166-72.		1994-1995	Scientific literature
Diarrheal diseases	Carmona RCC, Timenetsky M do CST, da Silva FF, Granato CFH. Characterization of rotavirus strains from hospitalized and outpatient children with acute diarrhoea in São Paulo, Brazil. . 2004; 74(1): 166-72.		1994-1995	Scientific literature
Diarrheal diseases	Carmona RCC, Timenetsky M do CST, da Silva FF, Granato CFH. Characterization of rotavirus strains from hospitalized and outpatient children with acute diarrhoea in São Paulo, Brazil. . 2004; 74(1): 166-72.		1994-1995	Scientific literature
Diarrheal diseases	Carmona RCC, Timenetsky M do CST, da Silva FF, Granato CFH. Characterization of rotavirus strains from hospitalized and outpatient children with acute diarrhoea in São Paulo, Brazil. . 2004; 74(1): 166-72.		1994-1995	Scientific literature
Diarrheal diseases	Carmona RCC, Timenetsky M do CST, da Silva FF, Granato CFH. Characterization of rotavirus strains from hospitalized and outpatient children with acute diarrhoea in São Paulo, Brazil. . 2004; 74(1): 166-72.		1994-1995	Scientific literature
Diarrheal diseases	Da Silva Domingues AL, da Silva Vaz MG, Moreno M, Câmara FP. Molecular epidemiology of group A rotavirus causing acute diarrhea in infants and young children hospitalized in Rio de Janeiro, Brazil, 1995-1996. . 2000; 4(3): 119-25.		1995-1996	Scientific literature
Diarrheal diseases	Da Silva Domingues AL, da Silva Vaz MG, Moreno M, Câmara FP. Molecular epidemiology of group A rotavirus causing acute diarrhea in infants and young children hospitalized in Rio de Janeiro, Brazil, 1995-1996. . 2000; 4(3): 119-25.		1995-1996	Scientific literature
Diarrheal diseases	Fernandes JV, Fonseca SM, Azevedo JC, Maranhão H de S, Fonseca MH, Dantas MT, Meissner R de V. Rotavirus detection in feces of children with acute diarrhea. . 2000; 76(4): 300-4.		1996-1998	Scientific literature
Diarrheal diseases	Fernandes JV, Fonseca SM, Azevedo JC, Maranhão H de S, Fonseca MH, Dantas MT, Meissner R de V. Rotavirus detection in feces of children with acute diarrhea. . 2000; 76(4): 300-4.		1996-1998	Scientific literature
Diarrheal diseases	Lima AA, Guerrant RL. Persistent diarrhea in children: epidemiology, risk factors, pathophysiology, nutritional impact, and management. . 1992; 14: 222-42.		1989-1991	Scientific literature
Diarrheal diseases	Carmona RCC, Timenetsky M do CST, da Silva FF, Granato CFH. Characterization of rotavirus strains from hospitalized and outpatient children with acute diarrhoea in São Paulo, Brazil. . 2004; 74(1): 166-72.		1994-1995	Scientific literature
Diarrheal diseases	Guerrant RL, Kirchhoff LV, Shields DS, Nations MK, Leslie J, de Sousa MA, Araujo JG, Correia LL, Sauer KT, McClelland KE. Prospective study of diarrheal illnesses in northeastern Brazil: patterns of disease, nutritional impact, etiologies, and risk factors. . 1983; 148(6): 986-97.		1978-1980	Scientific literature
Diarrheal diseases	Sartori AMC, Valentim J, de Soárez PC, Novaes HMD. Rotavirus morbidity and mortality in children in Brazil. . 2008; 23(2): 92-100.		1986-2006	Scientific literature
Diarrheal diseases	Guerrant RL, Kirchhoff LV, Shields DS, Nations MK, Leslie J, de Sousa MA, Araujo JG, Correia LL, Sauer KT, McClelland KE. Prospective study of diarrheal illnesses in northeastern Brazil: patterns of disease, nutritional impact, etiologies, and risk factors. . 1983; 148(6): 986-97.		1978-1980	Scientific literature
Diarrheal diseases	Lozer DM, Souza TB, Monfardini MV, Vicentini F, Kitagawa SS, Scaletsky IC, Spano LC. Genotypic and phenotypic analysis of diarrheagenic Escherichia coli strains isolated from Brazilian children living in low socioeconomic level communities. . 2013; 418.		2007-2008	Scientific literature
Diarrheal diseases	Lozer DM, Souza TB, Monfardini MV, Vicentini F, Kitagawa SS, Scaletsky IC, Spano LC. Genotypic and phenotypic analysis of diarrheagenic Escherichia coli strains isolated from Brazilian children living in low socioeconomic level communities. . 2013; 418.		2007-2008	Scientific literature
Diarrheal diseases	Lozer DM, Souza TB, Monfardini MV, Vicentini F, Kitagawa SS, Scaletsky IC, Spano LC. Genotypic and phenotypic analysis of diarrheagenic Escherichia coli strains isolated from Brazilian children living in low socioeconomic level communities. . 2013; 418.		2007-2008	Scientific literature
Diarrheal diseases	Lozer DM, Souza TB, Monfardini MV, Vicentini F, Kitagawa SS, Scaletsky IC, Spano LC. Genotypic and phenotypic analysis of diarrheagenic Escherichia coli strains isolated from Brazilian children living in low socioeconomic level communities. . 2013; 418.		2007-2008	Scientific literature
Diarrheal diseases	Lozer DM, Souza TB, Monfardini MV, Vicentini F, Kitagawa SS, Scaletsky IC, Spano LC. Genotypic and phenotypic analysis of diarrheagenic Escherichia coli strains isolated from Brazilian children living in low socioeconomic level communities. . 2013; 418.		2007-2008	Scientific literature
Diarrheal diseases	Ministry of Health (Brazil). Brazil Hospital Information System 1998-2002.		1998-2002	Administrative record
Diarrheal diseases	Ministry of Health (Brazil). Brazil Hospital Information System 2003-2007.		2003-2007	Administrative record
Diarrheal diseases	Ministry of Health (Brazil). Brazil Hospital Information System 2008-2012.		2008-2012	Administrative record
Diarrheal diseases	Ministry of Health (Brazil). Brazil Hospital Information System 2013-2014.		2013-2017	Administrative record
Diarrheal diseases	Boccolini CS, Boccolini P de MM, de Carvalho ML, de Oliveira MIC. [Exclusive breastfeeding and diarrhea hospitalization patterns between 1999 and 2008 in Brazilian State Capitals]. . 2012; 17(7): 1857-63.		1999-2008	Scientific literature
Diarrheal diseases	Luchs A, Cilli A, Morillo SG, de Cassia Compagnoli Carmona R, do Carmo Sampaio Tavares Timenetsky M. Rotavirus in adults, Brazil, 2004-2011: G2P[4] dominance and potential impact on vaccination. . 2014; 18(1): 539.		2004-2011	Scientific literature
Diarrheal diseases	Luchs A, Cilli A, Morillo SG, de Cassia Compagnoli Carmona R, do Carmo Sampaio Tavares Timenetsky M. Rotavirus in adults, Brazil, 2004-2011: G2P[4] dominance and potential impact on vaccination. . 2014; 18(1): 539.		2004-2011	Scientific literature
Diarrheal diseases	Fogarty International Center, National Institutes of Health (NIH), Foundation for the National Institutes of Health (FNIH), National Institute of Science, Technology, and Biomedicine of Semi-Arid Brazil (INCT-IBISAB). Brazil - Fortaleza Malnutrition and Enteric Disease Study 2009-2014.	Ceará	2009-2014	Survey
Diarrheal diseases	Fogarty International Center, National Institutes of Health (NIH), Foundation for the National Institutes of Health (FNIH), National Institute of Science, Technology, and Biomedicine of Semi-Arid Brazil (INCT-IBISAB). Brazil - Fortaleza Malnutrition and Enteric Disease Study 2009-2014.	Ceará	2009-2014	Survey
Diarrheal diseases	Lozer DM, Souza TB, Monfardini MV, Vicentini F, Kitagawa SS, Scaletsky IC, Spano LC. Genotypic and phenotypic analysis of diarrheagenic Escherichia coli strains isolated from Brazilian children living in low socioeconomic level communities. . 2013; 418.		2007-2008	Scientific literature
Diarrheal diseases	Fogarty International Center, National Institutes of Health (NIH), Foundation for the National Institutes of Health (FNIH), National Institute of Science, Technology, and Biomedicine of Semi-Arid Brazil (INCT-IBISAB). Brazil - Fortaleza Malnutrition and Enteric Disease Study 2009-2014.	Ceará	2009-2014	Survey
Diarrheal diseases	Fogarty International Center, National Institutes of Health (NIH), Foundation for the National Institutes of Health (FNIH), National Institute of Science, Technology, and Biomedicine of Semi-Arid Brazil (INCT-IBISAB). Brazil - Fortaleza Malnutrition and Enteric Disease Study 2009-2014.	Ceará	2009-2014	Survey
Diarrheal diseases	Fogarty International Center, National Institutes of Health (NIH), Foundation for the National Institutes of Health (FNIH), National Institute of Science, Technology, and Biomedicine of Semi-Arid Brazil (INCT-IBISAB). Brazil - Fortaleza Malnutrition and Enteric Disease Study 2009-2014.	Ceará	2009-2014	Survey
Diarrheal diseases	Fogarty International Center, National Institutes of Health (NIH), Foundation for the National Institutes of Health (FNIH), National Institute of Science, Technology, and Biomedicine of Semi-Arid Brazil (INCT-IBISAB). Brazil - Fortaleza Malnutrition and Enteric Disease Study 2009-2014.	Ceará	2009-2014	Survey
Diarrheal diseases	Fogarty International Center, National Institutes of Health (NIH), Foundation for the National Institutes of Health (FNIH), National Institute of Science, Technology, and Biomedicine of Semi-Arid Brazil (INCT-IBISAB). Brazil - Fortaleza Malnutrition and Enteric Disease Study 2009-2014.	Ceará	2009-2014	Survey
Diarrheal diseases	Fogarty International Center, National Institutes of Health (NIH), Foundation for the National Institutes of Health (FNIH), National Institute of Science, Technology, and Biomedicine of Semi-Arid Brazil (INCT-IBISAB). Brazil - Fortaleza Malnutrition and Enteric Disease Study 2009-2014.	Ceará	2009-2014	Survey
Diarrheal diseases	Fogarty International Center, National Institutes of Health (NIH), Foundation for the National Institutes of Health (FNIH), National Institute of Science, Technology, and Biomedicine of Semi-Arid Brazil (INCT-IBISAB). Brazil - Fortaleza Malnutrition and Enteric Disease Study 2009-2014.	Ceará	2009-2014	Survey
Diarrheal diseases	Fogarty International Center, National Institutes of Health (NIH), Foundation for the National Institutes of Health (FNIH), National Institute of Science, Technology, and Biomedicine of Semi-Arid Brazil (INCT-IBISAB). Brazil - Fortaleza Malnutrition and Enteric Disease Study 2009-2014.	Ceará	2009-2014	Survey
Diarrheal diseases	Fogarty International Center, National Institutes of Health (NIH), Foundation for the National Institutes of Health (FNIH), National Institute of Science, Technology, and Biomedicine of Semi-Arid Brazil (INCT-IBISAB). Brazil - Fortaleza Malnutrition and Enteric Disease Study 2009-2014.	Ceará	2009-2014	Survey
Diarrheal diseases	Fogarty International Center, National Institutes of Health (NIH), Foundation for the National Institutes of Health (FNIH), National Institute of Science, Technology, and Biomedicine of Semi-Arid Brazil (INCT-IBISAB). Brazil - Fortaleza Malnutrition and Enteric Disease Study 2009-2014.	Ceará	2009-2014	Survey
Diarrheal diseases	admission of Brazilian children with non-rotavirus diarrhoea: a case control-study. . 2015; 109(7): 454-61.		2008-2011	Scientific literature
Diarrheal diseases	admission of Brazilian children with non-rotavirus diarrhoea: a case control-study. . 2015; 109(7): 454-61.		2008-2011	Scientific literature
Diarrheal diseases	The prevalence of norovirus, astrovirus and adenovirus infections among hospitalised children with acute gastroenteritis in Porto Velho, state of Rondonia, western Brazilian Amazon. . 2015; 110(2): 215-21.		2010-2012	Scientific literature
Diarrheal diseases	The prevalence of norovirus, astrovirus and adenovirus infections among hospitalised children with acute gastroenteritis in Porto Velho, state of Rondonia, western Brazilian Amazon. . 2015; 110(2): 215-21.		2010-2012	Scientific literature

Diarrheal diseases	The prevalence of norovirus, astrovirus and adenovirus infections among hospitalised children with acute gastroenteritis in Porto Velho, state of Rondonia, western Brazilian Amazon. . 2015; 110(2): 215-21.		2010-2012	Scientific literature
Diarrheal diseases	Fogarty International Center, National Institutes of Health (NIH), Foundation for the National Institutes of Health (FNIH), National Institute of Science, Technology, and Biomedicine of Semi-Arid Brazil (INCT-IBISAB). Brazil - Fortaleza Malnutrition and Enteric Disease Study 2009-2014.	Ceará	2009-2014	Survey
Diarrheal diseases	Guerrant RL, Kirchhoff LV, Shields DS, Nations MK, Leslie J, de Sousa MA, Araujo JG, Correia LL, Sauer KT, McClelland KE. Prospective study of diarrheal illnesses in northeastern Brazil: patterns of disease, nutritional impact, etiologies, and risk factors. . 1983; 148(6): 986-97.		1978-1980	Scientific literature
Diarrheal diseases	Dulgheroff AC, Figueiredo EF, Moreira LP, Moreira KC, Moura LM, Gouv[illegible] VS, Domingues AL. Distribution of rotavirus genotypes after vaccine introduction in the Triängulo Mineiro region of Brazil: 4-Year follow-up study. . 2012; 55(1): 67-71.		2007-2010	Scientific literature
Diarrheal diseases	Siqueira JA, Linhares Ada C, de Carvalho TC, AragÜo GC, Oliveira Dde S, Dos Santos MC, de Sousa MS, Justino MC, Mascarenhas JD, Gabbay YB. Norovirus infection in children admitted to hospital for acute gastroenteritis in Belém, Parà, Northern Brazil. . 2013; 85(4): 737-44.		2008-2010	Scientific literature
Diarrheal diseases	Linhares AC, Monção HC, Gabbay YB, de Araújo VL, Serruya AC, Loureiro EC. Acute diarrhoea associated with rotavirus among children living in Belém, Brazil. . 1983; 77(3): 384-90.		1979-1980	Scientific literature
Diarrheal diseases	Linhares AC, Monção HC, Gabbay YB, de Araújo VL, Serruya AC, Loureiro EC. Acute diarrhoea associated with rotavirus among children living in Belém, Brazil. . 1983; 77(3): 384-90.		1979-1980	Scientific literature
Diarrheal diseases	Linhares AC, Monção HC, Gabbay YB, de Araújo VL, Serruya AC, Loureiro EC. Acute diarrhoea associated with rotavirus among children living in Belém, Brazil. . 1983; 77(3): 384-90.		1979-1980	Scientific literature
Diarrheal diseases	Linhares AC, Monção HC, Gabbay YB, de Araújo VL, Serruya AC, Loureiro EC. Acute diarrhoea associated with rotavirus among children living in Belém, Brazil. . 1983; 77(3): 384-90.		1979-1980	Scientific literature
Diarrheal diseases	Rácz ML, Candeias JA, Trabulsi JR, Murahowski J. Diarrheal diseases in Brazil: clinical features of rotavirus-associated gastroenteritis in children. . 1988; 4(3): 382-5.		1986-1987	Scientific literature
Diarrheal diseases	Rácz ML, Candeias JA, Trabulsi JR, Murahowski J. Diarrheal diseases in Brazil: clinical features of rotavirus-associated gastroenteritis in children. . 1988; 4(3): 382-5.		1986-1987	Scientific literature
Diarrheal diseases	Nunes MRCM, Magalhães PP, Penna FJ, Nunes JMM, Mendes EN. Diarrhea associated with Shigella in children and susceptibility to antimicrobials. . 2012; 88(2): 125-8.		2004-2007	Scientific literature
Diarrheal diseases	Nunes MRCM, Magalhães PP, Penna FJ, Nunes JMM, Mendes EN. Diarrhea associated with Shigella in children and susceptibility to antimicrobials. . 2012; 88(2): 125-8.		2004-2007	Scientific literature
Diarrheal diseases	Nunes AA, de Mello LM, Parrode RN, Bittar JPM, Domingues AL da S. Prevalence of rotavirus in acute diarrhea and its association with clinical signs and symptoms. . 2010; 56(3): 212-3.		2005-2007	Scientific literature
Diarrheal diseases	Nunes AA, de Mello LM, Parrode RN, Bittar JPM, Domingues AL da S. Prevalence of rotavirus in acute diarrhea and its association with clinical signs and symptoms. . 2010; 56(3): 212-3.		2005-2007	Scientific literature
Diarrheal diseases	Sáfadi MAP, Berezin EN, Munford V, Almeida FJ, de Moraes JC, Pinheiro CF, Racz ML. Hospital-based surveillance to evaluate the impact of rotavirus vaccination in São Paulo, Brazil. . 2010; 29(11): 1019-22.		2004-2008	Scientific literature
Diarrheal diseases	Sáfadi MAP, Berezin EN, Munford V, Almeida FJ, de Moraes JC, Pinheiro CF, Racz ML. Hospital-based surveillance to evaluate the impact of rotavirus vaccination in São Paulo, Brazil. . 2010; 29(11): 1019-22.		2004-2008	Scientific literature
Diarrheal diseases	Parashar UD, Cunliffe NA, Nakagomi T. Effectiveness of monovalent rotavirus vaccine (Rotarix) against severe diarrhea caused by serotypically unrelated G2P[4] strains in Brazil. . 2010; 201(3): 363-9.		2006-2008	Scientific literature
Diarrheal diseases	Parashar UD, Cunliffe NA, Nakagomi T. Effectiveness of monovalent rotavirus vaccine (Rotarix) against severe diarrhea caused by serotypically unrelated G2P[4] strains in Brazil. . 2010; 201(3): 363-9.		2006-2008	Scientific literature
Diarrheal diseases	Dulgheroff AC, Figueiredo EF, Moreira LP, Moreira KC, Moura LM, Gouv[illegible] VS, Domingues AL. Distribution of rotavirus genotypes after vaccine introduction in the Triängulo Mineiro region of Brazil: 4-Year follow-up study. . 2012; 55(1): 67-71.		2007-2010	Scientific literature
Diarrheal diseases	Gurgel RG, Bohland AK, Vieira SCF, Oliveira DMP, Fontes PB, Barros VF, Ramos MF, Dove W, Nakagomi T, Nakagomi O, Correia JB, Cunliffe N, Cuevas LE. Incidence of rotavirus and all-cause diarrhea in northeast Brazil following the introduction of a national vaccination program. . 2009; 137(6): 1970-5.		2004-2008	Scientific literature
Diarrheal diseases	Sartori AMC, Valentim J, de Soárez PC, Novaes HMD. Rotavirus morbidity and mortality in children in Brazil. . 2008; 23(2): 92-100.		1986-2006	Scientific literature
Diarrheal diseases	Fang GD, Lima AA, Martins CV, Nataro JP, Guerrant RL. Etiology and epidemiology of persistent diarrhea in northeastern Brazil: a hospital-based, prospective, case-control study. . 1995; 21(2): 137-44.		1988-1991	Scientific literature
Diarrheal diseases	World Health Organization (WHO). WHO Global Health Observatory - Cholera: Number of Reported Cases by Country. Geneva, Switzerland: World Health Organization (WHO).	Global	1991-2011	Epi surveillance
Diarrheal diseases	World Health Organization (WHO). WHO Global Health Observatory - Cholera: Number of Reported Cases by Country. Geneva, Switzerland: World Health Organization (WHO).	Global	1991-2011	Epi surveillance
Diarrheal diseases	Brazilian Center for Analysis and Planning (CEBRAP), Brazilian Institute of Public Opinion and Statistics (IBOPE), Ministry of Health (Brazil). Brazil National Demographic and Health Survey of Children and Women 2006-2007. Rio de Janeiro, Brazil: Ministry of Health (Brazil).	Country	2006-2007	Survey
Diarrheal diseases	Health Institute (São Paulo, Brazil), State University of Campinas, São Paulo Municipal Health Department, São Paulo State University, University of São Paulo. Brazil - São Paulo Health Survey 2008-2009.	São Paulo	2002-2008	Survey
Diarrheal diseases	Brazilian Institute of Geography and Statistics (IBGE), Ministry of Health (Brazil), Ministry of Planning, Budget, and Management (Brazil). Brazil National Health Survey 2013. Rio de Janeiro, Brazil: Brazilian Institute of Geography and Statistics (IBGE).	Country	2013-2014	Survey
Diarrheal diseases	Norwegian Institute of Public Health. Norway Cause of Death Registry 1982. Oslo, Norway: Norwegian Institute of Public Health.	Country	2007-2008	Vital registration
Diarrheal diseases	Norwegian Institute of Public Health. Norway Cause of Death Registry 1982. Oslo, Norway: Norwegian Institute of Public Health.	Country	2007-2008	Vital registration
Diarrheal diseases	Norwegian Institute of Public Health. Norway Cause of Death Registry 1982. Oslo, Norway: Norwegian Institute of Public Health.	Country	2007-2008	Vital registration
Diarrheal diseases	Norwegian Institute of Public Health. Norway Cause of Death Registry 1982. Oslo, Norway: Norwegian Institute of Public Health.	Country	2007-2008	Vital registration
Diarrheal diseases	Sousa M[illegible]Mendes EN, Collares GB, P?ret-Filho LA, Penna FJ, MagalhÜes PP. Shigella in Brazilian children with acute diarrhoea: prevalence, antimicrobial resistance and virulence genes. . 2013; 108(1): 30-5.		2004-2005	Scientific literature
Diarrheal diseases	Sousa M[illegible]Mendes EN, Collares GB, P?ret-Filho LA, Penna FJ, MagalhÜes PP. Shigella in Brazilian children with acute diarrhoea: prevalence, antimicrobial resistance and virulence genes. . 2013; 108(1): 30-5.		2004-2005	Scientific literature
Diarrheal diseases	Siqueira JA, Linhares Ada C, de Carvalho TC, AragÜo GC, Oliveira Dde S, Dos Santos MC, de Sousa MS, Justino MC, Mascarenhas JD, Gabbay YB. Norovirus infection in children admitted to hospital for acute gastroenteritis in Belém, Parà, Northern Brazil. . 2013; 85(4): 737-44.		2008-2010	Scientific literature
Diarrheal diseases	Gurgel RG, Bohland AK, Vieira SCF, Oliveira DMP, Fontes PB, Barros VF, Ramos MF, Dove W, Nakagomi T, Nakagomi O, Correia JB, Cunliffe N, Cuevas LE. Incidence of rotavirus and all-cause diarrhea in northeast Brazil following the introduction of a national vaccination program. . 2009; 137(6): 1970-5.		2006-2008	Scientific literature
Diarrheal diseases	Fang GD, Lima AA, Martins CV, Nataro JP, Guerrant RL. Etiology and epidemiology of persistent diarrhea in northeastern Brazil: a hospital-based, prospective, case-control study. . 1995; 21(2): 137-44.		1988-1991	Scientific literature
Diarrheal diseases	The prevalence of norovirus, astrovirus and adenovirus infections among hospitalised children with acute gastroenteritis in Porto Velho, state of Rondonia, western Brazilian Amazon. . 2015; 110(2): 215-21.		2010-2012	Scientific literature
Diarrheal diseases	Brazilian Society for Family Welfare (BEMFAM), Westinghouse; Institute for Resource Development. Brazil Demographic and Health Survey 1986. Columbia, United States: Westinghouse; Institute for Resource Development.	Country	1986	Survey
Diarrheal diseases	Longitudinal study of Cryptosporidium infection in children in northeastern Brazil. . 1999; 180(1): 167-75.		1989-1993	Scientific literature
Diarrheal diseases	Schorling JB, Wanke CA, Schorling SK, McAuliffe JF, De Souza MA, Guerrant RL. A prospective study of persistent diarrhea among children in an urban Brazilian slum. Patterns of occurrence and etiologic agents. . 1990; 132(1): 144-56.		1985-1986	Scientific literature
Diarrheal diseases	Brazilian Society for Family Welfare (BEMFAM), Macro International, Inc. Brazil Demographic and Health Survey 1991. Calverton, United States: Macro International, Inc.	Country	1991	Survey
Diarrheal diseases	Schorling JB, Wanke CA, Schorling SK, McAuliffe JF, De Souza MA, Guerrant RL. A prospective study of persistent diarrhea among children in an urban Brazilian slum. Patterns of occurrence and etiologic agents. . 1990; 132(1): 144-56.		1985-1986	Scientific literature
Diarrheal diseases	Lima AA, Moore SR, Barboza MS Jr, Soares AM, Schleupner MA, Newman RD, Sears CL, Nataro JP, Fedorko DP, Wuhib T, Schorling JB, Guerrant RL. Persistent diarrhea signals a critical period of increased diarrhea burdens and nutritional shortfalls: a prospective cohort study among children in northeastern Brazil. . 2000; 181(5): 1643-51.		1989-1993	Scientific literature

Diarrheal diseases	Brazilian Society for Family Welfare (BEMFAM), Macro International, Inc. Brazil Demographic and Health Survey 1996. Calverton, United States: Macro International, Inc.	Country	1996	Survey
Diarrheal diseases	Schorling JB, Wanke CA, Schorling SK, McAuliffe JF, De Souza MA, Guerrant RL. A prospective study of persistent diarrhea among children in an urban Brazilian slum. Patterns of occurrence and etiologic agents. . 1990; 132(1): 144-56.		1985-1986	Scientific literature
Diarrheal diseases	Lima AA, Moore SR, Barboza MS Jr, Soares AM, Schleupner MA, Newman RD, Sears CL, Nataro JP, Fedorko DP, Wuhib T, Schorling JB, Guerrant RL. Persistent diarrhea signals a critical period of increased diarrhea burdens and nutritional shortfalls: a prospective cohort study among children in northeastern Brazil. . 2000; 181(5): 1643-51.		1989-1993	Scientific literature
Diarrheal diseases	Brazil World Health Survey 2003	Country	2002-2003	Survey
Diarrheal diseases	Schorling JB, Wanke CA, Schorling SK, McAuliffe JF, De Souza MA, Guerrant RL. A prospective study of persistent diarrhea among children in an urban Brazilian slum. Patterns of occurrence and etiologic agents. . 1990; 132(1): 144-56.		1985-1986	Scientific literature
Diarrheal diseases	Longitudinal study of Cryptosporidium infection in children in northeastern Brazil. . 1999; 180(1): 167-75.		1989-1993	Scientific literature
Diarrheal diseases	Lima AA, Moore SR, Barboza MS Jr, Soares AM, Schleupner MA, Newman RD, Sears CL, Nataro JP, Fedorko DP, Wuhib T, Schorling JB, Guerrant RL. Persistent diarrhea signals a critical period of increased diarrhea burdens and nutritional shortfalls: a prospective cohort study among children in northeastern Brazil. . 2000; 181(5): 1643-51.		1989-1993	Scientific literature
Diarrheal diseases	Lima AA, Moore SR, Barboza MS Jr, Soares AM, Schleupner MA, Newman RD, Sears CL, Nataro JP, Fedorko DP, Wuhib T, Schorling JB, Guerrant RL. Persistent diarrhea signals a critical period of increased diarrhea burdens and nutritional shortfalls: a prospective cohort study among children in northeastern Brazil. . 2000; 181(5): 1643-51.		1989-1993	Scientific literature
Diarrheal diseases	Lima AA, Moore SR, Barboza MS Jr, Soares AM, Schleupner MA, Newman RD, Sears CL, Nataro JP, Fedorko DP, Wuhib T, Schorling JB, Guerrant RL. Persistent diarrhea signals a critical period of increased diarrhea burdens and nutritional shortfalls: a prospective cohort study among children in northeastern Brazil. . 2000; 181(5): 1643-51.		1989-1993	Scientific literature
Diarrheal diseases	Schorling JB, Wanke CA, Schorling SK, McAuliffe JF, De Souza MA, Guerrant RL. A prospective study of persistent diarrhea among children in an urban Brazilian slum. Patterns of occurrence and etiologic agents. . 1990; 132(1): 144-56.		1985-1986	Scientific literature
Diarrheal diseases	Lima AA, Moore SR, Barboza MS Jr, Soares AM, Schleupner MA, Newman RD, Sears CL, Nataro JP, Fedorko DP, Wuhib T, Schorling JB, Guerrant RL. Persistent diarrhea signals a critical period of increased diarrhea burdens and nutritional shortfalls: a prospective cohort study among children in northeastern Brazil. . 2000; 181(5): 1643-51.		1989-1993	Scientific literature
Diarrheal diseases	Lima AA, Moore SR, Barboza MS Jr, Soares AM, Schleupner MA, Newman RD, Sears CL, Nataro JP, Fedorko DP, Wuhib T, Schorling JB, Guerrant RL. Persistent diarrhea signals a critical period of increased diarrhea burdens and nutritional shortfalls: a prospective cohort study among children in northeastern Brazil. . 2000; 181(5): 1643-51.		1989-1993	Scientific literature
Diarrheal diseases	Ministry of Health (Brazil). Brazil Hospital Information System 1997. Rio de Janeiro, Brazil: Ministry of Health (Brazil).	Country	1993-1997	Administrative record
Diarrheal diseases	Lima AA, Moore SR, Barboza MS Jr, Soares AM, Schleupner MA, Newman RD, Sears CL, Nataro JP, Fedorko DP, Wuhib T, Schorling JB, Guerrant RL. Persistent diarrhea signals a critical period of increased diarrhea burdens and nutritional shortfalls: a prospective cohort study among children in northeastern Brazil. . 2000; 181(5): 1643-51.		1989-1993	Scientific literature
Diarrheal diseases	Barreto ML, Santos LM, Assis AM, Araújo MP, Farenzena GG, Santos PA, Fiaccone RL. Effect of vitamin A supplementation on diarrhoea and acute lower-respiratory-tract infections in young children in Brazil. . 1994; 344(8917): 228-31.		1990-1991	Scientific literature
Diarrheal diseases	Lima AA, Moore SR, Barboza MS Jr, Soares AM, Schleupner MA, Newman RD, Sears CL, Nataro JP, Fedorko DP, Wuhib T, Schorling JB, Guerrant RL. Persistent diarrhea signals a critical period of increased diarrhea burdens and nutritional shortfalls: a prospective cohort study among children in northeastern Brazil. . 2000; 181(5): 1643-51.		1989-1993	Scientific literature
Intestinal infectious diseases	Ministry of Health (Brazil). Brazil Information System for Notifiable Diseases - Typhoid Fever.	Country	2001-2011	Epi surveillance
Lower respiratory infections	Ministry of Health (Brazil). Brazil Hospital Information System 1997. Rio de Janeiro, Brazil: Ministry of Health (Brazil).	Country	1993-1997	Administrative record
Lower respiratory infections	Health Institute (São Paulo, Brazil), State University of Campinas, São Paulo Municipal Health Department, São Paulo State University, University of São Paulo. Brazil - São Paulo Health Survey 2008-2009.	São Paulo	2002-2008	Survey
Lower respiratory infections	Buzzo AR, Roberts C, Mollinedo LG, Quevedo JM, Casas GL, Soldevilla JMS. Morbidity and mortality of pneumonia in adults in six Latin American countries. . 2013; 17(9): e673-677.		2009	Scientific literature
Lower respiratory infections	Ministry of Health (Brazil). Brazil Hospital Information System 1998-2002.		1998-2002	Administrative record
Lower respiratory infections	Salomão Junior JB, Gardinassi LGA, Simas PVM, Bittar CO, Souza FP, Rahal P, Zanetta DMT. Human respiratory syncytial virus in children hospitalized for acute lower respiratory infection. . 2011; 87(3): 219[illegible]24.		2004-2005	Scientific literature
Lower respiratory infections	Guatura SB, Watanabe AS, Camargo CN, Passos AM, Parmezan SN, Tomazella TK, Carraro E, Kamikawa J, Granato C, Bellei N. Surveillance of influenza A H1N1 2009 among school children during 2009 and 2010 in São Paulo, Brazil. . 2012; 45(5): 563-6.		2009-2010	Scientific literature
Lower respiratory infections	Guatura SB, Watanabe AS, Camargo CN, Passos AM, Parmezan SN, Tomazella TK, Carraro E, Kamikawa J, Granato C, Bellei N. Surveillance of influenza A H1N1 2009 among school children during 2009 and 2010 in São Paulo, Brazil. . 2012; 45(5): 563-6.		2009-2010	Scientific literature
Lower respiratory infections	Brazilian Institute of Geography and Statistics (IBGE), Ministry of Health (Brazil), Ministry of Planning, Budget, and Management (Brazil). Brazil National Health Survey 2013. Rio de Janeiro, Brazil: Brazilian Institute of Geography and Statistics (IBGE).	Country	2013-2014	Survey
Lower respiratory infections	Brazil World Health Survey 2003	Country	2002-2003	Survey
Lower respiratory infections	Ministry of Health (Brazil). Brazil Hospital Information System 2013-2014.		2013-2017	Administrative record
Lower respiratory infections	Ministry of Health (Brazil). Brazil Hospital Information System 2008-2012.		2008-2012	Administrative record
Lower respiratory infections	Ministry of Health (Brazil). Brazil Hospital Information System 2003-2007.		2003-2007	Administrative record
Lower respiratory infections	Salomão Junior JB, Gardinassi LGA, Simas PVM, Bittar CO, Souza FP, Rahal P, Zanetta DMT. Human respiratory syncytial virus in children hospitalized for acute lower respiratory infection. . 2011; 87(3): 219[illegible]24.		2004-2005	Scientific literature
Upper respiratory infections	Cunha A. Relationship between acute respiratory infection and malnutrition in children under 5 years of age. . 2000; 5(89): 608-609.		1991	Scientific literature
Upper respiratory infections	Brazilian Society for Family Welfare (BEMFAM), Macro International, Inc. Brazil Demographic and Health Survey 1991. Calverton, United States: Macro International, Inc.	Country	1991	Survey
Upper respiratory infections	Brazilian Society for Family Welfare (BEMFAM), Macro International, Inc. Brazil Demographic and Health Survey 1996. Calverton, United States: Macro International, Inc.	Country	1996	Survey
Otitis media	Godinho RN, Gonçalves TM, Nunes FB, Becker CG, Becker HM, Guimarães RE, Sanfins F, Colosimo EA, Oliveira RG, Lamounier JA. Prevalence and impact of chronic otitis media in school age children in Brazil First epidemiologic study concerning chronic otitis media in Latin America. . 2001; 61(3): 223-32.		1999-2001	Scientific literature
Meningitis	Ministry of Health (Brazil). Brazil Hospital Information System 1997. Rio de Janeiro, Brazil: Ministry of Health (Brazil).	Country	1993-1997	Administrative record
Meningitis	Health Institute (São Paulo, Brazil), State University of Campinas, São Paulo Municipal Health Department, São Paulo State University, University of São Paulo. Brazil - São Paulo Health Survey 2008-2009.	São Paulo	2002-2008	Survey
Meningitis	Brazilian Institute of Geography and Statistics (IBGE), Ministry of Health (Brazil), Ministry of Planning, Budget, and Management (Brazil). Brazil National Health Survey 2013. Rio de Janeiro, Brazil: Brazilian Institute of Geography and Statistics (IBGE).	Country	2013-2014	Survey
Meningitis	Ministry of Health (Brazil). Brazil Hospital Information System 1998-2002.		1998-2002	Administrative record
Meningitis	Ministry of Health (Brazil). Brazil Hospital Information System 2003-2007.		2003-2007	Administrative record
Meningitis	Facklam RR, Reis MG, Ko AI. Population-based survey of antimicrobial susceptibility and serotype distribution of Streptococcus pneumoniae from meningitis patients in Salvador, Brazil. . 2002; 40(1): 275-7.		1995-1999	Scientific literature
Meningitis	Ministry of Health (Brazil). Brazil Hospital Information System 2008-2012.		2008-2012	Administrative record
Meningitis	Berezin EN, Carvalho ES, Casagrande S, Brandileone MC, Mimica IM, Farhat CK. Streptococcus pneumoniae penicillin-nonsusceptible strains in invasive infections in Sao Paulo, Brazil. . 1996; 15(11): 1051-3.		1989-1993	Scientific literature
Meningitis	Ministry of Health (Brazil). Brazil Hospital Information System 2013-2014.		2013-2017	Administrative record
Meningitis	Brazil World Health Survey 2003	Country	2002-2003	Survey
Meningitis	Souza SF de, Costa M da CN, Paim JS, Natividade MS da, Pereira SM, Andrade AM de S, Teixeira MG. Bacterial meningitis and living conditions. . 2012; 45(3): 323-8.		2004-2009	Scientific literature
Pneumococcal meningitis	Antoniuk SA, Zanon França M, Tannous Tahan T, Oliveira Rossoni AM, Dal-Ri Moreira S, Rodrigues Cruz C, Dal-Pra Ducci R, Hamdar F, Tieko Frare Kira A. Study of 312 children with meningitis treated at a University Hospital in the South of Brazil. . 2009; 69(1 Pt 1): 127-32.		2003-2007	Scientific literature
Pneumococcal meningitis	Souza SF de, Costa M da CN, Paim JS, Natividade MS da, Pereira SM, Andrade AM de S, Teixeira MG. Bacterial meningitis and living conditions. . 2012; 45(3): 323-8.		2004-2009	Scientific literature

H influenzae type B meningitis	Antoniuk SA, Zanon França M, Tannous Tahan T, Oliveira Rossoni AM, Dal-Ri Moreira S, Rodrigues Cruz C, Dal-Pra Ducci R, Hamdar F, Tieko Frare Kira A. Study of 312 children with meningitis treated at a University Hospital in the South of Brazil. . 2009; 69(1 Pt 1): 127-32.		2003-2007	Scientific literature
H influenzae type B meningitis	Souza SF de, Costa M da CN, Paim JS, Natividade MS da, Pereira SM, Andrade AM de S, Teixeira MG. Bacterial meningitis and living conditions. . 2012; 45(3): 323-8.		2004-2009	Scientific literature
Meningococcal meningitis	Souza SF de, Costa M da CN, Paim JS, Natividade MS da, Pereira SM, Andrade AM de S, Teixeira MG. Bacterial meningitis and living conditions. . 2012; 45(3): 323-8.		2004-2009	Scientific literature
Meningococcal meningitis	Antoniuk SA, Zanon França M, Tannous Tahan T, Oliveira Rossoni AM, Dal-Ri Moreira S, Rodrigues Cruz C, Dal-Pra Ducci R, Hamdar F, Tieko Frare Kira A. Study of 312 children with meningitis treated at a University Hospital in the South of Brazil. . 2009; 69(1 Pt 1): 127-32.		2003-2007	Scientific literature
Other meningitis	Souza SF de, Costa M da CN, Paim JS, Natividade MS da, Pereira SM, Andrade AM de S, Teixeira MG. Bacterial meningitis and living conditions. . 2012; 45(3): 323-8.		2004-2009	Scientific literature
Other meningitis	Antoniuk SA, Zanon França M, Tannous Tahan T, Oliveira Rossoni AM, Dal-Ri Moreira S, Rodrigues Cruz C, Dal-Pra Ducci R, Hamdar F, Tieko Frare Kira A. Study of 312 children with meningitis treated at a University Hospital in the South of Brazil. . 2009; 69(1 Pt 1): 127-32.		2003-2007	Scientific literature
Encephalitis	Brazil World Health Survey 2003	Country	2002-2003	Survey
Encephalitis	Brazilian Institute of Geography and Statistics (IBGE), Ministry of Health (Brazil), Ministry of Planning, Budget, and Management (Brazil). Brazil National Health Survey 2013. Rio de Janeiro, Brazil: Brazilian Institute of Geography and Statistics (IBGE).	Country	2013-2014	Survey
Encephalitis	Ministry of Health (Brazil). Brazil Hospital Information System 2013-2014.		2013-2017	Administrative record
Encephalitis	Ministry of Health (Brazil). Brazil Hospital Information System 2008-2012.		2008-2012	Administrative record
Encephalitis	Ministry of Health (Brazil). Brazil Hospital Information System 2003-2007.		2003-2007	Administrative record
Encephalitis	Health Institute (São Paulo, Brazil), State University of Campinas, São Paulo Municipal Health Department, São Paulo State University, University of São Paulo. Brazil - São Paulo Health Survey 2008-2009.	São Paulo	2002-2008	Survey
Encephalitis	Ministry of Health (Brazil). Brazil Hospital Information System 1998-2002.		1998-2002	Administrative record
Encephalitis	Ministry of Health (Brazil). Brazil Hospital Information System 1997. Rio de Janeiro, Brazil: Ministry of Health (Brazil).	Country	1993-1997	Administrative record
Whooping cough	UNICEF Reported Disease Incidence Time Series. Geneva, Switzerland: World Health Organization (WHO).	Global	1982-2015	Epi surveillance
Measles	UNICEF Reported Disease Incidence Time Series. Geneva, Switzerland: World Health Organization (WHO).	Global	1980-2015	Epi surveillance
Varicella and herpes zoster	of children with different social behaviour in the State of São Paulo, Brazil. . 2001; 127(3): 493-500.		1992-1998	Scientific literature
Varicella and herpes zoster	Yu AL, Costa JM, Amaku M, Pannuti CS, Souza VA, Zanetta DM, Burattini MN, Massad E, Azevedo RS. Three year seroepidemiological study of varicella-zoster virus in São Paulo, Brazil. . 2000; 42(3): 125-8.		1992-1994	Scientific literature
Malaria	Seasonal variation of anti-Plasmodium falciparum antibodies directed against a repetitive peptide of gametocyte antigen pfs2400 in the State of Amap , Brazil as it appears in Malaria Atlas Project. Malaria Atlas Project Plasmodium Falciparum Parasite Rate Database. Oxford, United Kingdom: Malaria Atlas Project.		1995	Scientific literature
Malaria	Amazon basin: to treat or not to treat? as it appears in Malaria Atlas Project. Malaria Atlas Project Plasmodium Falciparum Parasite Rate Database. Oxford, United Kingdom: Malaria Atlas Project.		2006	Scientific literature
Malaria	SPf66 vaccine trial in Brazil: conceptual framework study design and analytical approach as it appears in Malaria Atlas Project. Malaria Atlas Project Plasmodium Falciparum Parasite Rate Database. Oxford, United Kingdom: Malaria Atlas Project.		1991	Scientific literature
Malaria	Malaria is associated with poor school performance in an endemic area of the Brazilian Amazon as it appears in Malaria Atlas Project. Malaria Atlas Project Plasmodium Falciparum Parasite Rate Database. Oxford, United Kingdom: Malaria Atlas Project.		2008	Scientific literature
Malaria	Amazon basin of Brazil as it appears in Malaria Atlas Project. Malaria Atlas Project Plasmodium Falciparum Parasite Rate Database. Oxford, United Kingdom: Malaria Atlas Project.		1987	Scientific literature
Malaria	Sorologia da malaria vivax no foco Aldeia dos Indios, Municipio de Peruibe, Estado de Sao Paulo, 1984 a 1986 as it appears in Malaria Atlas Project. Malaria Atlas Project Plasmodium Falciparum Parasite Rate Database. Oxford, United Kingdom: Malaria Atlas Project.		1985-1986	Scientific literature
Malaria	Epidemiology of disappearing Plasmodium vivax malaria: a case study in rural Amazonia as it appears in Malaria Atlas Project. Malaria Atlas Project Plasmodium Falciparum Parasite Rate Database. Oxford, United Kingdom: Malaria Atlas Project.		2010-2013	Scientific literature
Malaria	Naturally acquired antibodies to Plasmodium vivax Duffy binding protein (DBP) in Brazilian Amazon as it appears in Malaria Atlas Project. Malaria Atlas Project Plasmodium Falciparum Parasite Rate Database. Oxford, United Kingdom: Malaria Atlas Project.	Country	2004-2005	Scientific literature
Malaria	Concurrent helminthic infection protects schoolchildren with Plasmodium vivax from anemia as it appears in Malaria Atlas Project. Malaria Atlas Project Plasmodium Falciparum Parasite Rate Database. Oxford, United Kingdom: Malaria Atlas Project.	Country	2008	Scientific literature
Malaria	Studies on Malaria in Serra do Navio Region, Amapa State, Brazil as it appears in Malaria Atlas Project. Malaria Atlas Project Plasmodium Falciparum Parasite Rate Database. Oxford, United Kingdom: Malaria Atlas Project.	Amapá	1989-1991	Report
Malaria	Epidemiology of Malaria and Factors Associated with Asymptomatic Plasmodium Infection in a Population of Goldmongers of the Brazilian Amazon as it appears in Malaria Atlas Project. Malaria Atlas Project Plasmodium Falciparum Parasite Rate Database. Oxford, United Kingdom: Malaria Atlas Project.	Amazonas	1996	Report
Malaria	Brazil Plasmodium Falciparum Parasite Rate Data, Personal Communication with A.M. Siqueira, Instituto Nacional de Infectologia Evandro Chagas-Fiocruz, Rio de Janeiro 2015 as it appears in Malaria Atlas Project. Malaria Atlas Project Plasmodium Falciparum Parasite Rate Database. Oxford, United Kingdom: Malaria Atlas Project.	Country	2008-2010	Survey
Malaria	in the Amazon Rio Negro area, Brazil as it appears in Malaria Atlas Project. Malaria Atlas Project Plasmodium Falciparum Parasite Rate Database. Oxford, United Kingdom: Malaria Atlas Project.		2002-2004	Scientific literature
Malaria	Cross-sectional study defines difference in malaria morbidity in two Yanomami communities on Amazonian boundary between Brazil and Venezuela as it appears in Malaria Atlas Project. Malaria Atlas Project Plasmodium Falciparum Parasite Rate Database. Oxford, United Kingdom: Malaria Atlas Project.		1991	Scientific literature
Malaria	Diagnosis of health conditions in a pan-mining community in the Tapajós River Basin, Itaituba, Par , Brazil, 1992 as it appears in Malaria Atlas Project. Malaria Atlas Project Plasmodium Falciparum Parasite Rate Database. Oxford, United Kingdom: Malaria Atlas Project.		1992	Scientific literature
Malaria	Malaria epidemiology in low-endemicity areas of the Atlantic Forest in the Vale do Ribeira, São Paulo, Brazil as it appears in Malaria Atlas Project. Malaria Atlas Project Plasmodium Falciparum Parasite Rate Database. Oxford, United Kingdom: Malaria Atlas Project.		2002	Scientific literature
Malaria	Low Frequency of Anti-Plasmodium Falciparum Circumsporozoite Repeat Antibodies and Rate of High Malaria Transmission in Endemic Areas of Rondonia State in Northwestern Brazil as it appears in Malaria Atlas Project. Malaria Atlas Project Plasmodium Falciparum Parasite Rate Database. Oxford, United Kingdom: Malaria Atlas Project.		1986-1989	Scientific literature
Malaria	Naturally acquired humoral and cellular immune responses to Plasmodium vivax merozoite surface protein 9 in Northwestern Amazon individuals as it appears in Malaria Atlas Project. Malaria Atlas Project Plasmodium Falciparum Parasite Rate Database. Oxford, United Kingdom: Malaria Atlas Project.		2004	Scientific literature
Malaria	IL10A genotypic association with decreased IL-10 circulating levels in malaria infected individuals from endemic area of the Brazilian Amazon as it appears in Malaria Atlas Project. Malaria Atlas Project Plasmodium Falciparum Parasite Rate Database. Oxford, United Kingdom: Malaria Atlas Project.		2010-2011	Scientific literature
Malaria	World Malaria Report 2013 as it appears in Malaria Atlas Project. Malaria Atlas Project Annual Parasite Incidence Database.	Global	1990-1999	Report
Malaria	World Malaria Report 2015 as it appears in Malaria Atlas Project. Malaria Atlas Project Annual Parasite Incidence Database.	Global	2001-2009	Report
Malaria	World Malaria Report 2016 as it appears in Malaria Atlas Project. Malaria Atlas Project Annual Parasite Incidence Database.	Global	2000-2015	Report
Malaria	Brazil Epidemiological Surveillance Information System Malaria Case Notifications 2016 as it appears in Malaria Atlas Project. Malaria Atlas Project Annual Parasite Incidence Database.	Country	2009-2015	Epi surveillance
Malaria	Unstable hypoendemic malaria in Rondonia (western Amazon region, Brazil): epidemic outbreaks and work-associated incidence in an agro-industrial rural settlement as it appears in Malaria Atlas Project. Malaria Atlas Project Plasmodium Falciparum Parasite Rate Database. Oxford, United Kingdom: Malaria Atlas Project.		1991-1992	Scientific literature
Malaria	Velho, Rondônia, in the Amazon region of Brazil as it appears in Malaria Atlas Project. Malaria Atlas Project Plasmodium Falciparum Parasite Rate Database. Oxford, United Kingdom: Malaria Atlas Project.		2006	Scientific literature

Malaria	Antibodies anti Bloodstream and Circumsporozoite Antigens (Plasmodium vivax and Plasmodium malariae/P. brasilianum) in Areas of Very Low Malaria Endemicity in Brazil as it appears in Malaria Atlas Project. Malaria Atlas Project Plasmodium Falciparum Parasite Rate Database. Oxford, United Kingdom: Malaria Atlas Project.		1993-1994	Scientific literature
Malaria	from the state of Amapé, Brazil as it appears in Malaria Atlas Project. Malaria Atlas Project Plasmodium Falciparum Parasite Rate Database. Oxford, United Kingdom: Malaria Atlas Project.		1992	Scientific literature
Malaria	native Amazonian populations as it appears in Malaria Atlas Project. Malaria Atlas Project Plasmodium Falciparum Parasite Rate Database. Oxford, United Kingdom: Malaria Atlas Project.		1998-2000	Scientific literature
Malaria	Urban malaria in the Brazilian Western Amazon Region I: high prevalence of asymptomatic carriers in an urban riverside district is associated with a high level of clinical malaria as it appears in Malaria Atlas Project. Malaria Atlas Project Plasmodium Falciparum Parasite Rate Database. Oxford, United Kingdom: Malaria Atlas Project.		2001-2004	Scientific literature
Malaria	Effects of immigration on the prevalence of malaria in rural areas of the Amazon basin of Brazil as it appears in Malaria Atlas Project. Malaria Atlas Project Plasmodium Falciparum Parasite Rate Database. Oxford, United Kingdom: Malaria Atlas Project.		1985-1986	Scientific literature
Malaria	[Effect of bed nets impregnated with deltamethrin on malaria morbidity in an area of the Brazilian Amazones] as it appears in Malaria Atlas Project. Malaria Atlas Project Plasmodium Falciparum Parasite Rate Database. Oxford, United Kingdom: Malaria Atlas Project.		1991	Scientific literature
Malaria	Mercury exposure and malaria prevalence among gold miners in Pará, Brazil as it appears in Malaria Atlas Project. Malaria Atlas Project Plasmodium Falciparum Parasite Rate Database. Oxford, United Kingdom: Malaria Atlas Project.		1997	Scientific literature
Malaria	Amazonian population as it appears in Malaria Atlas Project. Malaria Atlas Project Plasmodium Falciparum Parasite Rate Database. Oxford, United Kingdom: Malaria Atlas Project.		2004-2006	Scientific literature
Malaria	Antibody response to the N and C-terminal regions of the Plasmodium vivax Merozoite Surface Protein 1 in individuals living in an area of exclusive transmission of P. vivax malaria in the north of Brazil as it appears in Malaria Atlas Project. Malaria Atlas Project Plasmodium Falciparum Parasite Rate Database. Oxford, United Kingdom: Malaria Atlas Project.		1996-1997	Scientific literature
Malaria	Region of Rio Negro, Amazon] as it appears in Malaria Atlas Project. Malaria Atlas Project Plasmodium Falciparum Parasite Rate Database. Oxford, United Kingdom: Malaria Atlas Project.		2006	Scientific literature
Malaria	Amazon Basin of Brazil as it appears in Malaria Atlas Project. Malaria Atlas Project Plasmodium Falciparum Parasite Rate Database. Oxford, United Kingdom: Malaria Atlas Project.		2002-2003	Scientific literature
Malaria	parasites, including a variant of Plasmodium vivax, in the population of two epidemiologically distinct areas in the state of Acre, Brazil as it appears in Malaria Atlas Project. Malaria Atlas Project Plasmodium Falciparum Parasite Rate Database. Oxford, United Kingdom: Malaria Atlas Project.		1990	Scientific literature
Malaria	[Malaria and hematological aspects among residents to be impacted by reservoirs for the Santo Antônio and Jirau Hydroelectric Power Stations, Rondônia State, Brazil] as it appears in Malaria Atlas Project. Malaria Atlas Project Plasmodium Falciparum Parasite Rate Database. Oxford, United Kingdom: Malaria Atlas Project.		2004-2005	Scientific literature
Malaria	Plasmodium vivax Duffy binding protein: baseline antibody responses and parasite polymorphisms in a well-consolidated settlement of the Amazon Region as it appears in Malaria Atlas Project. Malaria Atlas Project Plasmodium Falciparum Parasite Rate Database. Oxford, United Kingdom: Malaria Atlas Project.		2008	Scientific literature
Malaria	Epidemiological and ecological aspects related to malaria in the area of influence of the lake at Porto Primavera dam, in western Sao Paulo State, Brazil as it appears in Malaria Atlas Project. Malaria Atlas Project Plasmodium Falciparum Parasite Rate Database. Oxford, United Kingdom: Malaria Atlas Project.		2000	Scientific literature
Malaria	Epidemiology of malaria in a hypoendemic Brazilian Amazon migrant population: a cohort study as it appears in Malaria Atlas Project. Malaria Atlas Project Plasmodium Falciparum Parasite Rate Database. Oxford, United Kingdom: Malaria Atlas Project.		1996-1997	Scientific literature
Malaria	enzyme immunoassay using bloodspot eluates as it appears in Malaria Atlas Project. Malaria Atlas Project Plasmodium Falciparum Parasite Rate Database. Oxford, United Kingdom: Malaria Atlas Project.		1991	Scientific literature
Malaria	Duffy-negative and Duffy-positive individuals as it appears in Malaria Atlas Project. Malaria Atlas Project Plasmodium Falciparum Parasite Rate Database. Oxford, United Kingdom: Malaria Atlas Project.		2009	Scientific literature
Malaria	risk groups in an urban locality as it appears in Malaria Atlas Project. Malaria Atlas Project Plasmodium Falciparum Parasite Rate Database. Oxford, United Kingdom: Malaria Atlas Project.		1990	Scientific literature
Malaria	The epidemiology of malaria in Rondonia (Western Amazon region, Brazil): study of a riverine population as it appears in Malaria Atlas Project. Malaria Atlas Project Plasmodium Falciparum Parasite Rate Database. Oxford, United Kingdom: Malaria Atlas Project.		1994-1995	Scientific literature
Malaria	The Wai Wai Indians of South America: history and genetics as it appears in Malaria Atlas Project. Malaria Atlas Project Plasmodium Falciparum Parasite Rate Database. Oxford, United Kingdom: Malaria Atlas Project.		1988	Scientific literature
Malaria	Brazilian Society for Family Welfare (BEMFAM), Macro International, Inc. Brazil Demographic and Health Survey 1991. Calverton, United States: Macro International, Inc.	Country	1991	Survey
Malaria	Brazilian Society for Family Welfare (BEMFAM), Macro International, Inc. Brazil Demographic and Health Survey 1996. Calverton, United States: Macro International, Inc.	Country	1996	Survey
Malaria	Malaria prevalence amongst Brazilian Indians assessed by a new mathematical model as it appears in Malaria Atlas Project. Malaria Atlas Project Plasmodium Falciparum Parasite Rate Database. Oxford, United Kingdom: Malaria Atlas Project.		1985	Scientific literature
Malaria	parasites in five states of the Amazon region of Brazil as it appears in Malaria Atlas Project. Malaria Atlas Project Plasmodium Falciparum Parasite Rate Database. Oxford, United Kingdom: Malaria Atlas Project.		1995-2003	Scientific literature
Malaria	Humoral immune response to the 72 kDa heat shock protein from Plasmodium falciparum in populations at hypoendemic areas of malaria in western Brazilian Amazon as it appears in Malaria Atlas Project. Malaria Atlas Project Plasmodium Falciparum Parasite Rate Database. Oxford, United Kingdom: Malaria Atlas Project.		1995	Scientific literature
Malaria	Parasitological Survey of the Population of Guariba-Colniza for Identification of Individuals with Asymptomatic Infection by Plasmodium, Colniza, Mato Grosso as it appears in Malaria Atlas Project. Malaria Atlas Project Plasmodium Falciparum Parasite Rate Database. Oxford, United Kingdom: Malaria Atlas Project.	Mato Grosso	1996-2006	Report
Malaria	Soroprevalência da infecção pelo vírus da hepatite B e pelo plasmódio em Lábrea, Amazonas: estimativa da ocorrência de prováveis coinfecções as it appears in Malaria Atlas Project. Malaria Atlas Project Plasmodium Falciparum Parasite Rate Database. Oxford, United Kingdom: Malaria Atlas Project.		2000	Scientific literature
Malaria	Assessment of Mercury Exposure and Malaria in a Brazilian Amazon Riverine Community as it appears in Malaria Atlas Project. Malaria Atlas Project Plasmodium Falciparum Parasite Rate Database. Oxford, United Kingdom: Malaria Atlas Project.		1999	Scientific literature
Malaria	Epidemiologic aspects of the malaria transmission cycle in an area of very low incidence in Brazil as it appears in Malaria Atlas Project. Malaria Atlas Project Plasmodium Falciparum Parasite Rate Database. Oxford, United Kingdom: Malaria Atlas Project.		2002-2003	Scientific literature
Malaria	Institute for Health Metrics and Evaluation (IHME). IHME GBD 2013 DisMod model input -	Global	1990-2016	Modeled data
Chagas disease	Surveillance of Trypanosoma cruzi transmission by serological screening of schoolchildren. . 1992; 70(5): 6259.		1991	Scientific literature
Chagas disease	Pan American Health Organization (PAHO), Southern Cone Initiative (INCOSUR). XIth Meeting of the Intergovernmental Committee for the Elimination of Triatoma Infestans and the Interruption of American Trypanosomiasis by Transfusion. Washington, D.C., United States: Pan American Health Organization (PAHO), 2002.	Country	2001	Report
Chagas disease	Carvalho EOC de, Rosa JA da, de Carvalho AA, Chaves HCO, Souza EA de, Ostermayer AL, Camargo LMA de. Study on Chagas disease occurrence in the municipality of Monte Negro, State of Rondônia, Brazilian Amazon. . 2011; 44(6): 703-7.		2007-2010	Scientific literature
Chagas disease	Ostermayer AL, Passos ADC, Silveira AC, Ferreira AW, Macedo V, Prata AR. The national survey of seroprevalence for evaluation of the control of Chagas disease in Brazil (2001-2008). . 2011; 44(Suppl 2): 108-21.		2001-2008	Scientific literature
Chagas disease	Lima-Costa MF, Matos DL, Ribeiro ALP. Chagas disease predicts 10-year stroke mortality in community-dwelling elderly: the Bambui cohort study of aging. . 2010; 41(11): 2477-82.	Country	1997	Scientific literature
Chagas disease	Silva EM da, Rocha MO da C, Silva RC, Paixão G do C, Buzzati H, Santos AN, Nunes M do CP. Clinic and epidemiological study on Chagas disease in the Serra Azul district of Mateus Leme, central-western region of the State of Minas Gerais, Brazil. . 2010; 43(2): 178-81.		2007	Scientific literature

Chagas disease	Araújo AB, Castagno VD, Gallina T, Berne MEA. Prevalence of Chagas disease among pregnant women in the southern region of Rio Grande do Sul. . 2009; 42(6): 732-3.	Country	2004	Scientific literature
Chagas disease	Silva RA da, Goldenberg P. Chagas' disease in Porto Letícia, São Paulo: a comparative study in the Pontal the Paranapanema. . 2008; 41(6): 621-7.		2003-2005	Scientific literature
Chagas disease	Pan American Health Organization (PAHO), World Health Organization (WHO). Quantitative Estimation of Chagas in the Americas.	Global	2005	Epi surveillance
Chagas disease	Salles G, Xavier S, Sousa A, Hasslocher-Moreno A, Cardoso C. Prognostic value of QT interval parameters for mortality risk stratification in Chagas' disease: results of a long-term follow-up study [Unpublished data]. . 2003; 108(3): 305-12.		1990-2015	Scientific literature
Chagas disease	Coutinho CF de S, Souza-Santos R, Teixeira NFD, Georg I, Gomes TF, Boia MN, dos Reis NB, Maia A de O, Lima MM. An entomoepidemiological investigation of Chagas disease in the state of Ceará, Northeast Region of Brazil. . 2014; 30(4): 785[illegible]3.		2008-2009	Scientific literature
Chagas disease	Ribeiro AL, dos Reis AM, Barros MV, de Sousa MR, Rocha AL, Perez AA, Pereira JB, Machado FS, Rocha MO. Brain natriuretic peptide and left ventricular dysfunction in Chagas' disease. . 2002; 360(9331): 461-2.		1999-2001	Scientific literature
Chagas disease	Brito CR do N, Sampaio GHF, Câmara ACJ da, Nunes DF, Azevedo PRM de, Chiari E, Galvão LM da C. Seroepidemiology of Trypanosoma cruzi infection in the semiarid rural zone of the State of Rio Grande do Norte, Brazil. . 2012; 45(3): 346[illegible]2.		2007-2009	Scientific literature
Chagas disease	Magalhães BML, Coelho LIARC, Maciel MG, Ferreira JMBB, Umezawa ES, Coura JR, Guerra JA de O, Barbosa M das GV. Serological survey for Chagas disease in the rural areas of Manaus, Coari, and Tefé in the Western Brazilian Amazon. . 2011; 44(5): 697[illegible]02.		2007-2008	Scientific literature
Chagas disease	Ribeiro AL, Marcolino MS, Prineas RJ, Lima-Costa MF. Electrocardiographic abnormalities in elderly Chagas disease patients: 10-year follow-up of the Bambuí Cohort Study of Aging. . 2014; 3(1): e000632.		2011	Scientific literature
Chagas disease	Borges-Pereira J, Sarquis O, Zauza PL, Britto C, Lima MM. [Epidemiology of Chagas disease in four rural localities in Jaguaruana, State of Ceará: seroprevalence of infection, parasitemia and clinical characteristics]. . 2008; 41(4): 345[illegible]1.		2000-2002	Scientific literature
Chagas disease	Coura JR, Naranjo MA, Willcox HP. Chagas[illegible]disease in the Brazilian Amazon. II. A serological survey. . 1995; 37(2): 103[illegible].		1974-1994	Scientific literature
Chagas disease	Institute for Health Metrics and Evaluation (IHME). Modeled Chagas Birth Prevalence		1990-2016	Modeled data
Chagas disease	Nastari L, Fernandes F, Patavino GM, Sachdev V, Capuani L, de Almeida-Neto C, Carrick DM, Wright D, Kavounis K, Goncalez TT, Carneiro-Proietti AB, Custer B, Busch MP, Murphy EL; National Heart, Lung, and Blood Institute Retrovirus Epidemiology Donor Study-II (REDS-II), International Component. Ten-Year Incidence of Chagas Cardiomyopathy Among Asymptomatic Trypanosoma cruzi[illegible]eropositive Former Blood Donors. . 2013; 127(10): 1105-15.		1996-2010	Scientific literature
Chagas disease	Destination and Origin - 2013 Revision. New York City, United States: United Nations Population Division.	Global	1990-2013	Administrative record
Chagas disease	Nunes MC, Kreuser LJ, Ribeiro AL, Sousa GR, Costa HS, Botoni FA, de Souza AC, Gomes Marques VE, Fernandez AB, Teixeira AL, da Costa Rocha MO. Prevalence and risk factors of embolic cerebrovascular events associated with Chagas heart disease [Unpublished data]. . 2015; 10(3): 151-7.		1990-2015	Scientific literature
Visceral leishmaniasis	Health Institute (São Paulo, Brazil), State University of Campinas, São Paulo Municipal Health Department, São Paulo State University, University of São Paulo. Brazil - São Paulo Health Survey 2008-2009.	São Paulo	2002-2008	Survey
Visceral leishmaniasis	Ministry of Health (Brazil). Brazil Information System for Notifiable Diseases 2008.	Country	2008	Epi surveillance
Visceral leishmaniasis	Ministry of Health (Brazil). Brazil Information System for Notifiable Diseases 2007.	Country	2007	Epi surveillance
Visceral leishmaniasis	Ministry of Health (Brazil). Brazil Information System for Notifiable Diseases 2004.	Country	2004	Epi surveillance
Visceral leishmaniasis	Ministry of Health (Brazil). Brazil Information System for Notifiable Diseases 2003.	Country	2003	Epi surveillance
Visceral leishmaniasis	Ministry of Health (Brazil). Brazil Information System for Notifiable Diseases 2002.	Country	2002	Epi surveillance
Visceral leishmaniasis	Ministry of Health (Brazil). Brazil Information System for Notifiable Diseases 2001.	Country	2000-2001	Epi surveillance
Visceral leishmaniasis	Ministry of Health (Brazil). Brazil Information System for Notifiable Diseases 2009.	Country	2009	Epi surveillance
Visceral leishmaniasis	areas for surveillance and control in a visceral leishmaniasis endemic area in Brazil. . 2014; 56-62.		2005	Scientific literature
Visceral leishmaniasis	World Health Organization (WHO). Brazil WHO Leishmaniasis Country Profile 1984-2010.	Country	1984-1997	Epi surveillance
Visceral leishmaniasis	Souza VAF de, Cortez LRP de B, Dias RA, Amaku M, Ferreira Neto JS, Kuroda RB dos S, Ferreira F. Space-time cluster analysis of American visceral leishmaniasis in Bauru, São Paulo State, Brazil. . 2012; 28(10): 1949-64.		2003-2007	Scientific literature
Visceral leishmaniasis	Ministry of Health (Brazil). Brazil Information System for Notifiable Diseases 2005.	Country	2005	Epi surveillance
Visceral leishmaniasis	Barata RA, Peixoto JC, Tanure A, Gomes ME, Apolinário EC, Bodevan EC, de Araújo HS, Dias ES, Pinheiro Ada C. Epidemiology of visceral leishmaniasis in a reemerging focus of intense transmission in Minas Gerais State, Brazil. . 2013; 405083.		2009	Scientific literature
Visceral leishmaniasis	Ministry of Health (Brazil). Brazil Information System for Notifiable Diseases 2011.	Country	2011	Epi surveillance
Visceral leishmaniasis	World Health Organization (WHO). WHO Global Health Observatory Interactive Graph - Number of Cases of Visceral Leishmaniasis Reported 2005. Geneva, Switzerland: World Health Organization (WHO).	Global	2005	Epi surveillance
Visceral leishmaniasis	World Health Organization (WHO). WHO Global Health Observatory Interactive Graph - Number of Cases of Visceral Leishmaniasis Reported 2013. Geneva, Switzerland: World Health Organization (WHO).	Global	2013	Epi surveillance
Visceral leishmaniasis	World Health Organization (WHO). WHO Global Health Observatory Interactive Graph - Number of Cases of Visceral Leishmaniasis Reported 2012. Geneva, Switzerland: World Health Organization (WHO).	Global	2012	Epi surveillance
Visceral leishmaniasis	World Health Organization (WHO). WHO Global Health Observatory Interactive Graph - Number of Cases of Visceral Leishmaniasis Reported 2011. Geneva, Switzerland: World Health Organization (WHO).	Global	2011	Epi surveillance
Visceral leishmaniasis	World Health Organization (WHO). WHO Global Health Observatory Interactive Graph - Number of Cases of Visceral Leishmaniasis Reported 2010. Geneva, Switzerland: World Health Organization (WHO).	Global	2010	Epi surveillance
Visceral leishmaniasis	World Health Organization (WHO). WHO Global Health Observatory Interactive Graph - Number of Cases of Visceral Leishmaniasis Reported 2009. Geneva, Switzerland: World Health Organization (WHO).	Global	2009	Epi surveillance
Visceral leishmaniasis	World Health Organization (WHO). WHO Global Health Observatory Interactive Graph - Number of Cases of Visceral Leishmaniasis Reported 2008. Geneva, Switzerland: World Health Organization (WHO).	Global	2008	Epi surveillance
Visceral leishmaniasis	World Health Organization (WHO). WHO Global Health Observatory Interactive Graph - Number of Cases of Visceral Leishmaniasis Reported 2007. Geneva, Switzerland: World Health Organization (WHO).	Global	2007	Epi surveillance
Visceral leishmaniasis	World Health Organization (WHO). WHO Global Health Observatory Interactive Graph - Number of Cases of Visceral Leishmaniasis Reported 2006. Geneva, Switzerland: World Health Organization (WHO).	Global	2006	Epi surveillance
Visceral leishmaniasis	World Health Organization (WHO). WHO Global Health Observatory Interactive Graph - Number of Cases of Visceral Leishmaniasis Reported 2004. Geneva, Switzerland: World Health Organization (WHO).	Global	2004	Epi surveillance
Visceral leishmaniasis	World Health Organization (WHO). WHO Global Health Observatory Interactive Graph - Number of Cases of Visceral Leishmaniasis Reported 2003. Geneva, Switzerland: World Health Organization (WHO).	Global	2003	Epi surveillance
Visceral leishmaniasis	World Health Organization (WHO). WHO Global Health Observatory Interactive Graph - Number of Cases of Visceral Leishmaniasis Reported 2001. Geneva, Switzerland: World Health Organization (WHO).	Global	2001	Epi surveillance
Visceral leishmaniasis	World Health Organization (WHO). WHO Global Health Observatory Interactive Graph - Number of Cases of Visceral Leishmaniasis Reported 2015. Geneva, Switzerland: World Health Organization (WHO).	Global	2015	Epi surveillance
Visceral leishmaniasis	World Health Organization (WHO). WHO Global Health Observatory Interactive Graph - Number of Cases of Visceral Leishmaniasis Reported 2000. Geneva, Switzerland: World Health Organization (WHO).	Global	2000	Epi surveillance
Visceral leishmaniasis	World Health Organization (WHO). WHO Global Health Observatory Interactive Graph - Number of Cases of Visceral Leishmaniasis Reported 1998. Geneva, Switzerland: World Health Organization (WHO).	Global	1998	Epi surveillance
Visceral leishmaniasis	Ministry of Health (Brazil). Brazil Information System for Notifiable Diseases 2010.	Country	2010	Epi surveillance
Visceral leishmaniasis	World Health Organization (WHO). WHO Global Health Observatory Interactive Graph - Number of Cases of Cutaneous Leishmaniasis Reported 2008. Geneva, Switzerland: World Health Organization (WHO).	Global	2002	Epi surveillance
Visceral leishmaniasis	Pigott DM, Bhatt S, Golding N, Duda KA, Battle KE, Brady OJ, Messina JP, Balard Y, Bastien P, Pratlong F, Brownstein JS, Freifeld CC, Mekaru SR, Gething PW, George DB, Myers MF, Reithinger R, Hay SI. Global distribution maps of the leishmaniases. . 2014; 3: nan.		1980-2016	Scientific literature

Visceral leishmaniasis	Brazilian Institute of Geography and Statistics (IBGE), Ministry of Health (Brazil), Ministry of Planning, Budget, and Management (Brazil). Brazil National Health Survey 2013. Rio de Janeiro, Brazil: Brazilian Institute of Geography and Statistics (IBGE).	Country	2013-2014	Survey
Visceral leishmaniasis	World Health Organization (WHO). Brazil WHO Leishmaniasis Country Profile 2014. Geneva, Switzerland: World Health Organization (WHO), 2016.	Country	2014	Epi surveillance
Visceral leishmaniasis	Ministry of Health (Brazil). Brazil Information System for Notifiable Diseases 2015.	Country	2015-2016	Epi surveillance
Visceral leishmaniasis	Ministry of Health (Brazil). Brazil Information System for Notifiable Diseases 2014.	Country	2014	Epi surveillance
Visceral leishmaniasis	Ministry of Health (Brazil). Brazil Information System for Notifiable Diseases 2006.	Country	2006	Epi surveillance
Visceral leishmaniasis	Brazil World Health Survey 2003	Country	2002-2003	Survey
Visceral leishmaniasis	Ministry of Health (Brazil). Brazil Information System for Notifiable Diseases 2012.	Country	2012	Epi surveillance
Visceral leishmaniasis	World Health Organization (WHO). WHO Global Health Observatory Interactive Graph - Number of Cases of Visceral Leishmaniasis Reported 1999. Geneva, Switzerland: World Health Organization (WHO).	Global	1999	Epi surveillance
Visceral leishmaniasis	Ministry of Health (Brazil). Brazil Information System for Notifiable Diseases 2013.	Country	2013	Epi surveillance
Cutaneous and mucocutaneous leishmaniasis	Ministry of Health (Brazil). Brazil Information System for Notifiable Diseases 2010.	Country	2010	Epi surveillance
Cutaneous and mucocutaneous leishmaniasis	Brazil World Health Survey 2003	Country	2002-2003	Survey
Cutaneous and mucocutaneous leishmaniasis	World Health Organization (WHO). WHO Global Health Observatory Interactive Graph - Number of Cases of Cutaneous Leishmaniasis Reported 2015. Geneva, Switzerland: World Health Organization (WHO).	Global	2015	Epi surveillance
Cutaneous and mucocutaneous leishmaniasis	World Health Organization (WHO). WHO Global Health Observatory Interactive Graph - Number of Cases of Cutaneous Leishmaniasis Reported 2013. Geneva, Switzerland: World Health Organization (WHO).	Global	2013	Epi surveillance
Cutaneous and mucocutaneous leishmaniasis	Brazilian Institute of Geography and Statistics (IBGE), Ministry of Health (Brazil), Ministry of Planning, Budget, and Management (Brazil). Brazil National Health Survey 2013. Rio de Janeiro, Brazil: Brazilian Institute of Geography and Statistics (IBGE).	Country	2013-2014	Survey
Cutaneous and mucocutaneous leishmaniasis	World Health Organization (WHO). WHO Global Health Observatory Interactive Graph - Number of Cases of Cutaneous Leishmaniasis Reported 2004. Geneva, Switzerland: World Health Organization (WHO).	Global	2004	Epi surveillance
Cutaneous and mucocutaneous leishmaniasis	World Health Organization (WHO). WHO Global Health Observatory Interactive Graph - Number of Cases of Cutaneous Leishmaniasis Reported 2010. Geneva, Switzerland: World Health Organization (WHO).	Global	2010	Epi surveillance
Cutaneous and mucocutaneous leishmaniasis	World Health Organization (WHO). WHO Global Health Observatory Interactive Graph - Number of Cases of Cutaneous Leishmaniasis Reported 2011. Geneva, Switzerland: World Health Organization (WHO).	Global	2011	Epi surveillance
Cutaneous and mucocutaneous leishmaniasis	Ministry of Health (Brazil). Brazil Information System for Notifiable Diseases 2013.	Country	2013	Epi surveillance
Cutaneous and mucocutaneous leishmaniasis	World Health Organization (WHO). WHO Global Health Observatory Interactive Graph - Number of Cases of Cutaneous Leishmaniasis Reported 2012. Geneva, Switzerland: World Health Organization (WHO).	Global	2012	Epi surveillance
Cutaneous and mucocutaneous leishmaniasis	World Health Organization (WHO). WHO Global Health Observatory Interactive Graph - Number of Cases of Cutaneous Leishmaniasis Reported 2003. Geneva, Switzerland: World Health Organization (WHO).	Global	2003	Epi surveillance
Cutaneous and mucocutaneous leishmaniasis	Ministry of Health (Brazil). Brazil Information System for Notifiable Diseases 2007.	Country	2007	Epi surveillance
Cutaneous and mucocutaneous leishmaniasis	Ministry of Health (Brazil). Brazil Information System for Notifiable Diseases 2012.	Country	2012	Epi surveillance
Cutaneous and mucocutaneous leishmaniasis	World Health Organization (WHO). Brazil WHO Leishmaniasis Country Profile 2014. Geneva, Switzerland: World Health Organization (WHO), 2016.	Country	2014	Epi surveillance
Cutaneous and mucocutaneous leishmaniasis	World Health Organization (WHO). Brazil WHO Leishmaniasis Country Profile 1984-2010.	Country	1989-2010	Epi surveillance
Cutaneous and mucocutaneous leishmaniasis	Guerra JA, Maciel MG, Guerra MV, Talhari AC, Prestes SR, Fernandes MA, Da-Cruz AM, Martins A, Coelho LI, Romero GA, Barbosa Md. Tegumentary leishmaniasis in the State of Amazonas: what have we learned and what do we need?. . 2015; 12-9.		1981-2006	Scientific literature
Cutaneous and mucocutaneous leishmaniasis	Fonseca Eda S, D'Andrea LA, Taniguchi HH, Hiramoto RM, Tolezano JE, Guimarães RB. Spatial epidemiology of American cutaneous leishmaniasis in a municipality of west São Paulo State, Brazil. . 2014; 51(4): 271-5.		1998-2011	Scientific literature
Cutaneous and mucocutaneous leishmaniasis	World Health Organization (WHO). WHO Global Health Observatory Interactive Graph - Number of Cases of Cutaneous Leishmaniasis Reported 1998. Geneva, Switzerland: World Health Organization (WHO).	Global	1998	Epi surveillance
Cutaneous and mucocutaneous leishmaniasis	World Health Organization (WHO). WHO Global Health Observatory Interactive Graph - Number of Cases of Cutaneous Leishmaniasis Reported 1999. Geneva, Switzerland: World Health Organization (WHO).	Global	1999	Epi surveillance
Cutaneous and mucocutaneous leishmaniasis	Ministry of Health (Brazil). Brazil Information System for Notifiable Diseases 2011.	Country	2011	Epi surveillance
Cutaneous and mucocutaneous leishmaniasis	World Health Organization (WHO). WHO Global Health Observatory Interactive Graph - Number of Cases of Cutaneous Leishmaniasis Reported 2005. Geneva, Switzerland: World Health Organization (WHO).	Global	2005	Epi surveillance
Cutaneous and mucocutaneous leishmaniasis	World Health Organization (WHO). WHO Global Health Observatory Interactive Graph - Number of Cases of Cutaneous Leishmaniasis Reported 2008. Geneva, Switzerland: World Health Organization (WHO).	Global	2008	Epi surveillance
Cutaneous and mucocutaneous leishmaniasis	Ministry of Health (Brazil). Brazil Information System for Notifiable Diseases 2009.	Country	2009	Epi surveillance
Cutaneous and mucocutaneous leishmaniasis	World Health Organization (WHO). WHO Global Health Observatory Interactive Graph - Number of Cases of Cutaneous Leishmaniasis Reported 2006. Geneva, Switzerland: World Health Organization (WHO).	Global	2006	Epi surveillance
Cutaneous and mucocutaneous leishmaniasis	World Health Organization (WHO). WHO Global Health Observatory Interactive Graph - Number of Cases of Cutaneous Leishmaniasis Reported 2007. Geneva, Switzerland: World Health Organization (WHO).	Global	2007-2014	Epi surveillance
Cutaneous and mucocutaneous leishmaniasis	World Health Organization (WHO). WHO Global Health Observatory Interactive Graph - Number of Cases of Cutaneous Leishmaniasis Reported 2009. Geneva, Switzerland: World Health Organization (WHO).	Global	2009	Epi surveillance
Cutaneous and mucocutaneous leishmaniasis	Health Institute (São Paulo, Brazil), State University of Campinas, São Paulo Municipal Health Department, São Paulo State University, University of São Paulo. Brazil - São Paulo Health Survey 2008-2009.	São Paulo	2002-2008	Survey
Cutaneous and mucocutaneous leishmaniasis	World Health Organization (WHO). WHO Global Health Observatory Interactive Graph - Number of Cases of Cutaneous Leishmaniasis Reported 2001. Geneva, Switzerland: World Health Organization (WHO).	Global	2001	Epi surveillance
Cutaneous and mucocutaneous leishmaniasis	World Health Organization (WHO). WHO Global Health Observatory Interactive Graph - Number of Cases of Cutaneous Leishmaniasis Reported 2002. Geneva, Switzerland: World Health Organization (WHO).	Global	2002	Epi surveillance
Cutaneous and mucocutaneous leishmaniasis	World Health Organization (WHO). WHO Global Health Observatory Interactive Graph - Number of Cases of Cutaneous Leishmaniasis Reported 2000. Geneva, Switzerland: World Health Organization (WHO).	Global	2000	Epi surveillance
Cutaneous and mucocutaneous leishmaniasis	Ministry of Health (Brazil). Brazil Information System for Notifiable Diseases 2008.	Country	2008	Epi surveillance
Schistosomiasis	GGL, Loverde PT, Correa-Oliveira R, Gazzinelli A. Schistosoma mansoni infection in a rural area of the Jequitinhonha Valley, Minas Gerais, Brazil: analysis of exposure risk. . 2010; 113(1): 34[illegible]41.		2001	Scientific literature
Schistosomiasis	Gomes EC de S, Barbosa CS. Spatial pattern, water use and risk levels associated with the transmission of schistosomiasis on the north coast of Pernambuco, Brazil. . 2010; 26(5): 1013[illegible]23.		2006-2007	Scientific literature
Schistosomiasis	Barbosa CS, Favre TC, Wanderley TN, Callou AC, Pieri OS. Assessment of schistosomiasis, through school surveys, in the Forest Zone of Pernambuco, Brazil. . 2006; 101 Suppl 1: 55[illegible]2.		2004	Scientific literature
Schistosomiasis	Guimarães ICS, Tavares-Neto J. [Urban transmission of schistosomiasis in children from a neighborhood of Salvador, Bahia]. . 2006; 39(5): 451[illegible].		2004	Scientific literature
Schistosomiasis	Moura EC, Bragazza LM, Coelho MF, Aun SM. [Prevalence of intestinal parasitosis in schoolchildren]. . 1997; 73(6): 406[illegible]0.		1994-1995	Scientific literature
Schistosomiasis	Soares MS, Barreto MG, da Silva CL, Pereira JB, Moza PG, Rey L, Calçado MS, Lustoza A, Maspero R. Schistosomiasis in a low prevalence area: incomplete urbanization increasing risk of infection in Paracambi, RJ, Brazil. . 1995; 90(4): 451[illegible].		1990-1991	Scientific literature
Schistosomiasis	Tashima NT, Simões MJS. Enteroparasitic occurrence in fecal samples analyzed at the University of Western São Paulo-UNOESTE Clinical Laboratory, Presidente Prudente, São Paulo state, Brazil. . 2004; 46(5): 243[illegible].		2001	Scientific literature
Schistosomiasis	Perez E, Gazin P, Furtado A, Miranda P, Marques NM, Silva MR, Varela R. [Intestinal parasite infections and schistosomiasis in a poor urban area, in townships of the sugar cane belt and in villages of the semi-arid area of North-East Brazil]. . 2000; 10(2): 127[illegible].		1993-1998	Scientific literature
Schistosomiasis	MCND, Freire RL, De Freitas JC, Santana MAZ, Navarro IT. Occurrence of enteroparasitosis in schoolchildren of the municipal district of Jataizinho, State of Paraná, Brazil. . 2006; 28(2): 107[illegible]1.		2004	Scientific literature
Schistosomiasis	London School of Hygiene and Tropical Medicine. Global Atlas of Helminth Infections - Schistosomiasis. London, United Kingdom: London School of Hygiene and Tropical Medicine.	Global	1995-2005	Estimate
Schistosomiasis	Institute for Health Metrics and Evaluation (IHME). IHME GBD Schistosomiasis DisMod Prevalence Estimates.	Global	1990-2016	Modeled data

Schistosomiasis	Institute for Health Metrics and Evaluation (IHME). IHME GBD Custom Model Estimates of Schistosomiasis Remission.	Global	2010-2012	Modeled data
Schistosomiasis	Bethony J, Williams JT, Kloos H, Blangero J, Alves-Fraga L, Buck G, Michalek A, Williams-Blangero S, Loverde PT, Corréa-Oliveira R, Gazzinelli A. Exposure to Schistosoma mansoni infection in a rural area in Brazil. II: household risk factors. . 2001; 6(2): 13645.		1999	Scientific literature
Schistosomiasis	the examination of compressed fecal samples for Schistosoma mansoni eggs. . 2003; 36(4): 5037.		1998-2000	Scientific literature
Schistosomiasis	Maranhense, state of Maranhão, Brazil: cross-sectional studies performed in 1987 and 1993. . 1998; 40(3): 16571.		1987-1993	Scientific literature
Schistosomiasis	Guimarães MD, de Barros HL, Katz N. A clinical epidemiologic study in a schistosomiasis mansoni endemic area (Tuparecê, Minas Gerais). . 1985; 27(3): 123-31.		1984-1985	Scientific literature
Schistosomiasis	Rodrigues RN, Murta C, Teixeira Júnior MA, Cury GC, Rocha MO. Clinical-epidemiologic study of schistosomiasis mansoni in Ponte do Pasmado, a village in the municipality of Itinga, state of Minas Gerais, Brazil, 1992. . 1995; 37(1): 815.		1992	Scientific literature
Schistosomiasis	Coutinho E, Barbosa FS, Barbosa JM, Pessoa P, Pinto RF, Oliveira PA, Rodrigues BA. Inquérito clínico-nutricional e antropométrico preliminar, em áreas endêmicas de esquistossomose mansônica, no Nordeste do Brasil. . 1972; 6: 211-36.		1965-1967	Scientific literature
Schistosomiasis	Assis AMO, Prado MS, Barreto ML, Reis MG, Conceição Pinheiro SM, Parraga IM, Blanton RE. Childhood stunting in Northeast Brazil: the role of Schistosoma mansoni infection and inadequate dietary intake. . 2004; 58(7): 1022-9.		2004	Scientific literature
Schistosomiasis	Assis AM, Barreto ML, Prado MS, Reis MG, Parraga IM, Blanton RE. Schistosoma mansoni infection and nutritional status in schoolchildren: a randomized, double-blind trial in northeastern Brazil. . 1998; 68(6): 1247-53.		1992-1993	Scientific literature
Schistosomiasis	Teixeira-Carvalho A, Silveira AMS. Effect of chemotherapy with praziquantel on the production of cytokines and morbidity associated with schistosomiasis mansoni. . 2008; 52(8): 2780-6.		2006	Scientific literature
Schistosomiasis	Coutinho EM, Abath FG, Barbosa CS, Domingues AL, Melo MC, Montenegro SM, Lucena MA, Romani SA, Souza WV, Coutinho AD. Factors involved in Schistosoma mansoni infection in rural areas of northeast Brazil. . 1997; 92(5): 707-15.		1994-1995	Scientific literature
Schistosomiasis	TAKEUCHI T. PARASITOLOGICAL AND SEROLOGICAL STUDIES ON AMEBIASIS AND OTHER INTESTINAL PARASITIC INFECTIONS IN THE RURAL SECTOR AROUND RECIFE, NORTHEAST BRAZIL. . 1990; 32(6): 42835.		1989	Scientific literature
Schistosomiasis	Kloetzel K, Chieffi PP, de Siqueira JG. Repeated mass treatment of schistosomiasis mansoni: experience in hyperendemic areas of Brazil. 3. Techniques for assessment and surveillance. . 1990; 84(1): 74-9.		1987-1990	Scientific literature
Schistosomiasis	infection with Schistosoma mansoni in a rural community in northeast Brazil. . 1976; 25(2): 285-94.		1972	Scientific literature
Schistosomiasis	differences in growth of school-aged children with schistosomiasis and geohelminth infection. . 1996; 55(2): 150-6.		1992	Scientific literature
Schistosomiasis	Brito LL, Barreto ML, Silva RDCR, Assis AMO, Reis MG, Parraga IM, Blanton RE. Moderate- and low-intensity co-infections by intestinal helminths and Schistosoma mansoni, dietary iron intake, and anemia in Brazilian children. . 2006; 75(5): 939-44.		1997	Scientific literature
Schistosomiasis	measures in relation to Schistosomiasis mansoni and socioeconomic variables. . 1988; 17(4): 880-6.		1981	Scientific literature
Schistosomiasis	De Lima e Costa MF, Rocha RS, Colley D, Gazzinelli G, Katz N. Validity of selected clinical signs and symptoms in diagnosis of Schistosoma mansoni infection. . 1991; 33(1): 12-7.		1986	Scientific literature
Schistosomiasis	sociodemographic characteristics and water contact patterns predictive of infection. . 1996; 25(6): 1292-300.		1991-1992	Scientific literature
Schistosomiasis	Gonçalves EM do N, Chieffi PP, Luna EJ de A, Pinho JRR, Carrilho FJ, Gryschek RCB. Comparative Study of the Accuracy of Different Techniques for the Laboratory Diagnosis of Schistosomiasis Mansoni in Areas of Low Endemicity in Barra Mansa City, Rio de Janeiro State, Brazil. . 2015; 2015: 135689.		2011	Scientific literature
Schistosomiasis	Palmeira DCC, Carvalho AG de, Rodrigues K, Couto JLA. [Prevalence of Schistosoma mansoni infection in two municipalities of the State of Alagoas, Brazil]. . 2010; 43(3): 3137.		2009	Scientific literature
Schistosomiasis	Proietti FA, Paulino UH, Chiari CA, Proietti AB, Antunes CM. Epidemiology of Schistosoma mansoni infection in a low-endemic area in Brazil: clinical and nutritional characteristics. . 1992; 34(5): 409-19.		1990-1992	Scientific literature
Schistosomiasis	Brito LL, Barreto ML, Silva Rde C, Assis AM, Reis MG, Parraga I, Blanton RE. [Risk factors for iron-deficiency anemia in children and adolescents with intestinal helminthic infections]. . 2003; 14(6): 422-31.		2002	Scientific literature
Schistosomiasis	Enk MJ, Lima ACL, Massara CL, Coelho PMZ, Schall VT. A combined strategy to improve the control of Schistosoma mansoni in areas of low prevalence in Brazil. . 2008; 78(1): 1406.		2005-2006	Scientific literature
Schistosomiasis	Schistosoma mansoni egg count in an hyperendemic area in the State of Minas Gerais. . 1985; 27(2): 66-75.		1984-1985	Scientific literature
Schistosomiasis	Chitsulo L, Engels D, Montresor A, Savioli L. The global status of schistosomiasis and its control. . 2000; 77(1): 41-51.	Global	1990-2016	Scientific literature
Cysticercosis	Valença MM, Valença LP. [Etiology of the epileptic seizures in Recife city, Brazil: study of 249 patients]. . 2000; 58(4): 106472.	Country	1987-1990	Scientific literature
Cysticercosis	Calcified cysticercotic lesions and intractable epilepsy: a cross sectional study of 512 patients		2003	Scientific literature
Cystic echinococcosis	Ministry of Health (Brazil). Brazil Hospital Information System 1997. Rio de Janeiro, Brazil: Ministry of Health (Brazil).	Country	1993-1997	Administrative record
Cystic echinococcosis	Health Institute (São Paulo, Brazil), State University of Campinas, São Paulo Municipal Health Department, São Paulo State University, University of São Paulo. Brazil - São Paulo Health Survey 2008-2009.	São Paulo	2002-2008	Survey
Cystic echinococcosis	Ministry of Health (Brazil). Brazil Hospital Information System 2013-2014.		2013-2017	Administrative record
Cystic echinococcosis	Ministry of Health (Brazil). Brazil Hospital Information System 2008-2012.		2008-2012	Administrative record
Cystic echinococcosis	Brazilian Institute of Geography and Statistics (IBGE), Ministry of Health (Brazil), Ministry of Planning, Budget, and Management (Brazil). Brazil National Health Survey 2013. Rio de Janeiro, Brazil: Brazilian Institute of Geography and Statistics (IBGE).	Country	2013-2014	Survey
Cystic echinococcosis	Ministry of Health (Brazil). Brazil Hospital Information System 2003-2007.		2003-2007	Administrative record
Cystic echinococcosis	Ministry of Health (Brazil). Brazil Hospital Information System 1998-2002.		1998-2002	Administrative record
Cystic echinococcosis	Cystic Echinococcosis Endemicity Estimates	Global	1980-2015	Scientific literature
Cystic echinococcosis	Brazil World Health Survey 2003	Country	2002-2003	Survey
Lymphatic filariasis	Institute for Health Metrics and Evaluation (IHME). IHME GBD Custom Model Estimates of Lymphatic Filiariasis Remission.	Global	2003-2016	Modeled data
Lymphatic filariasis	Institute for Health Metrics and Evaluation (IHME). IHME GBD Lymphatic Filariasis DisMod Prevalence Estimates.	Global	1990-2016	Modeled data
Lymphatic filariasis	Netto MJ, Bonfim C, Brandao E, Aguiar-Santos AM, Medeiros Z. Burden of lymphatic filariasis morbidity in an area of low endemicity in Brazil. . 2016; 163: 54-60.		2015	Scientific literature
Lymphatic filariasis	Brandao E, Bonfim C, Alves A, Oliveira C, Montenegro CE, Costa T, Maciel A, Medeiros Z. Lymphatic filariasis among children and adolescents: spatial identification via socio-environmental indicators to define priority areas for elimination. . 2015; 7(5): 324-31.		2014	Scientific literature
Lymphatic filariasis	World Health Organization (WHO). WHO PCT Databank - Lymphatic Filariasis. Geneva, Switzerland: World Health Organization (WHO).	Global	1990-2016	Epi surveillance
Lymphatic filariasis	Shriram AN, Krishnamoorthy K, Sivan A, Saha BP, Kumaraswami V, Vijayachari P. Impact of MDA and the prospects of elimination of the lone focus of diurnally sub periodic lymphatic filariasis in Nicobar Islands, India. . 2014; 93-7.		2011	Scientific literature
Lymphatic filariasis	Medeiros Z, Alves A, Brito JA, Borba L, Santos Z, Costa JP, do Espírito Santo ME, Netto MJE. The present situation regarding lymphatic filariasis in Cabo de Santo Agostinho, Pernambuco, Northeast Brazil. . 2006; 48(5): 263-7.		2000	Scientific literature
Lymphatic filariasis	Lymphatic Filariasis. London, United Kingdom: London School of Hygiene and Tropical Medicine.	Global	1990-2015	Epi surveillance
Lymphatic filariasis	Survey of Bancroftian filariasis infection in humans and Culex mosquitoes in the western Brazilian Amazon region: implications for transmission and control as it appears in London School of Hygiene and Tropical Medicine. Global Atlas of Helminth Infections - Lymphatic Filariasis. London, United Kingdom: London School of Hygiene and Tropical Medicine.		2008-2009	Scientific literature
Lymphatic filariasis	Korte RL, Fontes G, Camargo Jde S, Rocha EM, Araújo EA, Oliveira MZ, Santos RV, Camargo LM. Survey of Bancroftian filariasis infection in humans and Culex mosquitoes in the western Brazilian Amazon region: implications for transmission and control. . 2013; 46.0(2): 214-20.		2008-2009	Scientific literature
Lymphatic filariasis	it appears in London School of Hygiene and Tropical Medicine. Global Atlas of Helminth Infections - Lymphatic Filariasis. London, United Kingdom: London School of Hygiene and Tropical Medicine.		2007-2010	Scientific literature
Lymphatic filariasis	Aguiar-Santos AM, Medeiros Z, Bonfim C, Rocha AC, Brandão E, Miranda T, Oliveira P, Sarinho ESC. Epidemiological assessment of neglected diseases in children: lymphatic filariasis and soil-transmitted helminthiasis. . 2013; 89(3): 250-5.		2009-2010	Scientific literature

Lymphatic filariasis	Braga C, de Albuquerque MF, Schindler H, Rezende A, Maciel A, Silva MC, Furtado A, de Carvalho AB, Lapa T, Ximenes RA. [Epidemiological pattern of lymphatic filariasis in children living in endemic areas]. . 1997; 73(2): 95-100.		1990-1991	Scientific literature
Lymphatic filariasis	Fontes G, Rocha EM, Brito AC, Antunes CM. Lymphatic filariasis in Brazilian urban area (Maceió, Alagoas). . 1998; 93(6): 705-10.		1992-1995	Scientific literature
Lymphatic filariasis	A. Epidemiological study of bancroftian filariasis in Recife, northeastern Brazil. . 1996; 91(4): 449-55.		1991	Scientific literature
Lymphatic filariasis	Medeiros Z, Oliveira C, Quaresma J, Barbosa E, Aguiar-Santos AM, Bonfim C, Almeida J, Lessa F. Lymphatic filariasis in Moreno, Northeast Brazil. . 2004; 7(1): 73-9.		2001-2003	Scientific literature
Lymphatic filariasis	Brazil as it appears in London School of Hygiene and Tropical Medicine. Global Atlas of Helminth Infections - Lymphatic Filariasis. London, United Kingdom: London School of Hygiene and Tropical Medicine.		2000-2002	Scientific literature
Lymphatic filariasis	Oliveira P, Braga C, Alexander N, Brandão E, Silva A, Wanderley L, Aguiar AM, Diniz G, Medeiros Z, Rocha A. Evaluation of diagnostic tests for Wuchereria bancrofti infection in Brazilian schoolchildren. . 2014; 47.0(3): 359-66.		2007-2009	Scientific literature
Lymphatic filariasis	Medeiros Z, Bonfim C, Alves A, Oliveira C, Netto MJE, Aguiar-Santos AM. The epidemiological delimitation of lymphatic filariasis in an endemic area of Brazil, 41 years after the first recorded case. . 2008; 102(6): 509-19.		2000-2002	Scientific literature
Lymphatic filariasis	kernel density estimates to investigate lymphatic filariasis in northeast Brazil. . 2012; 106(2): 113-7.		2000-2002	Scientific literature
Lymphatic filariasis	Bancroftian filariasis in two urban areas of Recife, Brazil: pre-control observations on infection and disease as it appears in London School of Hygiene and Tropical Medicine. Global Atlas of Helminth Infections - Lymphatic Filariasis. London, United Kingdom: London School of Hygiene and Tropical Medicine.		1990-1991	Scientific literature
Lymphatic filariasis	Brandão E, Bonfim C, Cabral D, Lima JL, Aguiar-Santos AM, Maciel A, Medeiros Z. Mapping of Wuchereria bancrofti infection in children and adolescents in an endemic area of Brazil. . 2011; 120(1-2): 151-4.		2008-2010	Scientific literature
Lymphatic filariasis	appears in London School of Hygiene and Tropical Medicine. Global Atlas of Helminth Infections - Lymphatic Filariasis. London, United Kingdom: London School of Hygiene and Tropical Medicine.		1991	Scientific literature
Lymphatic filariasis	A socioenvironmental composite index as a tool for identifying urban areas at risk of lymphatic filariasis as it appears in London School of Hygiene and Tropical Medicine. Global Atlas of Helminth Infections - Lymphatic Filariasis. London, United Kingdom: London School of Hygiene and Tropical Medicine.		2000-2002	Scientific literature
Lymphatic filariasis	appears in London School of Hygiene and Tropical Medicine. Global Atlas of Helminth Infections - Lymphatic Filariasis. London, United Kingdom: London School of Hygiene and Tropical Medicine.		1993	Scientific literature
Lymphatic filariasis	urban areas of Alagoas State, Northeast Brazil: study in the general population]. . 2000; 33(6): 545-51.		1997-1999	Scientific literature
Lymphatic filariasis	[Bancroftian filariasis in urban areas of Alagoas State, Northeast Brazil: study in the general population] as it appears in London School of Hygiene and Tropical Medicine. Global Atlas of Helminth Infections - Lymphatic Filariasis. London, United Kingdom: London School of Hygiene and Tropical Medicine.		1995-2000	Scientific literature
Lymphatic filariasis	case of an endemic area in Jaboatão dos Guararapes, Pernambuco, Brazil] as it appears in London School of Hygiene and Tropical Medicine. Global Atlas of Helminth Infections - Lymphatic Filariasis. London, United Kingdom: London School of Hygiene and Tropical Medicine.		2000-2002	Scientific literature
Lymphatic filariasis	it appears in London School of Hygiene and Tropical Medicine. Global Atlas of Helminth Infections - Lymphatic Filariasis. London, United Kingdom: London School of Hygiene and Tropical Medicine.		1980-2003	Scientific literature
Lymphatic filariasis	[Evaluation of a social and environmental indicator used in the identification of lymphatic filariasis transmission in urban centers] as it appears in London School of Hygiene and Tropical Medicine. Global Atlas of Helminth Infections - Lymphatic Filariasis. London, United Kingdom: London School of Hygiene and Tropical Medicine.		1996-2001	Scientific literature
Lymphatic filariasis	as it appears in London School of Hygiene and Tropical Medicine. Global Atlas of Helminth Infections - Lymphatic Filariasis. London, United Kingdom: London School of Hygiene and Tropical Medicine.		1991-2000	Scientific literature
Lymphatic filariasis	Field evaluation of the whole blood immunochromatographic test for rapid bancroftian filariasis diagnosis in the northeast of Brazil as it appears in London School of Hygiene and Tropical Medicine. Global Atlas of Helminth Infections - Lymphatic Filariasis. London, United Kingdom: London School of Hygiene and Tropical Medicine.		1999	Scientific literature
Onchocerciasis	Banic DM, Calvão-Brito RHS, Marchon-Silva V, Schuertez JC, de Lima Pinheiro LR, da Costa Alves M, Têva A, Maia-Herzog M. Impact of 3 years ivermectin treatment on onchocerciasis in Yanomami communities in the Brazilian Amazon. . 2009; 112(2): 125[illegible]30.		2001-2004	Scientific literature
Onchocerciasis	Rassi E, Lacerda N, Guaimaraes JA. Study of the area affected by onchocerciasis in Brazil: survey of local residents. . 1976; 10(1): 33-45.		1974	Scientific literature
Onchocerciasis	Moraes MAP, Fraiha H, Chaves GM. Onchocercosis in Brazil. . 1973; 7(4): 50-6.		1973	Scientific literature
Onchocerciasis	of onchocerciasis in the area of the Toototobi river, Amazonas State, Brazil]. . 1978; 84(6): 510-9.		1976	Scientific literature
Onchocerciasis	Onchocerciasis Elimination Program for the Americas (OEPA). Onchocerciasis Elimination Program for the Americas. Guatemala City, Guatemala: Onchocerciasis Elimination Program for the Americas (OEPA).	Country	1950-2017	Epi surveillance
Trachoma	International Centre for Eye Health (ICEH). Brazil - Campinas Rapid Assessment of Avoidable Blindness 2004. Grootebroek, Netherlands: RAAB Repository.	São Paulo	2003	Survey
Trachoma	World Health Organization (WHO). WHO Global Health Observatory - Population Living in Trachoma Endemic Areas. Geneva, Switzerland: World Health Organization (WHO).	Global	2007-2012	Estimate
Trachoma	Carter Center, International Trachoma Initiative, London School of Hygiene and Tropical Medicine. Global Atlas of Trachoma. Decatur, United States: International Trachoma Initiative.	Global	2013	Estimate
Dengue	Pan American Health Organization (PAHO). Number of Reported Cases of Dengue and Dengue Hemorrhagic Fever (DHF) in the Americas, by Country 2003. Washington, D.C., United States: Pan American Health Organization (PAHO), 2005.	Global	2003	Epi surveillance
Dengue	Pan American Health Organization (PAHO). Number of Reported Cases of Dengue and Severe Dengue (SD) in the Americas, by Country 2014. Washington, D.C., United States: Pan American Health Organization (PAHO), 2015.	Global	2014	Epi surveillance
Dengue	Ministry of Health (Brazil). Brazil Information System for Notifiable Diseases 2009.	Country	2009	Epi surveillance
Dengue	Pan American Health Organization (PAHO). Number of Reported Cases of Dengue and Severe Dengue (DS) in the Americas, by Country 2013. Washington, D.C., United States: Pan American Health Organization (PAHO), 2014.	Global	2013	Epi surveillance
Dengue	Pan American Health Organization (PAHO). Number of Reported Cases of Dengue and Severe Dengue (DS) in the Americas, by Country 2012. Washington, D.C., United States: Pan American Health Organization (PAHO), 2013.	Global	2012	Epi surveillance
Dengue	Pan American Health Organization (PAHO). Number of Reported Cases of Dengue and Severe Dengue (DS) in the Americas, by Country 2011. Washington, D.C., United States: Pan American Health Organization (PAHO), 2012.	Global	2011	Epi surveillance
Dengue	Pan American Health Organization (PAHO). Number of Reported Cases of Dengue and Severe Dengue (DS) in the Americas, by Country 2010. Washington, D.C., United States: Pan American Health Organization (PAHO), 2011.	Global	2010	Epi surveillance
Dengue	Pan American Health Organization (PAHO). Number of Reported Cases of Dengue and Severe Dengue (DS) in the Americas, by Country 2009. Washington, D.C., United States: Pan American Health Organization (PAHO), 2010.	Global	2009	Epi surveillance
Dengue	Pan American Health Organization (PAHO). Number of Reported Cases of Dengue and Severe Dengue (DS) in the Americas, by Country 2008. Washington, D.C., United States: Pan American Health Organization (PAHO), 2009.	Global	2008	Epi surveillance
Dengue	Pan American Health Organization (PAHO). Number of Reported Cases of Dengue and Dengue Hemorrhagic Fever (DHF) in the Americas, by Country 2007. Washington, D.C., United States: Pan American Health Organization (PAHO), 2008.	Global	2007	Epi surveillance
Dengue	Pan American Health Organization (PAHO). Number of Reported Cases of Dengue and Dengue Hemorrhagic Fever (DHF) in the Americas, by Country 2006. Washington, D.C., United States: Pan American Health Organization (PAHO), 2007.	Global	2006	Epi surveillance
Dengue	Pan American Health Organization (PAHO). Number of Reported Cases of Dengue and Dengue Hemorrhagic Fever (DHF) in the Americas, by Country 2005. Washington, D.C., United States: Pan American Health Organization (PAHO), 2006.	Global	2005	Epi surveillance
Dengue	World Health Organization (WHO). WHO DengueNet. Geneva, Switzerland: World Health Organization (WHO).	Global	1988-2002	Epi surveillance
Dengue	Secretariat of Health Surveillance, Ministry of Health (Brazil). Brazil Dengue Incidence Rate--Indicators and Basic Data 2008. Rio de Janeiro, Brazil: Ministry of Health (Brazil), 2008.	Country	2003-2007	Epi surveillance

Dengue	Pan American Health Organization (PAHO). Number of Reported Cases of Dengue and Dengue Hemorrhagic Fever (DHF) in the Americas, by Country 2004. Washington, D.C., United States: Pan American Health Organization (PAHO), 2005.	Global	2004	Epi surveillance
Dengue	Ministry of Health (Brazil). Brazil Information System for Notifiable Diseases 2008.	Country	2008	Epi surveillance
Dengue	Ministry of Health (Brazil). Brazil Information System for Notifiable Diseases 2007.	Country	2007	Epi surveillance
Yellow fever	WHO Department of Communicable Disease Surveillance and Response. WHO Report on Global Surveillance of Epidemic-prone Infectious Diseases 2000.	Country	1980-1981	Epi surveillance
Yellow fever	Yellow fever in Africa and South America, 2007 - Weekly Epidemiological Record 2009	Country	2007	Epi surveillance
Yellow fever	Yellow fever in Africa and South America, 2006 - Weekly Epidemiological Record 2008	Country	2006	Epi surveillance
Yellow fever	Vasconcelos PF, Costa ZG, Travassos Da Rosa ES, Luna E, Rodrigues SG, Barros VL, Dias JP, Monteiro HA, Oliva OF, Vasconcelos HB, Oliveira RC, Sousa MR, Barbosa Da Silva J, Cruz AC, Martins EC, Travassos Da Rosa JF. Epidemic of jungle yellow fever in Brazil, 2000: implications of climatic alterations in disease spread. . 2001; 598-604.		2000	Scientific literature
Yellow fever	Barros VL, Da Rosa AP. An epidemic of sylvatic yellow fever in the southeast region of Maranhao State, Brazil, 1993-1994: epidemiologic and entomologic findings. . 1997; 57(2): 132-7.	Maranhão	1993	Scientific literature
Yellow fever	Vasconcelos PF da C. Febre amarela. . 2003; 36(2): 275-93.		1982-1998	Scientific literature
Yellow fever	UNICEF Reported Disease Incidence Time Series. Geneva, Switzerland: World Health Organization (WHO).	Global	1990-2015	Epi surveillance
Intestinal nematode infections	Global numbers of infection and disease burden of soil transmitted helminth infections in 2010 [Unpublished data]	Global	1990-2010	Scientific literature
Intestinal nematode infections	World Health Organization (WHO). WHO PCT Databank - Soil-transmitted Helminthiases. Geneva, Switzerland: World Health Organization (WHO).	Global	2003-2015	Estimate
Ascariasis	Global numbers of infection and disease burden of soil transmitted helminth infections in 2010 [Unpublished data]	Global	1990-2010	Scientific literature
Trichuriasis	Global numbers of infection and disease burden of soil transmitted helminth infections in 2010 [Unpublished data]	Global	1990-2010	Scientific literature
Hookworm disease	Global numbers of infection and disease burden of soil transmitted helminth infections in 2010 [Unpublished data]	Global	1990-2010	Scientific literature
Leprosy	Leprosy - Global situation - Weekly Epidemiological Record 2000	Global	1999	Epi surveillance
Leprosy	Leprosy - Weekly Epidemiological Record 2001	Global	2000	Epi surveillance
Leprosy	Global leprosy situation, September 1999 - Weekly Epidemiological Record 1999	Global	1998	Epi surveillance
Leprosy	Progress towards eliminating leprosy as a public health problem. Part I - Weekly Epidemiological Record 1994	Global	1993	Epi surveillance
Leprosy	Ministry of Health (Brazil). Brazil Information System for Notifiable Diseases - Leprosy.	Country	2001-2012	Epi surveillance
Leprosy	Progress towards leprosy elimination - Weekly Epidemiological Record 1997	Global	1996	Epi surveillance
Leprosy	Progress towards the elimination of leprosy as a public health problem. Part I - Weekly Epidemiological Record 1995	Global	1994	Epi surveillance
Leprosy	Progress towards the elimination of leprosy as a public health problem - Weekly Epidemiological Record 1996	Global	1995	Epi surveillance
Leprosy	Progress towards the elimination of leprosy as a public health problem - Weekly Epidemiological Record 1993	Country	1992	Epi surveillance
Zika virus	Ministry of Health (Brazil). Brazil Epidemiological Situation - Zika Data. Rio de Janeiro, Brazil: Ministry of Health (Brazil).	Country	2016	Epi surveillance
Zika virus	Pan American Health Organization (PAHO), World Health Organization (WHO). Cumulative Zika Confirmed and Suspected Cases Reported by Countries and Territories in the Americas 2015-2016, Updated as of February 12, 2016. Washington, D.C., United States: Pan American Health Organization (PAHO), 2016.		2015	Epi surveillance
Zika virus	dos Santos T, Rodriguez A, Almiron M, Sanhueza A, Ramon P, de Oliveira WK, Coelho GE, Badaró R, Cortez J, Ospina M, Pimentel R, Masis R, Hernandez F, Lara B, Montoya R, Jubithana B, Melchor A, Alvarez A, Aldighieri S, Dye C, Espinal MA. Zika Virus and the Guillain-Barré Syndrome - Case Series from Seven Countries. . 2016; 375(16): 1598-601.		2015	Scientific literature
Zika virus	Pan American Health Organization (PAHO), World Health Organization (WHO). Zika Cases and Congenital Syndrome Associated with Zika Virus, Reported by Countries and Territories in the Americas 2015-2016. Washington, D.C., United States: Pan American Health Organization (PAHO), 2016.	Country	2015-2016	Epi surveillance
Zika virus	Ministry of Health (Brazil). Epidemiology Report 2016 No. 18-57, Monitoring the Cases of Microcephaly in Brazil, Weeks 1-50. Rio de Janeiro, Brazil: Ministry of Health (Brazil), 2016.	Country	2015-2016	Epi surveillance
Maternal disorders	Galvão LPL, Alvim-Pereira F, de Mendonça CMM, Menezes FEF, Góis KA do N, Ribeiro RF, Gurgel RQ. The prevalence of severe maternal morbidity and near miss and associated factors in Sergipe, Northeast Brazil. . 2014; 25.		2011-2012	Scientific literature
Maternal disorders	Rocha Filho EA, Costa ML, Cecatti JG, Parpinelli MA, Haddad SM, Sousa MH, Melo EF, Surita FG, Souza JP, Brazilian Network for Surveillance of Severe Maternal Morbidity Study Group. Contribution of antepartum and intrapartum hemorrhage to the burden of maternal near miss and death in a national surveillance study. . 2015; 94(1): 50-8.		2009-2010	Scientific literature
Maternal disorders	Amaral E, Souza JP, Surita F, Luz AG, Sousa MH, Cecatti JG, Campbell O. A population-based surveillance study on severe acute maternal morbidity (near-miss) and adverse perinatal outcomes in Campinas, Brazil: the Vigimoma Project. . 2011; 11: 9.		2005	Scientific literature
Maternal hemorrhage	Galvão LPL, Alvim-Pereira F, de Mendonça CMM, Menezes FEF, Góis KA do N, Ribeiro RF, Gurgel RQ. The prevalence of severe maternal morbidity and near miss and associated factors in Sergipe, Northeast Brazil. . 2014; 25.		2011-2012	Scientific literature
Maternal hemorrhage	Rocha Filho EA, Costa ML, Cecatti JG, Parpinelli MA, Haddad SM, Sousa MH, Melo EF, Surita FG, Souza JP, Brazilian Network for Surveillance of Severe Maternal Morbidity Study Group. Contribution of antepartum and intrapartum hemorrhage to the burden of maternal near miss and death in a national surveillance study. . 2015; 94(1): 50-8.		2009-2010	Scientific literature
Maternal hemorrhage	Amaral E, Souza JP, Surita F, Luz AG, Sousa MH, Cecatti JG, Campbell O. A population-based surveillance study on severe acute maternal morbidity (near-miss) and adverse perinatal outcomes in Campinas, Brazil: the Vigimoma Project. . 2011; 11: 9.		2005	Scientific literature
Maternal sepsis and other maternal infections	Ministry of Health (Brazil). Brazil Hospital Information System 1997. Rio de Janeiro, Brazil: Ministry of Health (Brazil).	Country	1993-1997	Administrative record
Maternal sepsis and other maternal infections	Ministry of Health (Brazil). Brazil Hospital Information System 2008-2012.		2008-2012	Administrative record
Maternal sepsis and other maternal infections	Institute for Health Metrics and Evaluation (IHME). IHME GBD DisMod Maternal Live Birth Adjusted Estimates.	Global	1990-2016	Modeled data
Maternal sepsis and other maternal infections	Madeiro AP, Rufino AC, Lacerda EZ, Brasil LG. Incidence and determinants of severe maternal morbidity: a transversal study in a referral hospital in Teresina, Piaui, Brazil. . 2015; 15: 210.		2012-2013	Scientific literature
Maternal sepsis and other maternal infections	Morse ML, Fonseca SC, Gottgtroy CL, Waldmann CS, Gueller E. Severe maternal morbidity and near misses in a regional reference hospital. . 2011; 14(2): 310-22.		2009	Scientific literature
Maternal sepsis and other maternal infections	Health Institute (São Paulo, Brazil), State University of Campinas, São Paulo Municipal Health Department, São Paulo State University, University of São Paulo. Brazil - São Paulo Health Survey 2008-2009.	São Paulo	2002-2008	Survey
Maternal sepsis and other maternal infections	Amaral E, Souza JP, Surita F, Luz AG, Sousa MH, Cecatti JG, Campbell O. A population-based surveillance study on severe acute maternal morbidity (near-miss) and adverse perinatal outcomes in Campinas, Brazil: the Vigimoma Project. . 2011; 11: 9.		2005	Scientific literature
Maternal sepsis and other maternal infections	Ministry of Health (Brazil). Brazil Hospital Information System 1998-2002.		1998-2002	Administrative record
Maternal sepsis and other maternal infections	Ministry of Health (Brazil). Brazil Hospital Information System 2003-2007.		2003-2007	Administrative record
Maternal sepsis and other maternal infections	Brazilian Institute of Geography and Statistics (IBGE), Ministry of Health (Brazil), Ministry of Planning, Budget, and Management (Brazil). Brazil National Health Survey 2013. Rio de Janeiro, Brazil: Brazilian Institute of Geography and Statistics (IBGE).	Country	2013-2014	Survey
Maternal sepsis and other maternal infections	Brazil World Health Survey 2003	Country	2002-2003	Survey
Maternal sepsis and other maternal infections	Ministry of Health (Brazil). Brazil Hospital Information System 2013-2014.		2013-2017	Administrative record
Maternal hypertensive disorders	Amaral E, Souza JP, Surita F, Luz AG, Sousa MH, Cecatti JG, Campbell O. A population-based surveillance study on severe acute maternal morbidity (near-miss) and adverse perinatal outcomes in Campinas, Brazil: the Vigimoma Project. . 2011; 11: 9.		2005	Scientific literature
Maternal hypertensive disorders	Ministry of Health (Brazil). Brazil Hospital Information System 2003-2007.		2003-2007	Administrative record
Maternal hypertensive disorders	Ministry of Health (Brazil). Brazil Hospital Information System 2013-2014.		2013-2017	Administrative record
Maternal hypertensive disorders	Brazilian Institute of Geography and Statistics (IBGE), Ministry of Health (Brazil), Ministry of Planning, Budget, and Management (Brazil). Brazil National Health Survey 2013. Rio de Janeiro, Brazil: Brazilian Institute of Geography and Statistics (IBGE).	Country	2013-2014	Survey
Maternal hypertensive disorders	Galvão LPL, Alvim-Pereira F, de Mendonça CMM, Menezes FEF, Góis KA do N, Ribeiro RF, Gurgel RQ. The prevalence of severe maternal morbidity and near miss and associated factors in Sergipe, Northeast Brazil. . 2014; 25.		2011-2012	Scientific literature
Maternal hypertensive disorders	Ministry of Health (Brazil). Brazil Hospital Information System 2008-2012.		2008-2012	Administrative record
Maternal hypertensive disorders	Morse ML, Fonseca SC, Gottgtroy CL, Waldmann CS, Gueller E. Severe maternal morbidity and near misses in a regional reference hospital. . 2011; 14(2): 310-22.		2009	Scientific literature
Maternal hypertensive disorders	Health Institute (São Paulo, Brazil), State University of Campinas, São Paulo Municipal Health Department, São Paulo State University, University of São Paulo. Brazil - São Paulo Health Survey 2008-2009.	São Paulo	2002-2008	Survey

Maternal hypertensive disorders	Madeiro AP, Rufino AC, Lacerda EZ, Brasil LG. Incidence and determinants of severe maternal morbidity: a transversal study in a referral hospital in Teresina, Piaui, Brazil. . 2015; 15: 210.		2012-2013	Scientific literature
Maternal hypertensive disorders	Giordano JC, Parpinelli MA, Cecatti JG, Haddad SM, Costa ML, Surita FG, Pinto E Silva JL, Sousa MH. The burden of eclampsia: results from a multicenter study on surveillance of severe maternal morbidity in Brazil. . 2014; 9(5): e97401.		2009-2010	Scientific literature
Maternal hypertensive disorders	Institute for Health Metrics and Evaluation (IHME). IHME GBD DisMod Maternal Live Birth Adjusted Estimates.	Global	1990-2016	Modeled data
Maternal hypertensive disorders	Souza JP, Cecatti JG, Faundes A, Morais SS, Villar J, Carroli G, Gulmezoglu M, Wojdyla D, Zavaleta N, Donner A, Velazco A, Bataglia V, Valladares E, Kublickas M, Acosta A. Maternal near miss and maternal death in the World Health Organization's 2005 global survey on maternal and perinatal health. . 2010; 88(2): 113-9.		2005	Scientific literature
Maternal hypertensive disorders	Ministry of Health (Brazil). Brazil Hospital Information System 1997. Rio de Janeiro, Brazil: Ministry of Health (Brazil).	Country	1993-1997	Administrative record
Maternal hypertensive disorders	Zanette E, Parpinelli MA, Surita FG, Costa ML, Haddad SM, Sousa MH, E Silva JLP, Souza JP, Cecatti JG, Brazilian Network for Surveillance of Severe Maternal Morbidity Group. Maternal near miss and death among women with severe hypertensive disorders: a Brazilian multicenter surveillance study. . 2014; 11(1): 4.		2009-2010	Scientific literature
Maternal hypertensive disorders	Vogel JP, Lee ACC, Souza JP. Maternal morbidity and preterm birth in 22 low- and middle-income countries: a secondary analysis of the WHO Global Survey dataset. . 2014; 56.		2004-2005	Scientific literature
Maternal hypertensive disorders	Brazil World Health Survey 2003	Country	2002-2003	Survey
Maternal hypertensive disorders	Ministry of Health (Brazil). Brazil Hospital Information System 1998-2002.		1998-2002	Administrative record
Maternal abortion, miscarriage, and ectopic pregnancy	Ministry of Health (Brazil). Brazil Hospital Information System 1997. Rio de Janeiro, Brazil: Ministry of Health (Brazil).	Country	1993-1997	Administrative record
Maternal abortion, miscarriage, and ectopic pregnancy	Health Institute (São Paulo, Brazil), State University of Campinas, São Paulo Municipal Health Department, São Paulo State University, University of São Paulo. Brazil - São Paulo Health Survey 2008-2009.	São Paulo	2002-2008	Survey
Maternal abortion, miscarriage, and ectopic pregnancy	Madeiro AP, Rufino AC, Lacerda EZ, Brasil LG. Incidence and determinants of severe maternal morbidity: a transversal study in a referral hospital in Teresina, Piaui, Brazil. . 2015; 15: 210.		2012-2013	Scientific literature
Maternal abortion, miscarriage, and ectopic	Ministry of Health (Brazil). Brazil Hospital Information System 1998-2002.		1998-2002	Administrative record
Maternal abortion, miscarriage, and ectopic	Brazil World Health Survey 2003	Country	2002-2003	Survey
Maternal abortion, miscarriage, and ectopic pregnancy	Brazilian Institute of Geography and Statistics (IBGE), Ministry of Health (Brazil), Ministry of Planning, Budget, and Management (Brazil). Brazil National Health Survey 2013. Rio de Janeiro, Brazil: Brazilian Institute of Geography and Statistics (IBGE).	Country	2013-2014	Survey
Maternal abortion, miscarriage, and ectopic	Ministry of Health (Brazil). Brazil Hospital Information System 2013-2014.		2013-2017	Administrative record
Maternal abortion, miscarriage, and ectopic	Ministry of Health (Brazil). Brazil Hospital Information System 2003-2007.		2003-2007	Administrative record
Maternal abortion, miscarriage, and ectopic pregnancy	Santos TF, Andreoni S, de Souza e Silva R. Prevalence and characteristics of women with induced abortion--Favela México 70, São Vicente-São Paulo. . 2012; 15(1): 123-33.		2008	Scientific literature
Maternal abortion, miscarriage, and ectopic	Ministry of Health (Brazil). Brazil Hospital Information System 2008-2012.		2008-2012	Administrative record
Neonatal preterm birth complications	Brazilian Institute of Geography and Statistics (IBGE), Ministry of Health (Brazil), Ministry of Planning, Budget, and Management (Brazil). Brazil National Health Survey 2013. Rio de Janeiro, Brazil: Brazilian Institute of Geography and Statistics (IBGE).	Country	2013-2014	Survey
Neonatal preterm birth complications	Health Institute (São Paulo, Brazil), State University of Campinas, São Paulo Municipal Health Department, São Paulo State University, University of São Paulo. Brazil - São Paulo Health Survey 2008-2009.	São Paulo	2002-2008	Survey
Neonatal preterm birth complications	Ministry of Health (Brazil). Brazil Hospital Information System 2003-2007.		2003-2007	Administrative record
Neonatal preterm birth complications	Ministry of Health (Brazil). Brazil DATASUS TABNET Vital Statistics Live Births 1996. Rio de Janeiro, Brazil: Ministry of Health (Brazil).	Country	1996	Vital registration
Neonatal preterm birth complications	Ministry of Health (Brazil). Brazil Hospital Information System 2008-2012.		2008-2012	Administrative record
Neonatal preterm birth complications	Ministry of Health (Brazil). Brazil Hospital Information System 1997. Rio de Janeiro, Brazil: Ministry of Health (Brazil).	Country	1993-1997	Administrative record
Neonatal preterm birth complications	Brazil World Health Survey 2003	Country	2002-2003	Survey
Neonatal preterm birth complications	Ministry of Health (Brazil). Brazil Hospital Information System 2013-2014.		2013-2017	Administrative record
Neonatal preterm birth complications	Ministry of Health (Brazil). Brazil Hospital Information System 1998-2002.		1998-2002	Administrative record
Neonatal preterm birth complications	Straughn HK, Goldenberg RL, Tolosa JE, Daly S, de Codes J, Festin MR, Limpongsanurak S, Lumbiganon P, Paul VK, Peedicayil A, Purwar M, Sabogal JC, Shenoy S. Birthweight-specific neonatal mortality in developing countries and obstetric practices. . 2003; 80(1): 71-8.		2003	Scientific literature
Neonatal preterm birth complications	Ministry of Health (Brazil). Brazil DATASUS TABNET Vital Statistics Live Births 2000. Rio de Janeiro, Brazil: Ministry of Health (Brazil).	Country	2000	Vital registration
Neonatal preterm birth complications	Ministry of Health (Brazil). Brazil DATASUS TABNET Vital Statistics Live Births 1997. Rio de Janeiro, Brazil: Ministry of Health (Brazil).	Country	1997	Vital registration
Neonatal preterm birth complications	Ministry of Health (Brazil). Brazil DATASUS TABNET Vital Statistics Live Births 2004. Rio de Janeiro, Brazil: Ministry of Health (Brazil).	Country	2004	Vital registration
Neonatal preterm birth complications	Neonatal Units in Rio de Janeiro: Screening Criteria and Workload Implications. . 2010; 126(2): e410-e417.		2005	Scientific literature
Neonatal preterm birth complications	Ministry of Health (Brazil). Brazil DATASUS TABNET Vital Statistics Live Births 2012. Rio de Janeiro, Brazil: Ministry of Health (Brazil).	Country	2012	Vital registration
Neonatal preterm birth complications	Ministry of Health (Brazil). Brazil DATASUS TABNET Vital Statistics Live Births 2002. Rio de Janeiro, Brazil: Ministry of Health (Brazil).	Country	2002	Vital registration
Neonatal preterm birth complications	Filho JBF, Eckert GU, Procianoy L, Barros CK, Procianoy RS. Incidence and risk factors for retinopathy of prematurity in very low and in extremely low birth weight infants in a unit-based approach in southern Brazil. . 2007; 23(1): 25-30.		2004	Scientific literature
Neonatal preterm birth complications	Ministry of Health (Brazil). Brazil DATASUS TABNET Vital Statistics Live Births 2005. Rio de Janeiro, Brazil: Ministry of Health (Brazil).	Country	2005	Vital registration
Neonatal preterm birth complications	Ministry of Health (Brazil). Brazil DATASUS TABNET Vital Statistics Live Births 2006. Rio de Janeiro, Brazil: Ministry of Health (Brazil).	Country	2006	Vital registration
Neonatal preterm birth complications	Ministry of Health (Brazil). Brazil DATASUS TABNET Vital Statistics Live Births 2007. Rio de Janeiro, Brazil: Ministry of Health (Brazil).	Country	2007	Vital registration
Neonatal preterm birth complications	Graziano RM, Leone CR, Cunha SL, Pinheiro AC. [Prevalence of retinopathy of prematurity in very low birth weight infants]. . 1996; 73(6): 377-82.		1993	Scientific literature
Neonatal preterm birth complications	Ministry of Health (Brazil). Brazil DATASUS TABNET Vital Statistics Live Births 2001. Rio de Janeiro, Brazil: Ministry of Health (Brazil).	Country	2001	Vital registration
Neonatal preterm birth complications	Ministry of Health (Brazil). Brazil DATASUS TABNET Vital Statistics Live Births 2008. Rio de Janeiro, Brazil: Ministry of Health (Brazil).	Country	2008	Vital registration
Neonatal preterm birth complications	Ministry of Health (Brazil). Brazil DATASUS TABNET Vital Statistics Live Births 1998. Rio de Janeiro, Brazil: Ministry of Health (Brazil).	Country	1998	Vital registration
Neonatal preterm birth complications	Ministry of Health (Brazil). Brazil DATASUS TABNET Vital Statistics Live Births 1999. Rio de Janeiro, Brazil: Ministry of Health (Brazil).	Country	1999	Vital registration
Neonatal preterm birth complications	Ministry of Health (Brazil). Brazil DATASUS TABNET Vital Statistics Live Births 2009. Rio de Janeiro, Brazil: Ministry of Health (Brazil).	Country	2009	Vital registration
Neonatal preterm birth complications	Ministry of Health (Brazil). Brazil DATASUS TABNET Vital Statistics Live Births 2010. Rio de Janeiro, Brazil: Ministry of Health (Brazil).	Country	2010	Vital registration
Neonatal preterm birth complications	Ministry of Health (Brazil). Brazil DATASUS TABNET Vital Statistics Live Births 2011. Rio de Janeiro, Brazil: Ministry of Health (Brazil).	Country	2011	Vital registration
Neonatal preterm birth complications	Ministry of Health (Brazil). Brazil DATASUS TABNET Vital Statistics Live Births 2003. Rio de Janeiro, Brazil: Ministry of Health (Brazil).	Country	2003	Vital registration
Neonatal encephalopathy due to birth asphyxia and trauma	Brazilian Institute of Geography and Statistics (IBGE), Ministry of Health (Brazil), Ministry of Planning, Budget, and Management (Brazil). Brazil National Health Survey 2013. Rio de Janeiro, Brazil: Brazilian Institute of Geography and Statistics (IBGE).	Country	2013-2014	Survey
Neonatal encephalopathy due to birth asphyxia	Brazil World Health Survey 2003	Country	2002-2003	Survey
Neonatal encephalopathy due to birth asphyxia and trauma	Da Silva LFG, Höefel Filho JR, Anés M, Nunes ML. Prognostic value of 1H-MRS in neonatal encephalopathy. . 2006; 34(5): 360-6.		2003-2004	Scientific literature
Neonatal encephalopathy due to birth asphyxia and trauma	Health Institute (São Paulo, Brazil), State University of Campinas, São Paulo Municipal Health Department, São Paulo State University, University of São Paulo. Brazil - São Paulo Health Survey 2008-2009.	São Paulo	2002-2008	Survey
Neonatal sepsis and other neonatal infections	Brazilian Institute of Geography and Statistics (IBGE), Ministry of Health (Brazil), Ministry of Planning, Budget, and Management (Brazil). Brazil National Health Survey 2013. Rio de Janeiro, Brazil: Brazilian Institute of Geography and Statistics (IBGE).	Country	2013-2014	Survey
Neonatal sepsis and other neonatal infections	Ministry of Health (Brazil). Brazil Hospital Information System 1998-2002.		1998-2002	Administrative record
Neonatal sepsis and other neonatal infections	Ministry of Health (Brazil). Brazil Hospital Information System 1997. Rio de Janeiro, Brazil: Ministry of Health (Brazil).	Country	1993-1997	Administrative record
Neonatal sepsis and other neonatal infections	Ministry of Health (Brazil). Brazil Hospital Information System 2003-2007.		2003-2007	Administrative record
Neonatal sepsis and other neonatal infections	Ministry of Health (Brazil). Brazil Hospital Information System 2013-2014.		2013-2017	Administrative record
Neonatal sepsis and other neonatal infections	Brazil World Health Survey 2003	Country	2002-2003	Survey
Neonatal sepsis and other neonatal infections	Health Institute (São Paulo, Brazil), State University of Campinas, São Paulo Municipal Health Department, São Paulo State University, University of São Paulo. Brazil - São Paulo Health Survey 2008-2009.	São Paulo	2002-2008	Survey
Neonatal sepsis and other neonatal infections	Ministry of Health (Brazil). Brazil Hospital Information System 2008-2012.		2008-2012	Administrative record

Hemolytic disease and other neonatal jaundice	Institute for Health Metrics and Evaluation (IHME). IHME GBD DisMod Neonatal Hemolytic Disease Excess Mortality Estimates.	Global	1990-2016	Modeled data
Hemolytic disease and other neonatal jaundice	Bhutani VK, Zipursky A, Blencowe H, Khanna R, Sgro M, Ebbesen F, Bell J, Mori R, Slusher TM, Fahmy N, Paul VK, Du L, Okolo AA, de Almeida MF, Olusanya BO, Kumar P, Cousens S, Lawn JE. Neonatal hyperbilirubinemia and Rhesus disease of the newborn: incidence and impairment estimates for 2010 at regional and global levels. . 2013; 74(Suppl 1): 86-100.		2010	Scientific literature
Hemolytic disease and other neonatal jaundice	Institute for Health Metrics and Evaluation (IHME). IHME GBD 2013 DisMod model input - Neonatal Conditions.	Global	1990-2016	Modeled data
Hemolytic disease and other neonatal jaundice	Brazilian Society for Family Welfare (BEMFAM), Macro International, Inc. Brazil Demographic and Health Survey - Complete Birth History Data.		1982-1997	Survey
Vitamin A deficiency	[Epidemiologic Survey of xerophthalmia in the State of Paraiba] as it appears in		1981-1983	Scientific literature
Vitamin A deficiency	Clinical, Subclinical, and Epidemiological Aspects of Vitamin A Deficiency in the State of Paraíba as it appears in	Paraíba	1992	Report
Vitamin A deficiency	Brazil - Pernambuco Second State Survey of Health and Nutrition 1997 as it appears in	Pernambuco	1997	Survey
Vitamin A deficiency	Martins MC, Santos LMP, Assis AMO. [Prevalence of hypovitaminosis A among preschool children from northeastern Brazil, 1998]. . 2004; 38(4): 537-42.		1998	Scientific literature
Vitamin A deficiency	Azevedo MMS de, Cabral PC, Diniz A da S, Fisberg M, Fisberg RM, Arruda IKG de. [Vitamin A deficiency in preschool children of Recife, Northeast of Brazil]. . 2010; 60(1): 36-41.		2007	Scientific literature
Iron-deficiency anemia	Fogarty International Center, National Institutes of Health (NIH), Foundation for the National Institutes of Health (FNIH), National Institute of Science, Technology, and Biomedicine of Semi-Arid Brazil (INCT-IBISAB). Brazil - Fortaleza Malnutrition and Enteric Disease Study 2009-2014.	Ceará	2010-2013	Survey
Iron-deficiency anemia	Zago MA, Costa FF. Hereditary haemoglobin disorders in Brazil. . 1985; 79(3): 385-8.		1983-1985	Scientific literature
Iron-deficiency anemia	Brazilian Center for Analysis and Planning (CEBRAP), Brazilian Institute of Public Opinion and Statistics (IBOPE), Ministry of Health (Brazil). Brazil National Demographic and Health Survey of Children and Women 2006-2007. Rio de Janeiro, Brazil: Ministry of Health (Brazil).	Country	2006-2007	Survey
Iron-deficiency anemia	Ministry of Health (Brazil). Brazil Hospital Information System 2008-2012.		2008-2012	Administrative record
Iron-deficiency anemia	King CH, Dickman K, Tisch DJ. Reassessment of the cost of chronic helmintic infection: a meta-analysis of disability-related outcomes in endemic schistosomiasis. . 2005; 365(9470): 15619.		1966-1978	Scientific literature
Iron-deficiency anemia	Assis AMO, Gaudenzi EN, Gomes G, Ribeiro R de C, Szarfarc SC, Souza SB de. [Hemoglobin concentration, breastfeeding and complementary feeding in the first year of life]. . 2004; 38(4): 543-51.		1998-1999	Scientific literature
Iron-deficiency anemia	Adolescents: Health, Education, and Work 1991. Teresina, Brazil: Piaui State Government, 1992.	Piaui	1991	Report
Iron-deficiency anemia	Rondo PH, Abbott R, Rodrigues LC, Tomkins AM. Vitamin A, folate, and iron concentrations in cord and maternal blood of intra-uterine growth retarded and appropriate birth weight babies. . 1995; 49(6): 391-9.		1991-1992	Scientific literature
Iron-deficiency anemia	Osório MM, Lira PI, Batista-Filho M, Ashworth A. Prevalence of anemia in children 6-59 months old in the state of Pernambuco, Brazil. . 2001; 10(2): 101-7.		2006	Scientific literature
Iron-deficiency anemia	Muniz-Junqueira MI, Queiroz EFO. Relationship between protein-energy malnutrition, vitamin A, and parasitoses in living in Brasília. . 2002; 35(2): 133-41.		1983	Scientific literature
Iron-deficiency anemia	Brazil - School Lunch: History, Evolution and Contribution in Addressing the Nutritional Needs of the Child		1996	Report
Iron-deficiency anemia	Ministry of Health (Brazil). Brazil Hospital Information System 2013-2014.		2013-2017	Administrative record
Iron-deficiency anemia	Iron Deficiency and Iron Deficiency Anemia in the Population of 6 Months to 6 Years in Vitória, Espírito Santo, Southeastern Brazil	Espírito Santo	2001-2003	Report
Iron-deficiency anemia	Ministry of Health (Brazil). Brazil Hospital Information System 1997. Rio de Janeiro, Brazil: Ministry of Health (Brazil).	Country	1993-1997	Administrative record
Iron-deficiency anemia	Ministry of Health (Brazil). Brazil Hospital Information System 2003-2007.		2003-2007	Administrative record
Iron-deficiency anemia	Ministry of Health (Brazil). Brazil Hospital Information System 1998-2002.		1998-2002	Administrative record
Syphilis	Ministry of Health (Brazil). Brazil Hospital Information System 2013-2014.		2013-2017	Administrative record
Syphilis	Ministry of Health (Brazil). Brazil Hospital Information System 2008-2012.		2008-2012	Administrative record
Syphilis	Joint United Nations Program on HIV/AIDS (UNAIDS), United Nations Children's Fund (UNICEF), World Health Organization (WHO). Brazil Global AIDS Response Progress Reporting (GARPR) System - Antenatal Care Attendees Positive for Syphilis.	Country	2010-2012	Epi surveillance
Syphilis	Rodrigues CS, Guimaraes MDC, Grupo Nacional de Estudo sobre Sifilis Congenita. [Syphilis positivity in puerperal women: still a challenge in Brazil]. . 2004; 16(3): 16875.		1999-2000	Scientific literature
Syphilis	Nobrega I, Dantas P, Rocha P, Rios I, Abraao M, Netto EM, Brites C. Syphilis and HIV-1 among parturient women in Salvador, Brazil: low prevalence of syphilis and high rate of loss to follow-up in HIV-infected women. . 2013; 17(2): 18493.		2008-2009	Scientific literature
Syphilis	Ribeiro D, Rezende EF, Pinto VM, Pereira GFM, Miranda AE. Prevalence of and risk factors for syphilis in Brazilian armed forces conscripts. . 2012; 88(1): 324.		2007	Scientific literature
Syphilis	Szwarcwald CL, de Carvalho MF, Barbosa Junior A, Barreira D, Speranza FAB, de Castilho EA. Temporal trends of HIV-related risk behavior among Brazilian military conscripts, 1997-2002. . 2005; 60(5): 36774.		1997-2002	Scientific literature
Syphilis	Amaral E, Faundes A, Gonçales NS, Pellegrino Junior J, de Souza CA, Pinto e Silva JL. Prevalence of HIV and Treponema pallidum infections in pregnant women in Campinas and their association with socio-demographic factors. . 1996; 114(2): 110816.	São Paulo	1991	Scientific literature
Syphilis	Baião AM, Kupek E, Petry A. Syphilis seroprevalence estimates of Santa Catarina blood donors in 2010. . 2014; 47(2): 17985.		2010	Scientific literature
Syphilis	Boa-Sorte N, Purificação A, Amorim T, Assunção L, Reis A, Galvão-Castro B. Dried blood spot testing for the antenatal screening of HTLV, HIV, syphilis, toxoplasmosis and hepatitis B and C: prevalence, accuracy and operational aspects. . 2014; 18(6): 61824.		2009-2010	Scientific literature
Syphilis	Miranda AE, Figueiredo NC, Schmidt R, Page-Shafer K. A population-based survey of the prevalence of HIV, syphilis, hepatitis B and hepatitis C infections, and associated risk factors among young women in Vitoria, Brazil. . 2008; 12(4 Suppl): S25-31.		2006	Scientific literature
Syphilis	Benzaken AS, Sabido M, Galban E, Pedroza V, Araujo AJ, Peeling RW, Mabey D. Field performance of a rapid point-of-care diagnostic test for antenatal syphilis screening in the Amazon region, Brazil. . 2011; 22(1): 15-8.		2006	Scientific literature
Syphilis	Ministry of Health (Brazil). Brazil Hospital Information System 2003-2007.		2003-2007	Administrative record
Syphilis	De Souza NCN, Botelho CAO, Honer MR. Retrospective study of a pioneer antenatal screening program with 8,477 pregnant women in Brazil. . 2004; 31(3): 217-20.		2002-2003	Scientific literature
Syphilis	Ministry of Health (Brazil). Brazil Hospital Information System 1998-2002.		1998-2002	Administrative record
Syphilis	Brazil World Health Survey 2003	Country	2002-2003	Survey
Syphilis	Brazilian Institute of Geography and Statistics (IBGE), Ministry of Health (Brazil), Ministry of Planning, Budget, and Management (Brazil). Brazil National Health Survey 2013. Rio de Janeiro, Brazil: Brazilian Institute of Geography and Statistics (IBGE).	Country	2013-2014	Survey
Syphilis	Health Institute (São Paulo, Brazil), State University of Campinas, São Paulo Municipal Health Department, São Paulo State University, University of São Paulo. Brazil - São Paulo Health Survey 2008-2009.	São Paulo	2002-2008	Survey
Syphilis	Ministry of Health (Brazil). Brazil Hospital Information System 1997. Rio de Janeiro, Brazil: Ministry of Health (Brazil).	Country	1993-1997	Administrative record
Syphilis	Madi JM, Souza R da S de, Araújo BF de, Oliveira Filho PF de, Rombaldi RL, Mitchell C, Lorencetti J, Marcon NO. Prevalence of toxoplasmosis, HIV, syphilis and rubella in a population of puerperal women using Whatman 903 filter paper. . 2010; 14(1): 24-9.		2007-2008	Scientific literature
Syphilis	Miranda AE, Figueiredo NC, Pinto VM, Page K, Talhari S. Risk factors for syphilis in young women attending a family health program in Vitória, Brazil. . 2012; 87(1): 76-83.		2010	Scientific literature
Syphilis	Joint United Nations Program on HIV/AIDS (UNAIDS), Ministry of Health (Brazil). Brazil Progress Report on the Response to HIV/AIDS 2012. Geneva, Switzerland: Joint United Nations Program on HIV/AIDS (UNAIDS), 2012.	Country	2012	Report
Chlamydial infection	Institute for Health Metrics and Evaluation (IHME). IHME GBD 2015 DisMod All Causes Excess Mortality Estimates.	Global	1990-2016	Modeled data
Chlamydial infection	Guimarães EMB, Guimarães MDC, Vieira MAS, Bontempo NM, Seixas MSS, Garcia MSD, Daud LES, Côrtes RLM, Alves M de FC. Lack of utility of risk score and gynecological examination for screening for sexually transmitted infections in sexually active adolescents. . 2009; 7: 8.		2002	Scientific literature
Chlamydial infection	De Lima YAR, Turchi MD, Fonseca ZC, Garcia FLB, de Brito e Cardoso FA, da Guarda Reis MN, de Britto Guimarães EM, Alves RRF, Carvalho NR, de Fátima Costa Alves M. Sexually transmitted bacterial infections among young women in Central Western Brazil. . 2014; 1621.		2007-2009	Scientific literature
Chlamydial infection	Rodrigues MM, Fernandes PÁ, Haddad JP, Paiva MC, Souza MDCM, Andrade TCA, Fernandes AP. Frequency of Chlamydia trachomatis, Neisseria gonorrhoeae, Mycoplasma genitalium, Mycoplasma hominis and Ureaplasma species in cervical samples. . 2011; 31(3): 237-41.		2000-2009	Scientific literature
Chlamydial infection	trachomatis prevalence and risk behaviors in parturient women aged 15 to 24 in Brazil. . 2011; 38(10): 957-61.		2009	Scientific literature
Chlamydial infection	Brazil - Seroprevalence Survey of AIDS in Mental Health 2006	Country	2004	Survey
Chlamydial infection	Rocha DAP, Filho RAAB, Mariño JM, dos Santos CMB. Hidden sexually transmitted infections among women in primary care health services, Amazonas, Brazil. . 2014; 25(12): 87886.		2010	Scientific literature

Chlamydial infection	Borborema-Alfaia APB de, Freitas NS de L, Astolfi Filho S, Borborema-Santos CM. Chlamydia trachomatis infection in a sample of northern Brazilian pregnant women: prevalence and prenatal importance. . 2013; 17(5): 54550.		2005	Scientific literature
Gonococcal infection	Rocha DAP, Filho RAAB, Mariño JM, dos Santos CMB. Hidden sexually transmitted infections among women in primary care health services, Amazonas, Brazil. . 2014; 25(12): 87886.		2010	Scientific literature
Gonococcal infection	trachomatis prevalence and risk behaviors in parturient women aged 15 to 24 in Brazil. . 2011; 38(10): 957-61.		2009	Scientific literature
Gonococcal infection	Institute for Health Metrics and Evaluation (IHME). IHME GBD 2015 DisMod All Causes Excess Mortality Estimates.	Global	1990-2016	Modeled data
Gonococcal infection	De Lima YAR, Turchi MD, Fonseca ZC, Garcia FLB, de Brito e Cardoso FA, da Guarda Reis MN, de Britto Guimarães EM, Alves RRF, Carvalho NR, de Fátima Costa Alves M. Sexually transmitted bacterial infections among young women in Central Western Brazil. . 2014; 1621.		2007-2009	Scientific literature
Gonococcal infection	Brazil - Seroprevalence Survey of AIDS in Mental Health 2006	Country	2004	Survey
Gonococcal infection	Guimarães EMB, Guimarães MDC, Vieira MAS, Bontempo NM, Seixas MSS, Garcia MSD, Daud LES, Côrtes RLM, Alves M de FC. Lack of utility of risk score and gynecological examination for screening for sexually transmitted infections in sexually active adolescents. . 2009; 7: 8.		2002	Scientific literature
Trichomoniasis	Miranda AE, Pinto VM, Gaydos CA. Trichomonas vaginalis infection among young pregnant women in Brazil. . 2014; 18(6): 66971.		2009	Scientific literature
Trichomoniasis	Grama DF, Casarotti L da S, Morato MGV de A, Silva LS, Mendonça DF, Limongi JE, Viana J da C, Cury MC. Prevalence of Trichomonas vaginalis and risk factors in women treated at public health units in Brazil: a transversal study. . 2013; 107(9): 58491.		2009-2010	Scientific literature
Trichomoniasis	Rocha DAP, Filho RAAB, Mariño JM, dos Santos CMB. Hidden sexually transmitted infections among women in primary care health services, Amazonas, Brazil. . 2014; 25(12): 87886.		2010	Scientific literature
Trichomoniasis	Mascarenhas REM, Machado MSC, Costa e Silva BFB da, Pimentel RFW, Ferreira TT, Leoni FMS, Grassi MFR. Prevalence and risk factors for bacterial vaginosis and other vulvovaginitis in a population of sexually active adolescents from Salvador, Bahia, Brazil. . 2012; 378640.		2008-2010	Scientific literature
Genital herpes	Smith JS, Herrero R, Bosetti C, Muñoz N, Bosch FX, Eluf-Neto J, Castellsagué X, Meijer CJLM, Van den Brule AJC, Franceschi S, Ashley R, International Agency for Research on Cancer (IARC) Multicentric Cervical Cancer Study Group. Herpes simplex virus-2 as a human papillomavirus cofactor in the etiology of invasive cervical cancer. . 2002; 94(21): 160413.		1999-2001	Scientific literature
Genital herpes	Nascimento MC, Sumita LM, Souza VU, Weiss HA, Oliveira J, Mascheretti M, Quiroga M, Vela RAR, Sabino E, Pannuti CS, Mayaud P. Seroprevalence of Kaposi sarcoma-associated herpesvirus and other serologic markers in the Brazilian Amazon. . 2009; 15(4): 6637.		2003-2004	Scientific literature
Genital herpes	Alberts CJ, Schim van der Loeff MF, Papenfuss MR, da Silva RJC, Villa LL, Lazcano-Ponce E, Nyitray AG, Giuliano AR. Association of Chlamydia trachomatis infection and herpes simplex virus type 2 serostatus with genital human papillomavirus infection in men: the HPV in men study. . 2013; 40(6): 50815.		2005-2009	Scientific literature
Genital herpes	Benzaken A, Sabidó M, Galban E, Rodrigues Dutra DL, Leturiondo AL, Mayaud P. HIV and sexually transmitted infections at the borderlands: situational analysis of sexual health in the Brazilian Amazon. . 2012; 88(4): 294300.		2009	Scientific literature
Genital herpes	Lupi O. Prevalence and risk factors for herpes simplex infection among patients at high risk for HIV infection in Brazil. . 2011; 50(6): 70913.		1996-1997	Scientific literature
Genital herpes	Clemens SAC, Farhat CK. Seroprevalence of herpes simplex 1-2 antibodies in Brazil. . 2010; 44(4): 72634.		1996-1997	Scientific literature
Genital herpes	immunodeficiency virus test-seeking motivation in blood donors, São Paulo, Brazil. . 2006; 90(3): 170-6.		2004	Scientific literature
Genital herpes	WS, Tateno A, Boulos M, Mayaud P, Pannuti CS. Human herpesvirus-8 infection and oral shedding in Amerindian and non-Amerindian populations in the Brazilian Amazon region. . 2007; 196(6): 844-52.		2003-2004	Scientific literature
Genital herpes	2 infection in pregnancy: asymptomatic viral excretion at delivery and seroepidemiologic survey of two socioeconomically distinct populations in São Paulo, Brazil. . 1993; 35(3): 285-90.		1988-1989	Scientific literature
Genital herpes	Smith JS, Herrero R, Muñoz N, Eluf-Neto J, Ngelangel C, Bosch FX, Ashley RL. Prevalence and risk factors for herpes simplex virus type 2 infection among middle-age women in Brazil and the Philippines. . 2001; 28(4): 187-94.		1990-1991	Scientific literature
Genital herpes	Cowan FM, French RS, Mayaud P, Gopal R, Robinson NJ, de Oliveira SA, Faillace T, Uusküla A, Nygård-Kibur M, Ramalingam S, Sridharan G, El Aouad R, Alami K, Rbai M, Sunil-Chandra NP, Brown DW. Seroepidemiological study of herpes simplex virus types 1 and 2 in Brazil, Estonia, India, Morocco, and Sri Lanka. . 2003; 79(4): 286-90.		2000	Scientific literature
Other sexually transmitted diseases	Institute for Health Metrics and Evaluation (IHME). IHME GBD 2015 DisMod All Causes Excess Mortality Estimates.	Global	1990-2016	Modeled data
Acute hepatitis A	LP, Amado LA, Engstrom EM, Fortes CDFM, Souza TC de, Dias MN, Gaspar AMC, Souto FJD. Declining prevalence of hepatitis A virus antibodies among children from low socioeconomic groups reinforces the need for the implementation of hepatitis A vaccination in Brazil. . 2012; 107(5): 6528.		2007-2009	Scientific literature
Acute hepatitis A	Vitral CL, da Silva-Nunes M, Pinto MA, de Oliveira JM, Gaspar AMC, Pereira RCC, Ferreira MU. Hepatitis A and E seroprevalence and associated risk factors: a community-based cross-sectional survey in rural Amazonia. . 2014; 14: 458.		2004	Scientific literature
Acute hepatitis A	Mantovani SAS, Delfino BM, Martins AC, Oliart-Guzmán H, Pereira TM, Branco FLCC, Braña AM, Filgueira-Júnior JA, Santos AP, Arruda RA, Guimarães AS, Ramalho AA, Oliveira CS de M, Araújo TS, Arróspide N, Estrada CHML, Codeço CT, da Silva-Nunes M. Socioeconomic inequities and hepatitis A virus infection in Western Brazilian Amazonian children: spatial distribution and associated factors. . 2015; 15: 428.		2011	Scientific literature
Acute hepatitis A	Pinheiro RS, Araújo LA de, Caetano KAA, Matos MA de, Carneiro MADS, Teles SA. INTERMEDIATE ENDEMICITY OF HEPATITIS A VIRUS INFECTION IN RURAL SETTLEMENT PROJECTS OF SOUTHWEST GOIÁS, BRAZIL. . 2015; 52(3): 2003.		2011	Scientific literature
Acute hepatitis A	Pereira TM, Mantovani SAS, Branco FLCC, Braña AM, Oliart-Guzmán H, Delfino BM, Martins AC, Araújo TS, Oliveira CSM, Muniz PT, da Silva-Nunes M. Hepatitis A seroprevalence in preschool children in Assis Brazil, Acre, Brazil, in 2003 and 2010. . 2015.		2003-2010	Scientific literature
Acute hepatitis A	Moreira ED Jr, Nassri VB, Santos RS, Matos JF, de Carvalho WA, Silvani CS, Santana e Sant'ana C. Association of Helicobacter pylori infection and giardiasis: results from a study of surrogate markers for fecal exposure among children. . 2005; 11(18): 2759-63.		2001-2002	Scientific literature
Acute hepatitis A	Black FL, Jacobson DL. Hepatitis A antibody in an isolated Amerindian tribe fifty years after exposure. . 1986; 19(1): 19-21.		1983-1984	Scientific literature
Acute hepatitis A	Gonçalves AAS, Oliveira LCM de. Seroprevalence of hepatitis A immunity among children and adolescents in two cities of the Triângulo Mineiro region, state of Minas Gerais, Brazil. . 2012; 16(5): 496-7.		2010-2011	Scientific literature
Acute hepatitis A	Gomes MAC, Ferreira A de SP, da Silva AAM, de Souza ER. Hepatitis A: seroprevalence and associated factors among schoolchildren of São Luís (MA), Brazil. . 2011; 14(4): 548-55.		2002-2004	Scientific literature
Acute hepatitis A	Markus JR, Cruz CR, Maluf EMCP, Tahan TT, Hoffmann MM. Seroprevalence of hepatitis A in children and adolescents. . 2011; 87(5): 419-24.		2006	Scientific literature
Acute hepatitis A	De Alencar Ximenes RA, Martelli CMT, Merchán-Hamann E, Montarroyos UR, Braga MC, de Lima MLC, Cardoso MRA, Turchi MD, Costa MA, de Alencar LCA, Moreira RC, Figueiredo GM, Pereira LMMB. Multilevel analysis of hepatitis A infection in children and adolescents: a household survey in the Northeast and Central-west regions of Brazil. . 2008; 37(4): 852-61.		2004-2005	Scientific literature
Hepatitis B	Dos Santos JI, Lopes MA, Deliège-Vasconcelos E, Couto-Fernandez JC, Patel BN, Barreto ML, Ferreira Júnior OC, Galvão-Castro B. Seroprevalence of HIV, HTLV-I/II and other perinatally-transmitted pathogens in Salvador, Bahia. . 1995; 37(4): 343-8.		1990-1991	Scientific literature
Hepatitis B	Coimbra Júnior CE, Santos RV, Yoshida CF, Baptista ML, Flowers NM, do Valle AC. Hepatitis B epidemiology and cultural practices in Amerindian populations of Amazonia: the Tupí-Mondé and the Xavánte from Brazil. . 1996; 42(12): 1735-43.		1990	Scientific literature
Hepatitis B	serological markers of hepatitis B virus in pregnant women from Paraná State, Brazil. . 2006; 39(8): 1083-90.		1998-2002	Scientific literature
Hepatitis B	Aquino JA, Pegado KA, Barros LP, Machado LFA. Soroprevalência de infecções por vírus da hepatite B e vírus da hepatite C em indivíduos do Estado do Pará. . 2008; 334-7.		2002-2005	Scientific literature
Hepatitis B	A, Spada C, Treitinger A. Hepatitis B marker seroprevalence and vaccination coverage in adolescents in the City of Itajaí, State of Santa Catarina, Southern Brazil, in 2008. . 2011; 44(4): 416-9.		2008	Scientific literature
Hepatitis B	Seroprevalence of hepatitis B and hepatitis C markers in adolescents in Southern Brazil. . 2011; 27(4): 753-8.		2008	Scientific literature
Hepatitis B	De Souza NCN, Botelho CAO, Honer MR. Retrospective study of a pioneer antenatal screening program with 8,477 pregnant women in Brazil. . 2004; 31(3): 217-20.		2002-2003	Scientific literature
Hepatitis B	Braga WSM, Castilho M da C, Borges FG, Martinho AC de S, Rodrigues IS, Azevedo EP de, Scazufca M, Menezes PR. Prevalence of hepatitis B virus infection and carriage after nineteen years of vaccination program in the Western Brazilian Amazon. . 2012; 45(1): 13-7.		2005-2007	Scientific literature

Hepatitis C	Pereira LMMB, Martelli CMT, Moreira RC, Merchan-Hamman E, Stein AT, Cardoso MRA, Figueiredo GM, Montarroyos UR, Braga C, Turchi MD, Coral G, Crespo D, Lima MLC, Alencar LCA, Costa M, dos Santos AA, Ximenes RAA. Prevalence and risk factors of Hepatitis C virus infection in Brazil, 2005 through 2009: a cross-sectional study. . 2013; 60.		2005-2009	Scientific literature
Hepatitis C	Carvalho MB, Hamerschlak N, Vaz RS, Ferreira OC Jr. Risk factor analysis and serological diagnosis of HIV-1/HIV-2 infection in a Brazilian blood donor population: validation of the World Health Organization strategy for HIV testing. . 1996; 10(10): 1135-40.		1992-1993	Scientific literature
Hepatitis C	Martins RM, Vanderborght BO, Rouzere C, Cardoso DD, Azevedo MS, Yoshida CF. Anti-HCV prevalence and risk factors analysis in pregnant women in central Brazil. . 1995; 90(1): 11.		1990-1992	Scientific literature
Hepatitis C	Institute for Health Metrics and Evaluation (IHME). IHME GBD 2013 DisMod model input - Hepatitis C.	Global	1990-2015	Modeled data
Hepatitis C	Menegol D, Spilki FR. Seroprevalence of Hepatitis B and C markers at the population level in the municipality of Caxias do Sul, southern Brazil. . 2013; 44(4): 1237-40.		2008-2011	Scientific literature
Hepatitis C	Aquino JA, Pegado KA, Barros LP, Machado LFA. Soroprevalência de infecções por vírus da hepatite B e vírus da hepatite C em indivíduos do Estado do Pará. . 2008; 334-7.		2002-2005	Scientific literature
Hepatitis C	De Souza NCN, Botelho CAO, Honer MR. Retrospective study of a pioneer antenatal screening program with 8,477 pregnant women in Brazil. . 2004; 31(3): 217-20.		2002-2003	Scientific literature
Acute hepatitis E	Parana R, Cotrim HP, Cortey-Boennec ML, Trepo C, Lyra L. Prevalence of hepatitis E virus IgG antibodies in patients from a referral unit of liver diseases in Salvador, Bahia, Brazil. . 1997; 57(1): 60-1.	Country	1992-1994	Scientific literature
Colon and rectum cancer	Institute for Health Metrics and Evaluation (IHME). IHME GBD 2015 DisMod Cancer Procedure Incidence Estimates.	Global	1990-2020	Modeled data
Liver cancer due to hepatitis B	Gonçalves PL, Zago-Gomes Mda P, Gonçalves CS, Pereira FE. Hepatitis virus and hepatocellular carcinoma in Brazil: a report from the State of Espírito Santo. . 2014; 47(5): 559-63.		1993-2011	Scientific literature
Liver cancer due to hepatitis B	Gonçalves CS, Pereira FE, Gayotto LC. Hepatocellular carcinoma in Brazil: report of a national survey (Florianópolis, SC, 1995). . 1997; 39(3): 165-70.		1992-1994	Scientific literature
Liver cancer due to hepatitis B	MTF, da Cruz E do RM, Demachki S, Bensabath G, Soares M do CP. [Hepatitis B and C virus infection and the hepatocellular carcinoma in the East Amazon, Brazil]. . 2004; 37(Supp 2): 47-51.		1992-1999	Scientific literature
Liver cancer due to hepatitis B	Osório FMF, Lauar GM, Lima AS, Vidigal PVT, Ferrari TCA, Couto CA. Epidemiological aspects of hepatocellular carcinoma in a referral center of Minas Gerais, Brazil. . 2013; 50(2): 97-100.		1998-2010	Scientific literature
Liver cancer due to hepatitis B	Fontes PR, Schmidt Cerski CT, Hartmann AA, Kretzmann Filho NA. Evaluation of the C3435T polymorphism in the MDR1 gene in patients with hepatocellular carcinoma. . 2012; 11(6): 899-906.		2000-2009	Scientific literature
Liver cancer due to hepatitis B	Clinical and epidemiological aspects of hepatocellular carcinoma in Brazil. . 2010; 65(12): 1285-90.		2004-2009	Scientific literature
Liver cancer due to hepatitis B	Hepatitis G virus infection in patients with hepatocellular carcinoma in Recife, Brazil. . 2007; 37(8): 632-6.	Country	1996-1999	Scientific literature
Liver cancer due to hepatitis B	Silva M, Mattos AA de, Fontes PRO, Waechter FL, Pereira-Lima L. Evaluation of hepatic resection for hepatocellular carcinoma on cirrhotic livers . . 2008; 45(2): 99-105.		1996-2005	Scientific literature
Liver cancer due to hepatitis C	Fontes PR, Schmidt Cerski CT, Hartmann AA, Kretzmann Filho NA. Evaluation of the C3435T polymorphism in the MDR1 gene in patients with hepatocellular carcinoma. . 2012; 11(6): 899-906.		2000-2009	Scientific literature
Liver cancer due to hepatitis C	Gonçalves PL, Zago-Gomes Mda P, Gonçalves CS, Pereira FE. Hepatitis virus and hepatocellular carcinoma in Brazil: a report from the State of Espírito Santo. . 2014; 47(5): 559-63.		1993-2011	Scientific literature
Liver cancer due to hepatitis C	Hepatitis G virus infection in patients with hepatocellular carcinoma in Recife, Brazil. . 2007; 37(8): 632-6.	Country	1996-1999	Scientific literature
Liver cancer due to hepatitis C	Osório FMF, Lauar GM, Lima AS, Vidigal PVT, Ferrari TCA, Couto CA. Epidemiological aspects of hepatocellular carcinoma in a referral center of Minas Gerais, Brazil. . 2013; 50(2): 97-100.		1998-2010	Scientific literature
Liver cancer due to hepatitis C	MTF, da Cruz E do RM, Demachki S, Bensabath G, Soares M do CP. [Hepatitis B and C virus infection and the hepatocellular carcinoma in the East Amazon, Brazil]. . 2004; 37(Supp 2): 47-51.		1992-1999	Scientific literature
Liver cancer due to hepatitis C	Silva M, Mattos AA de, Fontes PRO, Waechter FL, Pereira-Lima L. Evaluation of hepatic resection for hepatocellular carcinoma on cirrhotic livers . . 2008; 45(2): 99-105.		1996-2005	Scientific literature
Liver cancer due to hepatitis C	Clinical and epidemiological aspects of hepatocellular carcinoma in Brazil. . 2010; 65(12): 1285-90.		2004-2009	Scientific literature
Liver cancer due to hepatitis C	Gonçalves CS, Pereira FE, Gayotto LC. Hepatocellular carcinoma in Brazil: report of a national survey (Florianópolis, SC, 1995). . 1997; 39(3): 165-70.		1992-1994	Scientific literature
Liver cancer due to alcohol use	Osório FMF, Lauar GM, Lima AS, Vidigal PVT, Ferrari TCA, Couto CA. Epidemiological aspects of hepatocellular carcinoma in a referral center of Minas Gerais, Brazil. . 2013; 50(2): 97-100.		1998-2010	Scientific literature
Liver cancer due to alcohol use	Hepatitis G virus infection in patients with hepatocellular carcinoma in Recife, Brazil. . 2007; 37(8): 632-6.	Country	1996-1999	Scientific literature
Liver cancer due to alcohol use	Gonçalves CS, Pereira FE, Gayotto LC. Hepatocellular carcinoma in Brazil: report of a national survey (Florianópolis, SC, 1995). . 1997; 39(3): 165-70.		1992-1994	Scientific literature
Liver cancer due to alcohol use	Silva M, Mattos AA de, Fontes PRO, Waechter FL, Pereira-Lima L. Evaluation of hepatic resection for hepatocellular carcinoma on cirrhotic livers . . 2008; 45(2): 99-105.		1996-2005	Scientific literature
Liver cancer due to alcohol use	Clinical and epidemiological aspects of hepatocellular carcinoma in Brazil. . 2010; 65(12): 1285-90.		2004-2009	Scientific literature
Liver cancer due to alcohol use	Fontes PR, Schmidt Cerski CT, Hartmann AA, Kretzmann Filho NA. Evaluation of the C3435T polymorphism in the MDR1 gene in patients with hepatocellular carcinoma. . 2012; 11(6): 899-906.		2000-2009	Scientific literature
Liver cancer due to alcohol use	Gonçalves PL, Zago-Gomes Mda P, Gonçalves CS, Pereira FE. Hepatitis virus and hepatocellular carcinoma in Brazil: a report from the State of Espírito Santo. . 2014; 47(5): 559-63.		1993-2011	Scientific literature
Liver cancer due to other causes	Gonçalves PL, Zago-Gomes Mda P, Gonçalves CS, Pereira FE. Hepatitis virus and hepatocellular carcinoma in Brazil: a report from the State of Espírito Santo. . 2014; 47(5): 559-63.		1993-2011	Scientific literature
Liver cancer due to other causes	Clinical and epidemiological aspects of hepatocellular carcinoma in Brazil. . 2010; 65(12): 1285-90.		2004-2009	Scientific literature
Liver cancer due to other causes	Silva M, Mattos AA de, Fontes PRO, Waechter FL, Pereira-Lima L. Evaluation of hepatic resection for hepatocellular carcinoma on cirrhotic livers . . 2008; 45(2): 99-105.		1996-2005	Scientific literature
Liver cancer due to other causes	Fontes PR, Schmidt Cerski CT, Hartmann AA, Kretzmann Filho NA. Evaluation of the C3435T polymorphism in the MDR1 gene in patients with hepatocellular carcinoma. . 2012; 11(6): 899-906.		2000-2009	Scientific literature
Liver cancer due to other causes	Osório FMF, Lauar GM, Lima AS, Vidigal PVT, Ferrari TCA, Couto CA. Epidemiological aspects of hepatocellular carcinoma in a referral center of Minas Gerais, Brazil. . 2013; 50(2): 97-100.		1998-2010	Scientific literature
Liver cancer due to other causes	Gonçalves CS, Pereira FE, Gayotto LC. Hepatocellular carcinoma in Brazil: report of a national survey (Florianópolis, SC, 1995). . 1997; 39(3): 165-70.		1992-1994	Scientific literature
Liver cancer due to other causes	Hepatitis G virus infection in patients with hepatocellular carcinoma in Recife, Brazil. . 2007; 37(8): 632-6.	Country	1996-1999	Scientific literature
Larynx cancer	Institute for Health Metrics and Evaluation (IHME). IHME GBD 2015 DisMod Cancer Procedure Incidence Estimates.	Global	1990-2020	Modeled data
Non-melanoma skin cancer (squamous-cell carcinoma)	Minas Gerais Ministry of Health (Brazil), National Cancer Institute (Brazil). Brazil - Belo Horizonte BasePopWeb Database - Population Based Cancer Registry (RCBP Belo Horizonte) Statistics. Rio de Janeiro, Brazil: National Cancer Institute (Brazil).	Minas Gerais	2000-2008	Disease registry
Non-melanoma skin cancer (squamous-cell carcinoma)	National Cancer Institute (Brazil). Brazil - Distrito Federal BasePopWeb Database - Population Based Cancer Registry (RCBP Distrito Federal) Statistics. Rio de Janeiro, Brazil: National Cancer Institute (Brazil).	Distrito Federal	1999-2002	Disease registry
Non-melanoma skin cancer (squamous-cell carcinoma)	Municipal Secretariat of Health of Curitiba (Brazil), National Cancer Institute (Brazil). Brazil - Curitiba BasePopWeb Database - Population Based Cancer Registry (RCBP Curitiba) Statistics. Rio de Janeiro, Brazil: National Cancer Institute (Brazil).	Paraná	1998-2010	Disease registry
Non-melanoma skin cancer (squamous-cell carcinoma)	Association to Fight Cancer in Goiás (Brazil), National Cancer Institute (Brazil). Brazil - Goiânia BasePopWeb Database - Population Based Cancer Registry (RCBP Goiânia) Statistics. Rio de Janeiro, Brazil: National Cancer Institute (Brazil).	Goiás	1990-2009	Disease registry
Non-melanoma skin cancer (squamous-cell carcinoma)	National Cancer Institute (Brazil). Brazil - Espirito Santo BasePopWeb Database - Population Based Cancer Registry (RCBP do Estado do Espirito Santo) Statistics. Rio de Janeiro, Brazil: National Cancer Institute (Brazil).	Espírito Santo	1997-2012	Disease registry
Non-melanoma skin cancer (squamous-cell carcinoma)	Cancer Registry (RCBP Cuiaba) Statistics. Rio de Janeiro, Brazil: National Cancer Institute (Brazil).	Mato Grosso	2000-2007	Disease registry
Non-melanoma skin cancer (squamous-cell carcinoma)	Mato Grosso do Sul State Department of Health (Brazil), National Cancer Institute (Brazil). Brazil - Campo Grande BasePopWeb Database - Population Based Cancer Registry (RCBP Campo Grande) Statistics. Rio de Janeiro, Brazil: National Cancer Institute (Brazil).	Mato Grosso Do Sul	2001-2009	Disease registry
Non-melanoma skin cancer (squamous-cell carcinoma)	Cancer Registry (RCBP Fortaleza) Statistics. Rio de Janeiro, Brazil: National Cancer Institute (Brazil).	Ceará	1990-2006	Disease registry
Non-melanoma skin cancer (squamous-cell carcinoma)	National Cancer Institute (Brazil). Brazil - Florianópolis BasePopWeb Database - Population Based Cancer Registry (RCBP Florianópolis) Statistics. Rio de Janeiro, Brazil: National Cancer Institute (Brazil).	Santa Catarina	2008-2012	Disease registry

Non-melanoma skin cancer (squamous-cell carcinoma)	National Cancer Institute (Brazil). Brazil - Poços de Caldas BasePopWeb Database - Population Based Cancer Registry (RCBP Poços de Caldas) Statistics. Rio de Janeiro, Brazil: National Cancer Institute (Brazil).	Minas Gerais	2007-2011	Disease registry
Non-melanoma skin cancer (squamous-cell carcinoma)	National Cancer Institute (Brazil), Rio Grande do Sul State Center for Health Surveillance (CEVS) (Brazil), Rio Grande do Sul State Health Department (Brazil). Brazil - Porto Alegre BasePopWeb Database - Population Based Cancer Registry (RCBP Porto Alegre) Statistics. Rio de Janeiro, Brazil: National Cancer Institute (Brazil).	Rio Grande do Sul	1994-2006	Disease registry
Non-melanoma skin cancer (squamous-cell carcinoma)	Aristides Maltez Hospital (Brazil), Bahia League Against Cancer (Brazil), National Cancer Institute (Brazil). Brazil - Salvador BasePopWeb Database - Population Based Cancer Registry (RCBP Salvador) Statistics. Rio de Janeiro, Brazil: National Cancer Institute (Brazil).	Bahia	1996-2005	Disease registry
Non-melanoma skin cancer (squamous-cell carcinoma)	Cancer Registry (RCBP Roraima) Statistics. Rio de Janeiro, Brazil: National Cancer Institute (Brazil).	Roraima	2003-2009	Disease registry
Non-melanoma skin cancer (squamous-cell carcinoma)	Cancer Registry (RCBP Recife) Statistics. Rio de Janeiro, Brazil: National Cancer Institute (Brazil).	Pernambuco	1996-2010	Disease registry
Non-melanoma skin cancer (squamous-cell carcinoma)	National Cancer Institute (Brazil), São Paulo Population Based Cancer Registry. Brazil - Sao Paulo BasePopWeb Database - Population Based Cancer Registry (RCBP-SP) Statistics. Rio de Janeiro, Brazil: National Cancer Institute (Brazil).	São Paulo	1997-2010	Disease registry
Non-melanoma skin cancer (squamous-cell	Institute for Health Metrics and Evaluation (IHME). IHME GBD 2013 DisMod model input.	Global	1990-2013	Modeled data
Non-melanoma skin cancer (squamous-cell	Cancer Incidence in Five Continents Volumes I-VIII 1950-1997	Global	1973-1998	Disease registry
Non-melanoma skin cancer (squamous-cell carcinoma)	São Paulo Population Based Cancer Registry. Brazil - Sao Paulo Population Based Cancer Registry Statistics. São Paulo, Brazil: São Paulo Population Based Cancer Registry.	São Paulo	2011	Disease registry
Non-melanoma skin cancer (squamous-cell carcinoma)	Health Department of the State of Paraiba (Brazil), National Cancer Institute (Brazil). Brazil - João Pessoa BasePopWeb Database - Population Based Cancer Registry (RCBP João Pessoa) Statistics. Rio de Janeiro, Brazil: National Cancer Institute (Brazil).	Paraíba	1999-2006	Disease registry
Non-melanoma skin cancer (squamous-cell carcinoma)	Amazonas State Foundation Oncology Control Center (FCECON), National Cancer Institute (Brazil). Brazil - Manaus BasePopWeb Database - Population Based Cancer Registry (RCBP Manaus) Statistics. Rio de Janeiro, Brazil: National Cancer Institute (Brazil).	Amazonas	1999-2006	Disease registry
Non-melanoma skin cancer (squamous-cell carcinoma)	National Cancer Institute (Brazil). Brazil - Natal BasePopWeb Database - Population Based Cancer Registry (RCBP Natal) Statistics. Rio de Janeiro, Brazil: National Cancer Institute (Brazil).	Rio Grande do Norte	1999-2005	Disease registry
Non-melanoma skin cancer (squamous-cell carcinoma)	Ministry of Health of Tocantins (Brazil), National Cancer Institute (Brazil). Brazil - Palmas BasePopWeb Database - Population Based Cancer Registry (RCBP Palmas) Statistics. Rio de Janeiro, Brazil: National Cancer Institute (Brazil).	Tocantins	2001-2005	Disease registry
Non-melanoma skin cancer (squamous-cell carcinoma)	National Cancer Institute (Brazil), Pará State Department of Public Health (SESPA) (Brazil). Brazil - Belém BasePopWeb Database - Population Based Cancer Registry (RCBP Belém) Statistics. Rio de Janeiro, Brazil: National Cancer Institute (Brazil).	Pará	1996-2009	Disease registry
Non-melanoma skin cancer (squamous-cell carcinoma)	National Cancer Institute (Brazil), Oncology Center, Sergipe Emergency Hospital (Brazil). Brazil - Aracaju BasePopWeb Database - Population Based Cancer Registry (RCBP Aracaju) Statistics. Rio de Janeiro, Brazil: National Cancer Institute (Brazil).	Sergipe	1996-2012	Disease registry
Non-melanoma skin cancer (squamous-cell carcinoma)	Amaral Carvalho Hospital (Brazil), National Cancer Institute (Brazil), Secretary of Municipal Health of Jau (Brazil). Brazil - Jahú BasePopWeb Database - Population Based Cancer Registry (RCBP Jahú) Statistics. Rio de Janeiro, Brazil: National Cancer Institute (Brazil).	São Paulo	1996-2012	Disease registry
Non-melanoma skin cancer (squamous-cell carcinoma)	Cancer Registry (RCBP DRS Barretos) Statistics. Rio de Janeiro, Brazil: National Cancer Institute (Brazil).	São Paulo	2008-2013	Disease registry
Non-melanoma skin cancer (squamous-cell carcinoma)	Cancer Registry (RCBP Teresina) Statistics. Rio de Janeiro, Brazil: National Cancer Institute (Brazil).	Piaui	2001-2005	Disease registry
Non-melanoma skin cancer (squamous-cell carcinoma)	Cancer Registry (RCBP Santos) Statistics. Rio de Janeiro, Brazil: National Cancer Institute (Brazil).	São Paulo	2008-2009	Disease registry
Non-melanoma skin cancer (basal-cell carcinoma)	National Cancer Institute (Brazil). Brazil - Florianópolis BasePopWeb Database - Population Based Cancer Registry (RCBP Florianópolis) Statistics. Rio de Janeiro, Brazil: National Cancer Institute (Brazil).	Santa Catarina	2008-2012	Disease registry
Non-melanoma skin cancer (basal-cell carcinoma)	National Cancer Institute (Brazil). Brazil - Distrito Federal BasePopWeb Database - Population Based Cancer Registry (RCBP Distrito Federal) Statistics. Rio de Janeiro, Brazil: National Cancer Institute (Brazil).	Distrito Federal	1999-2002	Disease registry
Non-melanoma skin cancer (basal-cell carcinoma)	Cancer Registry (RCBP Teresina) Statistics. Rio de Janeiro, Brazil: National Cancer Institute (Brazil).	Piaui	2000-2006	Disease registry
Non-melanoma skin cancer (basal-cell carcinoma)	Municipal Secretariat of Health of Curitiba (Brazil), National Cancer Institute (Brazil). Brazil - Curitiba BasePopWeb Database - Population Based Cancer Registry (RCBP Curitiba) Statistics. Rio de Janeiro, Brazil: National Cancer Institute (Brazil).	Paraná	1998-2010	Disease registry
Non-melanoma skin cancer (basal-cell carcinoma)	National Cancer Institute (Brazil), Rio Grande do Sul State Center for Health Surveillance (CEVS) (Brazil), Rio Grande do Sul State Health Department (Brazil). Brazil - Porto Alegre BasePopWeb Database - Population Based Cancer Registry (RCBP Porto Alegre) Statistics. Rio de Janeiro, Brazil: National Cancer Institute (Brazil).	Rio Grande do Sul	1993-2006	Disease registry
Non-melanoma skin cancer (basal-cell carcinoma)	Cancer Registry (RCBP Recife) Statistics. Rio de Janeiro, Brazil: National Cancer Institute (Brazil).	Pernambuco	1995-2010	Disease registry
Non-melanoma skin cancer (basal-cell carcinoma)	Cancer Registry (RCBP Cuiaba) Statistics. Rio de Janeiro, Brazil: National Cancer Institute (Brazil).	Mato Grosso	2000-2007	Disease registry
Non-melanoma skin cancer (basal-cell carcinoma)	Cancer Registry (RCBP Roraima) Statistics. Rio de Janeiro, Brazil: National Cancer Institute (Brazil).	Roraima	2003-2010	Disease registry
Non-melanoma skin cancer (basal-cell carcinoma)	Association to Fight Cancer in Goiás (Brazil), National Cancer Institute (Brazil). Brazil - Goiânia BasePopWeb Database - Population Based Cancer Registry (RCBP Goiânia) Statistics. Rio de Janeiro, Brazil: National Cancer Institute (Brazil).	Goiás	1990-2009	Disease registry
Non-melanoma skin cancer (basal-cell carcinoma)	Cancer Registry (RCBP Santos) Statistics. Rio de Janeiro, Brazil: National Cancer Institute (Brazil).	São Paulo	2008-2009	Disease registry
Non-melanoma skin cancer (basal-cell carcinoma)	Mato Grosso do Sul State Department of Health (Brazil), National Cancer Institute (Brazil). Brazil - Campo Grande BasePopWeb Database - Population Based Cancer Registry (RCBP Campo Grande) Statistics. Rio de Janeiro, Brazil: National Cancer Institute (Brazil).	Mato Grosso Do Sul	2000-2009	Disease registry
Non-melanoma skin cancer (basal-cell carcinoma)	National Cancer Institute (Brazil). Brazil - Poços de Caldas BasePopWeb Database - Population Based Cancer Registry (RCBP Poços de Caldas) Statistics. Rio de Janeiro, Brazil: National Cancer Institute (Brazil).	Minas Gerais	2007-2011	Disease registry
Non-melanoma skin cancer (basal-cell carcinoma)	National Cancer Institute (Brazil), São Paulo Population Based Cancer Registry. Brazil - Sao Paulo BasePopWeb Database - Population Based Cancer Registry (RCBP-SP) Statistics. Rio de Janeiro, Brazil: National Cancer Institute (Brazil).	São Paulo	1997-2010	Disease registry
Non-melanoma skin cancer (basal-cell carcinoma)	National Cancer Institute (Brazil). Brazil - Espirito Santo BasePopWeb Database - Population Based Cancer Registry (RCBP do Estado do Espirito Santo) Statistics. Rio de Janeiro, Brazil: National Cancer Institute (Brazil).	Espírito Santo	1997-2012	Disease registry
Non-melanoma skin cancer (basal-cell carcinoma)	National Cancer Institute (Brazil). Brazil - Natal BasePopWeb Database - Population Based Cancer Registry (RCBP Natal) Statistics. Rio de Janeiro, Brazil: National Cancer Institute (Brazil).	Rio Grande do Norte	1999-2005	Disease registry
Non-melanoma skin cancer (basal-cell carcinoma)	Aristides Maltez Hospital (Brazil), Bahia League Against Cancer (Brazil), National Cancer Institute (Brazil). Brazil - Salvador BasePopWeb Database - Population Based Cancer Registry (RCBP Salvador) Statistics. Rio de Janeiro, Brazil: National Cancer Institute (Brazil).	Bahia	1996-2005	Disease registry
Non-melanoma skin cancer (basal-cell carcinoma)	Bariani RL, Nahas FX, Barbosa MVJ, Farah AB, Ferreira LM. Basal cell carcinoma: an updated epidemiological and therapeutically profile of an urban population. . 2006; 21(2): 66-73.		2001-2003	Scientific literature
Non-melanoma skin cancer (basal-cell carcinoma)	Amazonas State Foundation Oncology Control Center (FCECON), National Cancer Institute (Brazil). Brazil - Manaus BasePopWeb Database - Population Based Cancer Registry (RCBP Manaus) Statistics. Rio de Janeiro, Brazil: National Cancer Institute (Brazil).	Amazonas	1999-2006	Disease registry
Non-melanoma skin cancer (basal-cell carcinoma)	Cancer Registry (RCBP DRS Barretos) Statistics. Rio de Janeiro, Brazil: National Cancer Institute (Brazil).	São Paulo	2008-2013	Disease registry
Non-melanoma skin cancer (basal-cell carcinoma)	Health Department of the State of Paraiba (Brazil), National Cancer Institute (Brazil). Brazil - João Pessoa BasePopWeb Database - Population Based Cancer Registry (RCBP João Pessoa) Statistics. Rio de Janeiro, Brazil: National Cancer Institute (Brazil).	Paraíba	1999-2010	Disease registry
Non-melanoma skin cancer (basal-cell carcinoma)	Minas Gerais Ministry of Health (Brazil), National Cancer Institute (Brazil). Brazil - Belo Horizonte BasePopWeb Database - Population Based Cancer Registry (RCBP Belo Horizonte) Statistics. Rio de Janeiro, Brazil: National Cancer Institute (Brazil).	Minas Gerais	2000-2008	Disease registry
Non-melanoma skin cancer (basal-cell carcinoma)	National Cancer Institute (Brazil), Pará State Department of Public Health (SESPA) (Brazil). Brazil - Belém BasePopWeb Database - Population Based Cancer Registry (RCBP Belém) Statistics. Rio de Janeiro, Brazil: National Cancer Institute (Brazil).	Pará	1996-2009	Disease registry
Non-melanoma skin cancer (basal-cell carcinoma)	Amaral Carvalho Hospital (Brazil), National Cancer Institute (Brazil), Secretary of Municipal Health of Jau (Brazil). Brazil - Jahú BasePopWeb Database - Population Based Cancer Registry (RCBP Jahú) Statistics. Rio de Janeiro, Brazil: National Cancer Institute (Brazil).	São Paulo	1996-2012	Disease registry
Non-melanoma skin cancer (basal-cell carcinoma)	São Paulo Population Based Cancer Registry. Brazil - Sao Paulo Population Based Cancer Registry Statistics. São Paulo, Brazil: São Paulo Population Based Cancer Registry.	São Paulo	2011	Disease registry
Non-melanoma skin cancer (basal-cell carcinoma)	National Cancer Institute (Brazil), Oncology Center, Sergipe Emergency Hospital (Brazil). Brazil - Aracaju BasePopWeb Database - Population Based Cancer Registry (RCBP Aracaju) Statistics. Rio de Janeiro, Brazil: National Cancer Institute (Brazil).	Sergipe	1996-2012	Disease registry
Non-melanoma skin cancer (basal-cell carcinoma)	Cancer Incidence in Five Continents Volumes I-VIII 1950-1997	Global	1973-1998	Disease registry
Non-melanoma skin cancer (basal-cell carcinoma)	Institute for Health Metrics and Evaluation (IHME). IHME GBD 2013 DisMod model input.	Global	1990-2013	Modeled data

Non-melanoma skin cancer (basal-cell carcinoma)	Ministry of Health of Tocantins (Brazil), National Cancer Institute (Brazil). Brazil - Palmas BasePopWeb Database - Population Based Cancer Registry (RCBP Palmas) Statistics. Rio de Janeiro, Brazil: National Cancer Institute (Brazil).	Tocantins	2000-2012	Disease registry
Non-melanoma skin cancer (basal-cell carcinoma)	Cancer Registry (RCBP Fortaleza) Statistics. Rio de Janeiro, Brazil: National Cancer Institute (Brazil).	Ceará	1990-2006	Disease registry
Breast cancer	Institute for Health Metrics and Evaluation (IHME). IHME GBD 2015 DisMod Cancer Procedure Incidence Estimates.	Global	1990-2020	Modeled data
Prostate cancer	Institute for Health Metrics and Evaluation (IHME). IHME GBD 2015 DisMod Cancer Procedure Incidence Estimates.	Global	1990-2020	Modeled data
Bladder cancer	Institute for Health Metrics and Evaluation (IHME). IHME GBD 2015 DisMod Cancer Procedure Incidence Estimates.	Global	1990-2020	Modeled data
Rheumatic heart disease	Brazil World Health Survey 2003	Country	2002-2003	Survey
Rheumatic heart disease	Alves Meira ZM, de Castilho SR, Lins Barros MV, Maria Vitarelli A, Diniz Capanema F, Moreira NS, Moreira Camargos PA, Coelho Mota CC. Prevalence of rheumatic fever in children from a public high school in Belo Horizonte. . 1995; 65(4): 331-4.		1992	Scientific literature
Rheumatic heart disease	Miranda LP, Camargos PA, Torres RM, Meira ZM. Prevalence of rheumatic heart disease in a public school of Belo Horizonte. . 2014; 103(2.0): 89-97.		2010-2011	Scientific literature
Rheumatic heart disease	Institute for Health Metrics and Evaluation (IHME). IHME GBD 2015 DisMod Low-income Endemic Rheumatic Heart Disease Cause-specific Mortality Rates.	Global	1990-2016	Modeled data
Rheumatic heart disease	Institute for Health Metrics and Evaluation (IHME). IHME GBD 2015 DisMod High-income Non-endemic Rheumatic Heart Disease Cause-specific Mortality Rates.	Global	1990-2016	Modeled data
Rheumatic heart disease	Brazilian Institute of Geography and Statistics (IBGE), Ministry of Health (Brazil), Ministry of Planning, Budget, and Management (Brazil). Brazil National Health Survey 2013. Rio de Janeiro, Brazil: Brazilian Institute of Geography and Statistics (IBGE).	Country	2013-2014	Survey
Rheumatic heart disease	Health Institute (São Paulo, Brazil), State University of Campinas, São Paulo Municipal Health Department, São Paulo State University, University of São Paulo. Brazil - São Paulo Health Survey 2008-2009.	São Paulo	2002-2008	Survey
Ischemic heart disease	Institute for Health Metrics and Evaluation (IHME). IHME GBD DisMod Ischemic Heart Disease Excess Mortality Estimates.	Global	1990-2016	Modeled data
Ischemic heart disease	Health Institute (São Paulo, Brazil), State University of Campinas, São Paulo Municipal Health Department, São Paulo State University, University of São Paulo. Brazil - São Paulo Health Survey 2008-2009.	São Paulo	2002-2008	Survey
Ischemic heart disease	Brazilian Institute of Geography and Statistics (IBGE), Ministry of Health (Brazil), Ministry of Planning, Budget, and Management (Brazil). Brazil National Health Survey 2013. Rio de Janeiro, Brazil: Brazilian Institute of Geography and Statistics (IBGE).	Country	2013-2014	Survey
Ischemic heart disease	Institute for Health Metrics and Evaluation (IHME). IHME GBD 2015 DisMod Ischemic Heart Diseases Excess Mortality Estimates.	Global	1990-2016	Modeled data
Ischemic heart disease	Institute for Health Metrics and Evaluation (IHME). Cause of Death Ensemble Modeling Results	Global	1980-2015	Modeled data
Ischemic heart disease	myocardial infarction in Salvador, Brazil: I. Incidence, lethality, and mortality. . 1987; 21(1): 28-37.		1982	Scientific literature
Ischemic heart disease	Piva e Mattos LAL, Berwanger O, Santos ES dos, Reis HJL, Romano ER, Petriz JLF, Sousa ACS, Neuenschwander FC, Guimarães JI, Andrade JP de. Clinical outcomes at 30 days in the Brazilian Registry of Acute Coronary Syndromes (ACCEPT). . 2013; 100(1): 6[illegible]13.		2010-2011	Scientific literature
Ischemic heart disease	Brazil World Health Survey 2003	Country	2002-2003	Survey
Cerebrovascular disease	Fernandes TG, Goulart AC, Santos-Junior WR, Alencar AP, Benseñor IM, Lotufo PA. Educational levels and the functional dependence of ischemic stroke survivors. . 2012; 28(8): 1581[illegible]90.		2006-2009	Scientific literature
Ischemic stroke	Health Institute (São Paulo, Brazil), State University of Campinas, São Paulo Municipal Health Department, São Paulo State University, University of São Paulo. Brazil - São Paulo Health Survey 2008-2009.	São Paulo	2002-2008	Survey
Ischemic stroke	Institute for Health Metrics and Evaluation (IHME). IHME GBD 2015 DisMod Cerebrovascular Disease Excess Mortality Estimates.	Global	1990-2016	Modeled data
Ischemic stroke	Fernandes TG, Goulart AC, Santos-Junior WR, Alencar AP, Benseñor IM, Lotufo PA. Educational levels and the functional dependence of ischemic stroke survivors. . 2012; 28(8): 1581[illegible]90.		2006-2009	Scientific literature
Ischemic stroke	Cerebrovascular Disease, Hemorragic Stroke and Ischemic Stroke Cause-specific Mortality Rate Estimates.	Global	1990-2016	Modeled data
Ischemic stroke	Cabral NL, Gonçalves ARR, Longo AL, Moro CHC, Costa G, Amaral CH, Fonseca L a M, Eluf-Neto J. Incidence of stroke subtypes, prognosis and prevalence of risk factors in Joinville, Brazil: a 2 year community based study. . 2009; 80(7): 755-61.		2005-2006	Scientific literature
Ischemic stroke	Minelli C, Fu Fen L, Camara Minelli DP. Stroke Incidence, Prognosis, 30-Day, and 1-Year Case Fatality Rates in Matão, Brazil. . 2007; 38(11): 2906-11.		2003-2004	Scientific literature
Ischemic stroke	Brazilian Institute of Geography and Statistics (IBGE), Ministry of Health (Brazil), Ministry of Planning, Budget, and Management (Brazil). Brazil National Health Survey 2013. Rio de Janeiro, Brazil: Brazilian Institute of Geography and Statistics (IBGE).	Country	2013-2014	Survey
Ischemic stroke	Brazil - São Paulo Survey on Health, Well-Being, and Aging in Latin America and the Caribbean 1999-2000	São Paulo	1999-2000	Survey
Ischemic stroke	Brazil World Health Survey 2003	Country	2002-2003	Survey
Hemorrhagic stroke	Health Institute (São Paulo, Brazil), State University of Campinas, São Paulo Municipal Health Department, São Paulo State University, University of São Paulo. Brazil - São Paulo Health Survey 2008-2009.	São Paulo	2002-2008	Survey
Hemorrhagic stroke	Brazil World Health Survey 2003	Country	2002-2003	Survey
Hemorrhagic stroke	Institute for Health Metrics and Evaluation (IHME). IHME GBD 2015 DisMod Cerebrovascular Disease Excess Mortality Estimates.	Global	1990-2016	Modeled data
Hemorrhagic stroke	Minelli C, Fu Fen L, Camara Minelli DP. Stroke Incidence, Prognosis, 30-Day, and 1-Year Case Fatality Rates in Matão, Brazil. . 2007; 38(11): 2906-11.		2003-2004	Scientific literature
Hemorrhagic stroke	Cabral NL, Gonçalves ARR, Longo AL, Moro CHC, Costa G, Amaral CH, Fonseca L a M, Eluf-Neto J. Incidence of stroke subtypes, prognosis and prevalence of risk factors in Joinville, Brazil: a 2 year community based study. . 2009; 80(7): 755-61.		2005-2006	Scientific literature
Hemorrhagic stroke	Brazilian Institute of Geography and Statistics (IBGE), Ministry of Health (Brazil), Ministry of Planning, Budget, and Management (Brazil). Brazil National Health Survey 2013. Rio de Janeiro, Brazil: Brazilian Institute of Geography and Statistics (IBGE).	Country	2013-2014	Survey
Hemorrhagic stroke	Fernandes TG, Goulart AC, Santos-Junior WR, Alencar AP, Benseñor IM, Lotufo PA. Educational levels and the functional dependence of ischemic stroke survivors. . 2012; 28(8): 1581[illegible]90.		2006-2009	Scientific literature
Hemorrhagic stroke	Cerebrovascular Disease, Hemorragic Stroke and Ischemic Stroke Cause-specific Mortality Rate Estimates.	Global	1990-2016	Modeled data
Hemorrhagic stroke	Brazil - São Paulo Survey on Health, Well-Being, and Aging in Latin America and the Caribbean 1999-2000	São Paulo	1999-2000	Survey
Myocarditis	Health Institute (São Paulo, Brazil), State University of Campinas, São Paulo Municipal Health Department, São Paulo State University, University of São Paulo. Brazil - São Paulo Health Survey 2008-2009.	São Paulo	2002-2008	Survey
Myocarditis	Brazil World Health Survey 2003	Country	2002-2003	Survey
Myocarditis	Brazilian Institute of Geography and Statistics (IBGE), Ministry of Health (Brazil), Ministry of Planning, Budget, and Management (Brazil). Brazil National Health Survey 2013. Rio de Janeiro, Brazil: Brazilian Institute of Geography and Statistics (IBGE).	Country	2013-2014	Survey
Atrial fibrillation and flutter	Health Institute (São Paulo, Brazil), State University of Campinas, São Paulo Municipal Health Department, São Paulo State University, University of São Paulo. Brazil - São Paulo Health Survey 2008-2009.	São Paulo	2002-2008	Survey
Atrial fibrillation and flutter	Fornari LS, Calderaro D, Nassar IB, Lauretti C, Nakamura L, Bagnatori R, Ageno W, Caramelli B. Misuse of antithrombotic therapy in atrial fibrillation patients: frequent, pervasive and persistent. . 2006; 23(1): 65-71.		2002	Scientific literature
Atrial fibrillation and flutter	Brazil World Health Survey 2003	Country	2002-2003	Survey
Atrial fibrillation and flutter	Marcolino MS, Palhares DMF, Benjamin EJ, Ribeiro AL. Atrial fibrillation: prevalence in a large database of primary care patients in Brazil. . 2015; 17(12): 1787[illegible]90.		2011	Scientific literature
Atrial fibrillation and flutter	Institute for Health Metrics and Evaluation (IHME). IHME GBD Atrial Fibrillation and Flutter Excess Mortality Estimates.	Global	1990-2016	Modeled data
Atrial fibrillation and flutter	Brazilian Institute of Geography and Statistics (IBGE), Ministry of Health (Brazil), Ministry of Planning, Budget, and Management (Brazil). Brazil National Health Survey 2013. Rio de Janeiro, Brazil: Brazilian Institute of Geography and Statistics (IBGE).	Country	2013-2014	Survey
Endocarditis	Brazilian Institute of Geography and Statistics (IBGE), Ministry of Health (Brazil), Ministry of Planning, Budget, and Management (Brazil). Brazil National Health Survey 2013. Rio de Janeiro, Brazil: Brazilian Institute of Geography and Statistics (IBGE).	Country	2013-2014	Survey
Endocarditis	Ministry of Health (Brazil). Brazil Hospital Information System 2013-2014.		2013-2017	Administrative record
Endocarditis	Ministry of Health (Brazil). Brazil Hospital Information System 1998-2002.		1998-2002	Administrative record
Endocarditis	Institute for Health Metrics and Evaluation (IHME). IHME Cause of Death Ensemble Modeling (CODEM) Results 2010.	Global	1990-2010	Modeled data
Endocarditis	Ministry of Health (Brazil). Brazil Hospital Information System 2003-2007.		2003-2007	Administrative record
Endocarditis	Ministry of Health (Brazil). Brazil Hospital Information System 1997. Rio de Janeiro, Brazil: Ministry of Health (Brazil).	Country	1993-1997	Administrative record

Endocarditis	Brazil World Health Survey 2003	Country	2002-2003	Survey
Endocarditis	Ministry of Health (Brazil). Brazil Hospital Information System 2008-2012.		2008-2012	Administrative record
Endocarditis	Health Institute (São Paulo, Brazil), State University of Campinas, São Paulo Municipal Health Department, São Paulo State University, University of São Paulo. Brazil - São Paulo Health Survey 2008-2009.	São Paulo	2002-2008	Survey
Chronic obstructive pulmonary disease	Menezes A, Macedo SC, Gigante DP, da Costa JD, Olinto MT, Fiss E, Chatkin M, Hallal PC, Victora CG. Prevalence and Risk Factors for Chronic Obstructive Pulmonary Disease According to Symptoms and Spirometry. . 2004; 1(2): 173-9.		2001	Scientific literature
Chronic obstructive pulmonary disease	M, Talamo C, Hallal PC, Victora CG, PLATINO Team. Chronic obstructive pulmonary disease in five Latin American cities (the PLATINO study): a prevalence study. . 2005; 366(9500): 1875-81.	Country	2002-2004	Scientific literature
Silicosis	Brazil World Health Survey 2003	Country	2002-2003	Survey
Silicosis	Ministry of Health (Brazil). Brazil Hospital Information System 1998-2002.		1998-2002	Administrative record
Silicosis	Brazilian Institute of Geography and Statistics (IBGE), Ministry of Health (Brazil), Ministry of Planning, Budget, and Management (Brazil). Brazil National Health Survey 2013. Rio de Janeiro, Brazil: Brazilian Institute of Geography and Statistics (IBGE).	Country	2013-2014	Survey
Silicosis	Ministry of Health (Brazil). Brazil Hospital Information System 2003-2007.		2003-2007	Administrative record
Silicosis	Health Institute (São Paulo, Brazil), State University of Campinas, São Paulo Municipal Health Department, São Paulo State University, University of São Paulo. Brazil - São Paulo Health Survey 2008-2009.	São Paulo	2002-2008	Survey
Silicosis	Ministry of Health (Brazil). Brazil Hospital Information System 2013-2014.		2013-2017	Administrative record
Silicosis	Ministry of Health (Brazil). Brazil Hospital Information System 1997. Rio de Janeiro, Brazil: Ministry of Health (Brazil).	Country	1993-1997	Administrative record
Silicosis	Ministry of Health (Brazil). Brazil Hospital Information System 2008-2012.		2008-2012	Administrative record
Asbestosis	Brazil World Health Survey 2003	Country	2002-2003	Survey
Asbestosis	Ministry of Health (Brazil). Brazil Hospital Information System 1997. Rio de Janeiro, Brazil: Ministry of Health (Brazil).	Country	1993-1997	Administrative record
Asbestosis	Ministry of Health (Brazil). Brazil Hospital Information System 2013-2014.		2013-2017	Administrative record
Asbestosis	Health Institute (São Paulo, Brazil), State University of Campinas, São Paulo Municipal Health Department, São Paulo State University, University of São Paulo. Brazil - São Paulo Health Survey 2008-2009.	São Paulo	2002-2008	Survey
Asbestosis	Ministry of Health (Brazil). Brazil Hospital Information System 2008-2012.		2008-2012	Administrative record
Asbestosis	Ministry of Health (Brazil). Brazil Hospital Information System 1998-2002.		1998-2002	Administrative record
Asbestosis	Brazilian Institute of Geography and Statistics (IBGE), Ministry of Health (Brazil), Ministry of Planning, Budget, and Management (Brazil). Brazil National Health Survey 2013. Rio de Janeiro, Brazil: Brazilian Institute of Geography and Statistics (IBGE).	Country	2013-2014	Survey
Asbestosis	Ministry of Health (Brazil). Brazil Hospital Information System 2003-2007.		2003-2007	Administrative record
Coal workers pneumoconiosis	Health Institute (São Paulo, Brazil), State University of Campinas, São Paulo Municipal Health Department, São Paulo State University, University of São Paulo. Brazil - São Paulo Health Survey 2008-2009.	São Paulo	2002-2008	Survey
Coal workers pneumoconiosis	Ministry of Health (Brazil). Brazil Hospital Information System 2003-2007.		2003-2007	Administrative record
Coal workers pneumoconiosis	Brazil World Health Survey 2003	Country	2002-2003	Survey
Coal workers pneumoconiosis	Ministry of Health (Brazil). Brazil Hospital Information System 1997. Rio de Janeiro, Brazil: Ministry of Health (Brazil).	Country	1993-1997	Administrative record
Coal workers pneumoconiosis	Ministry of Health (Brazil). Brazil Hospital Information System 2013-2014.		2013-2017	Administrative record
Coal workers pneumoconiosis	Ministry of Health (Brazil). Brazil Hospital Information System 1998-2002.		1998-2002	Administrative record
Coal workers pneumoconiosis	Brazilian Institute of Geography and Statistics (IBGE), Ministry of Health (Brazil), Ministry of Planning, Budget, and Management (Brazil). Brazil National Health Survey 2013. Rio de Janeiro, Brazil: Brazilian Institute of Geography and Statistics (IBGE).	Country	2013-2014	Survey
Coal workers pneumoconiosis	Ministry of Health (Brazil). Brazil Hospital Information System 2008-2012.		2008-2012	Administrative record
Other pneumoconiosis	Brazilian Institute of Geography and Statistics (IBGE), Ministry of Health (Brazil), Ministry of Planning, Budget, and Management (Brazil). Brazil National Health Survey 2013. Rio de Janeiro, Brazil: Brazilian Institute of Geography and Statistics (IBGE).	Country	2013-2014	Survey
Other pneumoconiosis	Ministry of Health (Brazil). Brazil Hospital Information System 2008-2012.		2008-2012	Administrative record
Other pneumoconiosis	Brazil World Health Survey 2003	Country	2002-2003	Survey
Other pneumoconiosis	Ministry of Health (Brazil). Brazil Hospital Information System 1997. Rio de Janeiro, Brazil: Ministry of Health (Brazil).	Country	1993-1997	Administrative record
Other pneumoconiosis	Ministry of Health (Brazil). Brazil Hospital Information System 2013-2014.		2013-2017	Administrative record
Other pneumoconiosis	Ministry of Health (Brazil). Brazil Hospital Information System 2003-2007.		2003-2007	Administrative record
Other pneumoconiosis	Health Institute (São Paulo, Brazil), State University of Campinas, São Paulo Municipal Health Department, São Paulo State University, University of São Paulo. Brazil - São Paulo Health Survey 2008-2009.	São Paulo	2002-2008	Survey
Other pneumoconiosis	Ministry of Health (Brazil). Brazil Hospital Information System 1998-2002.		1998-2002	Administrative record
Asthma	Ministry of Health (Brazil), Secretariat of Health Surveillance, Ministry of Health (Brazil). Brazil Surveillance System of Risk Factors for Chronic Diseases by Telephone Interviews 2010.	Country	2010	Survey
Asthma	Silva AA, Barbieri MA, Cardoso VC, Batista RF, Simões VM, Vianna EO, Gutierrez MR, Figueiredo ML, Silva NA, Pereira TS, Rodriguez JD, Loureiro SR, Ribeiro VS, Bettiol H. Prevalence of non-communicable diseases in Brazilian children: follow-up at school age of two Brazilian birth cohorts of the 1990's. . 2011; 11: 486.		2004-2006	Scientific literature
Asthma	associated risk factors among infants in Recife, Pernambuco State, Brazil. . 2011; 27(8): 1551-9.		2007	Scientific literature
Asthma	morbidity in 13-14-year-old schoolchildren in the city of Fortaleza, Ceará State, Brazil. . 2011; 27(1): 103-12.		2006-2007	Scientific literature
Asthma	Lima WL, Lima EVNCL, Costa M do R da SR, Santos AM dos, Silva AAM da, Costa ES. Asthma and associated factors in students 13 and 14 years of age in São Luís, Maranhão State, Brazil. . 2012; 28(6): 1046-56.		2008-2009	Scientific literature
Asthma	Jucá SCBMP, Takano OA, Moraes LSL, Guimarães LV. Asthma prevalence and risk factors in adolescents 13 to 14 years of age in Cuiabá, Mato Grosso State, Brazil. . 2012; 28(4): 689-97.		2008	Scientific literature
Asthma	Chong Neto HJ, Rosário NA, Grasselli EA, Silva FC e, Bojarski L de FM, Rosário CS, Rosário BA, Chong FH. Recurrent wheezing in infants: epidemiological changes. . 2011; 87(6): 547-50.		2005-2010	Scientific literature
Asthma	Toledo MF, Rozov T, Leone C. Prevalence of asthma and allergies in 13- to 14-year-old adolescents and the frequency of risk factors in carriers of current asthma in Taubaté, São Paulo, Brazil. . 2011; 39(5): 284-90.		2008-2010	Scientific literature
Asthma	Souza da Cunha S, Barreto ML, Fiaccone RL, Cooper PJ, Alcantara-Neves NM, Simões S de M, Cruz AA, Rodrigues LC. Asthma cases in childhood attributed to atopy in tropical area in Brazil. . 2010; 28(6): 405-11.		2005-2006	Scientific literature
Asthma	Silva R de CR, Assis AMO, Goncalves MS, Fiaccone RL, Matos SMA, Barreto ML, Pinto E de J, Silva LA da, Rodrigues LC, Alcantara-Neves NM. The prevalence of wheezing and its association with body mass index and abdominal obesity in children. . 2013; 50(3): 267-73.		2010	Scientific literature
Asthma	Wehrmeister FC, Peres KG de A. Regional inequalities in the prevalence of asthma diagnosis in children: an analysis of the Brazilian National Household Sample Survey, 2003. . 2010; 26(9): 1839-52.		2003	Scientific literature
Asthma	Roelofs R, Gurgel RQ, Wendte J, Polderman J, Barreto-Filho JAS, Solé D, Motta-Franco J, De Munter J, Agyemang C. Relationship between asthma and high blood pressure among adolescents in Aracaju, Brazil. . 2010; 47(6): 639-43.		2008	Scientific literature
Asthma	Sousa CA, Cesar CL, Barros MB, Carandina L, Goldbaum M, Pereira JC. [Prevalence of asthma and risk factors associated: population based study in Sao Paulo, Southeastern Brazil, 2008-2009]. . 2012; 46(5): 825-33.		2008-2009	Scientific literature
Asthma	Mallol J, Solé D, Baeza-Bacab M, Aguirre-Camposano V, Soto-Quiros M, Baena-Cagnani C, Latin American ISAAC Group. Regional variation in asthma symptom prevalence in Latin American children. . 2010; 47(6): 644-50.		2001-2003	Scientific literature
Asthma	rhinitis among adolescents in the city of Fortaleza, Brazil: temporal changes. . 2013; 39(2): 128-37.		2006-2010	Scientific literature
Asthma	Schuh C, Fritscher LG, Chapman KR, Fritscher CC. The prevalence of asthma and atopy in schoolchildren from Porto Alegre, Brazil, has plateaued. . 2015; 109(3): 308-11.		2013	Scientific literature
Asthma	Wilmer FA, Maurici R, Nazario CA, Nazario KC, Passaro PF, Piazza HE, Bertoldi RA, Pizzichini E, Pizzichini MM. Temporal trends in the prevalence of asthma and rhinoconjunctivitis in adolescents. . 2015; 49: nan.		2001-2012	Scientific literature
Asthma	Brazilian Institute of Geography and Statistics (IBGE), Ministry of Education (Brazil), Ministry of Health (Brazil), Ministry of Planning, Budget, and Management (Brazil), Secretariat of Health Surveillance, Ministry of Health (Brazil). Brazil National Survey of School Health 2012.	Country	2012	Survey
Asthma	Worldwide variations in the prevalence of asthma symptoms: the International Study of Asthma and Allergies in Childhood (ISAAC)	Country	1993-1995	Scientific literature
Asthma	Brazil World Health Survey 2003	Country	2003	Survey

Asthma	Alcantara-Neves NM, Cruz ÁA, Simões S de M, Barreto ML, SCAALA (Social Change, Asthma and Allergy in Latin America) Study Group. Overweight, asthma symptoms, atopy and pulmonary function in children of 4-12 years of age: findings from the SCAALA cohort in Salvador, Bahia, Brazil. . 2011; 14(7): 1270-8.		2005	Scientific literature
Asthma	Lai CK, Beasley R, Crane J, Foliaki S, Shah J, Weiland S. Global variation in the prevalence and severity of asthma symptoms: phase three of the International Study of Asthma and Allergies in Childhood (ISAAC). . 2009; 64(6): 476-483.		2001-2003	Scientific literature
Asthma	De Farias MR de C, Rosa AM, Hacon S de S, de Castro HA, Ignotti E. Prevalence of asthma in schoolchildren in Alta Floresta- a municipality in the southeast of the Brazilian Amazon. . 2010; 13(1): 49-57.		2007	Scientific literature
Asthma	Fiori NS, Gonçalves H, Dumith SC, Cesar MADC, Menezes AMB, Macedo SEC. Ten-year trends in prevalence of asthma in adults in southern Brazil: comparison of two population-based studies. . 2012; 28(1): 135-44.		2010	Scientific literature
Asthma	Freitas MS, Monteiro JCS, Camelo-Nunes IC, Solé D. Prevalence of asthma symptoms and associated factors in schoolchildren from Brazilian Amazon islands. . 2012; 49(6): 600-5.		2007-2009	Scientific literature
Asthma	Garcia-Marcos L, Mallol J, Solé D, Brand PLP, EISL Study Group. International study of wheezing in infants: risk factors in affluent and non-affluent countries during the first year of life. . 2010; 21(5): 878-88.		2005-2007	Scientific literature
Asthma	Lukrafka JL, Fuchs SC, Moreira LB, Picon RV, Fischer GB, Fuchs FD. Performance of the ISAAC questionnaire to establish the prevalence of asthma in adolescents: a population-based study. . 2010; 47(2): 166-9.		2005-2007	Scientific literature
Asthma	Brazilian Institute of Geography and Statistics (IBGE), Ministry of Health (Brazil), Ministry of Planning, Budget, and Management (Brazil). Brazil National Health Survey 2013. Rio de Janeiro, Brazil: Brazilian Institute of Geography and Statistics (IBGE).	Country	2013	Survey
Asthma	Castro LKK de, Cerci Neto A, Ferreira Filho OF. Prevalence of symptoms of asthma, rhinitis and atopic eczema among students between 6 and 7 years of age in the city of Londrina, Brazil. . 2010; 36(3): 286-92.		2008	Scientific literature
Asthma	Feitosa CA, Santos DN, Barreto do Carmo MB, Santos LM, Teles CAS, Rodrigues LC, Barreto ML. Behavior problems and prevalence of asthma symptoms among Brazilian children. . 2011; 71(3): 160-5.		2006	Scientific literature
Interstitial lung disease and pulmonary sarcoidosis	Ministry of Health (Brazil). Brazil Hospital Information System 1997. Rio de Janeiro, Brazil: Ministry of Health (Brazil).	Country	1993-1997	Administrative record
Interstitial lung disease and pulmonary sarcoidosis	Ministry of Health (Brazil). Brazil Hospital Information System 2008-2012.		2008-2012	Administrative record
Interstitial lung disease and pulmonary sarcoidosis	Ministry of Health (Brazil). Brazil Hospital Information System 1998-2002.		1998-2002	Administrative record
Interstitial lung disease and pulmonary sarcoidosis	Brazilian Institute of Geography and Statistics (IBGE), Ministry of Health (Brazil), Ministry of Planning, Budget, and Management (Brazil). Brazil National Health Survey 2013. Rio de Janeiro, Brazil: Brazilian Institute of Geography and Statistics (IBGE).	Country	2013-2014	Survey
Interstitial lung disease and pulmonary sarcoidosis	Brazil World Health Survey 2003	Country	2002-2003	Survey
Interstitial lung disease and pulmonary sarcoidosis	Ministry of Health (Brazil). Brazil Hospital Information System 2013-2014.		2013-2017	Administrative record
Interstitial lung disease and pulmonary sarcoidosis	Health Institute (São Paulo, Brazil), State University of Campinas, São Paulo Municipal Health Department, São Paulo State University, University of São Paulo. Brazil - São Paulo Health Survey 2008-2009.	São Paulo	2002-2008	Survey
Interstitial lung disease and pulmonary sarcoidosis	Ministry of Health (Brazil). Brazil Hospital Information System 2003-2007.		2003-2007	Administrative record
Cirrhosis and other chronic liver diseases	Ministry of Health (Brazil). Brazil Hospital Information System 1997. Rio de Janeiro, Brazil: Ministry of Health (Brazil).	Country	1993-1997	Administrative record
Cirrhosis and other chronic liver diseases	Health Institute (São Paulo, Brazil), State University of Campinas, São Paulo Municipal Health Department, São Paulo State University, University of São Paulo. Brazil - São Paulo Health Survey 2008-2009.	São Paulo	2002-2008	Survey
Cirrhosis and other chronic liver diseases	Ministry of Health (Brazil). Brazil Hospital Information System 2008-2012.		2008-2012	Administrative record
Cirrhosis and other chronic liver diseases	Ministry of Health (Brazil). Brazil Hospital Information System 1998-2002.		1998-2002	Administrative record
Cirrhosis and other chronic liver diseases	Brazilian Institute of Geography and Statistics (IBGE), Ministry of Health (Brazil), Ministry of Planning, Budget, and Management (Brazil). Brazil National Health Survey 2013. Rio de Janeiro, Brazil: Brazilian Institute of Geography and Statistics (IBGE).	Country	2013-2014	Survey
Cirrhosis and other chronic liver diseases	Brazil World Health Survey 2003	Country	2002-2003	Survey
Cirrhosis and other chronic liver diseases	Ministry of Health (Brazil). Brazil Hospital Information System 2013-2014.		2013-2017	Administrative record
Cirrhosis and other chronic liver diseases	Ministry of Health (Brazil). Brazil Hospital Information System 2003-2007.		2003-2007	Administrative record
Cirrhosis and other chronic liver diseases due to	Ministry of Health (Brazil). Brazil Hospital Information System 1998-2002.		1998-2002	Administrative record
Cirrhosis and other chronic liver diseases due to	Ministry of Health (Brazil). Brazil Hospital Information System 2008-2012.		2008-2012	Administrative record
Cirrhosis and other chronic liver diseases due to	Ministry of Health (Brazil). Brazil Hospital Information System 2013-2014.		2013-2017	Administrative record
Cirrhosis and other chronic liver diseases due to hepatitis B	Souza NP, Villar LM, Garbin AJ, Rovida TA, Garbin CA. Assessment of health-related quality of life and related factors in patients with chronic liver disease. . 2015; 19(6): 590-5.		2011-2012	Scientific literature
Cirrhosis and other chronic liver diseases due to	Ministry of Health (Brazil). Brazil Hospital Information System 2003-2007.		2003-2007	Administrative record
Cirrhosis and other chronic liver diseases due to hepatitis B	Ministry of Health (Brazil). Brazil Hospital Information System 1997. Rio de Janeiro, Brazil: Ministry of Health (Brazil).	Country	1993-1997	Administrative record
Cirrhosis and other chronic liver diseases due to hepatitis B	Goncalves PL, Zago-Gomes, Mda P, Marques CC, Mendona AT, Goncalves CS, Pereira FE. Etiology of liver cirrhosis in Brazil: chronic alcoholism and hepatitis viruses in liver cirrhosis diagnosed in the state of Esp?rito Santo. . 2013; 68(3): 291-5.		1993-2011	Scientific literature
Cirrhosis and other chronic liver diseases due to	Ministry of Health (Brazil). Brazil Hospital Information System 1998-2002.		1998-2002	Administrative record
Cirrhosis and other chronic liver diseases due to hepatitis C	Souza NP, Villar LM, Garbin AJ, Rovida TA, Garbin CA. Assessment of health-related quality of life and related factors in patients with chronic liver disease. . 2015; 19(6): 590-5.		2011-2012	Scientific literature
Cirrhosis and other chronic liver diseases due to hepatitis C	Ministry of Health (Brazil). Brazil Hospital Information System 1997. Rio de Janeiro, Brazil: Ministry of Health (Brazil).	Country	1993-1997	Administrative record
Cirrhosis and other chronic liver diseases due to hepatitis C	Goncalves PL, Zago-Gomes, Mda P, Marques CC, Mendona AT, Goncalves CS, Pereira FE. Etiology of liver cirrhosis in Brazil: chronic alcoholism and hepatitis viruses in liver cirrhosis diagnosed in the state of Esp?rito Santo. . 2013; 68(3): 291-5.		1993-2011	Scientific literature
Cirrhosis and other chronic liver diseases due to	Ministry of Health (Brazil). Brazil Hospital Information System 2003-2007.		2003-2007	Administrative record
Cirrhosis and other chronic liver diseases due to	Ministry of Health (Brazil). Brazil Hospital Information System 2013-2014.		2013-2017	Administrative record
Cirrhosis and other chronic liver diseases due to	Ministry of Health (Brazil). Brazil Hospital Information System 2008-2012.		2008-2012	Administrative record
Cirrhosis and other chronic liver diseases due to	Ministry of Health (Brazil). Brazil Hospital Information System 2008-2012.		2008-2012	Administrative record
Cirrhosis and other chronic liver diseases due to alcohol use	Ministry of Health (Brazil). Brazil Hospital Information System 1997. Rio de Janeiro, Brazil: Ministry of Health (Brazil).	Country	1993-1997	Administrative record
Cirrhosis and other chronic liver diseases due to	Ministry of Health (Brazil). Brazil Hospital Information System 2013-2014.		2013-2017	Administrative record
Cirrhosis and other chronic liver diseases due to	Ministry of Health (Brazil). Brazil Hospital Information System 2003-2007.		2003-2007	Administrative record
Cirrhosis and other chronic liver diseases due to	Ministry of Health (Brazil). Brazil Hospital Information System 1998-2002.		1998-2002	Administrative record
Cirrhosis and other chronic liver diseases due to alcohol use	Souza NP, Villar LM, Garbin AJ, Rovida TA, Garbin CA. Assessment of health-related quality of life and related factors in patients with chronic liver disease. . 2015; 19(6): 590-5.		2011-2012	Scientific literature
Cirrhosis and other chronic liver diseases due to alcohol use	Goncalves PL, Zago-Gomes, Mda P, Marques CC, Mendona AT, Goncalves CS, Pereira FE. Etiology of liver cirrhosis in Brazil: chronic alcoholism and hepatitis viruses in liver cirrhosis diagnosed in the state of Esp?rito Santo. . 2013; 68(3): 291-5.		1993-2011	Scientific literature
Cirrhosis and other chronic liver diseases due to	Ministry of Health (Brazil). Brazil Hospital Information System 1998-2002.		1998-2002	Administrative record
Cirrhosis and other chronic liver diseases due to other causes	Ministry of Health (Brazil). Brazil Hospital Information System 1997. Rio de Janeiro, Brazil: Ministry of Health (Brazil).	Country	1993-1997	Administrative record
Cirrhosis and other chronic liver diseases due to other causes	Souza NP, Villar LM, Garbin AJ, Rovida TA, Garbin CA. Assessment of health-related quality of life and related factors in patients with chronic liver disease. . 2015; 19(6): 590-5.		2011-2012	Scientific literature
Cirrhosis and other chronic liver diseases due to	Ministry of Health (Brazil). Brazil Hospital Information System 2013-2014.		2013-2017	Administrative record
Cirrhosis and other chronic liver diseases due to	Ministry of Health (Brazil). Brazil Hospital Information System 2003-2007.		2003-2007	Administrative record
Cirrhosis and other chronic liver diseases due to	Ministry of Health (Brazil). Brazil Hospital Information System 2008-2012.		2008-2012	Administrative record
Cirrhosis and other chronic liver diseases due to other causes	Goncalves PL, Zago-Gomes, Mda P, Marques CC, Mendona AT, Goncalves CS, Pereira FE. Etiology of liver cirrhosis in Brazil: chronic alcoholism and hepatitis viruses in liver cirrhosis diagnosed in the state of Esp?rito Santo. . 2013; 68(3): 291-5.		1993-2011	Scientific literature
Peptic ulcer disease	Health Institute (São Paulo, Brazil), State University of Campinas, São Paulo Municipal Health Department, São Paulo State University, University of São Paulo. Brazil - São Paulo Health Survey 2008-2009.	São Paulo	2002-2008	Survey
Peptic ulcer disease	Ministry of Health (Brazil). Brazil Hospital Information System 2003-2007.		2003-2007	Administrative record
Peptic ulcer disease	Ministry of Health (Brazil). Brazil Hospital Information System 1997. Rio de Janeiro, Brazil: Ministry of Health (Brazil).	Country	1993-1997	Administrative record
Peptic ulcer disease	Ministry of Health (Brazil). Brazil Hospital Information System 1998-2002.		1998-2002	Administrative record
Peptic ulcer disease	Brazilian Institute of Geography and Statistics (IBGE), Ministry of Health (Brazil), Ministry of Planning, Budget, and Management (Brazil). Brazil National Health Survey 2013. Rio de Janeiro, Brazil: Brazilian Institute of Geography and Statistics (IBGE).	Country	2013-2014	Survey
Peptic ulcer disease	Brazil World Health Survey 2003	Country	2002-2003	Survey
Peptic ulcer disease	Ministry of Health (Brazil). Brazil Hospital Information System 2008-2012.		2008-2012	Administrative record
Peptic ulcer disease	Ministry of Health (Brazil). Brazil Hospital Information System 2013-2014.		2013-2017	Administrative record
Gastritis and duodenitis	Ministry of Health (Brazil). Brazil Hospital Information System 2003-2007.		2003-2007	Administrative record
Gastritis and duodenitis	Ministry of Health (Brazil). Brazil Hospital Information System 1997. Rio de Janeiro, Brazil: Ministry of Health (Brazil).	Country	1993-1997	Administrative record
Gastritis and duodenitis	Ministry of Health (Brazil). Brazil Hospital Information System 2013-2014.		2013-2017	Administrative record

Gastritis and duodenitis	Brazilian Institute of Geography and Statistics (IBGE), Ministry of Health (Brazil), Ministry of Planning, Budget, and Management (Brazil). Brazil National Health Survey 2013. Rio de Janeiro, Brazil: Brazilian Institute of Geography and Statistics (IBGE).	Country	2013-2014	Survey
Gastritis and duodenitis	Ministry of Health (Brazil). Brazil Hospital Information System 2008-2012.		2008-2012	Administrative record
Gastritis and duodenitis	Brazil World Health Survey 2003	Country	2002-2003	Survey
Gastritis and duodenitis	Ministry of Health (Brazil). Brazil Hospital Information System 1998-2002.		1998-2002	Administrative record
Gastritis and duodenitis	Health Institute (São Paulo, Brazil), State University of Campinas, São Paulo Municipal Health Department, São Paulo State University, University of São Paulo. Brazil - São Paulo Health Survey 2008-2009.	São Paulo	2002-2008	Survey
Appendicitis	Ministry of Health (Brazil). Brazil Hospital Information System 2008-2012.		2008-2012	Administrative record
Appendicitis	Health Institute (São Paulo, Brazil), State University of Campinas, São Paulo Municipal Health Department, São Paulo State University, University of São Paulo. Brazil - São Paulo Health Survey 2008-2009.	São Paulo	2002-2008	Survey
Appendicitis	Ministry of Health (Brazil). Brazil Hospital Information System 2003-2007.		2003-2007	Administrative record
Appendicitis	Ministry of Health (Brazil). Brazil Hospital Information System 1998-2002.		1998-2002	Administrative record
Appendicitis	Ministry of Health (Brazil). Brazil Hospital Information System 2013-2014.		2013-2017	Administrative record
Appendicitis	Brazilian Institute of Geography and Statistics (IBGE), Ministry of Health (Brazil), Ministry of Planning, Budget, and Management (Brazil). Brazil National Health Survey 2013. Rio de Janeiro, Brazil: Brazilian Institute of Geography and Statistics (IBGE).	Country	2013-2014	Survey
Appendicitis	Ministry of Health (Brazil). Brazil Hospital Information System 1997. Rio de Janeiro, Brazil: Ministry of Health (Brazil).	Country	1993-1997	Administrative record
Appendicitis	Brazil World Health Survey 2003	Country	2002-2003	Survey
Paralytic ileus and intestinal obstruction	Ministry of Health (Brazil). Brazil Hospital Information System 2008-2012.		2008-2012	Administrative record
Paralytic ileus and intestinal obstruction	Ministry of Health (Brazil). Brazil Hospital Information System 1998-2002.		1998-2002	Administrative record
Paralytic ileus and intestinal obstruction	Brazilian Institute of Geography and Statistics (IBGE), Ministry of Health (Brazil), Ministry of Planning, Budget, and Management (Brazil). Brazil National Health Survey 2013. Rio de Janeiro, Brazil: Brazilian Institute of Geography and Statistics (IBGE).	Country	2013-2014	Survey
Paralytic ileus and intestinal obstruction	Ministry of Health (Brazil). Brazil Hospital Information System 2013-2014.		2013-2017	Administrative record
Paralytic ileus and intestinal obstruction	Brazil World Health Survey 2003	Country	2002-2003	Survey
Paralytic ileus and intestinal obstruction	Ministry of Health (Brazil). Brazil Hospital Information System 1997. Rio de Janeiro, Brazil: Ministry of Health (Brazil).	Country	1993-1997	Administrative record
Paralytic ileus and intestinal obstruction	Ministry of Health (Brazil). Brazil Hospital Information System 2003-2007.		2003-2007	Administrative record
Paralytic ileus and intestinal obstruction	Health Institute (São Paulo, Brazil), State University of Campinas, São Paulo Municipal Health Department, São Paulo State University, University of São Paulo. Brazil - São Paulo Health Survey 2008-2009.	São Paulo	2002-2008	Survey
Inguinal, femoral, and abdominal hernia	Ministry of Health (Brazil). Brazil Hospital Information System 2013-2014.		2013-2017	Administrative record
Inguinal, femoral, and abdominal hernia	Ministry of Health (Brazil). Brazil Hospital Information System 1997. Rio de Janeiro, Brazil: Ministry of Health (Brazil).	Country	1993-1997	Administrative record
Inguinal, femoral, and abdominal hernia	Ministry of Health (Brazil). Brazil Hospital Information System 1998-2002.		1998-2002	Administrative record
Inguinal, femoral, and abdominal hernia	Ministry of Health (Brazil). Brazil Hospital Information System 2003-2007.		2003-2007	Administrative record
Inguinal, femoral, and abdominal hernia	Brazilian Institute of Geography and Statistics (IBGE), Ministry of Health (Brazil), Ministry of Planning, Budget, and Management (Brazil). Brazil National Health Survey 2013. Rio de Janeiro, Brazil: Brazilian Institute of Geography and Statistics (IBGE).	Country	2013-2014	Survey
Inguinal, femoral, and abdominal hernia	Ministry of Health (Brazil). Brazil Hospital Information System 2008-2012.		2008-2012	Administrative record
Inguinal, femoral, and abdominal hernia	Health Institute (São Paulo, Brazil), State University of Campinas, São Paulo Municipal Health Department, São Paulo State University, University of São Paulo. Brazil - São Paulo Health Survey 2008-2009.	São Paulo	2002-2008	Survey
Inguinal, femoral, and abdominal hernia	Brazil World Health Survey 2003	Country	2002-2003	Survey
Inflammatory bowel disease	Brazil World Health Survey 2003	Country	2002-2003	Survey
Inflammatory bowel disease	Ministry of Health (Brazil). Brazil Hospital Information System 1997. Rio de Janeiro, Brazil: Ministry of Health (Brazil).	Country	1993-1997	Administrative record
Inflammatory bowel disease	Ministry of Health (Brazil). Brazil Hospital Information System 2013-2014.		2013-2017	Administrative record
Inflammatory bowel disease	Ministry of Health (Brazil). Brazil Hospital Information System 2003-2007.		2003-2007	Administrative record
Inflammatory bowel disease	Health Institute (São Paulo, Brazil), State University of Campinas, São Paulo Municipal Health Department, São Paulo State University, University of São Paulo. Brazil - São Paulo Health Survey 2008-2009.	São Paulo	2002-2008	Survey
Inflammatory bowel disease	Brazilian Institute of Geography and Statistics (IBGE), Ministry of Health (Brazil), Ministry of Planning, Budget, and Management (Brazil). Brazil National Health Survey 2013. Rio de Janeiro, Brazil: Brazilian Institute of Geography and Statistics (IBGE).	Country	2013-2014	Survey
Inflammatory bowel disease	Ministry of Health (Brazil). Brazil Hospital Information System 1998-2002.		1998-2002	Administrative record
Inflammatory bowel disease	Victoria CR, Sassak LY, Nunes HR de C. Incidence and prevalence rates of inflammatory bowel diseases, in midwestern of São Paulo State, Brazil. . 2009; 46(1): 20-5.		1986-2005	Scientific literature
Inflammatory bowel disease	Ministry of Health (Brazil). Brazil Hospital Information System 2008-2012.		2008-2012	Administrative record
Vascular intestinal disorders	Health Institute (São Paulo, Brazil), State University of Campinas, São Paulo Municipal Health Department, São Paulo State University, University of São Paulo. Brazil - São Paulo Health Survey 2008-2009.	São Paulo	2002-2008	Survey
Vascular intestinal disorders	Ministry of Health (Brazil). Brazil Hospital Information System 1998-2002.		1998-2002	Administrative record
Vascular intestinal disorders	Ministry of Health (Brazil). Brazil Hospital Information System 2003-2007.		2003-2007	Administrative record
Vascular intestinal disorders	Brazilian Institute of Geography and Statistics (IBGE), Ministry of Health (Brazil), Ministry of Planning, Budget, and Management (Brazil). Brazil National Health Survey 2013. Rio de Janeiro, Brazil: Brazilian Institute of Geography and Statistics (IBGE).	Country	2013-2014	Survey
Vascular intestinal disorders	Ministry of Health (Brazil). Brazil Hospital Information System 2008-2012.		2008-2012	Administrative record
Vascular intestinal disorders	Ministry of Health (Brazil). Brazil Hospital Information System 2013-2014.		2013-2017	Administrative record
Vascular intestinal disorders	Brazil World Health Survey 2003	Country	2002-2003	Survey
Vascular intestinal disorders	Ministry of Health (Brazil). Brazil Hospital Information System 1997. Rio de Janeiro, Brazil: Ministry of Health (Brazil).	Country	1993-1997	Administrative record
Gallbladder and biliary diseases	Ministry of Health (Brazil). Brazil Hospital Information System 1997. Rio de Janeiro, Brazil: Ministry of Health (Brazil).	Country	1993-1997	Administrative record
Gallbladder and biliary diseases	Ministry of Health (Brazil). Brazil Hospital Information System 2008-2012.		2008-2012	Administrative record
Gallbladder and biliary diseases	Brazil World Health Survey 2003	Country	2002-2003	Survey
Gallbladder and biliary diseases	Health Institute (São Paulo, Brazil), State University of Campinas, São Paulo Municipal Health Department, São Paulo State University, University of São Paulo. Brazil - São Paulo Health Survey 2008-2009.	São Paulo	2002-2008	Survey
Gallbladder and biliary diseases	Ministry of Health (Brazil). Brazil Hospital Information System 2003-2007.		2003-2007	Administrative record
Gallbladder and biliary diseases	Ministry of Health (Brazil). Brazil Hospital Information System 1998-2002.		1998-2002	Administrative record
Gallbladder and biliary diseases	Brazilian Institute of Geography and Statistics (IBGE), Ministry of Health (Brazil), Ministry of Planning, Budget, and Management (Brazil). Brazil National Health Survey 2013. Rio de Janeiro, Brazil: Brazilian Institute of Geography and Statistics (IBGE).	Country	2013-2014	Survey
Gallbladder and biliary diseases	Ministry of Health (Brazil). Brazil Hospital Information System 2013-2014.		2013-2017	Administrative record
Pancreatitis	Ministry of Health (Brazil). Brazil Hospital Information System 2008-2012.		2008-2012	Administrative record
Pancreatitis	Ministry of Health (Brazil). Brazil Hospital Information System 2013-2014.		2013-2017	Administrative record
Pancreatitis	Ministry of Health (Brazil). Brazil Hospital Information System 1997. Rio de Janeiro, Brazil: Ministry of Health (Brazil).	Country	1993-1997	Administrative record
Pancreatitis	Health Institute (São Paulo, Brazil), State University of Campinas, São Paulo Municipal Health Department, São Paulo State University, University of São Paulo. Brazil - São Paulo Health Survey 2008-2009.	São Paulo	2002-2008	Survey
Pancreatitis	Ministry of Health (Brazil). Brazil Hospital Information System 2003-2007.		2003-2007	Administrative record
Pancreatitis	Brazilian Institute of Geography and Statistics (IBGE), Ministry of Health (Brazil), Ministry of Planning, Budget, and Management (Brazil). Brazil National Health Survey 2013. Rio de Janeiro, Brazil: Brazilian Institute of Geography and Statistics (IBGE).	Country	2013-2014	Survey
Pancreatitis	Ministry of Health (Brazil). Brazil Hospital Information System 1998-2002.		1998-2002	Administrative record
Pancreatitis	Brazil World Health Survey 2003	Country	2002-2003	Survey
Alzheimer disease and other dementias	Bottino CMC, Azevedo D Jr, Tatsch M, Hototian SR, Moscoso MA, Folquitto J, Scalco AZ, Bazzarella MC, Lopes MA, Litvoc J. Estimate of dementia prevalence in a community sample from São Paulo, Brazil. . 2008; 26(4): 291-9.		2002-2003	Scientific literature
Alzheimer disease and other dementias	Scazufca M, Menezes PR, Vallada HP, Crepaldi AL, Pastor-Valero M, Coutinho LMS, Di Rienzo VD, Almeida OP. High prevalence of dementia among older adults from poor socioeconomic backgrounds in São Paulo, Brazil. . 2008; 20(2): 394-405.		2003-2005	Scientific literature
Alzheimer disease and other dementias	Cesar KG, Brucki SM, Takada LT, Nascimento LF, Gomes CM, Almeida MC, Oliveira MO, Porto FH, Senaha ML, Bahia VS, Silva TB, Ianof JN, Spindola L, Schmidt MT, Jorge MS, Vale PH, Cecchini MA, Cassimiro L, Soares RT, Goncalves MR, Martins AC, Dare P, Smid J, Porto CS, Carthery-Goulart MT, Yassuda MS, Mansur LL, Nitrini R. Prevalence of Cognitive Impairment Without Dementia and Dementia in Tremembe, Brazil. . 2016; 30(3): 264-71.		2013-2015	Scientific literature
Alzheimer disease and other dementias	Institute for Health Metrics and Evaluation (IHME). IHME DisMod Alzheimer Disease and Other Dementias Excess Mortality Estimates.	Global	1990-2016	Modeled data

Alzheimer disease and other dementias	Fichman H, Porto CS, Carthery MT, Hartmann APJ, Huang N, Smid J, Lima EP, Takada LT, Takahashi DY. Incidence of dementia in a community-dwelling Brazilian population. . 2004; 18(4): 241-6.		1997-2000	Scientific literature
Alzheimer disease and other dementias	Herrera E Jr, Caramelli P, Silveira ASB, Nitrini R. Epidemiologic survey of dementia in a community-dwelling Brazilian population. . 2002; 16(2): 103-8.		1997-1998	Scientific literature
Alzheimer disease and other dementias	Lopes MA, Ferrioli E, Nakano EY, Litvoc J, Bottino CMC. High prevalence of dementia in a community-based survey of older people from Brazil: association with intellectual activity rather than education. . 2012; 32(2): 307-16.		2009-2011	Scientific literature
Parkinson disease	Institute for Health Metrics and Evaluation (IHME). IHME DisMod Parkinson's Disease Excess Mortality Estimates.	Global	1990-2016	Modeled data
Parkinson disease	Munhoz RP, Teive HA, Eleftherohorinou H, Coin LJ, Lees AJ, Silveira-Moriyama L. Demographic and motor features associated with the occurrence of neuropsychiatric and sleep complications of Parkinson's disease. . 2013; 84(8): 883-7.		0	Scientific literature
Epilepsy	Noronha ALA, Borges MA, Marques LHN, Zanetta DMT, Fernandes PT, de Boer H, Espíndola J, Miranda CT, Prilipko L, Bell GS, Sander JW, Li LM. Prevalence and pattern of epilepsy treatment in different socioeconomic classes in Brazil. . 2007; 48(5): 880-5.		2002	Scientific literature
Epilepsy	Gomes Md M da M, Zeitoune RG, Kropf LAL, Beeck Ed E da S van. A house-to-house survey of epileptic seizures in an urban community of Rio de Janeiro, Brazil. . 2002; 60(3-B): 708-11.		2000-2001	Scientific literature
Epilepsy	Fernandes J, Schmidt M, Monte T, Tozzi S, Sander J. Prevalence of epilepsy: the Porto Alegre Study. . 1992; 33(Suppl 3): 132.		1992	Scientific literature
Epilepsy	factors in children under five living in a deprived municipality of southern Brazil. . 2007; 65(3A): 581-6.		1998-1999	Scientific literature
Epilepsy	Marino Junior R, Cukiert A, Pinho E. Aspectos epidemiologicos da epilepsia em Sao Paulo: um estudo da prevalencia. . 1986; 44(3): 243-54.		1984-1985	Scientific literature
Epilepsy	Li LM, Fernandes PT, Noronha ALA, Marques LHN, Borges MA, Cendes F, Guerreiro CAM, Zanetta DMT, de Boer HM, Espíndola J, Miranda CT, Prilipko L, Sander JW. Demonstration Project on Epilepsy in Brazil: situation assessment. . 2007; 65(Supp 1): 5-13.		2007	Scientific literature
Epilepsy	Arruda WO. Etiology of epilepsy. A prospective study of 210 cases. . 1991; 49(3): 251�4.	Country	1991	Scientific literature
Epilepsy	Nunes ML, Geib LTC, Grupo Apego. Incidence of epilepsy and seizure disorders in childhood and association with social determinants: a birth cohort study. . 2011; 87(1): 50-6.		2003-2007	Scientific literature
Epilepsy	Valença MM, Valença LP. [Etiology of the epileptic seizures in Recife city, Brazil: study of 249 patients]. . 2000; 58(4): 1064�72.	Country	1996-1999	Scientific literature
Epilepsy	Borges MA, Barros EP de, Zanetta DMT, Borges APP. [Prevalence of epilepsy in Bakairi indians from Mato Grosso State, Brazil]. . 2002; 60(1): 80-5.		2000	Scientific literature
Multiple sclerosis	Arruda WO, Scola RH, Teive HA, Werneck LC. Multiple sclerosis: report on 200 cases from Curitiba, Southern Brazil and comparison with other Brazilian series. . 2001; 59(2-A): 165-70.		0	Scientific literature
Multiple sclerosis	Callegaro D, de Lolio CA, Radvany J, Tilbery CP, Mendonça RA, Melo AC. Prevalence of multiple sclerosis in the city of São Paulo, Brazil, in 1990. . 1992; 11(1): 11-4.		1990	Scientific literature
Multiple sclerosis	Lana-Peixoto MA, Frota ERC, Campos GB, Monteiro LP, Brazilian Committee for Treatment and Research in Multiple Sclerosis. The prevalence of multiple sclerosis in Belo Horizonte, Brazil. . 2012; 70(2): 102-7.		0-2001	Scientific literature
Multiple sclerosis	Callegaro D, Goldbaum M, Morais L, Tilbery CP, Moreira MA, Gabbai AA, Scaff M. The prevalence of multiple sclerosis in the city of São Paulo, Brazil, 1997. . 2001; 104(4): 208-13.		1997	Scientific literature
Multiple sclerosis	da Silva NL, Takemoto ML, Damasceno A, Fragoso YD, Finkelsztejn A, Becker J, Goncalves MV, Tilbery C, de Oliveira EM, Callegaro D, Boulos FC. Cost analysis of multiple sclerosis in Brazil: a cross-sectional multicenter study. . 2016; 16.0: 102.		0	Scientific literature
Motor neuron disease	amyotrophic lateral sclerosis in the city of Porto Alegre, in Southern Brazil. . 2013; 71(12): 959�62.		2010	Scientific literature
Migraine	Queiroz LP, Barea LM, Blank N. An epidemiological study of headache in Florianopolis, Brazil. . 2006; 26(2): 122-7.		1982-2000	Scientific literature
Migraine	Benseñor IM, Goulart AC, Lotufo PA, Menezes PR, Scazufca M. Cardiovascular risk factors associated with migraine among the elderly with a low income: the Sao Paulo Ageing and Health Study (SPAH). . 2011; 31(3): 331-7.		2003-2005	Scientific literature
Migraine	Wiehe M, Fuchs SC, Moreira LB, Moraes RS, Fuchs FD. Migraine is more frequent in individuals with optimal and normal blood pressure: a population-based study. . 2002; 20(7): 1303-6.		1996-1998	Scientific literature
Migraine	Garrido J, Macias-Islas M, Monzillo P, Nunez L, Plascencia N, Rodriguez C, Takeuchi Y, Latin American Migraine Study Group. Prevalence of migraine in Latin America. . 2005; 45(2): 106-17.		1999	Scientific literature
Migraine	Bensen~or IM, Lotufo PA, Goulart AC, Menezes PR, Scazufca M. The prevalence of headache among elderly in a low-income area of São Paulo, Brazil. . 2008; 28(4): 329-33.		2003-2005	Scientific literature
Migraine	Da Silva A Jr, Costa EC, Gomes JB, Leite FM, Gomez RS, Vasconcelos LP, Krymchantowski A, Moreira P, Teixeira AL. Chronic headache and comorbidities: a two-phase, population-based, cross-sectional study. . 2010; 50(8): 1306-12.		2005-2006	Scientific literature
Migraine	Arruda MA, Guidetti V, Galli F, Albuquerque RCAP, Bigal ME. Primary headaches in childhood � a population-based study. . 2010; 30(9): 1056-64.		2009	Scientific literature
Migraine	Arruda MA, Bigal ME. Behavioral and emotional symptoms and primary headaches in children: a population-based study. . 2012; 32(15): 1093-100.		2009	Scientific literature
Migraine	Falavigna A, Teles AR, Velho MC, Vedana VM, Silva RC da, Mazzocchin T, Basso M, Braga GL de. Prevalence and impact of headache in undergraduate students in Southern Brazil. . 2010; 68(6): 873-7.		2009	Scientific literature
Tension-type headache	Falavigna A, Teles AR, Velho MC, Vedana VM, Silva RC da, Mazzocchin T, Basso M, Braga GL de. Prevalence and impact of headache in undergraduate students in Southern Brazil. . 2010; 68(6): 873-7.		2009	Scientific literature
Tension-type headache	Wiehe M, Fuchs SC, Moreira LB, Moraes RS, Fuchs FD. Migraine is more frequent in individuals with optimal and normal blood pressure: a population-based study. . 2002; 20(7): 1303-6.		1996-1998	Scientific literature
Tension-type headache	Bensen~or IM, Lotufo PA, Goulart AC, Menezes PR, Scazufca M. The prevalence of headache among elderly in a low-income area of São Paulo, Brazil. . 2008; 28(4): 329-33.		2003-2005	Scientific literature
Tension-type headache	Da Silva A Jr, Costa EC, Gomes JB, Leite FM, Gomez RS, Vasconcelos LP, Krymchantowski A, Moreira P, Teixeira AL. Chronic headache and comorbidities: a two-phase, population-based, cross-sectional study. . 2010; 50(8): 1306-12.		2005-2006	Scientific literature
Tension-type headache	Queiroz LP, Peres MFP, Piovesan EJ, Kowacs F, Ciciarelli MC, Souza JA, Zukerman E. A nationwide population-based study of tension-type headache in Brazil. . 2009; 49(1): 71-8.		2006-2007	Scientific literature
Tension-type headache	Arruda MA, Bigal ME. Behavioral and emotional symptoms and primary headaches in children: a population-based study. . 2012; 32(15): 1093-100.		2009	Scientific literature
Tension-type headache	de Siqueira SR, Vilela TT, Florindo AA. Prevalence of headache and orofacial pain in adults and elders in a Brazilian community: an epidemiological study. . 2015; 32(2): 123-31.		2011-2012	Scientific literature
Tension-type headache	Ferreira KDS, Speciali JG. Epidemiology of chronic pain in the office of a pain specialist neurologist. . 2015; 73(7): 582�5.		2008	Scientific literature
Alcohol use disorders	RA, de Mello MF, Prince M, Ferri CP, Coutinho ES, Andreoli SB. The impact of epidemic violence on the prevalence of psychiatric disorders in Sao Paulo and Rio de Janeiro, Brazil. . 2013; 8(5): e63545.		2007-2008	Scientific literature
Alcohol use disorders	Coelho CLS, Laranjeira RR, Santos JLF, Pinsky I, Zaleski M, Caetano R, Crippa JAS. Depressive symptoms and alcohol correlates among Brazilians aged 14 years and older: a cross-sectional study. . 2014; 9: 29.		2005-2006	Scientific literature
Alcohol use disorders	Ipsos, National Institute of Public Policy for Alcohol and Other Drugs (INPAD) (Brazil), University of São Paulo. Brazil National Alcohol and Drugs Survey 2011-2012.	Country	2011-2012	Survey
Alcohol use disorders	National Institute of Public Policy for Alcohol and Other Drugs (INPAD) (Brazil). Brazil National Alcohol Survey 2005-2006.	Country	2005-2006	Survey
Alcohol use disorders	World Health Organization (WHO). Mental Illness in General Health Care: An International Study. Geneva, Switzerland: World Health Organization (WHO), 1995.	Country	1991-1992	Report
Alcohol use disorders	Grinfeld H, Goldenberg S, Segre CA, Chadi G. Fetal alcohol syndrome in São Paulo, Brazil. . 1999; 13(4): 496-7.		1997	Scientific literature
Alcohol use disorders	Ferreira LN, Bispo Junior JP, Sales ZN, Casotti CA, Braga Junior ACR. [Prevalence and associated factors of alcohol abuse and alcohol addiction]. . 2013; 18(11): 3409�18.		2010	Scientific literature
Alcohol use disorders	Ministry of Health (Brazil). Brazil Live Birth Information System SINASC 2007.	Country	2007	Vital registration
Alcohol use disorders	Ministry of Health (Brazil). Brazil Live Birth Information System SINASC 2006. Rio de Janeiro, Brazil: Ministry of Health (Brazil).	Country	2006	Vital registration
Alcohol use disorders	Barros MB, Marín-Leó L, Oliveira HB, Dalgalarrondo P, Botega JN. Alcohol drinking patterns: social and demographic differences in the municipality of Campinas, state of São Paulo, Brazil, 2003. . 2008; 17(4): 259-70.		2003	Scientific literature
Alcohol use disorders	Silveira CM, Siu ER, Anthony JC, Saito LP, de Andrade AG, Kutschenko A, Viana MC, Wang Y-P, Martins SS, Andrade LH. Drinking patterns and alcohol use disorders in São Paulo, Brazil: the role of neighborhood social deprivation and socioeconomic status. . 2014; 9(10): e108355.		2005-2007	Scientific literature
Alcohol use disorders	Barros MB de A, Botega NJ, Dalgalarrondo P, Marín-León L, de Oliveira HB. Prevalence of alcohol abuse and associated factors in a population-based study. . 2007; 41(4): 502-9.		2003	Scientific literature
Cocaine use disorders	Ribeiro M, Dunn J, Sesso R, Dias AC, Laranjeira R. Causes of death among crack cocaine users. . 2006; 28(3): 196-202.		1992-1999	Scientific literature

Cocaine use disorders	Galduróz JCF, Noto AR, Nappo SA, Carlini EA. Household survey on drug abuse in Brazil: study involving the 107 major cities of the country �2001. . 2005; 30(3): 545-56.		2001	Scientific literature
Cocaine use disorders	Dias AC, Ribeiro M, Dunn J, Sesso R, Laranjeira R. Follow-up study of crack cocaine users: situation of the patients after 2, 5, and 12 years. . 2008; 29(3): 71-9.		1995-2006	Scientific literature
Cocaine use disorders	Ipsos, National Institute of Public Policy for Alcohol and Other Drugs (INPAD) (Brazil), University of São Paulo. Brazil National Alcohol and Drugs Survey 2011-2012.	Country	2011-2012	Survey
Cocaine use disorders	Brazil Household Survey About the Use of Psychotropic Drugs 2005	Country	2005	Survey
Cocaine use disorders	use in Brazil: data from the II Brazilian national alcohol and drugs survey (BNADS). . 2014; 39(1): 297�301.		2011-2012	Scientific literature
Cocaine use disorders	Bitancourt T, Tissot MCRG, Fidalgo TM, Galduróz JCF, da Silveira Filho DX. Factors associated with illicit drugs�lifetime and frequent/heavy use among students results from a population survey. . 2016; 237: 290�.		2009	Scientific literature
Cocaine use disorders	Madruga CS, Laranjeira R, Caetano R, Pinsky I, Zaleski M, Ferri CP. Use of licit and illicit substances among adolescents in Brazil--a national survey. . 2012; 37(10): 1171�.		2009	Scientific literature
Amphetamine use disorders	Galduróz JCF, Noto AR, Nappo SA, Carlini EA. Household survey on drug abuse in Brazil: study involving the 107 major cities of the country �2001. . 2005; 30(3): 545-56.		2001	Scientific literature
Amphetamine use disorders	Galduróz JCF, Noto AR, Nappo SA, Carlini EA. Trends in drug use among students in Brazil: analysis of four surveys in 1987, 1989, 1993 and 1997. . 2004; 37(4): 523-31.		1993-1997	Scientific literature
Amphetamine use disorders	Bitancourt T, Tissot MCRG, Fidalgo TM, Galduróz JCF, da Silveira Filho DX. Factors associated with illicit drugs�lifetime and frequent/heavy use among students results from a population survey. . 2016; 237: 290�.		2009	Scientific literature
Amphetamine use disorders	Inter-American Drug Abuse Control Commission (CICAD), Organization of American States (OAS), National Commission for Development and Life without Drugs (DEVIDA) (Peru), National Council for Narcotics Control (CONACE) (Chile), National Council for the Control of Narcotic and Psychotropic Substances (CONSEP), National Drug Board (JND), Secretariat for Programming Drug Abuse Prevention and the Fight against Drug Trafficking (SEDRONAR), The National Council for the Fight against Illicit Drug Trafficking (CONALTID), United Nations Office on Drugs and Crime (UNODC). Youth and Drugs in South American Countries: A Public Policy Challenge. Washington, D.C., United States: Inter-American Drug Abuse Control Commission (CICAD), Organization of American States (OAS), 2006.	Country	2006-2007	Report
Cannabis use disorders	Ipsos, National Institute of Public Policy for Alcohol and Other Drugs (INPAD) (Brazil), University of São Paulo. Brazil National Alcohol and Drugs Survey 2011-2012.	Country	2011-2012	Survey
Cannabis use disorders	Bitancourt T, Tissot MCRG, Fidalgo TM, Galduróz JCF, da Silveira Filho DX. Factors associated with illicit drugs�lifetime and frequent/heavy use among students results from a population survey. . 2016; 237: 290�.		2009	Scientific literature
Cannabis use disorders	Brazil Household Survey About the Use of Psychotropic Drugs 2005	Country	2005	Survey
Cannabis use disorders	Inter-American Drug Abuse Control Commission (CICAD), Organization of American States (OAS), National Commission for Development and Life without Drugs (DEVIDA) (Peru), National Council for Narcotics Control (CONACE) (Chile), National Council for the Control of Narcotic and Psychotropic Substances (CONSEP), National Drug Board (JND), Secretariat for Programming Drug Abuse Prevention and the Fight against Drug Trafficking (SEDRONAR), The National Council for the Fight against Illicit Drug Trafficking (CONALTID), United Nations Office on Drugs and Crime (UNODC). Youth and Drugs in South American Countries: A Public Policy Challenge. Washington, D.C., United States: Inter-American Drug Abuse Control Commission (CICAD), Organization of American States (OAS), 2006.	Country	2005	Report
Cannabis use disorders	cannabis use in Brazil: data from the I Brazilian National Alcohol Survey (BNAS). . 2010; 35(3): 190-3.		2005-2006	Scientific literature
Cannabis use disorders	on the Epidemiology of Mental Disorders. Cambridge, United Kingdom: Cambridge University Press, 2008.	Country	2004-2006	Report
Cannabis use disorders	Madruga CS, Laranjeira R, Caetano R, Pinsky I, Zaleski M, Ferri CP. Use of licit and illicit substances among adolescents in Brazil--a national survey. . 2012; 37(10): 1171�.		2009	Scientific literature
Major depressive disorder	Brazilian Institute of Geography and Statistics (IBGE), Ministry of Health (Brazil), Ministry of Planning, Budget, and Management (Brazil). Brazil National Health Survey 2013. Rio de Janeiro, Brazil: Brazilian Institute of Geography and Statistics (IBGE).	Country	2013	Survey
Major depressive disorder	Anselmi L, Fleitlich-Bilyk B, Menezes AMB, Araújo CL, Rohde LA. Prevalence of psychiatric disorders in a Brazilian birth cohort of 11-year-olds. . 2010; 45(1): 135-42.		2004-2006	Scientific literature
Major depressive disorder	Andrade LH, Wang Y-P, Andreoni S, Silveira CM, Alexandrino-Silva C, Siu ER, Nishimura R, Anthony JC, Gattaz WF, Kessler RC, Viana MC. Mental disorders in megacities: findings from the São Paulo megacity mental health survey, Brazil. . 2012; 7(2): e31879.		2005-2007	Scientific literature
Major depressive disorder	Institute for Health Metrics and Evaluation (IHME). IHME Cause-Specific Mortality Rates for Suicide Attributable to Major Depressive Disorder.	Global	1990-2015	Modeled data
Major depressive disorder	Costa E, Barreto SM, Uchoa E, Firmo JOA, Lima-Costa MF, Prince M. Prevalence of International Classification of Diseases, 10th Revision Common Mental Disorders in the Elderly in a Brazilian Community: The Bambui Health Ageing Study. . 2007; 15(1): 17-27.		1997-2001	Scientific literature
Major depressive disorder	Petresco S, Anselmi L, Santos IS, Barros AJ, Fleitlich-Bilyk B, Barros FC, Matijasevich A. Prevalence and comorbidity of psychiatric disorders among 6-year-old children: 2004 Pelotas Birth Cohort. . 2014; 49(6): 975�.		2010	Scientific literature
Major depressive disorder	RA, de Mello MF, Prince M, Ferri CP, Coutinho ES, Andreoli SB. The impact of epidemic violence on the prevalence of psychiatric disorders in Sao Paulo and Rio de Janeiro, Brazil. . 2013; 8(5): e63545.		2007-2008	Scientific literature
Major depressive disorder	Bahls S. Epidemiology of depressive symptoms in adolescents of a public school in Curitiba, Brazil. . 2002; 63�.		1999-2001	Scientific literature
Major depressive disorder	da Silva SA, Scazufca M, Menezes PR. Population impact of depression on functional disability in elderly: results from �São Paulo Ageing & Health Study�(SPAH). . 2013; 263(2): 153�.		2003-2005	Scientific literature
Major depressive disorder	Barcelos-Ferreira R, Nakano EY, Steffens DC, Bottino CM. Quality of life and physical activity associated to lower prevalence of depression in community-dwelling elderly subjects from Sao Paulo. . 2013; 150(2): 616�.		2002-2004	Scientific literature
Major depressive disorder	Brazil World Health Survey 2003	Country	2003	Survey
Major depressive disorder	Coelho CL, Crippa JA, Santos JL, Pinsky I, Zaleski M, Caetano R, Laranjeira R. Higher prevalence of major depressive symptoms in Brazilians aged 14 and older. . 2013; 35(2): 142�.		2005-2006	Scientific literature
Major depressive disorder	Vorcaro CM, Lima-Costa MF, Barreto SM, Uchoa E. Unexpected high prevalence of 1-month depression in a small Brazilian community: the Bambui Study. . 2001; 104(4): 257�.		1996-1997	Scientific literature
Major depressive disorder	Lopez MRA, Ribeiro JP, Ores LC, Jansen K, Souza LDM, Pinheiro RT, Da Silva RA. Depressao e qualidade de vida em jovens de 18 a 24 anos no sul do Brasil. . 2011; 103�.		2007-2008	Scientific literature
Major depressive disorder	Chiavegatto Filho AD, Kawachi I, Wang YP, Viana MC, Andrade LH. Does income inequality get under the skin? A multilevel analysis of depression, anxiety and mental disorders in Sao Paulo, Brazil. . 2013; 966�.		2005-2007	Scientific literature
Major depressive disorder	Andrade L, Walters EE, Gentil V, Laurenti R. Prevalence of ICD-10 mental disorders in a catchment area in the city of São Paulo, Brazil. . 2002; 37(7): 316-25.		1994-1996	Scientific literature
Major depressive disorder	Kessler RC, Birnbaum HG, Shahly V, Bromet E, Hwang I, McLaughlin KA, Sampson N, Andrade LH, de Girolamo G, Demyttenaere K, Haro JM, Karam AN, Kostyuchenko S, Kovess V, Lara C, Levinson D, Matschinger H, Nakane Y, Browne MO, Ormel J, Posada-Villa J, Sagar R, Stein DJ. Age differences in the prevalence and co-morbidity of DSM-IV major depressive episodes: results from the WHO World Mental Health Survey Initiative. . 2010; 27(4): 351�.		2004-2007	Scientific literature
Major depressive disorder	Salum GA, Gadelha A, Pan PM, Moriyama TS, Graeff-Martins AS, Tamanaha AC, Alvarenga P, Valle Krieger F, Fleitlich-Bilyk B, Jackowski A, Sato JR, Brietzke E, Polanczyk GV, Brentani H, de Jesus Mari J, Do Rosário MC, Manfro GG, Bressan RA, Mercadante MT, Miguel EC, Rohde LA. High risk cohort study for psychiatric disorders in childhood: rationale, design, methods and preliminary results. . 2015; 24(1): 58�.		2012-2014	Scientific literature
Major depressive disorder	Fleitlich-Bilyk B, Goodman R. Prevalence of child and adolescent psychiatric disorders in southeast Brazil. . 2004; 43(6): 727-34.		2000-2001	Scientific literature
Dysthymia	Barcelos-Ferreira R, Nakano EY, Steffens DC, Bottino CM. Quality of life and physical activity associated to lower prevalence of depression in community-dwelling elderly subjects from Sao Paulo. . 2013; 150(2): 616�.		2002-2004	Scientific literature
Dysthymia	Andrade L, Walters EE, Gentil V, Laurenti R. Prevalence of ICD-10 mental disorders in a catchment area in the city of São Paulo, Brazil. . 2002; 37(7): 316-25.		1994-1996	Scientific literature
Dysthymia	Costa E, Barreto SM, Uchoa E, Firmo JOA, Lima-Costa MF, Prince M. Prevalence of International Classification of Diseases, 10th Revision Common Mental Disorders in the Elderly in a Brazilian Community: The Bambui Health Ageing Study. . 2007; 15(1): 17-27.		1997-2001	Scientific literature
Bipolar disorder	Merikangas KR, Jin R, He J-P, Kessler RC, Lee S, Sampson NA, Viana MC, Andrade LH, Hu C, Karam EG, Ladea M, Medina-Mora ME, Ono Y, Posada-Villa J, Sagar R, Wells JE, Zarkov Z. Prevalence and correlates of bipolar spectrum disorder in the World Mental Health Survey Initiative. . 2011; 68(3): 241-51.		2005-2007	Scientific literature

Bipolar disorder	Salum GA, Gadelha A, Pan PM, Moriyama TS, Graeff-Martins AS, Tamanaha AC, Alvarenga P, Valle Krieger F, Fleitlich-Bilyk B, Jackowski A, Sato JR, Brietzke E, Polanczyk GV, Brentani H, de Jesus Mari J, Do Rosário MC, Manfro GG, Bressan RA, Mercadante MT, Miguel EC, Rohde LA. High risk cohort study for psychiatric disorders in childhood: rationale, design, methods and preliminary results. . 2015; 24(1): 5873.		2012-2014	Scientific literature
Bipolar disorder	Andrade L, Walters EE, Gentil V, Laurenti R. Prevalence of ICD-10 mental disorders in a catchment area in the city of São Paulo, Brazil. . 2002; 37(7): 316-25.		1994-1996	Scientific literature
Anxiety disorders	Anselmi L, Fleitlich-Bilyk B, Menezes AMB, Araújo CL, Rohde LA. Prevalence of psychiatric disorders in a Brazilian birth cohort of 11-year-olds. . 2010; 45(1): 135-42.		2004-2005	Scientific literature
Anxiety disorders	Jaen-Varas D, Mari J de J, Coutinho E da S, Andreoli SB, Quintana MI, de Mello MF, Bressan RA, Ribeiro WS. A cross-sectional study to compare levels of psychiatric morbidity between young people and adults exposed to violence in a large urban center. . 2016; 16: 134.		2007-2008	Scientific literature
Anxiety disorders	Silva RA da. Anxiety disorders in young people: a population-based study. . 2013; 35(4): 347-52.		2007-2009	Scientific literature
Anxiety disorders	Petresco S, Anselmi L, Santos IS, Barros AJ, Fleitlich-Bilyk B, Barros FC, Matijasevich A. Prevalence and comorbidity of psychiatric disorders among 6-year-old children: 2004 Pelotas Birth Cohort. . 2014; 49(6): 97583.		2010	Scientific literature
Anxiety disorders	Andrade L, Walters EE, Gentil V, Laurenti R. Prevalence of ICD-10 mental disorders in a catchment area in the city of São Paulo, Brazil. . 2002; 37(7): 316-25.		1994-1996	Scientific literature
Anxiety disorders	Fleitlich-Bilyk B, Goodman R. Prevalence of child and adolescent psychiatric disorders in southeast Brazil. . 2004; 43(6): 727-34.		2000-2001	Scientific literature
Anxiety disorders	on the Epidemiology of Mental Disorders. Cambridge, United Kingdom: Cambridge University Press, 2008.	Country	2005-2007	Report
Bulimia nervosa	Kessler RC, Berglund PA, Chiu WT, Deitz AC, Hudson JI, Shahly V, Aguilar-Gaxiola S, Alonso J, Angermeyer MC, Benjet C, Bruffaerts R, de Girolamo G, de Graaf R, Haro JM, Kovess-Masfety V, ONeill S, Posada-Villa J, Sasu C, Scott K, Viana MC, Xavier M. The prevalence and correlates of binge eating disorder in the WHO World Mental Health Surveys. . 2013; 73(9): 904914.		2005-2007	Scientific literature
Bulimia nervosa	Andrade L, Walters EE, Gentil V, Laurenti R. Prevalence of ICD-10 mental disorders in a catchment area in the city of São Paulo, Brazil. . 2002; 37(7): 316-25.		1994-1996	Scientific literature
Autism	Salum GA, Gadelha A, Pan PM, Moriyama TS, Graeff-Martins AS, Tamanaha AC, Alvarenga P, Valle Krieger F, Fleitlich-Bilyk B, Jackowski A, Sato JR, Brietzke E, Polanczyk GV, Brentani H, de Jesus Mari J, Do Rosário MC, Manfro GG, Bressan RA, Mercadante MT, Miguel EC, Rohde LA. High risk cohort study for psychiatric disorders in childhood: rationale, design, methods and preliminary results. . 2015; 24(1): 5873.		2012-2014	Scientific literature
Autism	Institute for Health Metrics and Evaluation (IHME). IHME DisMod Autism Excess Mortality	Global	1990-2016	Modeled data
Autism	Petresco S, Anselmi L, Santos IS, Barros AJ, Fleitlich-Bilyk B, Barros FC, Matijasevich A. Prevalence and comorbidity of psychiatric disorders among 6-year-old children: 2004 Pelotas Birth Cohort. . 2014; 49(6): 97583.		2010	Scientific literature
Asperger syndrome and other autistic spectrum disorders	Salum GA, Gadelha A, Pan PM, Moriyama TS, Graeff-Martins AS, Tamanaha AC, Alvarenga P, Valle Krieger F, Fleitlich-Bilyk B, Jackowski A, Sato JR, Brietzke E, Polanczyk GV, Brentani H, de Jesus Mari J, Do Rosário MC, Manfro GG, Bressan RA, Mercadante MT, Miguel EC, Rohde LA. High risk cohort study for psychiatric disorders in childhood: rationale, design, methods and preliminary results. . 2015; 24(1): 5873.		2012-2014	Scientific literature
Attention-deficit/hyperactivity disorder	Goodman R, Neves dos Santos D, Robatto Nunes AP, Pereira de Miranda D, Fleitlich-Bilyk B, Almeida Filho N. The Ilha de Maré study: a survey of child mental health problems in a predominantly African-Brazilian rural community. . 2005; 40(1): 11-7.		2003	Scientific literature
Attention-deficit/hyperactivity disorder	Petresco S, Anselmi L, Santos IS, Barros AJ, Fleitlich-Bilyk B, Barros FC, Matijasevich A. Prevalence and comorbidity of psychiatric disorders among 6-year-old children: 2004 Pelotas Birth Cohort. . 2014; 49(6): 97583.		2010	Scientific literature
Attention-deficit/hyperactivity disorder	Salum GA, Gadelha A, Pan PM, Moriyama TS, Graeff-Martins AS, Tamanaha AC, Alvarenga P, Valle Krieger F, Fleitlich-Bilyk B, Jackowski A, Sato JR, Brietzke E, Polanczyk GV, Brentani H, de Jesus Mari J, Do Rosário MC, Manfro GG, Bressan RA, Mercadante MT, Miguel EC, Rohde LA. High risk cohort study for psychiatric disorders in childhood: rationale, design, methods and preliminary results. . 2015; 24(1): 5873.		2012-2014	Scientific literature
Attention-deficit/hyperactivity disorder	Rohde LA, Biederman J, Busnello EA, Zimmermann H, Schmitz M, Martins S, Tramontina S. ADHD in a School Sample of Brazilian Adolescents: A Study of Prevalence, Comorbid Conditions, and Impairments. . 1999; 38(6): 716-22.		1997	Scientific literature
Attention-deficit/hyperactivity disorder	Fleitlich-Bilyk B, Goodman R. Prevalence of child and adolescent psychiatric disorders in southeast Brazil. . 2004; 43(6): 727-34.		2002	Scientific literature
Attention-deficit/hyperactivity disorder	Matte B, Anselmi L, Salum GA, Kieling C, Gonçalves H, Menezes A, Grevet EH, Rohde LA. ADHD in DSM-5: A field trial in a large, representative sample of 18- to 19- year-old adults. . 2015; 45(2): 36173.		2011-2012	Scientific literature
Attention-deficit/hyperactivity disorder	Arruda MA, Querido CN, Bigal ME, Polanczyk GV. ADHD and mental health status in Brazilian school-age children. . 2015; 19(1): 117.		2009	Scientific literature
Attention-deficit/hyperactivity disorder	Anselmi L, Fleitlich-Bilyk B, Menezes AMB, Araújo CL, Rohde LA. Prevalence of psychiatric disorders in a Brazilian birth cohort of 11-year-olds. . 2010; 45(1): 135-42.		2004-2006	Scientific literature
Conduct disorder	Goodman R, Neves dos Santos D, Robatto Nunes AP, Pereira de Miranda D, Fleitlich-Bilyk B, Almeida Filho N. The Ilha de Maré study: a survey of child mental health problems in a predominantly African-Brazilian rural community. . 2005; 40(1): 11-7.		2001	Scientific literature
Conduct disorder	Anselmi L, Fleitlich-Bilyk B, Menezes AMB, Araújo CL, Rohde LA. Prevalence of psychiatric disorders in a Brazilian birth cohort of 11-year-olds. . 2010; 45(1): 135-42.		2004-2006	Scientific literature
Conduct disorder	Petresco S, Anselmi L, Santos IS, Barros AJ, Fleitlich-Bilyk B, Barros FC, Matijasevich A. Prevalence and comorbidity of psychiatric disorders among 6-year-old children: 2004 Pelotas Birth Cohort. . 2014; 49(6): 97583.		2010	Scientific literature
Conduct disorder	Salum GA, Gadelha A, Pan PM, Moriyama TS, Graeff-Martins AS, Tamanaha AC, Alvarenga P, Valle Krieger F, Fleitlich-Bilyk B, Jackowski A, Sato JR, Brietzke E, Polanczyk GV, Brentani H, de Jesus Mari J, Do Rosário MC, Manfro GG, Bressan RA, Mercadante MT, Miguel EC, Rohde LA. High risk cohort study for psychiatric disorders in childhood: rationale, design, methods and preliminary results. . 2015; 24(1): 5873.		2012-2014	Scientific literature
Conduct disorder	Fleitlich-Bilyk B, Goodman R. Prevalence of child and adolescent psychiatric disorders in southeast Brazil. . 2004; 43(6): 727-34.		2002	Scientific literature
Idiopathic developmental intellectual disability	Stein Z, Belmont L, Durkin M. Mild mental retardation and severe mental retardation compared: experiences in eight less developed countries. . 1987; 44: 89-96.		1987	Scientific literature
Diabetes mellitus	Muniz EC, Rocha RM, Reis ML, Santos VL, Grossi SA. Neuropathic and ischemic changes of the foot in Brazilian patients with diabetes. . 2003; 49(8): 60-70.		2000-2002	Scientific literature
Diabetes mellitus	Brazilian Institute of Geography and Statistics (IBGE), Ministry of Health (Brazil), Ministry of Planning, Budget, and Management (Brazil). Brazil National Health Survey 2013. Rio de Janeiro, Brazil: Brazilian Institute of Geography and Statistics (IBGE).	Country	2013	Survey
Diabetes mellitus	Schaan BD, Harzheim E, Gus I. [Cardiac risk profile in diabetes mellitus and impaired fasting glucose]. . 2004; 38(4): 529-36.		1999-2000	Scientific literature
Diabetes mellitus	Freitas MPD, Loyola Filho AI de, Lima-Costa MF. Birth cohort differences in cardiovascular risk factors in a Brazilian population of older elderly: the Bambuí Cohort Study of Aging (1997 and 2008). . 2011; S409-417.		1997-2008	Scientific literature
Diabetes mellitus	Lima-Costa MF, Mambrini JV, Leite ML, Peixoto SV, Firmo JO, Loyola Filho AI, Gouveia MH, Leal TP, Pereira AC, Macinko J, Tarazona-Santos E. Socioeconomic Position, But Not African Genomic Ancestry, Is Associated With Blood Pressure in the Bambui-Epigen (Brazil) Cohort Study of Aging. . 2016; 67(2): 349-55.		1997	Scientific literature
Diabetes mellitus	Rigo JC, Vieira JL, Dalacorte RR, Reichert CL. Prevalence of metabolic syndrome in an elderly community: comparison between three diagnostic methods. . 2009; 93(2): 85-91.		2005-2006	Scientific literature
Diabetes mellitus	Vieira-Santos IC, Souza WV, Carvalho EF, Medeiros MC, Nóbrega MG, Lima PM. Prevalence of diabetic foot and associated factors in the family health units of the city of Recife, Pernambuco State, Brazil, in 2005. . 2008; 24(12): 2861-70.		2006	Scientific literature
Diabetes mellitus	Arieta CEL, de Oliveira DF, Lupinacci AP de C, Novaes P, Paccola M, Jose NK, Limburg H. Cataract remains an important cause of blindness in Campinas, Brazil. . 2009; 16(1): 58-63.		2003	Scientific literature
Diabetes mellitus	Araújo Filho A, Salomão SR, Berezovsky A, Cinoto RW, Morales PHA, Santos FRG, Belfort R Jr. Prevalence of visual impairment, blindness, ocular disorders and cataract surgery outcomes in low-income elderly from a metropolitan region of São Paulo-Brazil. . 2008; 71(2): 246-53.		2002	Scientific literature
Diabetes mellitus	Lessa I, Magalhães L, Araújo MJ, de Almeida Filho N, Aquino E, Oliveira MM. Arterial hypertension in the adult population of Salvador (BA)--Brazil. . 2006; 87(6): 747-56.		1999-2000	Scientific literature
Diabetes mellitus	International Centre for Eye Health (ICEH). Brazil - Campinas Rapid Assessment of Avoidable Blindness 2004. Grootebroek, Netherlands: RAAB Repository.	São Paulo	2003	Survey
Acute glomerulonephritis	Health Institute (São Paulo, Brazil), State University of Campinas, São Paulo Municipal Health Department, São Paulo State University, University of São Paulo. Brazil - São Paulo Health Survey 2008-2009.	São Paulo	2002-2008	Survey
Acute glomerulonephritis	Ministry of Health (Brazil). Brazil Hospital Information System 1998-2002.		1998-2002	Administrative record
Acute glomerulonephritis	Ministry of Health (Brazil). Brazil Hospital Information System 2003-2007.		2003-2007	Administrative record

Acute glomerulonephritis	Brazilian Institute of Geography and Statistics (IBGE), Ministry of Health (Brazil), Ministry of Planning, Budget, and Management (Brazil). Brazil National Health Survey 2013. Rio de Janeiro, Brazil: Brazilian Institute of Geography and Statistics (IBGE).	Country	2013-2014	Survey
Acute glomerulonephritis	Brazil World Health Survey 2003	Country	2002-2003	Survey
Acute glomerulonephritis	Ministry of Health (Brazil). Brazil Hospital Information System 1997. Rio de Janeiro, Brazil: Ministry of Health (Brazil).	Country	1993-1997	Administrative record
Acute glomerulonephritis	Ministry of Health (Brazil). Brazil Hospital Information System 2013-2014.		2013-2017	Administrative record
Acute glomerulonephritis	Ministry of Health (Brazil). Brazil Hospital Information System 2008-2012.		2008-2012	Administrative record
Chronic kidney disease	Institute for Health Metrics and Evaluation (IHME). IHME DisMod Stage IV Chronic Kidney Disease Remission Estimates.	Global	1990-2016	Modeled data
Chronic kidney disease	Brazilian Society of Nephrology. Brazilian Society of Nephrology Dialysis Census 2009. São Paulo, Brazil: Brazilian Society of Nephrology.	Country	2009	Disease registry
Chronic kidney disease	Brazilian Society of Nephrology. Brazilian Society of Nephrology Dialysis Census 2011. São Paulo, Brazil: Brazilian Society of Nephrology.	Country	2011	Disease registry
Chronic kidney disease	Brazilian Society of Nephrology. Brazilian Society of Nephrology Dialysis Census 2012. São Paulo, Brazil: Brazilian Society of Nephrology.	Country	2012	Disease registry
Chronic kidney disease	Brazilian Society of Nephrology. Brazilian Society of Nephrology Dialysis Census 2013. São Paulo, Brazil: Brazilian Society of Nephrology.	Country	2013	Disease registry
Chronic kidney disease	De Moura L, Prestes IV, Duncan BB, Thome FS, Schmidt MI. Dialysis for end stage renal disease financed through the Brazilian National Health System, 2000 to 2012. . 2014; 111.		2001-2003	Scientific literature
Chronic kidney disease	Vidigal PG, Ribeiro AL, Lotufo PA, Mill JG. Chronic kidney disease among adult participants of the ELSA-Brasil cohort: association with race and socioeconomic position. . 2016; 70(4): 380-9.		2008-2010	Scientific literature
Chronic kidney disease	Cordeiro AC, Carrero JJ, Qureshi AR, Cunha RF da, Lindholm B, Castro I de, Noronha IL. Study of the incidence of dialysis in Sao Paulo, the largest Brazilian city. . 2013; 68(6): 7605.		2007-2011	Scientific literature
Chronic kidney disease	Brazilian Society of Nephrology. Brazilian Society of Nephrology Dialysis Census 2014. São Paulo, Brazil: Brazilian Society of Nephrology.	Country	2014	Disease registry
Chronic kidney disease	Prevalence of chronic renal disease in adults attended by the family health strategy. . 2016; 38(1): 22-30.		2011-2013	Scientific literature
Chronic kidney disease	Institute for Health Metrics and Evaluation (IHME). IHME DisMod Stage III Chronic Kidney Disease Remission Estimates.	Global	1990-2016	Modeled data
Chronic kidney disease	Brazilian Society of Nephrology. Brazilian Society of Nephrology Dialysis Census 2008. São Paulo, Brazil: Brazilian Society of Nephrology.	Country	2007	Disease registry
Chronic kidney disease	Cusumano AM, Gonzalez Bedat MC, Garcia-Garcia G, Maury Fernandez S, Lugon JR, Poblete Badal H, Elgueta Miranda S, Gomez R, Cerdas Calderon M, Almaguer Lopez M, Moscoso Tobar J, Leiva Merino R, Sanchez Polo J, Lou Meda R, Franco Acosta B, Ayala Ferrari R, Escudero E, Saavedra Lopez A, Mena Castro E, Milanes C, Carlini R, Duro Garcia V. Latin American Dialysis and Renal Transplant Registry: 2008 report (data 2006). . 2010; 74 Suppl 1: S38.		2006	Scientific literature
Chronic kidney disease	Pecoits-Filho R, Rosa-Diez G, Gonzalez-Bedat M, Marinovich S, Fernandez S, Lugon J, Poblete-Badal H, Elgueta-Miranda S, Gomez R, Cerdas-Calderon M, Almaguer-Lopez M, Freire N, Leiva-Merino R, Rodriguez G, Luna-Guerra J, Bochicchio T, Garcia-Garcia G, Cano N, Iron N. Renal replacement therapy in CKD: an update from the Latin American Registry of Dialysis and Transplantation. . 2015; 37(1): 913.		2010	Scientific literature
Chronic kidney disease	Institute for Health Metrics and Evaluation (IHME). IHME GBD End-stage Renal Disease DisMod Transplant Incidence Estimates.	Global	1990-2016	Modeled data
Chronic kidney disease	Cusumano AM, Di Gioia C, Hermida O, Lavorato C, Latin American Registry of Dialysis and Renal Transplantation. The Latin American Dialysis and Renal Transplantation Registry Annual Report 2002. . 2005; (97): S462.		2000-2001	Scientific literature
Chronic kidney disease	Cusumano A, Garcia Garcia G, Gonzalez Bedat C. The Latin American Dialysis and Transplant Registry: report 2006. . 2009; 19(1 Suppl 1): S13 6.		2005	Scientific literature
Chronic kidney disease	United States Renal Data System Annual Data Report 2015	Country	2003-2013	Disease registry
Chronic kidney disease	Brazilian Society of Nephrology. Brazilian Society of Nephrology Dialysis Census 2007.	Country	2006-2007	Disease registry
Chronic kidney disease	Brazilian Society of Nephrology. Brazilian Society of Nephrology Dialysis Census 2002. São Paulo, Brazil: Brazilian Society of Nephrology.	Country	2002	Disease registry
Chronic kidney disease	Brazilian Society of Nephrology. Brazilian Society of Nephrology Dialysis Census 2005. São Paulo, Brazil: Brazilian Society of Nephrology.	Country	2005	Disease registry
Chronic kidney disease	Brazilian Society of Nephrology. Brazilian Society of Nephrology Dialysis Census 2006. São Paulo, Brazil: Brazilian Society of Nephrology.	Country	2003-2006	Disease registry
Chronic kidney disease	Ferna´ndez-Cean J, Gonza´lez-Marti´nez F, Schwedt E, Mazzuchi N. Renal replacement therapy in Latin America. . 2000; 57(s74): 55-59.		1997	Scientific literature
Chronic kidney disease	Cusumano A, Garcia-Garcia G, Di Gioia C, Hermida O, Lavorato C, Carreño CA, Torrico MP, Batista PB, Romão JE, Badal HP, Miranda SE, Gomez R, Calderon MC, Sanchez SH, Lopez MA, Moscoso J, Merino RL, Polo JV, Lopez A, Romero NJ, Garcia R, Acosta BV, Lopez AS, Delpin ES, Mena E, González C, Milanés CL, Acchiardo S. End-stage renal disease and its treatment in Latin America in the twenty-first century. . 2006; 28(8): 631-7.	Country	2004	Scientific literature
Chronic kidney disease	Santiago-Delpín EA, García VD. Latin American Transplant Registry VIIIth Report: 1998. . 1999; 31(1-2): 214-6.		1995-1997	Scientific literature
Chronic kidney disease	transplants in Brazil: report of the Brazilian Registry of Renal Transplantation. . 1990; 5(11): 956-61.		1987-1989	Scientific literature
Chronic kidney disease	Brazilian Society of Nephrology. Brazilian Society of Nephrology Dialysis Census 2000. São Paulo, Brazil: Brazilian Society of Nephrology.	Country	2000	Disease registry
Chronic kidney disease	Brazilian Society of Nephrology. Brazilian Society of Nephrology Dialysis Census 2001. São Paulo, Brazil: Brazilian Society of Nephrology.	Country	2001	Disease registry
Chronic kidney disease	Brazilian Society of Nephrology. Brazilian Society of Nephrology Dialysis Census 2004. São Paulo, Brazil: Brazilian Society of Nephrology.	Country	1999-2004	Disease registry
Chronic kidney disease	Brazilian Society of Nephrology. Brazilian Society of Nephrology Dialysis Census 1999. São Paulo, Brazil: Brazilian Society of Nephrology.	Country	1999	Disease registry
Interstitial nephritis and urinary tract infections	Health Institute (São Paulo, Brazil), State University of Campinas, São Paulo Municipal Health Department, São Paulo State University, University of São Paulo. Brazil - São Paulo Health Survey 2008-2009.	São Paulo	2002-2008	Survey
Interstitial nephritis and urinary tract infections	Ministry of Health (Brazil). Brazil Hospital Information System 2013-2014.		2008-2017	Administrative record
Interstitial nephritis and urinary tract infections	Brazil World Health Survey 2003	Country	2002-2003	Survey
Interstitial nephritis and urinary tract infections	Ministry of Health (Brazil). Brazil Hospital Information System 1998-2002.		1998-2007	Administrative record
Interstitial nephritis and urinary tract infections	Ministry of Health (Brazil). Brazil Hospital Information System 2008-2012.		2008-2017	Administrative record
Interstitial nephritis and urinary tract infections	Ministry of Health (Brazil). Brazil Hospital Information System 2003-2007.		1998-2007	Administrative record
Interstitial nephritis and urinary tract infections	Brazilian Institute of Geography and Statistics (IBGE), Ministry of Health (Brazil), Ministry of Planning, Budget, and Management (Brazil). Brazil National Health Survey 2013. Rio de Janeiro, Brazil: Brazilian Institute of Geography and Statistics (IBGE).	Country	2013-2014	Survey
Interstitial nephritis and urinary tract infections	Ministry of Health (Brazil). Brazil Hospital Information System 1997. Rio de Janeiro, Brazil: Ministry of Health (Brazil).	Country	1993-1997	Administrative record
Urolithiasis	Ministry of Health (Brazil). Brazil Hospital Information System 2013-2014.		2013-2017	Administrative record
Urolithiasis	Ministry of Health (Brazil). Brazil Hospital Information System 2008-2012.		2008-2012	Administrative record
Urolithiasis	Ministry of Health (Brazil). Brazil Hospital Information System 2003-2007.		2003-2007	Administrative record
Urolithiasis	Brazil World Health Survey 2003	Country	2002-2003	Survey
Urolithiasis	Ministry of Health (Brazil). Brazil Hospital Information System 1997. Rio de Janeiro, Brazil: Ministry of Health (Brazil).	Country	1993-1997	Administrative record
Urolithiasis	Health Institute (São Paulo, Brazil), State University of Campinas, São Paulo Municipal Health Department, São Paulo State University, University of São Paulo. Brazil - São Paulo Health Survey 2008-2009.	São Paulo	2002-2008	Survey
Urolithiasis	Ministry of Health (Brazil). Brazil Hospital Information System 1998-2002.		1998-2002	Administrative record
Urolithiasis	Brazilian Institute of Geography and Statistics (IBGE), Ministry of Health (Brazil), Ministry of Planning, Budget, and Management (Brazil). Brazil National Health Survey 2013. Rio de Janeiro, Brazil: Brazilian Institute of Geography and Statistics (IBGE).	Country	2013-2014	Survey
Benign prostatic hyperplasia	Ministry of Health (Brazil). Brazil Hospital Information System 1997. Rio de Janeiro, Brazil: Ministry of Health (Brazil).	Country	1993-1997	Administrative record
Benign prostatic hyperplasia	Ministry of Health (Brazil). Brazil Hospital Information System 2013-2014.		2013-2017	Administrative record
Benign prostatic hyperplasia	Brazil World Health Survey 2003	Country	2002-2003	Survey
Benign prostatic hyperplasia	Brazilian Institute of Geography and Statistics (IBGE), Ministry of Health (Brazil), Ministry of Planning, Budget, and Management (Brazil). Brazil National Health Survey 2013. Rio de Janeiro, Brazil: Brazilian Institute of Geography and Statistics (IBGE).	Country	2013-2014	Survey
Benign prostatic hyperplasia	Ministry of Health (Brazil). Brazil Hospital Information System 2003-2007.		2003-2007	Administrative record
Benign prostatic hyperplasia	Health Institute (São Paulo, Brazil), State University of Campinas, São Paulo Municipal Health Department, São Paulo State University, University of São Paulo. Brazil - São Paulo Health Survey 2008-2009.	São Paulo	2002-2008	Survey
Benign prostatic hyperplasia	Ministry of Health (Brazil). Brazil Hospital Information System 2008-2012.		2008-2012	Administrative record
Benign prostatic hyperplasia	Ministry of Health (Brazil). Brazil Hospital Information System 1998-2002.		1998-2002	Administrative record
Gynecological diseases	Ministry of Health (Brazil). Brazil Hospital Information System 2008-2012.		2008-2012	Administrative record

Gynecological diseases	Ministry of Health (Brazil). Brazil Hospital Information System 1997. Rio de Janeiro, Brazil: Ministry of Health (Brazil).	Country	1993-1997	Administrative record
Gynecological diseases	Ministry of Health (Brazil). Brazil Hospital Information System 1998-2002.		1998-2002	Administrative record
Gynecological diseases	Ministry of Health (Brazil). Brazil Hospital Information System 2003-2007.		2003-2007	Administrative record
Gynecological diseases	Ministry of Health (Brazil). Brazil Hospital Information System 2013-2014.		2013-2017	Administrative record
Uterine fibroids	Ministry of Health (Brazil). Brazil Hospital Information System 2013-2014.		2013-2017	Administrative record
Uterine fibroids	Ministry of Health (Brazil). Brazil Hospital Information System 1997. Rio de Janeiro, Brazil: Ministry of Health (Brazil).	Country	1993-1997	Administrative record
Uterine fibroids	Ministry of Health (Brazil). Brazil Hospital Information System 1998-2002.		1998-2002	Administrative record
Uterine fibroids	Zimmermann A, Bernuit D, Gerlinger C, Schaefers M, Geppert K. Prevalence, symptoms and management of uterine fibroids: an international internet-based survey of 21,746 women. . 2012; 12: 6.		2009	Scientific literature
Uterine fibroids	Brazilian Institute of Geography and Statistics (IBGE), Ministry of Health (Brazil), Ministry of Planning, Budget, and Management (Brazil). Brazil National Health Survey 2013. Rio de Janeiro, Brazil: Brazilian Institute of Geography and Statistics (IBGE).	Country	2013-2014	Survey
Uterine fibroids	Ministry of Health (Brazil). Brazil Hospital Information System 2008-2012.		2008-2012	Administrative record
Uterine fibroids	Ministry of Health (Brazil). Brazil Hospital Information System 2003-2007.		2003-2007	Administrative record
Uterine fibroids	Brazil World Health Survey 2003	Country	2002-2003	Survey
Uterine fibroids	Health Institute (São Paulo, Brazil), State University of Campinas, São Paulo Municipal Health Department, São Paulo State University, University of São Paulo. Brazil - São Paulo Health Survey 2008-2009.	São Paulo	2002-2008	Survey
Polycystic ovarian syndrome	Ministry of Health (Brazil). Brazil Hospital Information System 2013-2014.		2013-2017	Administrative record
Polycystic ovarian syndrome	Brazil World Health Survey 2003	Country	2002-2003	Survey
Polycystic ovarian syndrome	Melo AS, Vieira CS, Barbieri MA, Rosa-E-Silva ACJS, Silva AAM, Cardoso VC, Reis RM, Ferriani RA, Silva-de-Sá MF, Bettiol H. High prevalence of polycystic ovary syndrome in women born small for gestational age. . 2010; 25(8): 2124-31.		2007-2008	Scientific literature
Polycystic ovarian syndrome	Ministry of Health (Brazil). Brazil Hospital Information System 2008-2012.		2008-2012	Administrative record
Polycystic ovarian syndrome	Health Institute (São Paulo, Brazil), State University of Campinas, São Paulo Municipal Health Department, São Paulo State University, University of São Paulo. Brazil - São Paulo Health Survey 2008-2009.	São Paulo	2002-2008	Survey
Polycystic ovarian syndrome	Ministry of Health (Brazil). Brazil Hospital Information System 1997. Rio de Janeiro, Brazil: Ministry of Health (Brazil).	Country	1993-1997	Administrative record
Polycystic ovarian syndrome	Brazilian Institute of Geography and Statistics (IBGE), Ministry of Health (Brazil), Ministry of Planning, Budget, and Management (Brazil). Brazil National Health Survey 2013. Rio de Janeiro, Brazil: Brazilian Institute of Geography and Statistics (IBGE).	Country	2013-2014	Survey
Polycystic ovarian syndrome	Ministry of Health (Brazil). Brazil Hospital Information System 1998-2002.		1998-2002	Administrative record
Polycystic ovarian syndrome	Ministry of Health (Brazil). Brazil Hospital Information System 2003-2007.		2003-2007	Administrative record
Endometriosis	Ministry of Health (Brazil). Brazil Hospital Information System 2008-2012.		2008-2012	Administrative record
Endometriosis	Ministry of Health (Brazil). Brazil Hospital Information System 1998-2002.		1998-2002	Administrative record
Endometriosis	Health Institute (São Paulo, Brazil), State University of Campinas, São Paulo Municipal Health Department, São Paulo State University, University of São Paulo. Brazil - São Paulo Health Survey 2008-2009.	São Paulo	2002-2008	Survey
Endometriosis	Ministry of Health (Brazil). Brazil Hospital Information System 2003-2007.		2003-2007	Administrative record
Endometriosis	Ministry of Health (Brazil). Brazil Hospital Information System 2013-2014.		2013-2017	Administrative record
Endometriosis	Ministry of Health (Brazil). Brazil Hospital Information System 1997. Rio de Janeiro, Brazil: Ministry of Health (Brazil).	Country	1993-1997	Administrative record
Endometriosis	Brazil World Health Survey 2003	Country	2002-2003	Survey
Endometriosis	Brazilian Institute of Geography and Statistics (IBGE), Ministry of Health (Brazil), Ministry of Planning, Budget, and Management (Brazil). Brazil National Health Survey 2013. Rio de Janeiro, Brazil: Brazilian Institute of Geography and Statistics (IBGE).	Country	2013-2014	Survey
Genital prolapse	Ministry of Health (Brazil). Brazil Hospital Information System 1998-2002.		1998-2002	Administrative record
Genital prolapse	Ministry of Health (Brazil). Brazil Hospital Information System 2013-2014.		2013-2017	Administrative record
Genital prolapse	Brazilian Institute of Geography and Statistics (IBGE), Ministry of Health (Brazil), Ministry of Planning, Budget, and Management (Brazil). Brazil National Health Survey 2013. Rio de Janeiro, Brazil: Brazilian Institute of Geography and Statistics (IBGE).	Country	2013-2014	Survey
Genital prolapse	Health Institute (São Paulo, Brazil), State University of Campinas, São Paulo Municipal Health Department, São Paulo State University, University of São Paulo. Brazil - São Paulo Health Survey 2008-2009.	São Paulo	2002-2008	Survey
Genital prolapse	Araujo MP, Girãao, Manoel Joãao Batista Castello, Girãao, Manoel Joãao Batista Castello, Sartori, Marair Gracio Ferreira. Pelvic floor disorders among indigenous women living in Xingu Indian Park, Brazil. . 2009; 20(9): 1079-84.		2006	Scientific literature
Genital prolapse	Ministry of Health (Brazil). Brazil Hospital Information System 2003-2007.		2003-2007	Administrative record
Genital prolapse	Ministry of Health (Brazil). Brazil Hospital Information System 2008-2012.		2008-2012	Administrative record
Genital prolapse	Ministry of Health (Brazil). Brazil Hospital Information System 1997. Rio de Janeiro, Brazil: Ministry of Health (Brazil).	Country	1993-1997	Administrative record
Genital prolapse	Brazil World Health Survey 2003	Country	2002-2003	Survey
Premenstrual syndrome	Silva CML da, Gigante DP, Carret MLV, Fassa AG. Population study of premenstrual syndrome. . 2006; 40(1): 47-56.		2003	Scientific literature
Premenstrual syndrome	Heinemann LAJ, Minh TD, Filonenko A, Uhl-Hochgräber K. Explorative evaluation of the impact of severe premenstrual disorders on work absenteeism and productivity. . 2010; 20(1): 58-65.		2007	Scientific literature
Premenstrual syndrome	Silva CML da, Gigante DP, Minten GC. Premenstrual symptoms and syndrome according to age at menarche in a 1982 birth cohort in southern Brazil. . 2008; 24(4): 835-44.		2004-2005	Scientific literature
Thalassemias	Melo-Reis PR de, Naoum PC, Diniz-Filho JAF, Dias-Penna KGB, Mesquita MM de, Balestra FA, Ternes YMF, Mascarenhas C do C, Chen LC. Prevalence of thalassemias and variant hemoglobins in the state of Goiás, Brazil. . 2006; 42(6): 425[illegible]0.		2003-2004	Scientific literature
Thalassemias	Carlos AM, Souza RA, Souza BM, Pereira Gde A, Tostes Junior S, Martins PR, Moraes-Souza H. Hemoglobinopathies in newborns in the southern region of the Triangulo Mineiro, Brazil. Cross-sectional study. . 2015; 133(5): 439-44.		2011-2013	Scientific literature
Thalassemias	Wagner SC, de Castro SM, Gonzalez TP, Santin AP, Zaleski CF, Azevedo LA, Dreau H, Henderson S, Old J, Hutz MH. Neonatal screening for hemoglobinopathies: results of a public health system in South Brazil. . 2010; 14(4): 565-9.		2004-2007	Scientific literature
Thalassemias	SM. Newborn screening program for hemoglobinopathies in Rio de Janeiro, Brazil. . 2014; 61(1): 34-9.		2000-2010	Scientific literature
Thalassemias	Sickle Cell and Thalassemias Prevalence Data, Personal Correspondence with David		1995-2010	Estimate
Thalassemias	Zago MA, Costa FF, Tone LG, Bottura C. Hereditary hemoglobin disorders in a Brazilian population. . 1983; 33(2): 125-9.		1979-1982	Scientific literature
Sickle cell disorders	Watanabe AM, Pianovski MAD, Zanis Neto J, Lichtvan LCL, Chautard-Freire-Maia EA, Domingos MT, Wittig EO. [Prevalence of hemoglobin S in the State of Parana, Brazil, based on neonatal screening]. . 2008; 24(5): 993[illegible]000.		2002-2004	Scientific literature
Sickle cell disorders	Paixao MC, Cunha Ferraz MH, Januario JN, Viana MB, Lima JM. Reliability of isoelectrofocusing for the detection of Hb S, Hb C, and HB D in a pioneering population-based program of newborn screening in Brazil. . 2001; 25(3): 297[illegible]303.		1998	Scientific literature
Sickle cell disorders	SM. Newborn screening program for hemoglobinopathies in Rio de Janeiro, Brazil. . 2014; 61(1): 34-9.		2000-2010	Scientific literature
Sickle cell disorders	Naoum PC, Angulo I de L, Brandao AC, Graciano RA, Spir M, Nomura E, Anjos ID. [Detection and awareness of patients with hemoglobinopathies in the regions of Sao Jose do Rio Preto and Presidente Prudente, SP (Brazil)]. . 1985; 19(4): 364[illegible]73.		1983-1984	Scientific literature
Sickle cell disorders	Pinheiro LS, Gonçalves RP, Tomé CAS, Alcântara AEE, Marques ARC, Silva MM da. Prevalência de hemoglobina S em recém-nascidos de Fortaleza: importância da investigação neonatal. . 2006; 28(2): 122-5.		2001-2002	Scientific literature
Sickle cell disorders	Azevêdo ES, Alves AF, Da Silva MC, Souza MG, Muniz Dias Lima AM, Azevedo WC. Distribution of abnormal hemoglobins and glucose-6-phosphate dehydrogenase variants in 1200 school children of Bahia, Brazil. . 1980; 53(4): 509-12.		1977-1979	Scientific literature
Sickle cell disorders	Fonseca GHH, Souza R, Salemi VMC, Jardim CVP, Gualandro SFM. Pulmonary hypertension diagnosed by right heart catheterisation in sickle cell disease. . 2012; 39(1): 112-8.		2007-2010	Scientific literature
Sickle cell disorders	with anemia in Amazonian children: a population-based, cross-sectional study. . 2012; 7(5): e36341.		2007	Scientific literature
Sickle cell disorders	Brandelise S, Pinheiro V, Gabetta CS, Hambleton I, Serjeant B, Serjeant G. Newborn screening for sickle cell disease in Brazil: the Campinas experience. . 2004; 26(1): 15-9.		1992-2000	Scientific literature
Sickle cell disorders	Diniz D, Guedes C, Barbosa L, Tauil PL, Magalhães I. Prevalence of sickle cell trait and sickle cell anemia among newborns in the Federal District, Brazil, 2004 to 2006. . 2009; 25(1): 188-94.		2004-2006	Scientific literature
Sickle cell disorders	De Araújo MCPE, Serafim ESS, de Castro Jr WAP, de Medeiros TMD. Prevalence of abnormal hemoglobins in newborns in Natal, Rio Grande do Norte, Brazil . . 2004; 20(1): 123-8.		2001	Scientific literature
Sickle cell disorders	Lobo CL de C, Bueno LM, Moura P, Ogeda LL, Castilho S, de Carvalho SMF. Neonatal screening for hemoglobinopathies in Rio de Janeiro, Brazil. . 2003; 13(2-3): 154-9.		2000-2001	Scientific literature
Sickle cell disorders	Fernandes APPC, Januário JN, Cangussu CB, Macedo DL de, Viana MB. Mortality of children with sickle cell disease: a population study. . 2010; 86(4): 279-84.		1998-2005	Scientific literature

Sickle cell disorders	Wagner SC, de Castro SM, Gonzalez TP, Santin AP, Zaleski CF, Azevedo LA, Dreau H, Henderson S, Old J, Hutz MH. Neonatal screening for hemoglobinopathies: results of a public health system in South Brazil. . 2010; 14(4): 565-9.		2004-2007	Scientific literature
Sickle cell disorders	Zago MA, Costa FF, Tone LG, Bottura C. Hereditary hemoglobin disorders in a Brazilian population. . 1983; 33(2): 125-9.		1979-1982	Scientific literature
Sickle cell disorders	Melo-Reis PR de, Naoum PC, Diniz-Filho JAF, Dias-Penna KGB, Mesquita MM de, Balestra FA, Ternes YMF, Mascarenhas C do C, Chen LC. Prevalence of thalassemias and variant hemoglobins in the state of Goiás, Brazil. . 2006; 42(6): 425‐30.		2003-2004	Scientific literature
Sickle cell disorders	Carlos AM, Souza RA, Souza BM, Pereira Gde A, Tostes Junior S, Martins PR, Moraes-Souza H. Hemoglobinopathies in newborns in the southern region of the Triangulo Mineiro, Brazil. Cross-sectional study. . 2015; 133(5): 439-44.		2011-2013	Scientific literature
Sickle cell disorders	Salzano FM, Tondo CV. Hemoglobin types in Brazilian populations. . 1982; 6(1): 85‐97.		1950-1980	Scientific literature
Sickle cell disorders	hydroxcarbamide therapy on survival of children with sickle cell disease. . 2013; 161(6): 852-60.		2000-2009	Scientific literature
Sickle cell disorders	Sickle Cell and Thalassemias Prevalence Data, Personal Correspondence with David		1995-2010	Estimate
G6PD deficiency	with anemia in Amazonian children: a population-based, cross-sectional study. . 2012; 7(5): e36341.		2007	Scientific literature
G6PD deficiency	Saldanha PH, Nóbrega FG, Maia JC. Distribution and heredity of erythrocyte G6PD activity and electrophoretic variants among different racial groups at São Paulo, Brazil. . 1969; 6(1): 48-54.		1967-1969	Scientific literature
G6PD deficiency	Barreto OCO. Erythrocyte glucose-6-phosphate dehydrogenase deficiency in São Paulo, Brazil. . 1970; 3(1/2): 61-5.		1968-1970	Scientific literature
G6PD deficiency	Neto EC, Portal L, Ferreira LF. G6PD deficiency in an unselected Brazilian population. . 1999; 30(Suppl 2): 87.		1997-1999	Scientific literature
G6PD deficiency	Compri MB, Saad ST, Ramalho AS. Genetico-epidemiological and molecular investigation of G-6-PD deficiency in a Brazilian community. . 2000; 16(2): 335-42.		1995-1998	Scientific literature
G6PD deficiency	Castro S, Weber R, Dadalt V, Tavares V, Giugliani R. Prevalence of G6PD deficiency in newborns in the south of Brazil. . 2006; 13(2): 85-6.		2003	Scientific literature
G6PD deficiency	Ondei LS, Silveira LM, Leite AA, Souza DRS, Pinhel MAS, Percário S, Ricci Júnior O, Bonini-Domingos CR. Lipid peroxidation and antioxidant capacity of G6PD-deficient patients with A-(202G>A) mutation. . 2009; 8(4): 1345-51.		2006-2008	Scientific literature
G6PD deficiency	Neto M, De JP, Dourado MV, Reis MG dos, Gonçalves MS. A novel c.197T -> A variant among Brazilian neonates with glucose-6-phosphate dehydrogenase deficiency. . 2008; 31(1): 33-5.		2000	Scientific literature
G6PD deficiency	Santana MS, Monteiro WM, Siqueira AM, Costa MF, Sampaio V, Lacerda MV, Alecrim MG. Glucose-6-phosphate dehydrogenase deficient variants are associated with reduced susceptibility to malaria in the Brazilian Amazon. . 2013; 107(5): 301-6.		2009-2010	Scientific literature
G6PD deficiency	Domingos IF, Hatzlhofer BL, Oliveira FB, Araujo FR, Araujo AS, Lucena-Araujo AR, Bezerra MA. Prevalence and molecular defect characterization of glucose-6-phosphate dehydrogenase deficiency in Brazilian blood donors. . 2015; 37(5): e109-11.		2010-2011	Scientific literature
G6PD deficiency	Azevêdo ES, Alves AF, Da Silva MC, Souza MG, Muniz Dias Lima AM, Azevedo WC. Distribution of abnormal hemoglobins and glucose-6-phosphate dehydrogenase variants in 1200 school children of Bahia, Brazil. . 1980; 53(4): 509-12.		1977-1979	Scientific literature
Endocrine, metabolic, blood, and immune	Ministry of Health (Brazil). Brazil Hospital Information System 2003-2007.		2003-2007	Administrative record
Endocrine, metabolic, blood, and immune disorders	Health Institute (São Paulo, Brazil), State University of Campinas, São Paulo Municipal Health Department, São Paulo State University, University of São Paulo. Brazil - São Paulo Health Survey 2008-2009.	São Paulo	2002-2008	Survey
Endocrine, metabolic, blood, and immune	Ministry of Health (Brazil). Brazil Hospital Information System 2013-2014.		2013-2017	Administrative record
Endocrine, metabolic, blood, and immune disorders	Ministry of Health (Brazil). Brazil Hospital Information System 1997. Rio de Janeiro, Brazil: Ministry of Health (Brazil).	Country	1993-1997	Administrative record
Endocrine, metabolic, blood, and immune disorders	Brazilian Institute of Geography and Statistics (IBGE), Ministry of Health (Brazil), Ministry of Planning, Budget, and Management (Brazil). Brazil National Health Survey 2013. Rio de Janeiro, Brazil: Brazilian Institute of Geography and Statistics (IBGE).	Country	2013-2014	Survey
Endocrine, metabolic, blood, and immune	Ministry of Health (Brazil). Brazil Hospital Information System 2008-2012.		2008-2012	Administrative record
Endocrine, metabolic, blood, and immune	Brazil World Health Survey 2003	Country	2002-2003	Survey
Endocrine, metabolic, blood, and immune	Ministry of Health (Brazil). Brazil Hospital Information System 1998-2002.		1998-2002	Administrative record
Rheumatoid arthritis	Senna ER, De Barros ALP, Silva EO, Costa IF, Pereira LVB, Ciconelli RM, Ferraz MB. Prevalence of rheumatic diseases in Brazil: a study using the COPCORD approach. . 2004; 31(3): 594-7.		2001	Scientific literature
Rheumatoid arthritis	Bennett K, Cardiel MH, Ferraz MB, Riedemann P, Goldsmith CH, Tugwell P. Community screening for rheumatic disorder: cross cultural adaptation and screening characteristics of the COPCORD Core Questionnaire in Brazil, Chile, and Mexico. . 1997; 24(1): 160-8.		1994	Scientific literature
Low back and neck pain	Meucci RD, Fassa AG, Paniz VM, Silva MC, Wegman DH. Increase of chronic low back pain prevalence in a medium-sized city of southern Brazil. . 2013; 155.		2002-2010	Scientific literature
Low back and neck pain	Meziat Filho N, Coutinho ES, Azevedo e Silva G. Association between home posture habits and low back pain in high school adolescents. . 2015; 24(3): 425-33.		2012-2013	Scientific literature
Low back and neck pain	Almeida ICGB, Sá KN, Silva M, Baptista A, Matos MA, Lessa Í. Chronic low back pain prevalence in the population of the city of Salvador. . 2008; 43(3): 96‐102.		2000	Scientific literature
Low back and neck pain	De Vitta A, Martinez MG, Piza NT, Simeão SF de AP, Ferreira NP. [Prevalence of lower back pain and associated factors in students]. . 2011; 27(8): 1520-8.		2007	Scientific literature
Low back and neck pain	Mendoza-Sassi R, Béria JU, Fiori N, Bortolotto A. Prevalence of signs and symptoms, associated sociodemographic factors and resulting actions in an urban center in southern Brazil. . 2006; 20(1): 22-8.		2000	Scientific literature
Low back and neck pain	Blay SL, Andreoli SB, Dewey ME, Gastal FL. Co-occurrence of chronic physical pain and psychiatric morbidity in a community sample of older people. . 2007; 22(9): 902-8.		2004	Scientific literature
Low back and neck pain	Onofrio AC, da Silva MC, Domingues MR, Rombaldi AJ. Acute low back pain in high school adolescents in Southern Brazil: prevalence and associated factors. . 2012; 21(7): 1234-40.		2009	Scientific literature
Low back and neck pain	associated factors of back pain in adults from southern Brazil: a population-based study. . 2011; 15(1): 31-6.		2007	Scientific literature
Low back and neck pain	Brazil World Health Survey 2003	Country	2003	Survey
Low back and neck pain	Silva MCD, Fassa AG, Valle NCJ. [Chronic low back pain in a Southern Brazilian adult population: prevalence and associated factors]. . 2004; 20(2): 377-85.		2002	Scientific literature
Low back pain	Brazil World Health Survey 2003	Country	2003	Survey
Low back pain	Silva MCD, Fassa AG, Valle NCJ. [Chronic low back pain in a Southern Brazilian adult population: prevalence and associated factors]. . 2004; 20(2): 377-85.		2002	Scientific literature
Low back pain	Meucci RD, Fassa AG, Paniz VM, Silva MC, Wegman DH. Increase of chronic low back pain prevalence in a medium-sized city of southern Brazil. . 2013; 155.		2002-2010	Scientific literature
Low back pain	associated factors of back pain in adults from southern Brazil: a population-based study. . 2011; 15(1): 31-6.		2007	Scientific literature
Low back pain	Blay SL, Andreoli SB, Dewey ME, Gastal FL. Co-occurrence of chronic physical pain and psychiatric morbidity in a community sample of older people. . 2007; 22(9): 902-8.		2004	Scientific literature
Low back pain	Almeida ICGB, Sá KN, Silva M, Baptista A, Matos MA, Lessa Í. Chronic low back pain prevalence in the population of the city of Salvador. . 2008; 43(3): 96‐102.		2000	Scientific literature
Low back pain	De Vitta A, Martinez MG, Piza NT, Simeão SF de AP, Ferreira NP. [Prevalence of lower back pain and associated factors in students]. . 2011; 27(8): 1520-8.		2007	Scientific literature
Low back pain	Meziat Filho N, Coutinho ES, Azevedo e Silva G. Association between home posture habits and low back pain in high school adolescents. . 2015; 24(3): 425-33.		2012-2013	Scientific literature
Low back pain	Onofrio AC, da Silva MC, Domingues MR, Rombaldi AJ. Acute low back pain in high school adolescents in Southern Brazil: prevalence and associated factors. . 2012; 21(7): 1234-40.		2009	Scientific literature
Low back pain	Mendoza-Sassi R, Béria JU, Fiori N, Bortolotto A. Prevalence of signs and symptoms, associated sociodemographic factors and resulting actions in an urban center in southern Brazil. . 2006; 20(1): 22-8.		2000	Scientific literature
Other musculoskeletal disorders	Bennett K, Cardiel MH, Ferraz MB, Riedemann P, Goldsmith CH, Tugwell P. Community screening for rheumatic disorder: cross cultural adaptation and screening characteristics of the COPCORD Core Questionnaire in Brazil, Chile, and Mexico. . 1997; 24(1): 160-8.		1994	Scientific literature
Congenital birth defects	Santos LM, Lecca RC, Cortez-Escalante JJ, Sanchez MN, Rodrigues HG. Prevention of neural tube defects by the fortification of flour with folic acid: a population-based retrospective study in Brazil. . 2016; 94(1): 22-9.		2001-2014	Scientific literature
Congenital birth defects	Brazil Latin American Collaborative Study of Congenital Malformations Data 1993-1998 - WHO as it appears in	Country	1993-1998	Disease registry
Congenital birth defects	Melo BF, Aguiar MB, Bouzada MC, Aguiar RL, Pereira AK, Paixao GM, Linhares MC, Valerio FC, Simoes E Silva AC, Oliveira EA. Early risk factors for neonatal mortality in CAKUT: analysis of 524 affected newborns. . 2012; 27(6): 965-72.		1996-2006	Scientific literature
Neural tube defects	Ministry of Health (Brazil). Brazil Hospital Information System 2013-2014.		2013-2017	Administrative record
Neural tube defects	Brazil Latin American Collaborative Study of Congenital Malformations Data 1993-1998 - WHO as it appears in	Country	1993-1998	Disease registry
Neural tube defects	Santos LM, Lecca RC, Cortez-Escalante JJ, Sanchez MN, Rodrigues HG. Prevention of neural tube defects by the fortification of flour with folic acid: a population-based retrospective study in Brazil. . 2016; 94(1): 22-9.		2001-2014	Scientific literature

Neural tube defects	Bellizzi S, Ali MM, Abalos E, Betran AP, Kapila J, Pileggi-Castro C, Vogel JP, Merialdi M. Are hypertensive disorders in pregnancy associated with congenital malformations in offspring? Evidence from the WHO Multicountry cross sectional survey on maternal and newborn health. . 2016; 16(1): 198.		2010-2011	Scientific literature
Neural tube defects	Institute for Health Metrics and Evaluation (IHME). IHME GBD DisMod Congenital Anomalies Birth Prevalence Estimates.	Global	1990-2016	Modeled data
Neural tube defects	Brazil World Health Survey 2003	Country	2002-2003	Survey
Neural tube defects	Brazilian Institute of Geography and Statistics (IBGE), Ministry of Health (Brazil), Ministry of Planning, Budget, and Management (Brazil). Brazil National Health Survey 2013. Rio de Janeiro, Brazil: Brazilian Institute of Geography and Statistics (IBGE).	Country	2013-2014	Survey
Neural tube defects	Ministry of Health (Brazil). Brazil Hospital Information System 1997. Rio de Janeiro, Brazil: Ministry of Health (Brazil).	Country	1993-1997	Administrative record
Neural tube defects	Ministry of Health (Brazil). Brazil Hospital Information System 2008-2012.		2008-2012	Administrative record
Neural tube defects	Ministry of Health (Brazil). Brazil Hospital Information System 2003-2007.		2003-2007	Administrative record
Neural tube defects	Health Institute (São Paulo, Brazil), State University of Campinas, São Paulo Municipal Health Department, São Paulo State University, University of São Paulo. Brazil - São Paulo Health Survey 2008-2009.	São Paulo	2002-2008	Survey
Neural tube defects	Ministry of Health (Brazil). Brazil Hospital Information System 1998-2002.		1998-2002	Administrative record
Congenital heart anomalies	Leite DCF, de Mendonça JT, Cipolotti R, de Melo EV. Heart defects treatment in Sergipe: propose of resources' rationalization to improve care. . 2012; 27(2): 224-30.		2000-2009	Scientific literature
Congenital heart anomalies	Brazilian Institute of Geography and Statistics (IBGE), Ministry of Health (Brazil), Ministry of Planning, Budget, and Management (Brazil). Brazil National Health Survey 2013. Rio de Janeiro, Brazil: Brazilian Institute of Geography and Statistics (IBGE).	Country	2013-2014	Survey
Congenital heart anomalies	Brazil World Health Survey 2003	Country	2002-2003	Survey
Congenital heart anomalies	Institute for Health Metrics and Evaluation (IHME). IHME GBD DisMod Less Severe Congenital Heart Anomalies Birth Prevalence Estimates.	Global	1990-2015	Modeled data
Congenital heart anomalies	Tennant PWG, Pearce MS, Bythell M, Rankin J. 20-year survival of children born with congenital anomalies: a population-based study. Lancet. 2010; 375(9715): 649-56. and Congenital Heart Anomalies Mortality Risk With No Diagnosis or Care Estimates as provided by the Global Burden of Disease 2010 congenital anomaly expert group. [Unpublished].		1990-2015	Estimate
Congenital heart anomalies	Roos-Hesselink J, Perlroth MG, McGhie J, Spitaels S. Atrial arrhythmias in adults after repair of tetralogy of Fallot. Correlations with clinical, exercise, and echocardiographic findings. . 1995; 91(8): 2214-9.		2009-2011	Scientific literature
Congenital heart anomalies	Ministry of Health (Brazil). Brazil Hospital Information System 2008-2012.		2008-2012	Administrative record
Congenital heart anomalies	Guitti JC. Epidemiological characteristics of congenital heart diseases in Londrina, Paraná south Brazil. . 2000; 74(5): 395-404.		1989-1998	Scientific literature
Congenital heart anomalies	Amorim LFP, Pires CAB, Lana AMA, Campos AS, Aguiar RALP, Tibúrcio JD, Siqueira AL, Mota CCC, Aguiar MJB. Presentation of congenital heart disease diagnosed at birth: analysis of 29,770 newborn infants. . 2008; 84(1): 83-90.		1990-2003	Scientific literature
Congenital heart anomalies	Ministry of Health (Brazil). Brazil Hospital Information System 1998-2002.		1998-2002	Administrative record
Congenital heart anomalies	Bellizzi S, Ali MM, Abalos E, Betran AP, Kapila J, Pileggi-Castro C, Vogel JP, Merialdi M. Are hypertensive disorders in pregnancy associated with congenital malformations in offspring? Evidence from the WHO Multicountry cross sectional survey on maternal and newborn health. . 2016; 16(1): 198.		2010-2011	Scientific literature
Congenital heart anomalies	Ministry of Health (Brazil). Brazil Hospital Information System 2013-2014.		2013-2017	Administrative record
Congenital heart anomalies	Ministry of Health (Brazil). Brazil Hospital Information System 1997. Rio de Janeiro, Brazil: Ministry of Health (Brazil).	Country	1993-1997	Administrative record
Congenital heart anomalies	Brazil Latin American Collaborative Study of Congenital Malformations Data 1993-1998 - WHO as it appears in	Country	1993-1998	Disease registry
Congenital heart anomalies	Ministry of Health (Brazil). Brazil Hospital Information System 2003-2007.		2003-2007	Administrative record
Congenital heart anomalies	Health Institute (São Paulo, Brazil), State University of Campinas, São Paulo Municipal Health Department, São Paulo State University, University of São Paulo. Brazil - São Paulo Health Survey 2008-2009.	São Paulo	2002-2008	Survey
Orofacial clefts	Ministry of Health (Brazil). Brazil Hospital Information System 2008-2012.		2008-2012	Administrative record
Orofacial clefts	Ministry of Health (Brazil). Brazil Hospital Information System 2003-2007.		2003-2007	Administrative record
Orofacial clefts	Brazilian Institute of Geography and Statistics (IBGE), Ministry of Health (Brazil), Ministry of Planning, Budget, and Management (Brazil). Brazil National Health Survey 2013. Rio de Janeiro, Brazil: Brazilian Institute of Geography and Statistics (IBGE).	Country	2013-2014	Survey
Orofacial clefts	Brazil World Health Survey 2003	Country	2002-2003	Survey
Orofacial clefts	Ministry of Health (Brazil). Brazil Hospital Information System 2013-2014.		2013-2017	Administrative record
Orofacial clefts	Health Institute (São Paulo, Brazil), State University of Campinas, São Paulo Municipal Health Department, São Paulo State University, University of São Paulo. Brazil - São Paulo Health Survey 2008-2009.	São Paulo	2002-2008	Survey
Orofacial clefts	Ministry of Health (Brazil). Brazil Hospital Information System 1997. Rio de Janeiro, Brazil: Ministry of Health (Brazil).	Country	1993-1997	Administrative record
Orofacial clefts	Bellizzi S, Ali MM, Abalos E, Betran AP, Kapila J, Pileggi-Castro C, Vogel JP, Merialdi M. Are hypertensive disorders in pregnancy associated with congenital malformations in offspring? Evidence from the WHO Multicountry cross sectional survey on maternal and newborn health. . 2016; 16(1): 198.		2010-2011	Scientific literature
Orofacial clefts	Ministry of Health (Brazil). Brazil Hospital Information System 1998-2002.		1998-2002	Administrative record
Down syndrome	Brazil World Health Survey 2003	Country	2002-2003	Survey
Down syndrome	Ministry of Health (Brazil). Brazil Hospital Information System 1998-2002.		1998-2002	Administrative record
Down syndrome	Brazilian Institute of Geography and Statistics (IBGE), Ministry of Health (Brazil), Ministry of Planning, Budget, and Management (Brazil). Brazil National Health Survey 2013. Rio de Janeiro, Brazil: Brazilian Institute of Geography and Statistics (IBGE).	Country	2013-2014	Survey
Down syndrome	Ministry of Health (Brazil). Brazil Hospital Information System 2003-2007.		2003-2007	Administrative record
Down syndrome	Ministry of Health (Brazil). Brazil Hospital Information System 2013-2014.		2013-2017	Administrative record
Down syndrome	Health Institute (São Paulo, Brazil), State University of Campinas, São Paulo Municipal Health Department, São Paulo State University, University of São Paulo. Brazil - São Paulo Health Survey 2008-2009.	São Paulo	2002-2008	Survey
Down syndrome	Castilla EE, Rittler M, Dutra MG, Lopez-Camelo JS, Campaña H, Paz JE, Orioli IM. Survival of children with Down syndrome in South America. ECLAMC-Downsurv Group. Latin American Collaborative Study of Congenital Malformations. . 1998; 79(2): 108-11.		1988-1995	Scientific literature
Down syndrome	Brazil Latin American Collaborative Study of Congenital Malformations Data 1993-1998 - WHO as it appears in	Country	1993-1998	Disease registry
Down syndrome	Ministry of Health (Brazil). Brazil Hospital Information System 1997. Rio de Janeiro, Brazil: Ministry of Health (Brazil).	Country	1993-1997	Administrative record
Down syndrome	Ministry of Health (Brazil). Brazil Hospital Information System 2008-2012.		2008-2012	Administrative record
Turner syndrome	Ministry of Health (Brazil). Brazil Hospital Information System 2013-2014.		2013-2017	Administrative record
Turner syndrome	Health Institute (São Paulo, Brazil), State University of Campinas, São Paulo Municipal Health Department, São Paulo State University, University of São Paulo. Brazil - São Paulo Health Survey 2008-2009.	São Paulo	2002-2008	Survey
Turner syndrome	Brazil World Health Survey 2003	Country	2002-2003	Survey
Turner syndrome	Ministry of Health (Brazil). Brazil Hospital Information System 2003-2007.		2003-2007	Administrative record
Turner syndrome	Ministry of Health (Brazil). Brazil Hospital Information System 1997. Rio de Janeiro, Brazil: Ministry of Health (Brazil).	Country	1993-1997	Administrative record
Turner syndrome	Brazilian Institute of Geography and Statistics (IBGE), Ministry of Health (Brazil), Ministry of Planning, Budget, and Management (Brazil). Brazil National Health Survey 2013. Rio de Janeiro, Brazil: Brazilian Institute of Geography and Statistics (IBGE).	Country	2013-2014	Survey
Turner syndrome	Ministry of Health (Brazil). Brazil Hospital Information System 2008-2012.		2008-2012	Administrative record
Turner syndrome	Ministry of Health (Brazil). Brazil Hospital Information System 1998-2002.		1998-2002	Administrative record
Klinefelter syndrome	Ministry of Health (Brazil). Brazil Hospital Information System 1997. Rio de Janeiro, Brazil: Ministry of Health (Brazil).	Country	1993-1997	Administrative record
Klinefelter syndrome	Ministry of Health (Brazil). Brazil Hospital Information System 2003-2007.		2003-2007	Administrative record
Klinefelter syndrome	Ministry of Health (Brazil). Brazil Hospital Information System 1998-2002.		1998-2002	Administrative record
Klinefelter syndrome	Brazilian Institute of Geography and Statistics (IBGE), Ministry of Health (Brazil), Ministry of Planning, Budget, and Management (Brazil). Brazil National Health Survey 2013. Rio de Janeiro, Brazil: Brazilian Institute of Geography and Statistics (IBGE).	Country	2013-2014	Survey
Klinefelter syndrome	Brazil World Health Survey 2003	Country	2002-2003	Survey
Klinefelter syndrome	Ministry of Health (Brazil). Brazil Hospital Information System 2013-2014.		2013-2017	Administrative record
Klinefelter syndrome	Health Institute (São Paulo, Brazil), State University of Campinas, São Paulo Municipal Health Department, São Paulo State University, University of São Paulo. Brazil - São Paulo Health Survey 2008-2009.	São Paulo	2002-2008	Survey
Klinefelter syndrome	Ministry of Health (Brazil). Brazil Hospital Information System 2008-2012.		2008-2012	Administrative record
Other chromosomal abnormalities	Polli JB, Groff D de P, Petry P, Mattos VF, Rosa RCM, Zen PRG, Graziadio C, Paskulin GA, Rosa RFM. Trisomy 13 (Patau syndrome) and congenital heart defects. . 2014; 164A(1): 272[illegible]5.		1990-2012	Scientific literature

Other chromosomal abnormalities	Petry P, Polli JB, Mattos VF, Rosa RCM, Zen PRG, Graziadio C, Paskulin GA, Rosa RFM. Clinical features and prognosis of a sample of patients with trisomy 13 (Patau syndrome) from Brazil. . 2013; 161A(6): 1278[illegible]83.		1975-2012	Scientific literature
Other chromosomal abnormalities	Brazil Latin American Collaborative Study of Congenital Malformations Data 1993-1998 - WHO as it appears in	Country	1993-1998	Disease registry
Congenital musculoskeletal and limb anomalies	Health Institute (São Paulo, Brazil), State University of Campinas, São Paulo Municipal Health Department, São Paulo State University, University of São Paulo. Brazil - São Paulo Health Survey 2008-2009.	São Paulo	2002-2008	Survey
Congenital musculoskeletal and limb anomalies	Ministry of Health (Brazil). Brazil Hospital Information System 2013-2014.		2013-2017	Administrative record
Congenital musculoskeletal and limb anomalies	Ministry of Health (Brazil). Brazil Hospital Information System 2008-2012.		2008-2012	Administrative record
Congenital musculoskeletal and limb anomalies	Ministry of Health (Brazil). Brazil Hospital Information System 1998-2002.		1998-2002	Administrative record
Congenital musculoskeletal and limb anomalies	Brazil Latin American Collaborative Study of Congenital Malformations Data 1993-1998 - WHO as it appears in	Country	1993-1998	Disease registry
Congenital musculoskeletal and limb anomalies	Brazil World Health Survey 2003	Country	2002-2003	Survey
Congenital musculoskeletal and limb anomalies	Bellizzi S, Ali MM, Abalos E, Betran AP, Kapila J, Pileggi-Castro C, Vogel JP, Merialdi M. Are hypertensive disorders in pregnancy associated with congenital malformations in offspring? Evidence from the WHO Multicountry cross sectional survey on maternal and newborn health. . 2016; 16(1): 198.		2010-2011	Scientific literature
Congenital musculoskeletal and limb anomalies	Ministry of Health (Brazil). Brazil Hospital Information System 1997. Rio de Janeiro, Brazil: Ministry of Health (Brazil).	Country	1993-1997	Administrative record
Congenital musculoskeletal and limb anomalies	Ministry of Health (Brazil). Brazil Hospital Information System 2003-2007.		2003-2007	Administrative record
Congenital musculoskeletal and limb anomalies	Brazilian Institute of Geography and Statistics (IBGE), Ministry of Health (Brazil), Ministry of Planning, Budget, and Management (Brazil). Brazil National Health Survey 2013. Rio de Janeiro, Brazil: Brazilian Institute of Geography and Statistics (IBGE).	Country	2013-2014	Survey
Urogenital congenital anomalies	Bellizzi S, Ali MM, Abalos E, Betran AP, Kapila J, Pileggi-Castro C, Vogel JP, Merialdi M. Are hypertensive disorders in pregnancy associated with congenital malformations in offspring? Evidence from the WHO Multicountry cross sectional survey on maternal and newborn health. . 2016; 16(1): 198.		2010-2011	Scientific literature
Urogenital congenital anomalies	Sircili MHP, e Silva FA de Q, Costa EMF, Brito VN, Arnhold IJP, Dénes FT, Inacio M, de Mendonca BB. Long-term surgical outcome of masculinizing genitoplasty in large cohort of patients with disorders of sex development. . 2010; 184(3): 1122[illegible]7.		1965-2008	Scientific literature
Urogenital congenital anomalies	Brazil Latin American Collaborative Study of Congenital Malformations Data 1993-1998 - WHO as it appears in	Country	1993-1998	Disease registry
Urogenital congenital anomalies	Melo BF, Aguiar MB, Bouzada MC, Aguiar RL, Pereira AK, Paixao GM, Linhares MC, Valerio FC, Simoes E Silva AC, Oliveira EA. Early risk factors for neonatal mortality in CAKUT: analysis of 524 affected newborns. . 2012; 27(6): 965-72.		1996-2006	Scientific literature
Digestive congenital anomalies	Brazil Latin American Collaborative Study of Congenital Malformations Data 1993-1998 - WHO as it appears in	Country	1993-1998	Disease registry
Dermatitis	Freitas MS, Monteiro JCS, Camelo-Nunes IC, Solé D. Prevalence of asthma symptoms and associated factors in schoolchildren from Brazilian Amazon islands. . 2012; 49(6): 600-5.		2007-2009	Scientific literature
Dermatitis	Castro LKK de, Cerci Neto A, Ferreira Filho OF. Prevalence of symptoms of asthma, rhinitis and atopic eczema among students between 6 and 7 years of age in the city of Londrina, Brazil. . 2010; 36(3): 286-92.		2008	Scientific literature
Dermatitis	Williams H, Stewart A, Von Mutius E, Cookson W, Anderson HR. Is eczema really on the increase worldwide. . 2008; 121(4): 947-954.	Country	1994-2003	Scientific literature
Dermatitis	Odhiambo JA, Williams HC, Clayton TO, Robertson CF, Asher MI, ISAAC Phase Three Study Group. Global variations in prevalence of eczema symptoms in children from ISAAC Phase Three. . 2009; 124(6): 1251-1258.		2000-2003	Scientific literature
Dermatitis	Naspitz CK. Changes in the Prevalence of Asthma and Allergic Diseases among Brazilian Schoolchildren (13-14 years old): Comparison between ISAAC Phases One and Three. . 2006; 53(1): 13[illegible]21.		1994-2003	Scientific literature
Dermatitis	Garcia-Marcos L, Robertson CF, Ross Anderson H, Ellwood P, Williams HC, Wong GW. Does migration affect asthma, rhinoconjunctivitis and eczema prevalence? Global findings from the international study of asthma and allergies in childhood. . 2014; 43(6): 1846-54.		2000-2003	Scientific literature
Dermatitis	prevalence of rhinoconjunctivitis but not asthma and atopic eczema in teenagers. . 2005; 15(3): 183.		1995-2001	Scientific literature
Dermatitis	Toledo MF, Rozov T, Leone C. Prevalence of asthma and allergies in 13- to 14-year-old adolescents and the frequency of risk factors in carriers of current asthma in Taubaté, São Paulo, Brazil. . 2011; 39(5): 284-90.		2008-2010	Scientific literature
Dermatitis	Mascarenhas JM, Silva Rde C, Assis AM, Pinto Ede J, Conceicao JS, Barreto ML. Symptoms of asthma and associated factors in adolescents from Salvador, Bahia. . 2016; 19(1): 181-93.		2009	Scientific literature
Dermatitis	Solé D, Mallol J, Wandalsen GF, Aguirre V, Latin American ISAAC Phase 3 Study Group. Prevalence of symptoms of eczema in Latin America: results of the International Study of Asthma and Allergies in Childhood (ISAAC) Phase 3. . 2010; 20(4): 311-23.		2001-2003	Scientific literature
Dermatitis	infections and immunizations with asthma and allergic sensitization in ISAAC Phase Two. . 2012; 23(8): 737-46.		1995-2005	Scientific literature
Dermatitis	Sole D, Rosario Filho NA, Sarinho ES, Camelo-Nunes IC, Barreto BA, Medeiros ML, Franco JM, Camargos PA, Mallol J, Gurgel R, Andrade DM, Furlan FP, Silva AR, Cardozo C, Andrade C. Prevalence of asthma and allergic diseases in adolescents: nine-year follow-up study (2003-2012). . 2015; 91(1): 30-5.		2003-2012	Scientific literature
Dermatitis	Palvo F, Toledo EC, Menin AM, Jorge PP, Godoy MF, Sole´ D. Risk factors of childhood asthma in Sao Jose do Rio Preto, Sao Paulo, Brazil. . 2008; 54(4): 253-7.		2003-2004	Scientific literature
Dermatitis	Camelo-Nunes IC, Wandalsen GF, Melo KC, Naspitz CK, Solé D. [Prevalence of atopic eczema and associated symptoms in school children]. . 2004; 80(1): 60[illegible]4.		1996-1999	Scientific literature
Dermatitis	Tejada C dos S, Mendoza-Sassi RA, Almeida HL de Jr, Figueiredo PN, Tejada VF dos S. Impact on the quality of life of dermatological patients in southern Brazil. . 2011; 86(6): 1113-21.		2008-2009	Scientific literature
Psoriasis	Fuji R, Mould JFJ, Tang B. Burden of disease in patients with diagnosed psoriasis in Brazil: results from 2011 national health and wellness survey (NHWS). . 2012; 15: A107.		2011	Scientific literature
Cellulitis	Brazil World Health Survey 2003	Country	2002-2003	Survey
Cellulitis	Ministry of Health (Brazil). Brazil Hospital Information System 1997. Rio de Janeiro, Brazil: Ministry of Health (Brazil).	Country	1993-1997	Administrative record
Cellulitis	Brazilian Institute of Geography and Statistics (IBGE), Ministry of Health (Brazil), Ministry of Planning, Budget, and Management (Brazil). Brazil National Health Survey 2013. Rio de Janeiro, Brazil: Brazilian Institute of Geography and Statistics (IBGE).	Country	2013-2014	Survey
Cellulitis	Ministry of Health (Brazil). Brazil Hospital Information System 1998-2002.		1998-2002	Administrative record
Cellulitis	Ministry of Health (Brazil). Brazil Hospital Information System 2013-2014.		2013-2017	Administrative record
Cellulitis	Health Institute (São Paulo, Brazil), State University of Campinas, São Paulo Municipal Health Department, São Paulo State University, University of São Paulo. Brazil - São Paulo Health Survey 2008-2009.	São Paulo	2002-2008	Survey
Cellulitis	Ministry of Health (Brazil). Brazil Hospital Information System 2003-2007.		2003-2007	Administrative record
Cellulitis	Ministry of Health (Brazil). Brazil Hospital Information System 2008-2012.		2008-2012	Administrative record
Pyoderma	Ministry of Health (Brazil). Brazil Hospital Information System 2008-2012.		2008-2012	Administrative record
Pyoderma	Ministry of Health (Brazil). Brazil Hospital Information System 2013-2014.		2013-2017	Administrative record
Pyoderma	Brazilian Institute of Geography and Statistics (IBGE), Ministry of Health (Brazil), Ministry of Planning, Budget, and Management (Brazil). Brazil National Health Survey 2013. Rio de Janeiro, Brazil: Brazilian Institute of Geography and Statistics (IBGE).	Country	2013-2014	Survey
Pyoderma	Health Institute (São Paulo, Brazil), State University of Campinas, São Paulo Municipal Health Department, São Paulo State University, University of São Paulo. Brazil - São Paulo Health Survey 2008-2009.	São Paulo	2002-2008	Survey
Pyoderma	Brazil World Health Survey 2003	Country	2002-2003	Survey
Pyoderma	Ministry of Health (Brazil). Brazil Hospital Information System 1997. Rio de Janeiro, Brazil: Ministry of Health (Brazil).	Country	1993-1997	Administrative record
Pyoderma	Ministry of Health (Brazil). Brazil Hospital Information System 2003-2007.		2003-2007	Administrative record
Pyoderma	Ministry of Health (Brazil). Brazil Hospital Information System 1998-2002.		1998-2002	Administrative record
Scabies	Bechelli LM, Haddad N, Pimenta WP, Pagnano PM, Melchior E Jr, Fregnan RC, Zanin LC, Arenas A. Epidemiological survey of skin diseases in schoolchildren living in the Purus Valley (Acre State, Amazonia, Brazil). . 1981; 163(1): 78-93.		1974-1975	Scientific literature
Scabies	Heukelbach J. The epidemiology of scabies in an impoverished community in rural Brazil: presence and severity of disease are associated with poor living conditions and illiteracy. . 2009; 60(3): 436-43.		2003	Scientific literature
Scabies	Heukelbach J, Wilcke T, Winter B, Feldmeier H. Epidemiology and morbidity of scabies and pediculosis capitis in resource-poor communities in Brazil. . 2005; 153(1): 150-6.		2001	Scientific literature
Acne vulgaris	Almeida Hd, Cecconi J, Duquia RP, Souza PR, Breunig J. Sensitivity and specificity of self-reported acne in 18-year-old adolescent males. . 2013; 52(8): 946-8.		2010-2012	Scientific literature
Acne vulgaris	Bechelli LM, Haddad N, Pimenta WP, Pagnano PM, Melchior E Jr, Fregnan RC, Zanin LC, Arenas A. Epidemiological survey of skin diseases in schoolchildren living in the Purus Valley (Acre State, Amazonia, Brazil). . 1981; 163(1): 78-93.		1974-1975	Scientific literature

Acne vulgaris	Laczynski CMM, Cestari S da CP. Prevalence of dermatosis in scholars in the region of ABC paulista. . 2011; 86(3): 469-76.		2006	Scientific literature
Alopecia areata	Bechelli LM, Haddad N, Pimenta WP, Pagnano PM, Melchior E Jr, Fregnan RC, Zanin LC, Arenas A. Epidemiological survey of skin diseases in schoolchildren living in the Purus Valley (Acre State, Amazonia, Brazil). . 1981; 163(1): 78-93.		1974-1975	Scientific literature
Decubitus ulcer	Ministry of Health (Brazil). Brazil Hospital Information System 1997. Rio de Janeiro, Brazil: Ministry of Health (Brazil).	Country	1993-1997	Administrative record
Decubitus ulcer	Ministry of Health (Brazil). Brazil Hospital Information System 2003-2007.		2003-2007	Administrative record
Decubitus ulcer	Ministry of Health (Brazil). Brazil Hospital Information System 1998-2002.		1998-2002	Administrative record
Decubitus ulcer	Ministry of Health (Brazil). Brazil Hospital Information System 2013-2014.		2013-2017	Administrative record
Decubitus ulcer	Ministry of Health (Brazil). Brazil Hospital Information System 2008-2012.		2008-2012	Administrative record
Decubitus ulcer	Health Institute (São Paulo, Brazil), State University of Campinas, São Paulo Municipal Health Department, São Paulo State University, University of São Paulo. Brazil - São Paulo Health Survey 2008-2009.	São Paulo	2002-2008	Survey
Decubitus ulcer	Brazil World Health Survey 2003	Country	2002-2003	Survey
Decubitus ulcer	Brazilian Institute of Geography and Statistics (IBGE), Ministry of Health (Brazil), Ministry of Planning, Budget, and Management (Brazil). Brazil National Health Survey 2013. Rio de Janeiro, Brazil: Brazilian Institute of Geography and Statistics (IBGE).	Country	2013-2014	Survey
Glaucoma	Arieta CEL, de Oliveira DF, Lupinacci AP de C, Novaes P, Paccola M, Jose NK, Limburg H. Cataract remains an important cause of blindness in Campinas, Brazil. . 2009; 16(1): 58-63.		2003	Scientific literature
Glaucoma	International Centre for Eye Health (ICEH). Brazil - Campinas Rapid Assessment of Avoidable Blindness 2004. Grootebroek, Netherlands: RAAB Repository.	São Paulo	2003	Survey
Glaucoma	Araújo Filho A, Salomão SR, Berezovsky A, Cinoto RW, Morales PHA, Santos FRG, Belfort R Jr. Prevalence of visual impairment, blindness, ocular disorders and cataract surgery outcomes in low-income elderly from a metropolitan region of São Paulo-Brazil. . 2008; 71(2): 246-53.		2002	Scientific literature
Glaucoma	Prevalence and causes of visual impairment in a Brazilian population: the Botucatu Eye Study. . 2009; 9: 8.		2006-2007	Scientific literature
Cataract	Prevalence and causes of visual impairment in a Brazilian population: the Botucatu Eye Study. . 2009; 9: 8.		2006-2007	Scientific literature
Cataract	International Centre for Eye Health (ICEH). Brazil - Campinas Rapid Assessment of Avoidable Blindness 2004. Grootebroek, Netherlands: RAAB Repository.	São Paulo	2003	Survey
Cataract	Araújo Filho A, Salomão SR, Berezovsky A, Cinoto RW, Morales PHA, Santos FRG, Belfort R Jr. Prevalence of visual impairment, blindness, ocular disorders and cataract surgery outcomes in low-income elderly from a metropolitan region of São Paulo-Brazil. . 2008; 71(2): 246-53.		2002	Scientific literature
Cataract	Arieta CEL, de Oliveira DF, Lupinacci AP de C, Novaes P, Paccola M, Jose NK, Limburg H. Cataract remains an important cause of blindness in Campinas, Brazil. . 2009; 16(1): 58-63.		2003	Scientific literature
Macular degeneration	International Centre for Eye Health (ICEH). Brazil - Campinas Rapid Assessment of Avoidable Blindness 2004. Grootebroek, Netherlands: RAAB Repository.	São Paulo	2003	Survey
Macular degeneration	Prevalence and causes of visual impairment in a Brazilian population: the Botucatu Eye Study. . 2009; 9: 8.		2006-2007	Scientific literature
Macular degeneration	Araújo Filho A, Salomão SR, Berezovsky A, Cinoto RW, Morales PHA, Santos FRG, Belfort R Jr. Prevalence of visual impairment, blindness, ocular disorders and cataract surgery outcomes in low-income elderly from a metropolitan region of São Paulo-Brazil. . 2008; 71(2): 246-53.		2002	Scientific literature
Refraction and accommodation disorders	Brazil World Health Survey 2003	Country	2003	Survey
Refraction and accommodation disorders	Araújo Filho A, Salomão SR, Berezovsky A, Cinoto RW, Morales PHA, Santos FRG, Belfort R Jr. Prevalence of visual impairment, blindness, ocular disorders and cataract surgery outcomes in low-income elderly from a metropolitan region of São Paulo-Brazil. . 2008; 71(2): 246-53.		2002	Scientific literature
Refraction and accommodation disorders	Arieta CEL, de Oliveira DF, Lupinacci AP de C, Novaes P, Paccola M, Jose NK, Limburg H. Cataract remains an important cause of blindness in Campinas, Brazil. . 2009; 16(1): 58-63.		2003	Scientific literature
Refraction and accommodation disorders	Moraes Ibrahim F, Moraes Ibrahim M, Pomepo de Camargo JR, Veronese Rodrigues M de L, Scott IU, Silva Paula J. Visual impairment and myopia in Brazilian children: a population-based study. . 2013; 90(3): 223-7.		2007	Scientific literature
Refraction and accommodation disorders	International Centre for Eye Health (ICEH). Brazil - Campinas Rapid Assessment of Avoidable Blindness 2004. Grootebroek, Netherlands: RAAB Repository.	São Paulo	2003	Survey
Refraction and accommodation disorders	Salomao SR, Cinoto RW, Berezovsky A, Araujo-Filho A, Mitsuhiro MRKH, Mendieta L, Morales PHA, Pokharel GP, Belfort R Jr, Ellwein LB. Prevalence and causes of vision impairment and blindness in older adults in Brazil: the Sao Paulo Eye Study. . 2008; 15(3): 167-75.		2004	Scientific literature
Refraction and accommodation disorders	Duarte WR, Barros AJD, Dias-da-Costa JS, Cattan JM. [Prevalence of near vision deficiency and related factors: a population-based study]. . 2003; 19(2): 551-9.		1999-2000	Scientific literature
Refraction and accommodation disorders	Prevalence and causes of visual impairment in a Brazilian population: the Botucatu Eye Study. . 2009; 9: 8.		2006-2007	Scientific literature
Age-related and other hearing loss	Baraky LR, Bento RF, Raposo NRB, Tibiriçá SHC, Ribeiro LC, Barone MMVB, Vasconcelos NB. Disabling hearing loss prevalence in Juiz de Fora, Brazil. . 2012; 78(4): 52-8.		2008-2011	Scientific literature
Age-related and other hearing loss	Gondim LMA, Balen SA, Zimmermann KJ, Pagnossin DF, Fialho I de M, Roggia SM. Study of the prevalence of impaired hearing and its determinants in the city of Itajaí, Santa Catarina State, Brazil. . 2012; 78(2): 27-34.		2008-2011	Scientific literature
Age-related and other hearing loss	Béria JU, Raymann BCW, Gigante LP, Figueiredo ACL, Jotz G, Roithman R, Selaimen da Costa S, Garcez V, Scherer C, Smith A. Hearing impairment and socioeconomic factors: a population-based survey of an urban locality in southern Brazil. . 2007; 21(6): 381-7.		2003	Scientific literature
Age-related and other hearing loss	Bevilacqua MC, Banhara MR, Oliveira AN, Moret AL, Alvarenga Kde F, Caldana Mde L, Camargo LM, Costa OA, Bastos JR. Survey of hearing disorders in an urban population in Rondonia, Brazil. . 2013; 47(2): 309-15.	Rondonia	2005-2007	Scientific literature
Age-related and other hearing loss	Brazil Demographic Census 2010 - IPUMS	Country	2010	Census
Age-related and other hearing loss	Brazil General Census 2000 - IPUMS	Country	2000	Census
Age-related and other hearing loss	Brazil General Census 1991 - IPUMS	Country	1991	Census
Age-related and other hearing loss	Bevilacqua MC, Alvarenga K de F, Costa OA, Moret ALM. The universal newborn hearing screening in Brazil: from identification to intervention. . 2010; 74(5): 510-5.	Country	2007-2009	Scientific literature
Other vision loss	Araújo Filho A, Salomão SR, Berezovsky A, Cinoto RW, Morales PHA, Santos FRG, Belfort R Jr. Prevalence of visual impairment, blindness, ocular disorders and cataract surgery outcomes in low-income elderly from a metropolitan region of São Paulo-Brazil. . 2008; 71(2): 246-53.		2002	Scientific literature
Other vision loss	International Centre for Eye Health (ICEH). Brazil - Campinas Rapid Assessment of Avoidable Blindness 2004. Grootebroek, Netherlands: RAAB Repository.	São Paulo	2003	Survey
Caries of deciduous teeth	Almeida TF de, Vianna MIP, Cabral MBB de S, Cangussu MCT, Floriano FR. Family context and incidence of dental caries in preschool children living in areas covered by the Family Health Strategy in Salvador, Bahia State, Brazil. . 2012; 28(6): 1183-95.		2007-2008	Scientific literature
Caries of deciduous teeth	Jeremias F, de Souza JF, Silva CM da C, Cordeiro R de CL, Zuanon ACC, Santos-Pinto L. Dental caries experience and Molar-Incisor Hypomineralization. . 2013; 71(3-4): 870-6.		2011-2012	Scientific literature
Caries of deciduous teeth	Carvalho JC, Silva EF, Gomes RR, Fonseca JAC, Mestrinho HD. Impact of enamel defects on early caries development in preschool children. . 2011; 45(4): 353-60.		2009-2010	Scientific literature
Caries of deciduous teeth	Rebelo MA, Lopes MC, Vieira JM, Parente RC. Dental caries and gingivitis among 15 to 19 year-old students in Manaus, AM, Brazil. . 2009; 23(3): 248-54.	Amazonas	2006-2008	Scientific literature
Caries of deciduous teeth	and social class in a random sample of five-year-old preschool children in a Brazilian city. . 2010; 8(2): 125-32.		2005	Scientific literature
Caries of deciduous teeth	Bastos JL, Nomura LH, Peres MA. Dental pain, socioeconomic status, and dental caries in young male adults from southern Brazil. . 2005; 21(5): 1416-23.	Santa Catarina	2003	Scientific literature
Caries of deciduous teeth	prevalence and severity of dental caries in children with primary dentition. . 2012; 26(6): 564-70.		2010	Scientific literature
Caries of deciduous teeth	Bönecker M, Ardenghi TM, Oliveira LB, Sheiham A, Marcenes W. Trends in dental caries in 1- to 4-year-old children in a Brazilian city between 1997 and 2008. . 2010; 20(2): 125-31.		1997-2008	Scientific literature
Caries of deciduous teeth	Caries is the main cause for dental pain in childhood: findings from a birth cohort. . 2012; 46(5): 488-95.		2009	Scientific literature
Caries of deciduous teeth	Marquezan M, Marquezan M, Faraco-Junior IM, Feldens CA, Kramer PF, Ferreira SH. Association between occlusal anomalies and dental caries in 3- to 5 year-old Brazilian children. . 2011; 38(1): 8-14.		2009-2010	Scientific literature
Caries of deciduous teeth	França-Pinto CC, Cenci MS, Correa MB, Romano AR, Peres MA, Peres KG, Matijasevich A, Santos IS, Barros AJD, Demarco FF. Association between black stains and dental caries in primary teeth: findings from a Brazilian population-based birth cohort. . 2012; 46(2): 170-6.		2009	Scientific literature
Caries of deciduous teeth	Carvalho JC, Figueiredo MJ, Vieira EO, Mestrinho HD. Caries trends in Brazilian non-privileged preschool children in 1996 and 2006. . 2009; 43(1): 2-9.		1996-2006	Scientific literature
Caries of deciduous teeth	Dini EL, Holt RD, Bedi R. Caries and its association with infant feeding and oral health-related behaviours in 3-4-year-old Brazilian children. . 2000; 28(4): 241-8.		1998-1999	Scientific literature
Caries of deciduous teeth	Maciel SM, Marcenes W, Sheiham A. The relationship between sweetness preference, levels of salivary mutans streptococci and caries experience in Brazilian pre-school children. . 2001; 11(2): 123-30.		1998-1999	Scientific literature
Caries of deciduous teeth	Bastos RS, Silva RPR, Maia-Junior AF, Carvalho FS, Merlini S, Caldana ML, Lauris JRP, Bastos JRM. Dental caries profile in Monte Negro, Amazonian state of Rondônia, Brazil, in 2008. . 2010; 18(5): 437-41.		2007-2008	Scientific literature

Caries of deciduous teeth	Ferreira SH, Béria JU, Kramer PF, Feldens EG, Feldens CA. Dental caries in 0- to 5-year-old Brazilian children: prevalence, severity, and associated factors. . 2007; 17(4): 289-96.		2005-2006	Scientific literature
Caries of deciduous teeth	Leite IC, Ribeiro RA. Dental caries in the primary dentition in public nursery school children in Juiz de Fora, Minas Gerais, Brazil. . 2000; 16(3): 717-22.		1998	Scientific literature
Caries of deciduous teeth	Mattos-Graner R de O, Rontani RM, Gavião MB, Bocatto HA. Caries prevalence in 6-36-month-old Brazilian children. . 1996; 13(2): 96-8.		1994-1995	Scientific literature
Caries of deciduous teeth	Bönecker M, Marcenes W, Sheiham A. Caries reductions between 1995, 1997 and 1999 in preschool children in Diadema, Brazil. . 2002; 12(3): 183-8.		1995-1999	Scientific literature
Caries of deciduous teeth	Piovesan C, Mendes FM, Ferreira FV, Guedes RS, Ardenghi TM. Socioeconomic inequalities in the distribution of dental caries in Brazilian preschool children. . 2010; 70(4): 319-26.		2008-2009	Scientific literature
Caries of deciduous teeth	Gradella CMF, Bernabé E, Bönecker M, Oliveira LB. Caries prevalence and severity, and quality of life in Brazilian 2- to 4-year-old children. . 2011; 39(6): 498-504.		2008-2010	Scientific literature
Caries of deciduous teeth	Goes PS, Watt R, Hardy RG, Sheiham A. The prevalence and severity of dental pain in 14-15 year old Brazilian schoolchildren. . 2007; 24(4): 217-24.	Pernambuco	2004-2006	Scientific literature
Caries of deciduous teeth	Parisotto TM, Steiner-Oliveira C, De Souza-E-Silva CM, Peres RCR, Rodrigues LKA, Nobre-Dos-Santos M. Assessment of cavitated and active non-cavitated caries lesions in 3- to 4-year-old preschool children: a field study. . 2012; 22(2): 92-9.		2010-2011	Scientific literature
Caries of deciduous teeth	Campos JADB, Melanda EA, Antunes J da S, Foschini ALR. Dental caries and the nutritional status of preschool children: a spatial analysis. . 2011; 16(10): 4161-8.		2006-2007	Scientific literature
Caries of deciduous teeth	Forte FDS, Martins RJ, Saliba Moimaz SA, das Saliba Garbin CA, das Saliba NA. Dental caries in preschool children in Bilac, Brazil. . 2005; 119(6): 556-7.		2003-2004	Scientific literature
Caries of permanent teeth	Bastos RS, Silva RPR, Maia-Junior AF, Carvalho FS, Merlini S, Caldana ML, Lauris JRP, Bastos JRM. Dental caries profile in Monte Negro, Amazonian state of Rondônia, Brazil, in 2008. . 2010; 18(5): 437-41.		2007-2008	Scientific literature
Caries of permanent teeth	Jamelli SR, Rodrigues CS, de Lira PI. Nutritional status and prevalence of dental caries among 12-year-old children at public schools: a case-control study. . 2010; 8(1): 77-84.		2001	Scientific literature
Caries of permanent teeth	Piovesan C, Mendes FM, Antunes JLF, Ardenghi TM. Inequalities in the distribution of dental caries among 12-year-old Brazilian schoolchildren. . 2011; 25(1): 69-75.		2008-2009	Scientific literature
Caries of permanent teeth	Traebert J, Jinbo Y, de Lacerda JT. Association between maternal schooling and caries prevalence: a cross-sectional study in southern Brazil. . 2011; 9(1): 47-52.		2006	Scientific literature
Caries of permanent teeth	Narvai PC, Castellanos RA, Frazão P. Dental caries prevalence in permanent teeth of schoolchildren in Brazil, 1970-1996. . 2000; 34(2): 196-200.		1986-1996	Scientific literature
Caries of permanent teeth	Mendes LGA, Biazevic MGH, Michael-Crosato E, Mendes MOA. Dental caries and associated factors among Brazilian adolescents: a longitudinal study. . 2008; 7(26): 1614-9.		2001-2005	Scientific literature
Caries of permanent teeth	Benazzi AS, da Silva RP, de Meneghim M, Ambrosano GM, Pereira AC. Dental caries and fluorosis prevalence and their relationship with socioeconomic and behavioural variables among 12-year-old schoolchildren. . 2012; 10(1): 65-73.		2007	Scientific literature
Caries of permanent teeth	Assessment of caries experience in 12-year-old adolescents in Piracicaba, Sao Paulo, Brazil. . 2010; 8(4): 361-7.		2008-2009	Scientific literature
Caries of permanent teeth	Pitanga Fernandes ET, Duarte Vargas AM, Oliveira AC, Camargo da Rosa MA, Dutra Lucas S, Ferreira E Ferreira E. Factors related to dental caries in adolescents in southeastern Brazil. . 2010; 11(4): 165-70.		2008	Scientific literature
Caries of permanent teeth	Jeremias F, de Souza JF, Silva CM da C, Cordeiro R de CL, Zuanon ACC, Santos-Pinto L. Dental caries experience and Molar-Incisor Hypomineralization. . 2013; 71(3-4): 870-6.		2011-2012	Scientific literature
Periodontal diseases	Silva-Boghossian CM, Luiz R, Colombo AP. Risk indicators for increased periodontal probing depth in subjects attending a public dental school in Brazil. . 2011; 9(3): 289-99.		2005-2008	Scientific literature
Periodontal diseases	Susin C, Valle P, Oppermann RV, Haugejorden O, Albandar JM. Occurrence and risk indicators of increased probing depth in an adult Brazilian population. . 2005; 32(2): 123-9.		2001	Scientific literature
Periodontal diseases	Dini EL, Guimarães LO. Periodontal conditions and treatment needs (CPITN) in a worker population in Araraquara, SP, Brazil. . 1994; 44(4): 309-11.		1992-1993	Scientific literature
Periodontal diseases	Flores-de-Jacoby L, Bruchmann S, Mengel R, Zafiropoulos GG. Periodontal conditions in Rio de Janeiro City (Brazil) using the CPITN. . 1991; 19(2): 127-8.		1988	Scientific literature
Periodontal diseases	reported measures for prediction of periodontitis in a sample of Brazilians. . 2011; 82(12): 1693-704.		2009-2010	Scientific literature
Periodontal diseases	Susin C, Vecchia CFD, Oppermann RV, Haugejorden O, Albandar JM. Periodontal Attachment Loss in an Urban Population of Brazilian Adults: Effect of Demographic, Behavioral, and Environmental Risk Indicators. . 2004; 75(7): 1033-41.		2001	Scientific literature
Periodontal diseases	Gaio EJ, Haas AN, Carrard VC, Oppermann RV, Albandar J, Susin C. Oral health status in elders from South Brazil: a population-based study. . 2012; 29(3): 214-23.		2009-2010	Scientific literature
Periodontal diseases	Susin C, Haas AN, Valle PM, Oppermann RV, Albandar JM. Prevalence and risk indicators for chronic periodontitis in adolescents and young adults in south Brazil. . 2011; 38(4): 326-33.		2001	Scientific literature
Periodontal diseases	Frias AC, Antunes JLF, Fratucci MVB, Zilbovicius C, Junqueira SR, de Souza SF, Yassui EM. [Population based study on periodontal conditions and socioeconomic determinants in adults in the city of Guarulhos (SP), Brazil, 2006]. . 2011; 14(3): 495-507.		2006	Scientific literature
Edentulism and severe tooth loss	Gaio EJ, Haas AN, Carrard VC, Oppermann RV, Albandar J, Susin C. Oral health status in elders from South Brazil: a population-based study. . 2012; 29(3): 214-23.		2001	Scientific literature
Edentulism and severe tooth loss	Brazil World Health Survey 2003	Country	2003	Survey
Edentulism and severe tooth loss	Susin C, Valle P, Oppermann RV, Haugejorden O, Albandar JM. Occurrence and risk indicators of increased probing depth in an adult Brazilian population. . 2005; 32(2): 123-9.		2001	Scientific literature
Edentulism and severe tooth loss	Paula AMB. Analysis of the normative conditions of oral health, depression and serotonin-transporter-linked promoter region polymorphisms in an elderly population. . 2013; 13(1): 98-106.		2011	Scientific literature
Edentulism and severe tooth loss	Rodrigues SM, Oliveira AC, Vargas AMD, Moreira AN, E Ferreira EF. Implications of edentulism on quality of life among elderly. . 2012; 9(1): 100-9.		2010	Scientific literature
Edentulism and severe tooth loss	Miranda L de P, Silveira MF, Oliveira TL, Alves SFF, Júnior HM, Batista AUD, Bonan PRF. Cognitive impairment, the Mini-Mental State Examination and socio-demographic and dental variables in the elderly in Brazil. . 2012; 29(2): e34-40.		2008-2009	Scientific literature
Edentulism and severe tooth loss	Ribeiro MTF, Rosa MAC da, Lima RMN de, Vargas AMD, Haddad JPA, Ferreira E Ferreira E. Edentulism and shortened dental arch in Brazilian elderly from the National Survey of Oral Health 2003. . 2011; 45(5): 817-23.		2002-2003	Scientific literature
Edentulism and severe tooth loss	Tôrres LH do N, da Silva DD, Neri AL, Hilgert JB, Hugo FN, Sousa M da LR de. Association between underweight and overweight/obesity with oral health among independently living Brazilian elderly. . 2013; 29(1): 152-7.		2008-2009	Scientific literature
Edentulism and severe tooth loss	by oral health problems in adults and the elderly in a southeastern Brazilian city]. . 2012; 17(2): 397-406.		2008	Scientific literature
Edentulism and severe tooth loss	Moreira R da S, Nico LS, Tomita NE. [Spatial risk and factors associated with edentulism among elderly persons in Southeast Brazil]. . 2011; 27(10): 2041-54.		2005	Scientific literature
Edentulism and severe tooth loss	De Andrade FB, Lebrão ML, Santos JLF, Duarte YA de O. Relationship between oral health and frailty in community-dwelling elderly individuals in Brazil. . 2013; 61(5): 809-14.		2006	Scientific literature
Edentulism and severe tooth loss	Brazilian Institute of Geography and Statistics (IBGE), Ministry of Health (Brazil), Secretariat of Health Surveillance, Ministry of Health (Brazil). Brazil National Oral Health Survey 2010. Rio de Janeiro, Brazil: Ministry of Health (Brazil).	Country	2010	Survey
Edentulism and severe tooth loss	De Marchi RJ, Hugo FN, Hilgert JB, Padilha DMP. Association between number of teeth, edentulism and use of dentures with percentage body fat in south Brazilian community-dwelling older people. . 2012; 29(2): e69-76.		2006	Scientific literature
Edentulism and severe tooth loss	Rihs LB, Silva DD da, Sousa M da LR de. Dental caries and tooth loss in adults in a Brazilian southeastern state. . 2009; 17(5): 392-6.		2007	Scientific literature
Edentulism and severe tooth loss	Cardoso EM, Parente RCP, Vettore MV, Rebelo MAB. Oral health conditions of elderly residents in the city of Manaus, Amazonas: estimates by sex. . 2011; 14(1): 131-40.		2007	Scientific literature
Edentulism and severe tooth loss	Corraini P, Baelum V, Pannuti CM, Pustiglioni AN, Romito GA, Pustiglioni FE. Periodontal attachment loss in an untreated isolated population of Brazil. . 2008; 79(4): 610-20.		2005-2006	Scientific literature
Injuries	Centre for Research on the Epidemiology of Disasters (CRED). EM-DAT: The OFDA/CRED International Disaster Database. Brussels, Belgium: Catholic University of Leuven.	Global	1964-2015	Estimate
Road injuries	Ministry of Health (Brazil). Brazil Hospital Information System 1997. Rio de Janeiro, Brazil: Ministry of Health (Brazil).	Country	1993-1997	Administrative record
Road injuries	Ministry of Health (Brazil). Brazil Hospital Information System 2008-2012.		2008-2012	Administrative record
Road injuries	Ministry of Health (Brazil). Brazil Hospital Information System 2013-2014.		2013-2017	Administrative record
Road injuries	Brazilian Institute of Geography and Statistics (IBGE), Ministry of Health (Brazil), Ministry of Planning, Budget, and Management (Brazil). Brazil National Health Survey 2013. Rio de Janeiro, Brazil: Brazilian Institute of Geography and Statistics (IBGE).	Country	2013-2014	Survey
Road injuries	Ministry of Health (Brazil). Brazil Hospital Information System 2003-2007.		2003-2007	Administrative record
Road injuries	Brazil World Health Survey 2003	Country	2002-2003	Survey
Road injuries	Ministry of Health (Brazil). Brazil Hospital Information System 1998-2002.		1998-2002	Administrative record
Road injuries	Health Institute (São Paulo, Brazil), State University of Campinas, São Paulo Municipal Health Department, São Paulo State University, University of São Paulo. Brazil - São Paulo Health Survey 2008-2009.	São Paulo	2002-2008	Survey
Pedestrian road injuries	Ministry of Health (Brazil). Brazil Hospital Information System 2003-2007.		2003-2007	Administrative record

Pedestrian road injuries	Health Institute (São Paulo, Brazil), State University of Campinas, São Paulo Municipal Health Department, São Paulo State University, University of São Paulo. Brazil - São Paulo Health Survey 2008-2009.	São Paulo	2002-2008	Survey
Pedestrian road injuries	Ministry of Health (Brazil). Brazil Hospital Information System 2008-2012.		2008-2012	Administrative record
Pedestrian road injuries	Ministry of Health (Brazil). Brazil Hospital Information System 1997. Rio de Janeiro, Brazil: Ministry of Health (Brazil).	Country	1993-1997	Administrative record
Pedestrian road injuries	Ministry of Health (Brazil). Brazil Hospital Information System 2013-2014.		2013-2017	Administrative record
Pedestrian road injuries	Brazil World Health Survey 2003	Country	2002-2003	Survey
Pedestrian road injuries	Brazilian Institute of Geography and Statistics (IBGE), Ministry of Health (Brazil), Ministry of Planning, Budget, and Management (Brazil). Brazil National Health Survey 2013. Rio de Janeiro, Brazil: Brazilian Institute of Geography and Statistics (IBGE).	Country	2013-2014	Survey
Pedestrian road injuries	Ministry of Health (Brazil). Brazil Hospital Information System 1998-2002.		1998-2002	Administrative record
Cyclist road injuries	Brazilian Institute of Geography and Statistics (IBGE), Ministry of Health (Brazil), Ministry of Planning, Budget, and Management (Brazil). Brazil National Health Survey 2013. Rio de Janeiro, Brazil: Brazilian Institute of Geography and Statistics (IBGE).	Country	2013-2014	Survey
Cyclist road injuries	Ministry of Health (Brazil). Brazil Hospital Information System 1998-2002.		1998-2002	Administrative record
Cyclist road injuries	Ministry of Health (Brazil). Brazil Hospital Information System 2008-2012.		2008-2012	Administrative record
Cyclist road injuries	Brazil World Health Survey 2003	Country	2002-2003	Survey
Cyclist road injuries	Health Institute (São Paulo, Brazil), State University of Campinas, São Paulo Municipal Health Department, São Paulo State University, University of São Paulo. Brazil - São Paulo Health Survey 2008-2009.	São Paulo	2002-2008	Survey
Cyclist road injuries	Ministry of Health (Brazil). Brazil Hospital Information System 2003-2007.		2003-2007	Administrative record
Cyclist road injuries	Ministry of Health (Brazil). Brazil Hospital Information System 2013-2014.		2013-2017	Administrative record
Cyclist road injuries	Ministry of Health (Brazil). Brazil Hospital Information System 1997. Rio de Janeiro, Brazil: Ministry of Health (Brazil).	Country	1993-1997	Administrative record
Motorcyclist road injuries	Ministry of Health (Brazil). Brazil Hospital Information System 2003-2007.		2003-2007	Administrative record
Motorcyclist road injuries	Brazilian Institute of Geography and Statistics (IBGE), Ministry of Health (Brazil), Ministry of Planning, Budget, and Management (Brazil). Brazil National Health Survey 2013. Rio de Janeiro, Brazil: Brazilian Institute of Geography and Statistics (IBGE).	Country	2013-2014	Survey
Motorcyclist road injuries	Ministry of Health (Brazil). Brazil Hospital Information System 1998-2002.		1998-2002	Administrative record
Motorcyclist road injuries	Health Institute (São Paulo, Brazil), State University of Campinas, São Paulo Municipal Health Department, São Paulo State University, University of São Paulo. Brazil - São Paulo Health Survey 2008-2009.	São Paulo	2002-2008	Survey
Motorcyclist road injuries	Ministry of Health (Brazil). Brazil Hospital Information System 2013-2014.		2013-2017	Administrative record
Motorcyclist road injuries	Ministry of Health (Brazil). Brazil Hospital Information System 1997. Rio de Janeiro, Brazil: Ministry of Health (Brazil).	Country	1993-1997	Administrative record
Motorcyclist road injuries	Brazil World Health Survey 2003	Country	2002-2003	Survey
Motorcyclist road injuries	Ministry of Health (Brazil). Brazil Hospital Information System 2008-2012.		2008-2012	Administrative record
Motor vehicle road injuries	Ministry of Health (Brazil). Brazil Hospital Information System 1997. Rio de Janeiro, Brazil: Ministry of Health (Brazil).	Country	1993-1997	Administrative record
Motor vehicle road injuries	Ministry of Health (Brazil). Brazil Hospital Information System 2013-2014.		2013-2017	Administrative record
Motor vehicle road injuries	Ministry of Health (Brazil). Brazil Hospital Information System 2003-2007.		2003-2007	Administrative record
Motor vehicle road injuries	Health Institute (São Paulo, Brazil), State University of Campinas, São Paulo Municipal Health Department, São Paulo State University, University of São Paulo. Brazil - São Paulo Health Survey 2008-2009.	São Paulo	2002-2008	Survey
Motor vehicle road injuries	Brazilian Institute of Geography and Statistics (IBGE), Ministry of Health (Brazil), Ministry of Planning, Budget, and Management (Brazil). Brazil National Health Survey 2013. Rio de Janeiro, Brazil: Brazilian Institute of Geography and Statistics (IBGE).	Country	2013-2014	Survey
Motor vehicle road injuries	Ministry of Health (Brazil). Brazil Hospital Information System 2008-2012.		2008-2012	Administrative record
Motor vehicle road injuries	Ministry of Health (Brazil). Brazil Hospital Information System 1998-2002.		1998-2002	Administrative record
Motor vehicle road injuries	Brazil World Health Survey 2003	Country	2002-2003	Survey
Other road injuries	Brazil World Health Survey 2003	Country	2002-2003	Survey
Other road injuries	Ministry of Health (Brazil). Brazil Hospital Information System 2013-2014.		2013-2017	Administrative record
Other road injuries	Brazilian Institute of Geography and Statistics (IBGE), Ministry of Health (Brazil), Ministry of Planning, Budget, and Management (Brazil). Brazil National Health Survey 2013. Rio de Janeiro, Brazil: Brazilian Institute of Geography and Statistics (IBGE).	Country	2013-2014	Survey
Other road injuries	Ministry of Health (Brazil). Brazil Hospital Information System 2003-2007.		2003-2007	Administrative record
Other road injuries	Ministry of Health (Brazil). Brazil Hospital Information System 1997. Rio de Janeiro, Brazil: Ministry of Health (Brazil).	Country	1993-1997	Administrative record
Other road injuries	Ministry of Health (Brazil). Brazil Hospital Information System 2008-2012.		2008-2012	Administrative record
Other road injuries	Ministry of Health (Brazil). Brazil Hospital Information System 1998-2002.		1998-2002	Administrative record
Other road injuries	Health Institute (São Paulo, Brazil), State University of Campinas, São Paulo Municipal Health Department, São Paulo State University, University of São Paulo. Brazil - São Paulo Health Survey 2008-2009.	São Paulo	2002-2008	Survey
Other transport injuries	Brazilian Institute of Geography and Statistics (IBGE), Ministry of Health (Brazil), Ministry of Planning, Budget, and Management (Brazil). Brazil National Health Survey 2013. Rio de Janeiro, Brazil: Brazilian Institute of Geography and Statistics (IBGE).	Country	2013-2014	Survey
Other transport injuries	Ministry of Health (Brazil). Brazil Hospital Information System 2013-2014.		2013-2017	Administrative record
Other transport injuries	Ministry of Health (Brazil). Brazil Hospital Information System 1998-2002.		1998-2002	Administrative record
Other transport injuries	Brazil World Health Survey 2003	Country	2002-2003	Survey
Other transport injuries	Health Institute (São Paulo, Brazil), State University of Campinas, São Paulo Municipal Health Department, São Paulo State University, University of São Paulo. Brazil - São Paulo Health Survey 2008-2009.	São Paulo	2002-2008	Survey
Other transport injuries	Ministry of Health (Brazil). Brazil Hospital Information System 2003-2007.		2003-2007	Administrative record
Other transport injuries	Ministry of Health (Brazil). Brazil Hospital Information System 1997. Rio de Janeiro, Brazil: Ministry of Health (Brazil).	Country	1993-1997	Administrative record
Other transport injuries	Ministry of Health (Brazil). Brazil Hospital Information System 2008-2012.		2008-2012	Administrative record
Falls	Brazil World Health Survey 2003	Country	2002-2003	Survey
Falls	Health Institute (São Paulo, Brazil), State University of Campinas, São Paulo Municipal Health Department, São Paulo State University, University of São Paulo. Brazil - São Paulo Health Survey 2008-2009.	São Paulo	2002-2008	Survey
Falls	Ministry of Health (Brazil). Brazil Hospital Information System 2008-2012.		2008-2012	Administrative record
Falls	Ministry of Health (Brazil). Brazil Hospital Information System 1997. Rio de Janeiro, Brazil: Ministry of Health (Brazil).	Country	1993-1997	Administrative record
Falls	Ministry of Health (Brazil). Brazil Hospital Information System 2003-2007.		2003-2007	Administrative record
Falls	Ministry of Health (Brazil). Brazil Hospital Information System 2013-2014.		2013-2017	Administrative record
Falls	Ministry of Health (Brazil). Brazil Hospital Information System 1998-2002.		1998-2002	Administrative record
Falls	Brazilian Institute of Geography and Statistics (IBGE), Ministry of Health (Brazil), Ministry of Planning, Budget, and Management (Brazil). Brazil National Health Survey 2013. Rio de Janeiro, Brazil: Brazilian Institute of Geography and Statistics (IBGE).	Country	2013-2014	Survey
Drowning	Brazil World Health Survey 2003	Country	2002-2003	Survey
Drowning	Ministry of Health (Brazil). Brazil Hospital Information System 1997. Rio de Janeiro, Brazil: Ministry of Health (Brazil).	Country	1993-1997	Administrative record
Drowning	Brazilian Institute of Geography and Statistics (IBGE), Ministry of Health (Brazil), Ministry of Planning, Budget, and Management (Brazil). Brazil National Health Survey 2013. Rio de Janeiro, Brazil: Brazilian Institute of Geography and Statistics (IBGE).	Country	2013-2014	Survey
Drowning	Ministry of Health (Brazil). Brazil Hospital Information System 2003-2007.		2003-2007	Administrative record
Drowning	Health Institute (São Paulo, Brazil), State University of Campinas, São Paulo Municipal Health Department, São Paulo State University, University of São Paulo. Brazil - São Paulo Health Survey 2008-2009.	São Paulo	2002-2008	Survey
Drowning	Ministry of Health (Brazil). Brazil Hospital Information System 2008-2012.		2008-2012	Administrative record
Drowning	Ministry of Health (Brazil). Brazil Hospital Information System 1998-2002.		1998-2002	Administrative record
Drowning	Ministry of Health (Brazil). Brazil Hospital Information System 2013-2014.		2013-2017	Administrative record
Fire, heat, and hot substances	Brazil World Health Survey 2003	Country	2002-2003	Survey
Fire, heat, and hot substances	Ministry of Health (Brazil). Brazil Hospital Information System 2013-2014.		2013-2017	Administrative record
Fire, heat, and hot substances	Health Institute (São Paulo, Brazil), State University of Campinas, São Paulo Municipal Health Department, São Paulo State University, University of São Paulo. Brazil - São Paulo Health Survey 2008-2009.	São Paulo	2002-2008	Survey
Fire, heat, and hot substances	Ministry of Health (Brazil). Brazil Hospital Information System 1997. Rio de Janeiro, Brazil: Ministry of Health (Brazil).	Country	1993-1997	Administrative record
Fire, heat, and hot substances	Ministry of Health (Brazil). Brazil Hospital Information System 1998-2002.		1998-2002	Administrative record
Fire, heat, and hot substances	Ministry of Health (Brazil). Brazil Hospital Information System 2003-2007.		2003-2007	Administrative record
Fire, heat, and hot substances	Brazilian Institute of Geography and Statistics (IBGE), Ministry of Health (Brazil), Ministry of Planning, Budget, and Management (Brazil). Brazil National Health Survey 2013. Rio de Janeiro, Brazil: Brazilian Institute of Geography and Statistics (IBGE).	Country	2013-2014	Survey
Fire, heat, and hot substances	Ministry of Health (Brazil). Brazil Hospital Information System 2008-2012.		2008-2012	Administrative record
Poisonings	Brazil World Health Survey 2003	Country	2002-2003	Survey

Poisonings	Ministry of Health (Brazil). Brazil Hospital Information System 1997. Rio de Janeiro, Brazil: Ministry of Health (Brazil).	Country	1993-1997	Administrative record
Poisonings	Ministry of Health (Brazil). Brazil Hospital Information System 2003-2007.		2003-2007	Administrative record
Poisonings	Ministry of Health (Brazil). Brazil Hospital Information System 1998-2002.		1998-2002	Administrative record
Poisonings	Ministry of Health (Brazil). Brazil Hospital Information System 2008-2012.		2008-2012	Administrative record
Poisonings	Brazilian Institute of Geography and Statistics (IBGE), Ministry of Health (Brazil), Ministry of Planning, Budget, and Management (Brazil). Brazil National Health Survey 2013. Rio de Janeiro, Brazil: Brazilian Institute of Geography and Statistics (IBGE).	Country	2013-2014	Survey
Poisonings	Ministry of Health (Brazil). Brazil Hospital Information System 2013-2014.		2013-2017	Administrative record
Poisonings	Health Institute (São Paulo, Brazil), State University of Campinas, São Paulo Municipal Health Department, São Paulo State University, University of São Paulo. Brazil - São Paulo Health Survey 2008-2009.	São Paulo	2002-2008	Survey
Exposure to mechanical forces	Ministry of Health (Brazil). Brazil Hospital Information System 2003-2007.		2003-2007	Administrative record
Exposure to mechanical forces	Brazilian Institute of Geography and Statistics (IBGE), Ministry of Health (Brazil), Ministry of Planning, Budget, and Management (Brazil). Brazil National Health Survey 2013. Rio de Janeiro, Brazil: Brazilian Institute of Geography and Statistics (IBGE).	Country	2013-2014	Survey
Exposure to mechanical forces	Ministry of Health (Brazil). Brazil Hospital Information System 1998-2002.		1998-2002	Administrative record
Exposure to mechanical forces	Ministry of Health (Brazil). Brazil Hospital Information System 2008-2012.		2008-2012	Administrative record
Exposure to mechanical forces	Ministry of Health (Brazil). Brazil Hospital Information System 1997. Rio de Janeiro, Brazil: Ministry of Health (Brazil).	Country	1993-1997	Administrative record
Exposure to mechanical forces	Ministry of Health (Brazil). Brazil Hospital Information System 2013-2014.		2013-2017	Administrative record
Exposure to mechanical forces	Brazil World Health Survey 2003	Country	2002-2003	Survey
Exposure to mechanical forces	Health Institute (São Paulo, Brazil), State University of Campinas, São Paulo Municipal Health Department, São Paulo State University, University of São Paulo. Brazil - São Paulo Health Survey 2008-2009.	São Paulo	2002-2008	Survey
Unintentional firearm injuries	Brazilian Institute of Geography and Statistics (IBGE), Ministry of Health (Brazil), Ministry of Planning, Budget, and Management (Brazil). Brazil National Health Survey 2013. Rio de Janeiro, Brazil: Brazilian Institute of Geography and Statistics (IBGE).	Country	2013-2014	Survey
Unintentional firearm injuries	Health Institute (São Paulo, Brazil), State University of Campinas, São Paulo Municipal Health Department, São Paulo State University, University of São Paulo. Brazil - São Paulo Health Survey 2008-2009.	São Paulo	2002-2008	Survey
Unintentional firearm injuries	Ministry of Health (Brazil). Brazil Hospital Information System 2008-2012.		2008-2012	Administrative record
Unintentional firearm injuries	Ministry of Health (Brazil). Brazil Hospital Information System 1997. Rio de Janeiro, Brazil: Ministry of Health (Brazil).	Country	1993-1997	Administrative record
Unintentional firearm injuries	Ministry of Health (Brazil). Brazil Hospital Information System 2003-2007.		2003-2007	Administrative record
Unintentional firearm injuries	Ministry of Health (Brazil). Brazil Hospital Information System 2013-2014.		2013-2017	Administrative record
Unintentional firearm injuries	Ministry of Health (Brazil). Brazil Hospital Information System 1998-2002.		1998-2002	Administrative record
Unintentional firearm injuries	Brazil World Health Survey 2003	Country	2002-2003	Survey
Unintentional suffocation	Health Institute (São Paulo, Brazil), State University of Campinas, São Paulo Municipal Health Department, São Paulo State University, University of São Paulo. Brazil - São Paulo Health Survey 2008-2009.	São Paulo	2002-2008	Survey
Unintentional suffocation	Brazil World Health Survey 2003	Country	2002-2003	Survey
Unintentional suffocation	Ministry of Health (Brazil). Brazil Hospital Information System 2008-2012.		2008-2012	Administrative record
Unintentional suffocation	Ministry of Health (Brazil). Brazil Hospital Information System 1997. Rio de Janeiro, Brazil: Ministry of Health (Brazil).	Country	1993-1997	Administrative record
Unintentional suffocation	Ministry of Health (Brazil). Brazil Hospital Information System 2003-2007.		2003-2007	Administrative record
Unintentional suffocation	Ministry of Health (Brazil). Brazil Hospital Information System 1998-2002.		1998-2002	Administrative record
Unintentional suffocation	Brazilian Institute of Geography and Statistics (IBGE), Ministry of Health (Brazil), Ministry of Planning, Budget, and Management (Brazil). Brazil National Health Survey 2013. Rio de Janeiro, Brazil: Brazilian Institute of Geography and Statistics (IBGE).	Country	2013-2014	Survey
Unintentional suffocation	Ministry of Health (Brazil). Brazil Hospital Information System 2013-2014.		2013-2017	Administrative record
Other exposure to mechanical forces	Ministry of Health (Brazil). Brazil Hospital Information System 2003-2007.		2003-2007	Administrative record
Other exposure to mechanical forces	Ministry of Health (Brazil). Brazil Hospital Information System 1997. Rio de Janeiro, Brazil: Ministry of Health (Brazil).	Country	1993-1997	Administrative record
Other exposure to mechanical forces	Ministry of Health (Brazil). Brazil Hospital Information System 2008-2012.		2008-2012	Administrative record
Other exposure to mechanical forces	Brazilian Institute of Geography and Statistics (IBGE), Ministry of Health (Brazil), Ministry of Planning, Budget, and Management (Brazil). Brazil National Health Survey 2013. Rio de Janeiro, Brazil: Brazilian Institute of Geography and Statistics (IBGE).	Country	2013-2014	Survey
Other exposure to mechanical forces	Brazil World Health Survey 2003	Country	2002-2003	Survey
Other exposure to mechanical forces	Ministry of Health (Brazil). Brazil Hospital Information System 1998-2002.		1998-2002	Administrative record
Other exposure to mechanical forces	Health Institute (São Paulo, Brazil), State University of Campinas, São Paulo Municipal Health Department, São Paulo State University, University of São Paulo. Brazil - São Paulo Health Survey 2008-2009.	São Paulo	2002-2008	Survey
Other exposure to mechanical forces	Ministry of Health (Brazil). Brazil Hospital Information System 2013-2014.		2013-2017	Administrative record
Adverse effects of medical treatment	Ministry of Health (Brazil). Brazil Hospital Information System 1998-2002.		1998-2002	Administrative record
Adverse effects of medical treatment	Ministry of Health (Brazil). Brazil Hospital Information System 1997. Rio de Janeiro, Brazil: Ministry of Health (Brazil).	Country	1993-1997	Administrative record
Adverse effects of medical treatment	Ministry of Health (Brazil). Brazil Hospital Information System 2013-2014.		2013-2017	Administrative record
Adverse effects of medical treatment	Ministry of Health (Brazil). Brazil Hospital Information System 2008-2012.		2008-2012	Administrative record
Adverse effects of medical treatment	Brazilian Institute of Geography and Statistics (IBGE), Ministry of Health (Brazil), Ministry of Planning, Budget, and Management (Brazil). Brazil National Health Survey 2013. Rio de Janeiro, Brazil: Brazilian Institute of Geography and Statistics (IBGE).	Country	2013-2014	Survey
Adverse effects of medical treatment	Ministry of Health (Brazil). Brazil Hospital Information System 2003-2007.		2003-2007	Administrative record
Adverse effects of medical treatment	Brazil World Health Survey 2003	Country	2002-2003	Survey
Adverse effects of medical treatment	Health Institute (São Paulo, Brazil), State University of Campinas, São Paulo Municipal Health Department, São Paulo State University, University of São Paulo. Brazil - São Paulo Health Survey 2008-2009.	São Paulo	2002-2008	Survey
Animal contact	Ministry of Health (Brazil). Brazil Hospital Information System 2013-2014.		2013-2017	Administrative record
Animal contact	Ministry of Health (Brazil). Brazil Hospital Information System 2008-2012.		2008-2012	Administrative record
Animal contact	Ministry of Health (Brazil). Brazil Hospital Information System 1997. Rio de Janeiro, Brazil: Ministry of Health (Brazil).	Country	1993-1997	Administrative record
Animal contact	Ministry of Health (Brazil). Brazil Hospital Information System 1998-2002.		1998-2002	Administrative record
Animal contact	Health Institute (São Paulo, Brazil), State University of Campinas, São Paulo Municipal Health Department, São Paulo State University, University of São Paulo. Brazil - São Paulo Health Survey 2008-2009.	São Paulo	2002-2008	Survey
Animal contact	Brazilian Institute of Geography and Statistics (IBGE), Ministry of Health (Brazil), Ministry of Planning, Budget, and Management (Brazil). Brazil National Health Survey 2013. Rio de Janeiro, Brazil: Brazilian Institute of Geography and Statistics (IBGE).	Country	2013-2014	Survey
Animal contact	Brazil World Health Survey 2003	Country	2002-2003	Survey
Animal contact	Ministry of Health (Brazil). Brazil Hospital Information System 2003-2007.		2003-2007	Administrative record
Venomous animal contact	Ministry of Health (Brazil). Brazil Hospital Information System 1997. Rio de Janeiro, Brazil: Ministry of Health (Brazil).	Country	1993-1997	Administrative record
Venomous animal contact	Brazil World Health Survey 2003	Country	2002-2003	Survey
Venomous animal contact	Ministry of Health (Brazil). Brazil Hospital Information System 2008-2012.		2008-2012	Administrative record
Venomous animal contact	Brazilian Institute of Geography and Statistics (IBGE), Ministry of Health (Brazil), Ministry of Planning, Budget, and Management (Brazil). Brazil National Health Survey 2013. Rio de Janeiro, Brazil: Brazilian Institute of Geography and Statistics (IBGE).	Country	2013-2014	Survey
Venomous animal contact	Ministry of Health (Brazil). Brazil Hospital Information System 2013-2014.		2013-2017	Administrative record
Venomous animal contact	Ministry of Health (Brazil). Brazil Hospital Information System 1998-2002.		1998-2002	Administrative record
Venomous animal contact	Health Institute (São Paulo, Brazil), State University of Campinas, São Paulo Municipal Health Department, São Paulo State University, University of São Paulo. Brazil - São Paulo Health Survey 2008-2009.	São Paulo	2002-2008	Survey
Venomous animal contact	Ministry of Health (Brazil). Brazil Hospital Information System 2003-2007.		2003-2007	Administrative record
Non-venomous animal contact	Health Institute (São Paulo, Brazil), State University of Campinas, São Paulo Municipal Health Department, São Paulo State University, University of São Paulo. Brazil - São Paulo Health Survey 2008-2009.	São Paulo	2002-2008	Survey
Non-venomous animal contact	Ministry of Health (Brazil). Brazil Hospital Information System 1998-2002.		1998-2002	Administrative record
Non-venomous animal contact	Brazil World Health Survey 2003	Country	2002-2003	Survey
Non-venomous animal contact	Ministry of Health (Brazil). Brazil Hospital Information System 2008-2012.		2008-2012	Administrative record
Non-venomous animal contact	Ministry of Health (Brazil). Brazil Hospital Information System 2013-2014.		2013-2017	Administrative record
Non-venomous animal contact	Ministry of Health (Brazil). Brazil Hospital Information System 1997. Rio de Janeiro, Brazil: Ministry of Health (Brazil).	Country	1993-1997	Administrative record
Non-venomous animal contact	Brazilian Institute of Geography and Statistics (IBGE), Ministry of Health (Brazil), Ministry of Planning, Budget, and Management (Brazil). Brazil National Health Survey 2013. Rio de Janeiro, Brazil: Brazilian Institute of Geography and Statistics (IBGE).	Country	2013-2014	Survey
Non-venomous animal contact	Ministry of Health (Brazil). Brazil Hospital Information System 2003-2007.		2003-2007	Administrative record
Pulmonary aspiration and foreign body in airway	Ministry of Health (Brazil). Brazil Hospital Information System 2003-2007.		2003-2007	Administrative record

Pulmonary aspiration and foreign body in airway	Ministry of Health (Brazil). Brazil Hospital Information System 1997. Rio de Janeiro, Brazil: Ministry of Health (Brazil).	Country	1993-1997	Administrative record
Pulmonary aspiration and foreign body in airway	Ministry of Health (Brazil). Brazil Hospital Information System 2008-2012.		2008-2012	Administrative record
Pulmonary aspiration and foreign body in airway	Ministry of Health (Brazil). Brazil Hospital Information System 1998-2002.		1998-2002	Administrative record
Pulmonary aspiration and foreign body in airway	Health Institute (São Paulo, Brazil), State University of Campinas, São Paulo Municipal Health Department, São Paulo State University, University of São Paulo. Brazil - São Paulo Health Survey 2008-2009.	São Paulo	2002-2008	Survey
Pulmonary aspiration and foreign body in airway	Brazil World Health Survey 2003	Country	2002-2003	Survey
Pulmonary aspiration and foreign body in airway	Brazilian Institute of Geography and Statistics (IBGE), Ministry of Health (Brazil), Ministry of Planning, Budget, and Management (Brazil). Brazil National Health Survey 2013. Rio de Janeiro, Brazil: Brazilian Institute of Geography and Statistics (IBGE).	Country	2013-2014	Survey
Pulmonary aspiration and foreign body in airway	Ministry of Health (Brazil). Brazil Hospital Information System 2013-2014.		2013-2017	Administrative record
Foreign body in eyes	Ministry of Health (Brazil). Brazil Hospital Information System 2003-2007.		2003-2007	Administrative record
Foreign body in eyes	Ministry of Health (Brazil). Brazil Hospital Information System 1998-2002.		1998-2002	Administrative record
Foreign body in eyes	Brazil World Health Survey 2003	Country	2002-2003	Survey
Foreign body in eyes	Brazilian Institute of Geography and Statistics (IBGE), Ministry of Health (Brazil), Ministry of Planning, Budget, and Management (Brazil). Brazil National Health Survey 2013. Rio de Janeiro, Brazil: Brazilian Institute of Geography and Statistics (IBGE).	Country	2013-2014	Survey
Foreign body in eyes	Health Institute (São Paulo, Brazil), State University of Campinas, São Paulo Municipal Health Department, São Paulo State University, University of São Paulo. Brazil - São Paulo Health Survey 2008-2009.	São Paulo	2002-2008	Survey
Foreign body in eyes	Ministry of Health (Brazil). Brazil Hospital Information System 2008-2012.		2008-2012	Administrative record
Foreign body in eyes	Ministry of Health (Brazil). Brazil Hospital Information System 1997. Rio de Janeiro, Brazil: Ministry of Health (Brazil).	Country	1993-1997	Administrative record
Foreign body in eyes	Ministry of Health (Brazil). Brazil Hospital Information System 2013-2014.		2013-2017	Administrative record
Foreign body in other body part	Ministry of Health (Brazil). Brazil Hospital Information System 2003-2007.		2003-2007	Administrative record
Foreign body in other body part	Brazilian Institute of Geography and Statistics (IBGE), Ministry of Health (Brazil), Ministry of Planning, Budget, and Management (Brazil). Brazil National Health Survey 2013. Rio de Janeiro, Brazil: Brazilian Institute of Geography and Statistics (IBGE).	Country	2013-2014	Survey
Foreign body in other body part	Ministry of Health (Brazil). Brazil Hospital Information System 2013-2014.		2013-2017	Administrative record
Foreign body in other body part	Health Institute (São Paulo, Brazil), State University of Campinas, São Paulo Municipal Health Department, São Paulo State University, University of São Paulo. Brazil - São Paulo Health Survey 2008-2009.	São Paulo	2002-2008	Survey
Foreign body in other body part	Ministry of Health (Brazil). Brazil Hospital Information System 2008-2012.		2008-2012	Administrative record
Foreign body in other body part	Ministry of Health (Brazil). Brazil Hospital Information System 1997. Rio de Janeiro, Brazil: Ministry of Health (Brazil).	Country	1993-1997	Administrative record
Foreign body in other body part	Ministry of Health (Brazil). Brazil Hospital Information System 1998-2002.		1998-2002	Administrative record
Foreign body in other body part	Brazil World Health Survey 2003	Country	2002-2003	Survey
Environmental heat and cold exposure	Brazilian Institute of Geography and Statistics (IBGE), Ministry of Health (Brazil), Ministry of Planning, Budget, and Management (Brazil). Brazil National Health Survey 2013. Rio de Janeiro, Brazil: Brazilian Institute of Geography and Statistics (IBGE).	Country	2013-2014	Survey
Environmental heat and cold exposure	Health Institute (São Paulo, Brazil), State University of Campinas, São Paulo Municipal Health Department, São Paulo State University, University of São Paulo. Brazil - São Paulo Health Survey 2008-2009.	São Paulo	2002-2008	Survey
Environmental heat and cold exposure	Ministry of Health (Brazil). Brazil Hospital Information System 1997. Rio de Janeiro, Brazil: Ministry of Health (Brazil).	Country	1993-1997	Administrative record
Environmental heat and cold exposure	Ministry of Health (Brazil). Brazil Hospital Information System 2008-2012.		2008-2012	Administrative record
Environmental heat and cold exposure	Ministry of Health (Brazil). Brazil Hospital Information System 1998-2002.		1998-2002	Administrative record
Environmental heat and cold exposure	Ministry of Health (Brazil). Brazil Hospital Information System 2013-2014.		2013-2017	Administrative record
Environmental heat and cold exposure	Brazil World Health Survey 2003	Country	2002-2003	Survey
Environmental heat and cold exposure	Ministry of Health (Brazil). Brazil Hospital Information System 2003-2007.		2003-2007	Administrative record
Other unintentional injuries	Ministry of Health (Brazil). Brazil Hospital Information System 1997. Rio de Janeiro, Brazil: Ministry of Health (Brazil).	Country	1993-1997	Administrative record
Other unintentional injuries	Ministry of Health (Brazil). Brazil Hospital Information System 2013-2014.		2013-2017	Administrative record
Other unintentional injuries	Ministry of Health (Brazil). Brazil Hospital Information System 2008-2012.		2008-2012	Administrative record
Other unintentional injuries	Brazilian Institute of Geography and Statistics (IBGE), Ministry of Health (Brazil), Ministry of Planning, Budget, and Management (Brazil). Brazil National Health Survey 2013. Rio de Janeiro, Brazil: Brazilian Institute of Geography and Statistics (IBGE).	Country	2013-2014	Survey
Other unintentional injuries	Ministry of Health (Brazil). Brazil Hospital Information System 1998-2002.		1998-2002	Administrative record
Other unintentional injuries	Ministry of Health (Brazil). Brazil Hospital Information System 2003-2007.		2003-2007	Administrative record
Other unintentional injuries	Health Institute (São Paulo, Brazil), State University of Campinas, São Paulo Municipal Health Department, São Paulo State University, University of São Paulo. Brazil - São Paulo Health Survey 2008-2009.	São Paulo	2002-2008	Survey
Other unintentional injuries	Brazil World Health Survey 2003	Country	2002-2003	Survey
Self-harm	Brazilian Institute of Geography and Statistics (IBGE), Ministry of Health (Brazil), Ministry of Planning, Budget, and Management (Brazil). Brazil National Health Survey 2013. Rio de Janeiro, Brazil: Brazilian Institute of Geography and Statistics (IBGE).	Country	2013-2014	Survey
Self-harm	Ministry of Health (Brazil). Brazil Hospital Information System 2013-2014.		2013-2017	Administrative record
Self-harm	Ministry of Health (Brazil). Brazil Hospital Information System 1998-2002.		1998-2002	Administrative record
Self-harm	Ministry of Health (Brazil). Brazil Hospital Information System 2008-2012.		2008-2012	Administrative record
Self-harm	Health Institute (São Paulo, Brazil), State University of Campinas, São Paulo Municipal Health Department, São Paulo State University, University of São Paulo. Brazil - São Paulo Health Survey 2008-2009.	São Paulo	2002-2008	Survey
Self-harm	Ministry of Health (Brazil). Brazil Hospital Information System 1997. Rio de Janeiro, Brazil: Ministry of Health (Brazil).	Country	1993-1997	Administrative record
Self-harm	Brazil World Health Survey 2003	Country	2002-2003	Survey
Self-harm	Ministry of Health (Brazil). Brazil Hospital Information System 2003-2007.		2003-2007	Administrative record
Self-harm by firearm	Ministry of Health (Brazil). Brazil Hospital Information System 2013-2014.		2013-2017	Administrative record
Self-harm by firearm	Brazilian Institute of Geography and Statistics (IBGE), Ministry of Health (Brazil), Ministry of Planning, Budget, and Management (Brazil). Brazil National Health Survey 2013. Rio de Janeiro, Brazil: Brazilian Institute of Geography and Statistics (IBGE).	Country	2013-2014	Survey
Self-harm by firearm	Brazil World Health Survey 2003	Country	2002-2003	Survey
Self-harm by firearm	Ministry of Health (Brazil). Brazil Hospital Information System 1997. Rio de Janeiro, Brazil: Ministry of Health (Brazil).	Country	1993-1997	Administrative record
Self-harm by firearm	Ministry of Health (Brazil). Brazil Hospital Information System 1998-2002.		1998-2002	Administrative record
Self-harm by firearm	Health Institute (São Paulo, Brazil), State University of Campinas, São Paulo Municipal Health Department, São Paulo State University, University of São Paulo. Brazil - São Paulo Health Survey 2008-2009.	São Paulo	2002-2008	Survey
Self-harm by firearm	Ministry of Health (Brazil). Brazil Hospital Information System 2008-2012.		2008-2012	Administrative record
Self-harm by firearm	Ministry of Health (Brazil). Brazil Hospital Information System 2003-2007.		2003-2007	Administrative record
Self-harm by other specified means	Ministry of Health (Brazil). Brazil Hospital Information System 1998-2002.		1998-2002	Administrative record
Self-harm by other specified means	Brazil World Health Survey 2003	Country	2002-2003	Survey
Self-harm by other specified means	Ministry of Health (Brazil). Brazil Hospital Information System 2013-2014.		2013-2017	Administrative record
Self-harm by other specified means	Ministry of Health (Brazil). Brazil Hospital Information System 2003-2007.		2003-2007	Administrative record
Self-harm by other specified means	Ministry of Health (Brazil). Brazil Hospital Information System 2008-2012.		2008-2012	Administrative record
Self-harm by other specified means	Health Institute (São Paulo, Brazil), State University of Campinas, São Paulo Municipal Health Department, São Paulo State University, University of São Paulo. Brazil - São Paulo Health Survey 2008-2009.	São Paulo	2002-2008	Survey
Self-harm by other specified means	Brazilian Institute of Geography and Statistics (IBGE), Ministry of Health (Brazil), Ministry of Planning, Budget, and Management (Brazil). Brazil National Health Survey 2013. Rio de Janeiro, Brazil: Brazilian Institute of Geography and Statistics (IBGE).	Country	2013-2014	Survey
Self-harm by other specified means	Ministry of Health (Brazil). Brazil Hospital Information System 1997. Rio de Janeiro, Brazil: Ministry of Health (Brazil).	Country	1993-1997	Administrative record
Interpersonal violence	Ministry of Health (Brazil). Brazil Hospital Information System 2008-2012.		2008-2012	Administrative record
Interpersonal violence	Ministry of Health (Brazil). Brazil Hospital Information System 2013-2014.		2013-2017	Administrative record
Interpersonal violence	Ministry of Health (Brazil). Brazil Hospital Information System 2003-2007.		2003-2007	Administrative record
Interpersonal violence	Brazilian Institute of Geography and Statistics (IBGE), Ministry of Health (Brazil), Ministry of Planning, Budget, and Management (Brazil). Brazil National Health Survey 2013. Rio de Janeiro, Brazil: Brazilian Institute of Geography and Statistics (IBGE).	Country	2013-2014	Survey
Interpersonal violence	Ministry of Health (Brazil). Brazil Hospital Information System 1998-2002.		1998-2002	Administrative record
Interpersonal violence	Brazil World Health Survey 2003	Country	2002-2003	Survey
Interpersonal violence	Ministry of Health (Brazil). Brazil Hospital Information System 1997. Rio de Janeiro, Brazil: Ministry of Health (Brazil).	Country	1993-1997	Administrative record
Interpersonal violence	Health Institute (São Paulo, Brazil), State University of Campinas, São Paulo Municipal Health Department, São Paulo State University, University of São Paulo. Brazil - São Paulo Health Survey 2008-2009.	São Paulo	2002-2008	Survey
Physical violence by firearm	Ministry of Health (Brazil). Brazil Hospital Information System 1998-2002.		1998-2002	Administrative record
Physical violence by firearm	Ministry of Health (Brazil). Brazil Hospital Information System 2008-2012.		2008-2012	Administrative record

Physical violence by firearm	Brazilian Institute of Geography and Statistics (IBGE), Ministry of Health (Brazil), Ministry of Planning, Budget, and Management (Brazil). Brazil National Health Survey 2013. Rio de Janeiro, Brazil: Brazilian Institute of Geography and Statistics (IBGE).	Country	2013-2014	Survey
Physical violence by firearm	Ministry of Health (Brazil). Brazil Hospital Information System 2003-2007.		2003-2007	Administrative record
Physical violence by firearm	Ministry of Health (Brazil). Brazil Hospital Information System 2013-2014.		2013-2017	Administrative record
Physical violence by firearm	Ministry of Health (Brazil). Brazil Hospital Information System 1997. Rio de Janeiro, Brazil: Ministry of Health (Brazil).	Country	1993-1997	Administrative record
Physical violence by firearm	Health Institute (São Paulo, Brazil), State University of Campinas, São Paulo Municipal Health Department, São Paulo State University, University of São Paulo. Brazil - São Paulo Health Survey 2008-2009.	São Paulo	2002-2008	Survey
Physical violence by firearm	Brazil World Health Survey 2003	Country	2002-2003	Survey
Physical violence by sharp object	Ministry of Health (Brazil). Brazil Hospital Information System 2003-2007.		2003-2007	Administrative record
Physical violence by sharp object	Ministry of Health (Brazil). Brazil Hospital Information System 2013-2014.		2013-2017	Administrative record
Physical violence by sharp object	Ministry of Health (Brazil). Brazil Hospital Information System 1997. Rio de Janeiro, Brazil: Ministry of Health (Brazil).	Country	1993-1997	Administrative record
Physical violence by sharp object	Ministry of Health (Brazil). Brazil Hospital Information System 2008-2012.		2008-2012	Administrative record
Physical violence by sharp object	Ministry of Health (Brazil). Brazil Hospital Information System 1998-2002.		1998-2002	Administrative record
Physical violence by sharp object	Brazil World Health Survey 2003	Country	2002-2003	Survey
Physical violence by sharp object	Brazilian Institute of Geography and Statistics (IBGE), Ministry of Health (Brazil), Ministry of Planning, Budget, and Management (Brazil). Brazil National Health Survey 2013. Rio de Janeiro, Brazil: Brazilian Institute of Geography and Statistics (IBGE).	Country	2013-2014	Survey
Physical violence by sharp object	Health Institute (São Paulo, Brazil), State University of Campinas, São Paulo Municipal Health Department, São Paulo State University, University of São Paulo. Brazil - São Paulo Health Survey 2008-2009.	São Paulo	2002-2008	Survey
Physical violence by other means	Brazilian Institute of Geography and Statistics (IBGE), Ministry of Health (Brazil), Ministry of Planning, Budget, and Management (Brazil). Brazil National Health Survey 2013. Rio de Janeiro, Brazil: Brazilian Institute of Geography and Statistics (IBGE).	Country	2013-2014	Survey
Physical violence by other means	Ministry of Health (Brazil). Brazil Hospital Information System 2003-2007.		2003-2007	Administrative record
Physical violence by other means	Brazil World Health Survey 2003	Country	2002-2003	Survey
Physical violence by other means	Health Institute (São Paulo, Brazil), State University of Campinas, São Paulo Municipal Health Department, São Paulo State University, University of São Paulo. Brazil - São Paulo Health Survey 2008-2009.	São Paulo	2002-2008	Survey
Physical violence by other means	Ministry of Health (Brazil). Brazil Hospital Information System 2008-2012.		2008-2012	Administrative record
Physical violence by other means	Ministry of Health (Brazil). Brazil Hospital Information System 1998-2002.		1998-2002	Administrative record
Physical violence by other means	Ministry of Health (Brazil). Brazil Hospital Information System 2013-2014.		2013-2017	Administrative record
Physical violence by other means	Ministry of Health (Brazil). Brazil Hospital Information System 1997. Rio de Janeiro, Brazil: Ministry of Health (Brazil).	Country	1993-1997	Administrative record
None	Brazil World Health Survey 2003	Country	2002-2003	Survey
None	World Health Organization (WHO). WHO Tuberculosis Case Notifications. Geneva, Switzerland: World Health Organization (WHO).	Global	2003-2014	Epi surveillance
None	Brazilian Institute of Geography and Statistics (IBGE), Ministry of Health (Brazil), Ministry of Planning, Budget, and Management (Brazil). Brazil National Health Survey 2013. Rio de Janeiro, Brazil: Brazilian Institute of Geography and Statistics (IBGE).	Country	2013-2014	Survey
None	Health Institute (São Paulo, Brazil), State University of Campinas, São Paulo Municipal Health Department, São Paulo State University, University of São Paulo. Brazil - São Paulo Health Survey 2008-2009.	São Paulo	2002-2008	Survey

Appendix Table 3: Data used in Brazil risk estimates, GBD 2016				
Risk	Citation	Coverage	Years	Data Type
Unsafe sanitation	Brazilian Society for Family Welfare (BEMFAM), Westinghouse; Institute for Resource Development. Brazil Demographic and Health Survey 1986. Columbia, United States: Westinghouse; Institute for Resource Development.	Country	1986	Survey
Unsafe sanitation	Brazilian Institute of Geography and Statistics (IBGE). Brazil National Household Sample Survey 2013. Rio de Janeiro, Brazil: Brazilian Institute of Geography and	Country	2013	Survey
Unsafe sanitation	Brazilian Institute of Geography and Statistics (IBGE). Brazil National Household Sample Survey 2012. Rio de Janeiro, Brazil: Brazilian Institute of Geography and	Country	2012	Survey
Unsafe sanitation	Brazilian Institute of Geography and Statistics (IBGE). Brazil National Household Sample Survey 2011. Rio de Janeiro, Brazil: Brazilian Institute of Geography and	Country	2011	Survey
Unsafe sanitation	Brazil Demographic Census 2010 - IPUMS	Country	2010	Census
Unsafe sanitation	Brazil General Census 1980 - IPUMS	Country	1980	Census
Unsafe sanitation	Brazil General Census 1991 - IPUMS	Country	1991	Census
Unsafe sanitation	Brazil World Health Survey 2003	Country	2003	Survey
Unsafe sanitation	Brazilian Society for Family Welfare (BEMFAM), Macro International, Inc. Brazil Demographic and Health Survey 1996. Calverton, United States: Macro	Country	1996	Survey
Unsafe sanitation	Brazilian Society for Family Welfare (BEMFAM), Macro International, Inc. Brazil Demographic and Health Survey 1991. Calverton, United States: Macro	Country	1991	Survey
Unsafe sanitation	Brazil General Census 2000 - IPUMS	Country	2000	Census
Air pollution	Brazil Air Quality Platform Database 2000-2014 as it appears in World Health Organization (WHO). WHO Urban Ambient Air Pollution Database Draft 2016.	Country	2014	Natural phenomena
Air pollution	Brazil - Curitiba Metropolitan Area Air Quality Annual Report 2012 as it appears in World Health Organization (WHO). WHO Urban Ambient Air Pollution	Paraná	2012	Report
Ambient particulate matter pollution	Brazil Air Quality Platform Database 2000-2014 as it appears in World Health Organization (WHO). WHO Urban Ambient Air Pollution Database Draft 2016.	Country	2014	Natural phenomena
Ambient particulate matter pollution	Brazil - Curitiba Metropolitan Area Air Quality Annual Report 2012 as it appears in World Health Organization (WHO). WHO Urban Ambient Air Pollution	Paraná	2012	Report
Household air pollution from solid fuels	Brazil World Health Survey 2003	Country	2003	Survey
Household air pollution from solid fuels	Brazil General Census 1991 - IPUMS	Country	1991	Census
Household air pollution from solid fuels	Brazil General Census 1980 - IPUMS	Country	1980	Census
Household air pollution from solid fuels	Brazil Living Standards Measurement Survey 1996-1997	Ceará, Espírito Santo, Maranhão, Minas Gerais, Paraíba, Pernambuco, Piaui, Rio Grande do orte, Rio de Janeiro, Sergipe, São Paulo, Alagoas, Bahia, Ceará, Espírito Santo, Maranhão, Minas Gerais,	1996	Survey
Household air pollution from solid fuels	Brazilian Institute of Geography and Statistics (IBGE). Brazil National Household Sample Survey 1999. Rio de Janeiro, Brazil: Brazilian Institute of Geography and	Country	1999	Survey
Residential radon	Paulo SR, Osorio AM, Aparecido R, Rodríguez C, Moreno V, Vasquez R, Espinosa G, Golzarri JI, Martínez T, Navarrete M, Cabrera I, Segovia N, Peña P, Taméz E, Pereyra P,López-Herrera ME, López-Herrera ME, Sajo-Bohus L. Indoor radon measurements in six Latin American countries. . 2002; 41(4): 453-7.		2000-2005	Scientific literature
Lead exposure	de Araujo GC, Mourao NT, Pinheiro IN, Xavier AR, Gameiro VS. Lead Toxicity Risks in Gunshot Victims. . 2015; 10(10): e0140220.		2014	Scientific literature
Lead exposure	Associated factors for higher lead and cadmium blood levels, and reference values derived from general population of Sao Paulo, Brazil. . 2016; 543(Pt A): 628-35.		2007	Scientific literature
Lead exposure	Carvalho SR, Alvarenga AL, Rezende MI, Figueiroa GA, Leite VG, Gutierrez AC, Lobo BC, Cascales RA. [Reference values for lead in blood in urban population in southern Brazil]. . 2001; 9(5): 315-9.		1995	Scientific literature
Lead exposure	de Araujo GC, Mourao NT, Pinheiro IN, Xavier AR, Gameiro VS. Lead Toxicity Risks in Gunshot Victims. . 2015; 10(10): e0140220.		2014	Scientific literature
Lead exposure	Ferron MM, Lima AK de, Saldiva PHN, Gouveia N. Environmental lead poisoning among children in Porto Alegre state, Southern Brazil. . 2012; 46(2): 226-33.		2006	Scientific literature
Lead exposure	Freire C, Koifman RJ, Fujimoto D, de Oliveira Souza VC, Barbosa F, Koifman S. Reference values of lead in blood and related factors among blood donors in the Western Amazon, Brazil. . 2014; 77(8): 426-40.		2010	Scientific literature
Lead exposure	Moura M, Goncalves Valente J. Blood lead levels during pregnancy in women living in Rio de Janeiro, Brazil. . 2002; 299(1-3): 123-9.		1996	Scientific literature
Lead exposure	Ferron MM, Lima AK de, Saldiva PHN, Gouveia N. Environmental lead poisoning among children in Porto Alegre state, Southern Brazil. . 2012; 46(2): 226-33.		2006	Scientific literature
Lead exposure	Freire C, Koifman RJ, Fujimoto D, de Oliveira Souza VC, Barbosa F, Koifman S. Reference values of lead in blood and related factors among blood donors in the Western Amazon, Brazil. . 2014; 77(8): 426-40.		2010	Scientific literature

Lead exposure	Associated factors for higher lead and cadmium blood levels, and reference values derived from general population of Sao Paulo, Brazil. . 2016; 543(Pt A): 628-35.		2007	Scientific literature
Lead exposure	Romieu I, Lacasana M, McConnell R. Lead exposure in Latin America and the Caribbean. Lead Research Group of the Pan-American Health Organization. .		1983	Scientific literature
Lead exposure	Paoliello MMB, De Capitani EM. Occupational and environmental human lead exposure in Brazil. . 2007; 103(2): 288-97.		1998	Scientific literature
Lead exposure	Paoliello MMB, De Capitani EM. Occupational and environmental human lead exposure in Brazil. . 2007; 103(2): 288-97.		1998	Scientific literature
Lead exposure	Cordeiro R, Lima Filho EC, Salgado PE, Santos CO, Constantino L, Malatesta ML. [Neurological disorders in workers with low levels of lead in the blood. II--Neuropsychological disorders]. . 1996; 30(4): 358-63.		1996	Scientific literature
Lead exposure	Cordeiro R, Lima Filho EC, Salgado PE, Santos CO, Constantino L, Malatesta ML. [Neurological disorders in workers with low levels of lead in the blood. II--Neuropsychological disorders]. . 1996; 30(4): 358-63.		1996	Scientific literature
Lead exposure	Dos Santos AC, Colacciopo S, Dal Bó CM, dos Santos NA. Occupational exposure to lead, kidney function tests, and blood pressure. . 1994; 26(5): 635-43.		1994	Scientific literature
Lead exposure	Dos Santos AC, Colacciopo S, Dal Bó CM, dos Santos NA. Occupational exposure to lead, kidney function tests, and blood pressure. . 1994; 26(5): 635-43.		1994	Scientific literature
Lead exposure	Nogueira DP, Colacioppo S, de Souza JM, Pezza CB, de Souza ML, Gomes JR. [Lead level in a sample of "non-exposed" volunteers living in greater São Paulo,		1976	Scientific literature
Lead exposure	Nogueira DP, Colacioppo S, de Souza JM, Pezza CB, de Souza ML, Gomes JR. [Lead level in a sample of "non-exposed" volunteers living in greater São Paulo,		1976	Scientific literature
Lead exposure	Romieu I, Lacasana M, McConnell R. Lead exposure in Latin America and the Caribbean. Lead Research Group of the Pan-American Health Organization. .		1983	Scientific literature
Lead exposure	de Almeida Lopes AC, Navas-Acien A, Zamoiski R, Silbergeld EK, Carvalho Mde F, Buzzo ML, Urbano MR, Martins Ada C, Paoliello MM. Risk factors for lead exposure in adult population in southern Brazil. . 2015; 78(2): 92-108.		2011	Scientific literature
Lead exposure	de Almeida Lopes AC, Navas-Acien A, Zamoiski R, Silbergeld EK, Carvalho Mde F, Buzzo ML, Urbano MR, Martins Ada C, Paoliello MM. Risk factors for lead exposure in adult population in southern Brazil. . 2015; 78(2): 92-108.		2011	Scientific literature
Lead exposure	Moura M, Goncalves Valente J. Blood lead levels during pregnancy in women living in Rio de Janeiro, Brazil. . 2002; 299(1-3): 123-9.		1996	Scientific literature
Lead exposure	Alvarenga AL, Carvalho SR, Figueiroa GA, Leite VG, Gutierrez AC, Nogueira KB, Inamine WA, Zavatti AM. [Reference values for lead levels in blood for the urban population]. . 1997; 31(2): 144-8.		1994	Scientific literature
Lead exposure	Alvarenga AL, Carvalho SR, Figueiroa GA, Leite VG, Gutierrez AC, Nogueira KB, Inamine WA, Zavatti AM. [Reference values for lead levels in blood for the urban population]. . 1997; 31(2): 144-8.		1994	Scientific literature
Lead exposure	Carvalho SR, Alvarenga AL, Rezende MI, Figueiroa GA, Leite VG, Gutierrez AC, Lobo BC, Cascales RA. [Reference values for lead in blood in urban population in southern Brazil]. . 2001; 9(5): 315-9.		1995	Scientific literature
Occupational risks	International Labour Organization (ILO). International Labour Organization Database (ILOSTAT) - Employment by Sex and Economic Activity. International	Global	1981-2014	Estimate
Occupational risks	International Labour Organization (ILO). International Labour Organization Database (ILOSTAT) - Employment to Population Ratio. International Labour	Global	2001-2015	Estimate
Occupational carcinogens	International Labour Organization (ILO). International Labour Organization Database (ILOSTAT) - Employment to Population Ratio. International Labour	Global	2001-2015	Estimate
Occupational carcinogens	L, Prüss-Ustün A, Leigh J, Corvalan C. The global burden of selected occupational diseases and injury risks: Methodology and summary. . 2005; 48(6): 400-18.	Global	1990-2010	Scientific literature
Occupational carcinogens	International Labour Organization (ILO). International Labour Organization Database (ILOSTAT) - Employment by Sex and Economic Activity. International	Global	1981-2014	Estimate
Occupational exposure to asbestos	International Labour Organization (ILO). International Labour Organization Database (ILOSTAT) - Employment by Sex and Economic Activity. International	Global	1981-2014	Estimate
Occupational exposure to asbestos	International Labour Organization (ILO). International Labour Organization Database (ILOSTAT) - Employment by Sex and Occupation. International Labour	Global	2000-2009	Estimate
Occupational exposure to arsenic	International Labour Organization (ILO). International Labour Organization Database (ILOSTAT) - Employment by Sex and Economic Activity. International	Global	1981-2014	Estimate
Occupational exposure to arsenic	International Labour Organization (ILO). International Labour Organization Database (ILOSTAT) - Employment by Sex and Occupation. International Labour	Global	2000-2009	Estimate
Occupational exposure to benzene	International Labour Organization (ILO). International Labour Organization Database (ILOSTAT) - Employment by Sex and Economic Activity. International	Global	1981-2014	Estimate
Occupational exposure to benzene	International Labour Organization (ILO). International Labour Organization Database (ILOSTAT) - Employment by Sex and Occupation. International Labour	Global	2000-2009	Estimate
Occupational exposure to beryllium	International Labour Organization (ILO). International Labour Organization Database (ILOSTAT) - Employment by Sex and Economic Activity. International	Global	1981-2014	Estimate
Occupational exposure to beryllium	International Labour Organization (ILO). International Labour Organization Database (ILOSTAT) - Employment by Sex and Occupation. International Labour	Global	2000-2009	Estimate
Occupational exposure to cadmium	International Labour Organization (ILO). International Labour Organization Database (ILOSTAT) - Employment by Sex and Economic Activity. International	Global	1981-2014	Estimate
Occupational exposure to cadmium	International Labour Organization (ILO). International Labour Organization Database (ILOSTAT) - Employment by Sex and Occupation. International Labour	Global	2000-2009	Estimate

Occupational exposure to chromium	International Labour Organization (ILO). International Labour Organization Database (ILOSTAT) - Employment by Sex and Occupation. International Labour	Global	2000-2009	Estimate
Occupational exposure to chromium	International Labour Organization (ILO). International Labour Organization Database (ILOSTAT) - Employment by Sex and Economic Activity. International	Global	1981-2014	Estimate
Occupational exposure to diesel engine exhau	International Labour Organization (ILO). International Labour Organization Database (ILOSTAT) - Employment by Sex and Occupation. International Labour	Global	2000-2009	Estimate
Occupational exposure to diesel engine exhau	International Labour Organization (ILO). International Labour Organization Database (ILOSTAT) - Employment by Sex and Economic Activity. International	Global	1981-2014	Estimate
Occupational exposure to secondhand smoke	International Labour Organization (ILO). International Labour Organization Database (ILOSTAT) - Employment by Sex and Occupation. International Labour	Global	2000-2009	Estimate
Occupational exposure to secondhand smoke	International Labour Organization (ILO). International Labour Organization Database (ILOSTAT) - Employment by Sex and Economic Activity. International	Global	1981-2014	Estimate
Occupational exposure to formaldehyde	International Labour Organization (ILO). International Labour Organization Database (ILOSTAT) - Employment by Sex and Economic Activity. International	Global	1981-2014	Estimate
Occupational exposure to formaldehyde	International Labour Organization (ILO). International Labour Organization Database (ILOSTAT) - Employment by Sex and Occupation. International Labour	Global	2000-2009	Estimate
Occupational exposure to nickel	International Labour Organization (ILO). International Labour Organization Database (ILOSTAT) - Employment by Sex and Occupation. International Labour	Global	2000-2009	Estimate
Occupational exposure to nickel	International Labour Organization (ILO). International Labour Organization Database (ILOSTAT) - Employment by Sex and Economic Activity. International	Global	1981-2014	Estimate
Occupational exposure to polycyclic aromatic	International Labour Organization (ILO). International Labour Organization Database (ILOSTAT) - Employment by Sex and Occupation. International Labour	Global	2000-2009	Estimate
Occupational exposure to polycyclic aromatic	International Labour Organization (ILO). International Labour Organization Database (ILOSTAT) - Employment by Sex and Economic Activity. International	Global	1981-2014	Estimate
Occupational exposure to silica	International Labour Organization (ILO). International Labour Organization Database (ILOSTAT) - Employment by Sex and Occupation. International Labour	Global	2000-2009	Estimate
Occupational exposure to silica	International Labour Organization (ILO). International Labour Organization Database (ILOSTAT) - Employment by Sex and Economic Activity. International	Global	1981-2014	Estimate
Occupational exposure to sulfuric acid	International Labour Organization (ILO). International Labour Organization Database (ILOSTAT) - Employment by Sex and Economic Activity. International	Global	1981-2014	Estimate
Occupational exposure to trichloroethylene	International Labour Organization (ILO). International Labour Organization Database (ILOSTAT) - Employment by Sex and Economic Activity. International	Global	1981-2014	Estimate
Occupational asthmagens	International Labour Organization (ILO). International Labour Organization Database (ILOSTAT) - Employment by Sex and Economic Activity. International	Global	1981-2014	Estimate
Occupational asthmagens	International Labour Organization (ILO). International Labour Organization Database (ILOSTAT) - Employment by Sex and Occupation. International Labour	Global	2000-2009	Estimate
Occupational particulate matter, gases, and fu	International Labour Organization (ILO). International Labour Organization Database (ILOSTAT) - Employment by Sex and Economic Activity. International	Global	1981-2014	Estimate
Occupational particulate matter, gases, and fu	International Labour Organization (ILO). International Labour Organization Database (ILOSTAT) - Employment by Sex and Occupation. International Labour	Global	2000-2009	Estimate
Occupational noise	International Labour Organization (ILO). International Labour Organization Database (ILOSTAT) - Employment by Sex and Occupation. International Labour	Global	2000-2009	Estimate
Occupational noise	International Labour Organization (ILO). International Labour Organization Database (ILOSTAT) - Employment by Sex and Economic Activity. International	Global	1981-2014	Estimate
Occupational injuries	International Labour Organization (ILO). International Labour Organization Database (ILOSTAT) - Employment by Sex and Economic Activity. International	Global	1981-2014	Estimate
Occupational injuries	International Labour Organization (ILO). International Labour Organization Database (ILOSTAT) - Fatal Injuries by Sex and Economic Activity. International	Global	2000-2011	Estimate
Occupational ergonomic factors	International Labour Organization (ILO). International Labour Organization Database (ILOSTAT) - Employment by Sex and Economic Activity. International	Global	1981-2014	Estimate
Occupational ergonomic factors	International Labour Organization (ILO). International Labour Organization Database (ILOSTAT) - Employment by Sex and Occupation. International Labour	Global	2000-2009	Estimate
Suboptimal breastfeeding	Brazilian Society for Family Welfare (BEMFAM), Westinghouse; Institute for Resource Development. Brazil Demographic and Health Survey 1986. Columbia, United States: Westinghouse; Institute for Resource Development.	Country	1986	Survey
Suboptimal breastfeeding	Brazilian Society for Family Welfare (BEMFAM), Macro International, Inc. Brazil Demographic and Health Survey 1996. Calverton, United States: Macro	Country	1996	Survey
Suboptimal breastfeeding	Brazilian Society for Family Welfare (BEMFAM), Macro International, Inc. Brazil Demographic and Health Survey 1991. Calverton, United States: Macro	Country	1991	Survey
Non-exclusive breastfeeding	Brazilian Society for Family Welfare (BEMFAM), Westinghouse; Institute for Resource Development. Brazil Demographic and Health Survey 1986. Columbia, United States: Westinghouse; Institute for Resource Development.	Country	1986	Survey
Non-exclusive breastfeeding	Brazilian Society for Family Welfare (BEMFAM), Macro International, Inc. Brazil Demographic and Health Survey 1996. Calverton, United States: Macro	Country	1996	Survey
Non-exclusive breastfeeding	Brazilian Society for Family Welfare (BEMFAM), Macro International, Inc. Brazil Demographic and Health Survey 1991. Calverton, United States: Macro	Country	1991	Survey
Discontinued breastfeeding	Brazilian Society for Family Welfare (BEMFAM), Westinghouse; Institute for Resource Development. Brazil Demographic and Health Survey 1986. Columbia, United States: Westinghouse; Institute for Resource Development.	Country	1986	Survey
Discontinued breastfeeding	Brazilian Society for Family Welfare (BEMFAM), Macro International, Inc. Brazil Demographic and Health Survey 1996. Calverton, United States: Macro	Country	1996	Survey
Discontinued breastfeeding	Brazilian Society for Family Welfare (BEMFAM), Macro International, Inc. Brazil Demographic and Health Survey 1991. Calverton, United States: Macro	Country	1991	Survey

Child underweight	Opinion and Statistics (IBOPE), Ministry of Health (Brazil). Brazil National Demographic and Health Survey of Children and Women 2006-2007. Rio de Janeiro, Brazil: Ministry of Health (Brazil).	Country	2006-2007	Survey
Child underweight	months, Niterói, Brazil as it appears in World Health Organization (WHO). WHO Global Database on Child Growth and Malnutrition - Historical. Geneva, Switzerland: World Health Organization (WHO).		1995	Scientific literature
Child underweight	Brazil Consumer Expenditure Survey 2002-2003 as it appears in World Health Organization (WHO). WHO Global Database on Child Growth and Malnutrition. Geneva, Switzerland: World Health Organization (WHO).	Country	2002	Survey
Child underweight	National Health and Nutrition Survey as it appears in World Health Organization (WHO). WHO Global Database on Child Growth and Malnutrition. Geneva, Switzerland: World Health Organization (WHO).	Country	1989	Report
Child underweight	Guatemala Reproductive Health Survey 2002 as it appears in World Health Organization (WHO). WHO Global Database on Child Growth and Malnutrition. Geneva, Switzerland: World Health Organization (WHO).	Country	2002	Survey
Child underweight	appears in World Health Organization (WHO). WHO Global Database on Child Growth and Malnutrition - Historical. Geneva, Switzerland: World Health Organization (WHO).	Country	1987-1991	Report
Child underweight	Brazilian Society for Family Welfare (BEMFAM), Macro International, Inc. Brazil Demographic and Health Survey 1996. Calverton, United States: Macro	Country	1996	Survey
Child underweight	Mexico National Nutrition Survey in Rural Areas 1989 as it appears in World Health Organization (WHO). WHO Global Database on Child Growth and Malnutrition. Geneva, Switzerland: World Health Organization (WHO).	Country	1989	Survey
Child underweight	Brazilian Society for Family Welfare (BEMFAM), Westinghouse; Institute for Resource Development. Brazil Demographic and Health Survey 1986. Columbia, United States: Westinghouse; Institute for Resource Development.	Country	1986	Survey
Child underweight	Brazil Living Standards Measurement Survey 1996-1997	Ceará, Espírito Santo, Maranhão, Minas Gerais, Paraíba, Pernambuco, Piaui, Rio Grande do orte, Rio de Janeiro, Sergipe, São Paulo, Alagoas, Bahia, Ceará, Espírito Santo, Maranhão, Minas Gerais,	1996-1997	Survey
Child underweight	Guinea-Bissau SMART Nutrition Survey 2008 as it appears in World Health Organization (WHO). WHO Global Database on Child Growth and Malnutrition. Geneva, Switzerland: World Health Organization (WHO).	Country	2008	Survey
Child underweight	Mexico National Nutrition Survey 1998-1999 as it appears in World Health Organization (WHO). WHO Global Database on Child Growth and Malnutrition. Geneva, Switzerland: World Health Organization (WHO).	Country	1998	Survey
Child underweight	Brazil Demographic and Health Survey 1996 as it appears in World Health Organization (WHO). WHO Global Database on Child Growth and Malnutrition. Geneva, Switzerland: World Health Organization (WHO).	Country	1996	Survey
Child wasting	Mexico National Nutrition Survey in Rural Areas 1989 as it appears in World Health Organization (WHO). WHO Global Database on Child Growth and Malnutrition. Geneva, Switzerland: World Health Organization (WHO).	Country	1989	Survey
Child wasting	Brazilian Society for Family Welfare (BEMFAM), Macro International, Inc. Brazil Demographic and Health Survey 1996. Calverton, United States: Macro	Country	1996	Survey
Child wasting	Brazil Demographic and Health Survey 1996 as it appears in World Health Organization (WHO). WHO Global Database on Child Growth and Malnutrition. Geneva, Switzerland: World Health Organization (WHO).	Country	1996	Survey
Child wasting	children of the Aripuanã Park, Brazilian Amazon as it appears in World Health Organization (WHO). WHO Global Database on Child Growth and Malnutrition - Historical. Geneva, Switzerland: World Health Organization (WHO).		1989	Scientific literature
Child wasting	Health Organization (WHO). WHO Global Database on Child Growth and Malnutrition - Historical. Geneva, Switzerland: World Health Organization (WHO).	Country	1989	Survey

Child wasting	Brazil Living Standards Measurement Survey 1996-1997	Ceará, Espírito Santo, Maranhão, Minas Gerais, Paraíba, Pernambuco, Piaui, Rio Grande do orte, Rio de Janeiro, Sergipe, São Paulo, Alagoas, Bahia, Ceará, Espírito Santo, Maranhão, Minas Gerais,	1996-1997	Survey
Child wasting	Mexico National Nutrition Survey 1998-1999 as it appears in World Health Organization (WHO). WHO Global Database on Child Growth and Malnutrition. Geneva, Switzerland: World Health Organization (WHO).	Country	1998	Survey
Child wasting	Brazilian Society for Family Welfare (BEMFAM), Westinghouse; Institute for Resource Development. Brazil Demographic and Health Survey 1986. Columbia, United States: Westinghouse; Institute for Resource Development.	Country	1986	Survey
Child wasting	[Nutritional and feeding status of preschool children in the semi-arid region of Bahia (Brazil): I. Anthropometric assessment] as it appears in World Health Organization (WHO). WHO Global Database on Child Growth and Malnutrition - Historical. Geneva, Switzerland: World Health Organization (WHO).		1989	Scientific literature
Child wasting	Guatemala Reproductive Health Survey 2002 as it appears in World Health Organization (WHO). WHO Global Database on Child Growth and Malnutrition. Geneva, Switzerland: World Health Organization (WHO).	Country	2002	Survey
Child wasting	Guinea-Bissau SMART Nutrition Survey 2008 as it appears in World Health Organization (WHO). WHO Global Database on Child Growth and Malnutrition. Geneva, Switzerland: World Health Organization (WHO).	Country	2008	Survey
Child wasting	appears in World Health Organization (WHO). WHO Global Database on Child Growth and Malnutrition - Historical. Geneva, Switzerland: World Health Organization (WHO).	Country	1987-1991	Report
Child wasting	months, Niterói, Brazil as it appears in World Health Organization (WHO). WHO Global Database on Child Growth and Malnutrition - Historical. Geneva, Switzerland: World Health Organization (WHO).		1995	Scientific literature
Child wasting	Opinion and Statistics (IBOPE), Ministry of Health (Brazil). Brazil National Demographic and Health Survey of Children and Women 2006-2007. Rio de Janeiro, Brazil: Ministry of Health (Brazil).	Country	2006-2007	Survey
Child stunting	Opinion and Statistics (IBOPE), Ministry of Health (Brazil). Brazil National Demographic and Health Survey of Children and Women 2006-2007. Rio de Janeiro, Brazil: Ministry of Health (Brazil).	Country	2006-2007	Survey
Child stunting	Guatemala Reproductive Health Survey 2002 as it appears in World Health Organization (WHO). WHO Global Database on Child Growth and Malnutrition. Geneva, Switzerland: World Health Organization (WHO).	Country	2002	Survey
Child stunting	National Health and Nutrition Survey as it appears in World Health Organization (WHO). WHO Global Database on Child Growth and Malnutrition. Geneva, Switzerland: World Health Organization (WHO).	Country	1989	Report
Child stunting	Brazilian Society for Family Welfare (BEMFAM), Macro International, Inc. Brazil Demographic and Health Survey 1996. Calverton, United States: Macro	Country	1996	Survey
Child stunting	Mexico National Nutrition Survey in Rural Areas 1989 as it appears in World Health Organization (WHO). WHO Global Database on Child Growth and Malnutrition. Geneva, Switzerland: World Health Organization (WHO).	Country	1989	Survey
Child stunting	Brazil Demographic and Health Survey 1996 as it appears in World Health Organization (WHO). WHO Global Database on Child Growth and Malnutrition. Geneva, Switzerland: World Health Organization (WHO).	Country	1996	Survey
Child stunting	Brazilian Society for Family Welfare (BEMFAM), Westinghouse; Institute for Resource Development. Brazil Demographic and Health Survey 1986. Columbia, United States: Westinghouse; Institute for Resource Development.	Country	1986	Survey
Child stunting	children of the Aripuanã Park, Brazilian Amazon as it appears in World Health Organization (WHO). WHO Global Database on Child Growth and Malnutrition - Historical. Geneva, Switzerland: World Health Organization (WHO).		1989	Scientific literature
Child stunting	Guinea-Bissau SMART Nutrition Survey 2008 as it appears in World Health Organization (WHO). WHO Global Database on Child Growth and Malnutrition. Geneva, Switzerland: World Health Organization (WHO).	Country	2008	Survey
Child stunting	appears in World Health Organization (WHO). WHO Global Database on Child Growth and Malnutrition - Historical. Geneva, Switzerland: World Health Organization (WHO).	Country	1987-1992	Report

Child stunting	Brazil Living Standards Measurement Survey 1996-1997	Ceará, Espírito Santo, Maranhão, Minas Gerais, Paraíba, Pernambuco, Piaui, Rio Grande do orte, Rio de Janeiro, Sergipe, São Paulo, Alagoas, Bahia, Ceará, Espírito Santo, Maranhão, Minas Gerais,	1996-1997	Survey
Child stunting	months, Niterói, Brazil as it appears in World Health Organization (WHO). WHO Global Database on Child Growth and Malnutrition - Historical. Geneva, Switzerland: World Health Organization (WHO).		1995	Scientific literature
Child stunting	Mexico National Nutrition Survey 1998-1999 as it appears in World Health Organization (WHO). WHO Global Database on Child Growth and Malnutrition. Geneva, Switzerland: World Health Organization (WHO).	Country	1998	Survey
Iron deficiency	the National Institutes of Health (FNIH), National Institute of Science, Technology, and Biomedicine of Semi-Arid Brazil (INCT-IBISAB). Brazil - Fortaleza Malnutrition and Enteric Disease Study 2009-2014.	Ceará, Ceará, Ceará, Ceará, Ceará	2010-2013	Survey
Iron deficiency	Opinion and Statistics (IBOPE), Ministry of Health (Brazil). Brazil National Demographic and Health Survey of Children and Women 2006-2007. Rio de Janeiro, Brazil: Ministry of Health (Brazil).	Country	2006-2007	Survey
Iron deficiency	Rondo PH, Abbott R, Rodrigues LC, Tomkins AM. Vitamin A, folate, and iron concentrations in cord and maternal blood of intra-uterine growth retarded and appropriate birth weight babies. . 1995; 49(6): 391-9.		1991-1992	Scientific literature
Iron deficiency	Osório MM, Lira PI, Batista-Filho M, Ashworth A. Prevalence of anemia in children 6-59 months old in the state of Pernambuco, Brazil. . 2001; 10(2): 101-		2006	Scientific literature
Iron deficiency	Muniz-Junqueira MI, Queiroz EFO. Relationship between protein-energy malnutrition, vitamin A, and parasitoses in living in Brasília. . 2002; 35(2): 133-		1983	Scientific literature
Iron deficiency	Brazil - School Lunch: History, Evolution and Contribution in Addressing the Nutritional Needs of the Child		1996	Report
Iron deficiency	Iron Deficiency and Iron Deficiency Anemia in the Population of 6 Months to 6 Years in Vitória, Espírito Santo, Southeastern Brazil	Espírito Santo	2001-2003	Report
Iron deficiency	Piaui State Government, United Nations Children's Fund (UNICEF). Brazil - Piauí Children and Adolescents: Health, Education, and Work 1991. Teresina, Brazil:	Piaui	1991	Report
Iron deficiency	Assis AMO, Gaudenzi EN, Gomes G, Ribeiro R de C, Szarfarc SC, Souza SB de. [Hemoglobin concentration, breastfeeding and complementary feeding in the first year of life]. . 2004; 38(4): 543-51.		1998-1999	Scientific literature
Vitamin A deficiency	World Bank. World Development Indicators - Vitamin A Supplementation Coverage Rate. Washington DC, United States: World Bank.	Global	1999	Administrative record
Vitamin A deficiency	Barreto ML, Santos LM, Assis AM, Araújo MP, Farenzena GG, Santos PA, Fiaccone RL. Effect of vitamin A supplementation on diarrhoea and acute lower-respiratory-tract infections in young children in Brazil. . 2010; CD008524.		1990-1991	Scientific literature
Vitamin A deficiency	Martins MC, Santos LMP, Assis AMO. [Prevalence of hypovitaminosis A among preschool children from northeastern Brazil, 1998]. . 2004; 38(4): 537-42.		1998	Scientific literature
Vitamin A deficiency	Azevedo MMS de, Cabral PC, Diniz A da S, Fisberg M, Fisberg RM, Arruda IKG de. [Vitamin A deficiency in preschool children of Recife, Northeast of Brazil]. .		2007	Scientific literature
Vitamin A deficiency	Brazil - Pernambuco Second State Survey of Health and Nutrition 1997 as it	Pernambuco	1997	Survey
Zinc deficiency	Kanerva N, Kaartinen NE, Ovaskainen M-L, Konttinen H, Kontto J, Männistö S. A diet following Finnish nutrition recommendations does not contribute to the current epidemic of obesity. . 2013; 16(5): 78694.		1996-1997	Scientific literature
Zinc deficiency	Food and Agriculture Organization of the United Nations (FAO). FAOSTAT Food Balance Sheets, October 2014. Rome, Italy: Food and Agriculture Organization	Global	1990-2015	Administrative record
Zinc deficiency	Verly Junior E, Fisberg RM, Cesar CLG, Marchioni DML. Sources of variation of energy and nutrient intake among adolescents in São Paulo, Brazil. . 2010;		2007-2008	Scientific literature
Zinc deficiency	Henn RL, Fuchs SC, Moreira LB, Fuchs FD. Development and validation of a food frequency questionnaire (FFQ-Porto Alegre) for adolescent, adult and elderly populations from Southern Brazil. . 2010; 26(11): 206879.		2007-2009	Scientific literature
Smoking	Brazilian Institute of Geography and Statistics (IBGE), Ministry of Education (Brazil), Ministry of Health (Brazil), Ministry of Planning, Budget, and Management (Brazil). Brazil National Survey of School Health 2009.	Country	2009	Survey
Smoking	Ministry of Health (Brazil), Secretariat of Health Surveillance, Ministry of Health (Brazil), University of São Paulo. Brazil Surveillance System of Risk Factors for Chronic Diseases by Telephone Interviews 2016.	Country	2016	Survey
Smoking	Ministry of Health (Brazil), Secretariat of Health Surveillance, Ministry of Health (Brazil), University of São Paulo. Brazil Surveillance System of Risk Factors for Chronic Diseases by Telephone Interviews 2016.	Country	2016	Survey
Smoking	Ministry of Health (Brazil), Secretariat of Health Surveillance, Ministry of Health (Brazil). Brazil Surveillance System of Risk Factors for Chronic Diseases by	Country	2008	Survey

Smoking	Ministry of Health (Brazil), Secretariat of Health Surveillance, Ministry of Health (Brazil). Brazil Surveillance System of Risk Factors for Chronic Diseases by	Country	2009	Survey
Smoking	Brazilian Institute of Geography and Statistics (IBGE), Ministry of Education (Brazil), Ministry of Health (Brazil), Ministry of Planning, Budget, and Management (Brazil). Brazil National Survey of School Health 2009.	Country	2009	Survey
Smoking	Ministry of Health (Brazil), Secretariat of Health Surveillance, Ministry of Health (Brazil), University of São Paulo. Brazil Surveillance System of Risk Factors for Chronic Diseases by Telephone Interviews 2015.	Country	2015	Survey
Smoking	Ministry of Health (Brazil), Secretariat of Health Surveillance, Ministry of Health (Brazil), University of São Paulo. Brazil Surveillance System of Risk Factors for Chronic Diseases by Telephone Interviews 2015.	Country	2015	Survey
Smoking	Ministry of Health (Brazil), Secretariat of Health Surveillance, Ministry of Health (Brazil). Brazil Surveillance System of Risk Factors for Chronic Diseases by	Country	2009	Survey
Smoking	Ministry of Health (Brazil), Secretariat of Health Surveillance, Ministry of Health (Brazil). Brazil Surveillance System of Risk Factors for Chronic Diseases by	Country	2010	Survey
Smoking	Brazilian Institute of Geography and Statistics (IBGE). Brazil National Survey of School Health 2015. Rio de Janeiro, Brazil: Brazilian Institute of Geography and	Country	2015	Survey
Smoking	Ministry of Health (Brazil), Secretariat of Health Surveillance, Ministry of Health (Brazil). Brazil Surveillance System of Risk Factors for Chronic Diseases by	Country	2010	Survey
Smoking	Ministry of Health (Brazil), Secretariat of Health Surveillance, Ministry of Health (Brazil). Brazil Surveillance System of Risk Factors for Chronic Diseases by	Country	2011	Survey
Smoking	Ministry of Health (Brazil), Secretariat of Health Surveillance, Ministry of Health (Brazil). Brazil Surveillance System of Risk Factors for Chronic Diseases by	Country	2008	Survey
Smoking	Brazil - Hearts of Brazil Survey 2004	Country	2004	Survey
Smoking	Brazilian Institute of Geography and Statistics (IBGE). Brazil National Survey of School Health 2015. Rio de Janeiro, Brazil: Brazilian Institute of Geography and	Country	2015	Survey
Smoking	Ministry of Health (Brazil), Secretariat of Health Surveillance, Ministry of Health (Brazil). Brazil Surveillance System of Risk Factors for Chronic Diseases by	Country	2011	Survey
Smoking	Brazil - Hearts of Brazil Survey 2004	Country	2004	Survey
Smoking	Ministry of Health (Brazil), Secretariat of Health Surveillance, Ministry of Health (Brazil). Brazil Surveillance System of Risk Factors for Chronic Diseases by	Country	2012	Survey
Smoking	(Brazil), University of São Paulo. Brazil Surveillance System of Risk Factors for Chronic Diseases by Telephone Interviews 2014. Rio de Janeiro, Brazil: Secretariat of Health Surveillance, Ministry of Health (Brazil).	Country	2014	Survey
Smoking	Brazil Household Survey About the Use of Psychotropic Drugs 2005	Country	2005	Survey
Smoking	Iser BPM, Yokota RTC, de Sa NNB, de Moura L, Malta DC. Prevalencia de fatores de risco e protecao para doencas cronicas nas capitais do Brasil - principais resultados do Vigitel 2010. . 2012; 17(9): 2343-56.		2010	Scientific literature
Smoking	Ministry of Health (Brazil), Secretariat of Health Surveillance, Ministry of Health (Brazil). Brazil Surveillance System of Risk Factors for Chronic Diseases by	Country	2012	Survey
Smoking	Ministry of Health (Brazil). Brazil Survey of Knowledge, Attitudes, and Practices	Country	2008	Survey
Smoking	Brazil Household Survey About the Use of Psychotropic Drugs 2005	Country	2005	Survey
Smoking	Ministry of Planning, Budget, and Management (Brazil). Brazil National Health Survey 2013. Rio de Janeiro, Brazil: Brazilian Institute of Geography and Statistics (IBGE).	Country	2013	Survey
Smoking	Ministry of Health (Brazil). Brazil Survey of Knowledge, Attitudes, and Practices	Country	2008	Survey
Smoking	(Brazil), University of São Paulo. Brazil Surveillance System of Risk Factors for Chronic Diseases by Telephone Interviews 2013. Rio de Janeiro, Brazil: Secretariat of Health Surveillance, Ministry of Health (Brazil).	Country	2013	Survey
Smoking	(Brazil), University of São Paulo. Brazil Surveillance System of Risk Factors for Chronic Diseases by Telephone Interviews 2013. Rio de Janeiro, Brazil: Secretariat of Health Surveillance, Ministry of Health (Brazil).	Country	2013	Survey
Smoking	Ministry of Planning, Budget, and Management (Brazil). Brazil National Health Survey 2013. Rio de Janeiro, Brazil: Brazilian Institute of Geography and Statistics (IBGE).	Country	2013	Survey
Smoking	(Brazil), University of São Paulo. Brazil Surveillance System of Risk Factors for Chronic Diseases by Telephone Interviews 2014. Rio de Janeiro, Brazil: Secretariat of Health Surveillance, Ministry of Health (Brazil).	Country	2014	Survey
Smoking	Iser BPM, Yokota RTC, de Sa NNB, de Moura L, Malta DC. Prevalencia de fatores de risco e protecao para doencas cronicas nas capitais do Brasil - principais resultados do Vigitel 2010. . 2012; 17(9): 2343-56.		2010	Scientific literature
Smoking	Brazil - Rio Grande do Sul Global Youth Tobacco Survey 2002	Rio Grande do	2002	Survey
Smoking	Iser BPM, Malta DC Claro RM, de Moura EC, Neto OlM. Fatores de risco e protecao para doencas cronicas nao transmissiveis obtidos por inquerito telefonico - VIGITEL Brasil - 2009. . 2011; 14(Suppl 1): 90-102.		2009	Scientific literature
Smoking	(Brazil), Ministry of Health (Brazil), Ministry of Planning, Budget, and Management (Brazil), Secretariat of Health Surveillance, Ministry of Health (Brazil). Brazil National Survey of School Health 2012.	Country	2012	Survey
Smoking	Brazil World Health Survey 2003	Country	2003	Survey
Smoking	Brazil World Health Survey 2003	Country	2003	Survey
Smoking	Brazilian Society for Family Welfare (BEMFAM), Macro International, Inc. Brazil Demographic and Health Survey 1996. Calverton, United States: Macro	Country	1996	Survey
Smoking	Brazilian Society for Family Welfare (BEMFAM), Macro International, Inc. Brazil Demographic and Health Survey 1996. Calverton, United States: Macro	Country	1996	Survey

Smoking	and Statistics (IBGE), Centers for Disease Control and Prevention (CDC), Ministry of Health (Brazil), National Cancer Institute (Brazil), Pan American Health Organization (PAHO), Secretariat of Health Surveillance, Ministry of Health (Brazil). Brazil Global Adult Tobacco Survey 2008. Atlanta, United States: Centers for Disease Control and Prevention (CDC).	Country	2008	Survey
Smoking	and Statistics (IBGE), Centers for Disease Control and Prevention (CDC), Ministry of Health (Brazil), National Cancer Institute (Brazil), Pan American Health Organization (PAHO), Secretariat of Health Surveillance, Ministry of Health (Brazil). Brazil Global Adult Tobacco Survey 2008. Atlanta, United States: Centers for Disease Control and Prevention (CDC).	Country	2008	Survey
Smoking	Brazilian Society for Family Welfare (BEMFAM), Macro International, Inc. Brazil Demographic and Health Survey 1991. Calverton, United States: Macro	Country	1991	Survey
Smoking	Brazil - Rio de Janeiro Global Youth Tobacco Survey 2005	Rio de Janeiro	2005	Survey
Smoking	Brazil - Rio de Janeiro Global Youth Tobacco Survey 2005	Rio de Janeiro	2005	Survey
Smoking	Brazil - Rio Grande do Norte Global Youth Tobacco Survey 2002	Rio Grande do	2002	Survey
Smoking	Brazil - Rio Grande do Norte Global Youth Tobacco Survey 2002	Rio Grande do	2002	Survey
Smoking	Brazil - Rio Grande do Sul Global Youth Tobacco Survey 2002	Rio Grande do	2002	Survey
Smoking	Brazil Risk Factor Morbidity Noncommunicable Disease Survey 2002-2005	Amazonas, Ceará, Distrito Federal, Espírito Santo, Mato Grosso Do Sul, Minas Gerais, Paraná, Paraíba, Pará, Pernambuco, Rio Grande do orte, Rio Grande do Sul, Rio de Janeiro, Santa Catarina, Sergipe, São Paulo, Amazonas, Ceará, Distrito Federal, Espírito Santo, Mato Grosso Do Sul, Minas Gerais, Paraná, Paraíba, Pará, Pernambuco, Rio Grande do orte, Rio Grande do Sul, Rio de Janeiro, Santa	2002	Survey
Smoking	Brazilian Society for Family Welfare (BEMFAM), Macro International, Inc. Brazil Demographic and Health Survey 1991. Calverton, United States: Macro	Country	1991	Survey

Smoking	Brazil Risk Factor Morbidity Noncommunicable Disease Survey 2002-2005	Amazonas, Ceará, Distrito Federal, Espírito Santo, Mato Grosso Do Sul, Minas Gerais, Paraná, Paraíba, Pará, Pernambuco, Rio Grande do orte, Rio Grande do Sul, Rio de Janeiro, Santa Catarina, Sergipe, São Paulo, Amazonas, Ceará, Distrito Federal, Espírito Santo, Mato Grosso Do Sul, Minas Gerais, Paraná, Paraíba, Pará, Pernambuco, Rio Grande do orte, Rio Grande do Sul, Rio de Janeiro, Santa	2002	Survey
Smoking	Brazil National Survey on Health and Nutrition 1989	Country	1989	Survey
Smoking	Ministry of Health (Brazil), Secretariat of Health Surveillance, Ministry of Health (Brazil), University of São Paulo. Brazil Surveillance System of Risk Factors for Chronic Diseases by Telephone Interviews 2006.	Country	2006	Survey
Smoking	Brazilian Society for Family Welfare (BEMFAM), Westinghouse; Institute for Resource Development. Brazil Demographic and Health Survey 1986. Columbia, United States: Westinghouse; Institute for Resource Development.	Country	1986	Survey
Smoking	Ministry of Health (Brazil), Secretariat of Health Surveillance, Ministry of Health (Brazil), University of São Paulo. Brazil Surveillance System of Risk Factors for Chronic Diseases by Telephone Interviews 2006.	Country	2006	Survey
Smoking	Ministry of Health (Brazil), Secretariat of Health Surveillance, Ministry of Health (Brazil). Brazil Surveillance System of Risk Factors for Chronic Diseases by	Country	2007	Survey
Smoking	Brazilian Society for Family Welfare (BEMFAM), Westinghouse; Institute for Resource Development. Brazil Demographic and Health Survey 1986. Columbia, United States: Westinghouse; Institute for Resource Development.	Country	1986	Survey
Smoking	Ministry of Health (Brazil), Secretariat of Health Surveillance, Ministry of Health (Brazil). Brazil Surveillance System of Risk Factors for Chronic Diseases by	Country	2007	Survey
Smoking	Iser BPM, Malta DC Claro RM, de Moura EC, Neto OlM. Fatores de risco e protecao para doencas cronicas nao transmissiveis obtidos por inquerito telefonico - VIGITEL Brasil - 2009. . 2011; 14(Suppl 1): 90-102.		2009	Scientific literature
Smoking	Centers for Disease Control and Prevention (CDC), World Health Organization (WHO). Brazil - Alagoas Global Youth Tobacco Survey 2004 . Atlanta, United States: Centers for Disease Control and Prevention (CDC).	Alagoas, Alagoas, Alagoas	2004	Survey
Smoking	Centers for Disease Control and Prevention (CDC), World Health Organization (WHO). Brazil - Mato Grosso Do Sul Global Youth Tobacco Survey 2002. Atlanta, United States: Centers for Disease Control and Prevention (CDC).	Sul, Mato Grosso Do Sul, Mato Grosso Do Sul	2002	Survey
Smoking	Centers for Disease Control and Prevention (CDC), World Health Organization (WHO). Brazil - Mato Grosso Do Sul Global Youth Tobacco Survey 2002. Atlanta, United States: Centers for Disease Control and Prevention (CDC).	Sul, Mato Grosso Do Sul, Mato Grosso Do Sul	2002	Survey
Smoking	Centers for Disease Control and Prevention (CDC), World Health Organization (WHO). Brazil - Paraíba Global Youth Tobacco Survey 2002 . Atlanta, United States: Centers for Disease Control and Prevention (CDC).	Paraíba, Paraíba, Paraíba	2002	Survey
Smoking	Centers for Disease Control and Prevention (CDC), World Health Organization (WHO). Brazil - Paraíba Global Youth Tobacco Survey 2002 . Atlanta, United States: Centers for Disease Control and Prevention (CDC).	Paraíba, Paraíba, Paraíba	2002	Survey
Smoking	Brazil National Survey on Health and Nutrition 1989	Country	1989	Survey
Smoking	Centers for Disease Control and Prevention (CDC), World Health Organization (WHO). Brazil - Alagoas Global Youth Tobacco Survey 2004 . Atlanta, United States: Centers for Disease Control and Prevention (CDC).	Alagoas, Alagoas, Alagoas	2004	Survey
Smoking	(Brazil), Ministry of Health (Brazil), Ministry of Planning, Budget, and Management (Brazil), Secretariat of Health Surveillance, Ministry of Health (Brazil). Brazil National Survey of School Health 2012.	Country	2012	Survey
Smokeless tobacco	Brazilian Institute of Geography and Statistics (IBGE). Brazil National Survey of School Health 2015. Rio de Janeiro, Brazil: Brazilian Institute of Geography and	Country	2015	Survey

Smokeless tobacco	and Statistics (IBGE), Centers for Disease Control and Prevention (CDC), Ministry of Health (Brazil), National Cancer Institute (Brazil), Pan American Health Organization (PAHO), Secretariat of Health Surveillance, Ministry of Health (Brazil). Brazil Global Adult Tobacco Survey 2008. Atlanta, United States: Centers for Disease Control and Prevention (CDC).	Country	2008	Survey
Secondhand smoke	Brazilian Society for Family Welfare (BEMFAM), Macro International, Inc. Brazil Demographic and Health Survey 1991. Calverton, United States: Macro	Country	1991	Survey
Secondhand smoke	Brazilian Society for Family Welfare (BEMFAM), Macro International, Inc. Brazil Demographic and Health Survey 1996. Calverton, United States: Macro	Country	1996	Survey
Secondhand smoke	Brazil General Census 1991 - IPUMS	Country	1991	Census
Secondhand smoke	Brazil General Census 1980 - IPUMS	Country	1980	Census
Secondhand smoke	Brazil General Census 2000 - IPUMS	Country	2000	Census
Secondhand smoke	Brazil Demographic Census 2010 - IPUMS	Country	2010	Census
Alcohol use	Brazil World Health Survey 2003	Country	2003	Survey
Alcohol use	Group, Public Health Institute, Centre for Addiction and Mental Health (Canada), Centre for Alcohol Policy Research, Turning Point Alcohol and Drug Centre (Australia), Kettil Bruun Society for Social and Epidemiological Research on Alcohol, University of North Dakota. Brazil - Botucatu Gender, Alcohol and Culture: An International Study (GENACIS) 2001-2002.	São Paulo, São Paulo, São Paulo	2001	Survey
Drug use	Ministry of Health (Brazil). Brazil Survey of Knowledge, Attitudes, and Practices	Country	2005	Survey
Drug use	Ministry of Health (Brazil). Brazil Survey of Knowledge, Attitudes, and Practices	Country	2005	Survey
Drug use	Bastos FI, Bertoni N, Hacker MA, Study Group on Population, Gender and AIDS (Brazil). [Drug and alcohol use: main findings of a national survey, Brazil 2005]. .		2005	Scientific literature
Drug use	Bastos FI, Bertoni N, Hacker MA, Study Group on Population, Gender and AIDS (Brazil). [Drug and alcohol use: main findings of a national survey, Brazil 2005]. .		2005	Scientific literature
Diet low in fruits	Food and Agriculture Organization of the United Nations (FAO). FAOSTAT Food Balance Sheets, October 2014. Rome, Italy: Food and Agriculture Organization	Global	1990-2015	Administrative record
Diet low in fruits	Euromonitor International. Euromonitor Passport - Fresh Foods Market Statistics. London, United Kingdom: Euromonitor International.	Country	2001-2015	Administrative record
Diet low in fruits	Barbosa F dos S, Sichieri R, Junger WL. Assessing usual dietary intake in complex sample design surveys: the National Dietary Survey. . 2013; 47 Suppl 1: 171S?6S.		2008	Scientific literature
Diet low in fruits	Brazil Consumer Expenditure Survey 2008-2009	Country	2008-2009	Survey
Diet low in vegetables	Brazil Consumer Expenditure Survey 2008-2009	Country	2008-2009	Survey
Diet low in vegetables	Food and Agriculture Organization of the United Nations (FAO). FAOSTAT Food Balance Sheets, October 2014. Rome, Italy: Food and Agriculture Organization	Global	1990-2015	Administrative record
Diet low in vegetables	Euromonitor International. Euromonitor Passport - Vegetables Market Statistics. London, United Kingdom: Euromonitor International.	Global	1990-2015	Administrative record
Diet low in legumes	Food and Agriculture Organization of the United Nations (FAO). FAOSTAT Food Balance Sheets, October 2014. Rome, Italy: Food and Agriculture Organization	Global	1990-2015	Administrative record
Diet low in legumes	Brazil Consumer Expenditure Survey 2008-2009	Country	2008-2009	Survey
Diet low in legumes	Euromonitor International. Euromonitor Passport - Pulses Market Statistics. London, United Kingdom: Euromonitor International.	Country	1990-2015	Administrative record
Diet low in whole grains	Brazil Consumer Expenditure Survey 2008-2009 as it appears in	Country	2008-2009	Survey
Diet low in nuts and seeds	Brazil Consumer Expenditure Survey 2008-2009	Country	2008-2009	Survey
Diet low in nuts and seeds	Food and Agriculture Organization of the United Nations (FAO). FAOSTAT Food Balance Sheets, October 2014. Rome, Italy: Food and Agriculture Organization	Global	1990-2015	Administrative record
Diet low in nuts and seeds	Euromonitor International. Euromonitor Passport - Nuts Market Statistics. London, United Kingdom: Euromonitor International.	Global	1990-2015	Administrative record
Diet low in milk	Brazil Consumer Expenditure Survey 2008-2009	Country	2008-2009	Survey
Diet low in milk	Food and Agriculture Organization of the United Nations (FAO). FAOSTAT Food Balance Sheets, October 2014. Rome, Italy: Food and Agriculture Organization	Global	1990-2015	Administrative record
Diet low in milk	Euromonitor International. Euromonitor Passport - Dairy Market Statistics. London, United Kingdom: Euromonitor International.	Global	2002-2015	Administrative record
Diet low in milk	Peters BSE, Verly E, Marchioni DML, Fisberg M, Martini LA. The influence of breakfast and dairy products on dietary calcium and vitamin D intake in postpubertal adolescents and young adults. . 2012; 25(1): 69?74.		2006	Scientific literature
Diet high in red meat	Food and Agriculture Organization of the United Nations (FAO). FAOSTAT Food Balance Sheets, October 2014. Rome, Italy: Food and Agriculture Organization	Global	1990-2015	Administrative record
Diet high in red meat	Euromonitor International. Euromonitor Passport - Meat Market Statistics. London, United Kingdom: Euromonitor International.	Global	2002-2015	Administrative record
Diet high in red meat	de Carvalho AM, Cesar CLG, Fisberg RM, Marchioni DM. Meat consumption in São Paulo-Brazil: trend in the last decade. . 2014; 9(5): e96667.		2003-2008	Scientific literature
Diet high in red meat	Brazil Consumer Expenditure Survey 2008-2009	Country	2008-2009	Survey
Diet high in processed meat	Euromonitor International. Partially Hydrogenated Vegetable Oil Sales	Country	1990-2015	Administrative record
Diet high in processed meat	Brazil Consumer Expenditure Survey 2008-2009	Country	2008-2009	Survey
Diet high in processed meat	Euromonitor International. Euromonitor Passport - Processed Meat and Seafood Market Statistics. London, United Kingdom: Euromonitor International.	Global	2002-2015	Administrative record
Diet high in sugar-sweetened beverages	Euromonitor International. Euromonitor Passport - Fruit Market Statistics. London, United Kingdom: Euromonitor International.	Global	2002-2015	Administrative record
Diet high in sugar-sweetened beverages	Brazil Consumer Expenditure Survey 2008-2009 as it appears in	Country	2008-2009	Survey
Diet high in sugar-sweetened beverages	Brazil Consumer Expenditure Survey 2008-2009	Country	2008-2009	Survey
Diet high in sugar-sweetened beverages	Pereira RA, Souza AM, Duffey KJ, Sichieri R, Popkin BM. Beverage consumption in Brazil: results from the first National Dietary Survey. . 2015; 18(7): 1164?72.		2008-2009	Scientific literature

Diet low in fiber	Molina MC, Bettiol H, Barbieri MA, Silva AA, Conceicao SI, Dos-Santos JE. Food consumption by young adults living in Ribeirao Preto, SP, 2002/2004. . 2007;		2002-2004	Scientific literature
Diet low in fiber	Verly Junior E, Fisberg RM, Cesar CLG, Marchioni DML. Sources of variation of energy and nutrient intake among adolescents in São Paulo, Brazil. . 2010;		2007-2008	Scientific literature
Diet low in fiber	Martinez MF, Philippi ST, Estima C, Leal G. Validity and reproducibility of a food frequency questionnaire to assess food group intake in adolescents. . 2013;		2010	Scientific literature
Diet low in fiber	Henn RL, Fuchs SC, Moreira LB, Fuchs FD. Development and validation of a food frequency questionnaire (FFQ-Porto Alegre) for adolescent, adult and elderly populations from Southern Brazil. . 2010; 26(11): 2068‐79.		2007-2009	Scientific literature
Diet low in fiber	Scagliusi FB, Moriguti EKU, Monteiro JP, Ferriolli E. Calibration of the food list and portion sizes of a food frequency questionnaire applied to free-living elderly people. . 2013; 29(5): 760‐4.		2010-2012	Scientific literature
Diet low in fiber	Brazil Consumer Expenditure Survey 2008-2009	Country	2008-2009	Survey
Diet low in fiber	Food and Agriculture Organization of the United Nations (FAO). FAOSTAT Food Balance Sheets, October 2014. Rome, Italy: Food and Agriculture Organization	Global	1990-2015	Administrative record
Diet low in fiber	Kanerva N, Kaartinen NE, Ovaskainen M-L, Konttinen H, Kontto J, Männistö S. A diet following Finnish nutrition recommendations does not contribute to the current epidemic of obesity. . 2013; 16(5): 786‐94.		1996-1997	Scientific literature
Diet low in calcium	Scagliusi FB, Moriguti EKU, Monteiro JP, Ferriolli E. Calibration of the food list and portion sizes of a food frequency questionnaire applied to free-living elderly people. . 2013; 29(5): 760‐4.		2010-2012	Scientific literature
Diet low in calcium	Food and Agriculture Organization of the United Nations (FAO). FAOSTAT Food Balance Sheets, October 2014. Rome, Italy: Food and Agriculture Organization	Global	1990-2015	Administrative record
Diet low in calcium	Kanerva N, Kaartinen NE, Ovaskainen M-L, Konttinen H, Kontto J, Männistö S. A diet following Finnish nutrition recommendations does not contribute to the current epidemic of obesity. . 2013; 16(5): 786‐94.		1996-1997	Scientific literature
Diet low in calcium	Martini LA, Verly E, Marchioni DML, Fisberg RM. Prevalence and correlates of calcium and vitamin D status adequacy in adolescents, adults, and elderly from the Health Survey-São Paulo. . 2013; 29(6): 845‐50.		2008	Scientific literature
Diet low in calcium	Verly Junior E, Fisberg RM, Cesar CLG, Marchioni DML. Sources of variation of energy and nutrient intake among adolescents in São Paulo, Brazil. . 2010;		2007-2008	Scientific literature
Diet low in calcium	Henn RL, Fuchs SC, Moreira LB, Fuchs FD. Development and validation of a food frequency questionnaire (FFQ-Porto Alegre) for adolescent, adult and elderly populations from Southern Brazil. . 2010; 26(11): 2068‐79.		2007-2009	Scientific literature
Diet low in calcium	Peters BSE, Verly E, Marchioni DML, Fisberg M, Martini LA. The influence of breakfast and dairy products on dietary calcium and vitamin D intake in postpubertal adolescents and young adults. . 2012; 25(1): 69‐74.		2006	Scientific literature
Diet low in calcium	Food and Agriculture Organization of the United Nations (FAO). FAOSTAT Food Balance Sheets, April 2015. Rome, Italy: Food and Agriculture Organization of	Global	1990-2015	Administrative record
Diet low in seafood omega-3 fatty acids	Food and Agriculture Organization of the United Nations (FAO). FAOSTAT Food Balance Sheets, October 2014. Rome, Italy: Food and Agriculture Organization	Global	1990-2015	Administrative record
Diet low in polyunsaturated fatty acids	Food and Agriculture Organization of the United Nations (FAO). FAOSTAT Food Balance Sheets, October 2014. Rome, Italy: Food and Agriculture Organization	Global	1990-2015	Administrative record
Diet low in polyunsaturated fatty acids	Henn RL, Fuchs SC, Moreira LB, Fuchs FD. Development and validation of a food frequency questionnaire (FFQ-Porto Alegre) for adolescent, adult and elderly populations from Southern Brazil. . 2010; 26(11): 2068‐79.		2007-2009	Scientific literature
Diet high in trans fatty acids	Euromonitor International. Partially Hydrogenated Vegetable Oil Sales	Country	2002-2015	Administrative record
Diet high in sodium	Verly Junior E, Fisberg RM, Cesar CLG, Marchioni DML. Sources of variation of energy and nutrient intake among adolescents in São Paulo, Brazil. . 2010;		2007-2008	Scientific literature
Childhood sexual abuse	Ipsos, National Institute of Public Policy for Alcohol and Other Drugs (INPAD) (Brazil), University of São Paulo. Brazil National Alcohol and Drugs Survey 2011-	Country	2011-2012	Survey
Childhood sexual abuse	Bassani DG, Palazzo LS, Béria JU, Gigante LP, Figueiredo AC, Aerts DR, Raymann BC. Child sexual abuse in southern Brazil and associated factors: a population-		2002-2003	Scientific literature
Childhood sexual abuse	Group, Public Health Institute, Centre for Addiction and Mental Health (Canada), Centre for Alcohol Policy Research, Turning Point Alcohol and Drug Centre (Australia), Kettil Bruun Society for Social and Epidemiological Research on Alcohol, University of North Dakota. Brazil - Botucatu Gender, Alcohol and Culture: An International Study (GENACIS) 2001-2002.	São Paulo, São Paulo, São Paulo	2001	Survey
Childhood sexual abuse	Bassani DG, Palazzo LS, Béria JU, Gigante LP, Figueiredo AC, Aerts DR, Raymann BC. Child sexual abuse in southern Brazil and associated factors: a population-		2002-2003	Scientific literature
Childhood sexual abuse	Group, Public Health Institute, Centre for Addiction and Mental Health (Canada), Centre for Alcohol Policy Research, Turning Point Alcohol and Drug Centre (Australia), Kettil Bruun Society for Social and Epidemiological Research on Alcohol, University of North Dakota. Brazil - Botucatu Gender, Alcohol and Culture: An International Study (GENACIS) 2001-2002.	São Paulo, São Paulo, São Paulo	2001	Survey
Childhood sexual abuse	Group, Public Health Institute, Centre for Addiction and Mental Health (Canada), Centre for Alcohol Policy Research, Turning Point Alcohol and Drug Centre (Australia), Kettil Bruun Society for Social and Epidemiological Research on Alcohol, University of North Dakota. Brazil - Botucatu Gender, Alcohol and Culture: An International Study (GENACIS) 2001-2002.	São Paulo, São Paulo, São Paulo	2001	Survey

Childhood sexual abuse	Federal University of Pernambuco, Feminist Collective for Health and Sexuality (São Paulo), University of São Paulo, World Health Organization (WHO). Brazil WHO Multi-country Study on Women's Health and Domestic Violence Against Women 2000-2001.	São Paulo, Pernambuco, São Paulo, Pernambuco, São Paulo, Pernambuco,	2000-2001	Survey
Childhood sexual abuse	Ipsos, National Institute of Public Policy for Alcohol and Other Drugs (INPAD) (Brazil), University of São Paulo. Brazil National Alcohol and Drugs Survey 2011-	Country	2011-2012	Survey
Childhood sexual abuse	Ipsos, National Institute of Public Policy for Alcohol and Other Drugs (INPAD) (Brazil), University of São Paulo. Brazil National Alcohol and Drugs Survey 2011-	Country	2011-2012	Survey
Childhood sexual abuse	Federal University of Pernambuco, Feminist Collective for Health and Sexuality (São Paulo), University of São Paulo, World Health Organization (WHO). Brazil WHO Multi-country Study on Women's Health and Domestic Violence Against Women 2000-2001.	São Paulo, Pernambuco, São Paulo, Pernambuco, São Paulo, Pernambuco,	2000-2001	Survey
Childhood sexual abuse	Bassani DG, Palazzo LS, Béria JU, Gigante LP, Figueiredo AC, Aerts DR, Raymann BC. Child sexual abuse in southern Brazil and associated factors: a population-		2002-2003	Scientific literature
Intimate partner violence	Brazilian Society for Family Welfare (BEMFAM), Westinghouse; Institute for Resource Development. Brazil Demographic and Health Survey 1986. Columbia, United States: Westinghouse; Institute for Resource Development.	Country	1986	Survey
Intimate partner violence	Group, Public Health Institute, Centre for Addiction and Mental Health (Canada), Centre for Alcohol Policy Research, Turning Point Alcohol and Drug Centre (Australia), Kettil Bruun Society for Social and Epidemiological Research on Alcohol, University of North Dakota. Brazil - Botucatu Gender, Alcohol and Culture: An International Study (GENACIS) 2001-2002.	São Paulo, São Paulo, São Paulo	2001	Survey
Intimate partner violence	Group, Public Health Institute, Centre for Addiction and Mental Health (Canada), Centre for Alcohol Policy Research, Turning Point Alcohol and Drug Centre (Australia), Kettil Bruun Society for Social and Epidemiological Research on Alcohol, University of North Dakota. Brazil - Botucatu Gender, Alcohol and Culture: An International Study (GENACIS) 2001-2002.	São Paulo, São Paulo, São Paulo	2001	Survey
Intimate partner violence	Schraiber LB, D'Oliveira AFPL, França Junior I. [Intimate partner sexual violence among men and women in urban Brazil, 2005]. . 2008; 127-37.		1998-2005	Scientific literature
Intimate partner violence	Zaleski M, Pinsky I, Laranjeira R, Ramisetty-Mikler S, Caetano R. Intimate partner violence and alcohol consumption. . 2010; 44(1): 53-9.		2005-2006	Scientific literature
Intimate partner violence	Zaleski M, Pinsky I, Laranjeira R, Ramisetty-Mikler S, Caetano R. Intimate partner violence and alcohol consumption. . 2010; 44(1): 53-9.		2005-2006	Scientific literature
Intimate partner violence	Lindner SR, Coelho EB, Bolsoni CC, Rojas PF, Boing AF. [Prevalence of intimate partner physical violence in men and women from Florianópolis, Santa Catarina State, Brazil: a population-based study]. . 2015; 31(4): 815-26.		2009-2010	Scientific literature
Intimate partner violence	Lindner SR, Coelho EB, Bolsoni CC, Rojas PF, Boing AF. [Prevalence of intimate partner physical violence in men and women from Florianópolis, Santa Catarina State, Brazil: a population-based study]. . 2015; 31(4): 815-26.		2009-2010	Scientific literature
Intimate partner violence	Brazilian Society for Family Welfare (BEMFAM), Macro International, Inc. Brazil Demographic and Health Survey 1996. Calverton, United States: Macro	Country	1996	Survey
Intimate partner violence	Federal University of Pernambuco, Feminist Collective for Health and Sexuality (São Paulo), University of São Paulo, World Health Organization (WHO). Brazil WHO Multi-country Study on Women's Health and Domestic Violence Against Women 2000-2001.	São Paulo, Pernambuco, São Paulo, Pernambuco, São Paulo, Pernambuco,	2000-2002	Survey
Intimate partner violence	Federal University of Pernambuco, Feminist Collective for Health and Sexuality (São Paulo), University of São Paulo, World Health Organization (WHO). Brazil WHO Multi-country Study on Women's Health and Domestic Violence Against Women 2000-2001.	São Paulo, Pernambuco, São Paulo, Pernambuco, São Paulo, Pernambuco,	2000-2002	Survey
Intimate partner violence	Schraiber LB, D'Oliveira AFPL, França Junior I. [Intimate partner sexual violence among men and women in urban Brazil, 2005]. . 2008; 127-37.		1998-2005	Scientific literature
Intimate partner violence	Brazilian Society for Family Welfare (BEMFAM), Macro International, Inc. Brazil Demographic and Health Survey 1996. Calverton, United States: Macro	Country	1996	Survey
Intimate partner violence	Ipsos, National Institute of Public Policy for Alcohol and Other Drugs (INPAD) (Brazil), University of São Paulo. Brazil National Alcohol and Drugs Survey 2011-	Country	2011-2012	Survey
Intimate partner violence	Brazilian Society for Family Welfare (BEMFAM), Macro International, Inc. Brazil Demographic and Health Survey 1991. Calverton, United States: Macro	Country	1991	Survey
Intimate partner violence	Brazilian Society for Family Welfare (BEMFAM), Macro International, Inc. Brazil Demographic and Health Survey 1991. Calverton, United States: Macro	Country	1991	Survey
Intimate partner violence	Ipsos, National Institute of Public Policy for Alcohol and Other Drugs (INPAD) (Brazil), University of São Paulo. Brazil National Alcohol and Drugs Survey 2011-	Country	2011-2012	Survey
Intimate partner violence	Brazilian Society for Family Welfare (BEMFAM), Westinghouse; Institute for Resource Development. Brazil Demographic and Health Survey 1986. Columbia, United States: Westinghouse; Institute for Resource Development.	Country	1986	Survey

Unsafe sex	Joint United Nations Program on HIV/AIDS (UNAIDS), Ministry of Health (Brazil). Brazil Progress Report on the Response to HIV/AIDS 2012. Geneva, Switzerland: Joint United Nations Program on HIV/AIDS (UNAIDS), 2012.	Country	2010	Report
Low physical activity	Bauman A, Bull F, Chey T, Craig CL, Ainsworth BE, Sallis JF, Bowles HR, Hagstromer M, Sjostrom M, Pratt M, IPS Group. The International Prevalence Study on Physical Activity: results from 20 countries. . 2009; 21.		2003	Scientific literature
Low physical activity	Ministry of Health (Brazil), Secretariat of Health Surveillance, Ministry of Health (Brazil). Brazil Surveillance System of Risk Factors for Chronic Diseases by	Country	2012	Survey
Low physical activity	Brazil Risk Factor Morbidity Noncommunicable Disease Survey 2002-2005	Amazonas, Ceará, Distrito Federal, Espírito Santo, Mato Grosso Do Sul, Minas Gerais, Paraná, Paraíba, Pará, Pernambuco, Rio Grande do orte, Rio Grande do Sul, Rio de Janeiro, Santa Catarina, Sergipe, São Paulo, Amazonas, Ceará, Distrito Federal, Espírito Santo, Mato Grosso Do Sul, Minas Gerais, Paraná, Paraíba, Pará, Pernambuco, Rio Grande do orte, Rio Grande do Sul, Rio de Janeiro, Santa	2002-2005	Survey
Low physical activity	Ministry of Health (Brazil), Secretariat of Health Surveillance, Ministry of Health (Brazil). Brazil Surveillance System of Risk Factors for Chronic Diseases by	Country	2007	Survey
Low physical activity	Ministry of Health (Brazil), Secretariat of Health Surveillance, Ministry of Health (Brazil), University of São Paulo. Brazil Surveillance System of Risk Factors for Chronic Diseases by Telephone Interviews 2006.	Country	2006	Survey
Low physical activity	Ministry of Health (Brazil), Secretariat of Health Surveillance, Ministry of Health (Brazil). Brazil Surveillance System of Risk Factors for Chronic Diseases by	Country	2008	Survey
Low physical activity	Ministry of Health (Brazil), Secretariat of Health Surveillance, Ministry of Health (Brazil). Brazil Surveillance System of Risk Factors for Chronic Diseases by	Country	2009	Survey
Low physical activity	Ministry of Health (Brazil), Secretariat of Health Surveillance, Ministry of Health (Brazil). Brazil Surveillance System of Risk Factors for Chronic Diseases by	Country	2010	Survey
Low physical activity	Ministry of Health (Brazil), Secretariat of Health Surveillance, Ministry of Health (Brazil). Brazil Surveillance System of Risk Factors for Chronic Diseases by	Country	2011	Survey
Low physical activity	Brazil World Health Survey 2003	Country	2002-2003	Survey
Low physical activity	Ministry of Planning, Budget, and Management (Brazil). Brazil National Health Survey 2013. Rio de Janeiro, Brazil: Brazilian Institute of Geography and Statistics (IBGE).	Country	2013	Survey
High fasting plasma glucose	National Institute of Public Health (Mexico). Mexico National Survey of Health and Nutrition 2005-2006. Cuernavaca, Mexico: National Institute of Public	Country	2006	Survey
High fasting plasma glucose	PF, Cunha MLMN, Stefanello II JVL, Brum LM, Oliveira LA, Silva CR, Ribeiro ALD. Diabetes mellitus and impaired glucose tolerance in urban adult population. . 2014; 60(2): 118-24.	Country	2010	Scientific literature
High fasting plasma glucose	Rigo JC, Vieira JL, Dalacorte RR, Reichert CL. Prevalence of metabolic syndrome in an elderly community: comparison between three diagnostic methods. .		2005	Scientific literature
High fasting plasma glucose	Marcopito LF, Rodrigues SSF, Pacheco MA, Shirassu MM, Goldfeder AJ, de Moraes MAOL. Prevalence of a set of risk factors for chronic diseases in the city		2001	Scientific literature
High fasting plasma glucose	Lima-Costa MF, Mambrini JV, Leite ML, Peixoto SV, Firmo JO, Loyola Filho AI, Gouveia MH, Leal TP, Pereira AC, Macinko J, Tarazona-Santos E. Socioeconomic Position, But Not African Genomic Ancestry, Is Associated With Blood Pressure in the Bambui-Epigen (Brazil) Cohort Study of Aging. . 2016; 67(2): 349-55.		1997	Scientific literature
High fasting plasma glucose	Marquezine GF, Oliveira CM, Pereira AC, Krieger JE, Mill JG. Metabolic syndrome determinants in an urban population from Brazil: social class and		2003	Scientific literature
High fasting plasma glucose	TM, Torquato MTCG, Souza GMD, Oishi J, Leal AMO. Prevalence of metabolic syndrome and its association with educational inequalities among Brazilian adults: a population-based study. . 2011; 44(7): 7139.		2007	Scientific literature
High fasting plasma glucose	Ministry of Planning, Budget, and Management (Brazil). Brazil National Health Survey 2013. Rio de Janeiro, Brazil: Brazilian Institute of Geography and Statistics (IBGE).	Country	2013	Survey

High fasting plasma glucose	Lessa I, Magalhães L, Araújo MJ, de Almeida Filho N, Aquino E, Oliveira MM. Arterial hypertension in the adult population of Salvador (BA)--Brazil. . 2006;		1999	Scientific literature
High total cholesterol	Garcez MR, Pereira JL, Fontanelli M de M, Marchioni DML, Fisberg RM. Prevalence of dyslipidemia according to the nutritional status in a		2008	Scientific literature
High total cholesterol	Passos VM de A, Barreto SM, Diniz LM, Lima-Costa MF. Type 2 diabetes: prevalence and associated factors in a Brazilian community--the Bambuí health		1996	Scientific literature
High total cholesterol	Krieger JE, Nascimento Neto RM, Chagas ACP, Hearts of Brazil Study and Peripheral Arterial Disease Committee of the Brazilian Society of Cardiology/Funcor. Prevalence and risk factors associated with peripheral arterial disease in the Hearts of Brazil Project. . 2008; 91(6): 370-82.		2004	Scientific literature
High total cholesterol	Chagas SV, Costa DM, Vianna Araujo D, Garcia Rosa ML. Racial differences in HbA1c: a cross-sectional analysis of a Brazilian public primary care population. . 2013; 7(2): 135□41.		2006	Scientific literature
High total cholesterol	Olinto MTA, Gigante DP, Horta B, Silveira V, Oliveira I, Willett W. Major dietary patterns and cardiovascular risk factors among young Brazilian adults. . 2012;		2004	Scientific literature
High total cholesterol	Gonçalves GC, Soler JMP, Andrade M de, Lorenzi-Filho G, Vallada H, Taporoski TP, Pedrazzoli M, Azambuja AP, Oliveira CM de, Alvim RO, Krieger JE, Pereira AC. Cohort profile: the Baependi Heart Study□ family-based, highly admixed cohort study in a rural Brazilian town. . 2016; 6(10): 011598.		2005	Scientific literature
High total cholesterol	Rigo JC, Vieira JL, Dalacorte RR, Reichert CL. Prevalence of metabolic syndrome in an elderly community: comparison between three diagnostic methods. .		2005	Scientific literature
High total cholesterol	Schaan BD, Harzheim E, Gus I. [Cardiac risk profile in diabetes mellitus and impaired fasting glucose]. . 2004; 38(4): 529-36.		1999	Scientific literature
High total cholesterol	Lima-Costa MF, Firmo JOA, Uchoa E. Cohort profile: the Bambui (Brazil) Cohort Study of Ageing. . 2011; 40(4): 862□.		1997	Scientific literature
High total cholesterol	Freitas MPD, Loyola Filho AI de, Lima-Costa MF. Birth cohort differences in cardiovascular risk factors in a Brazilian population of older elderly: the Bambuí Cohort Study of Aging (1997 and 2008). . 2011; S409-417.		1997-2008	Scientific literature
High systolic blood pressure	Olinto MTA, Gigante DP, Horta B, Silveira V, Oliveira I, Willett W. Major dietary patterns and cardiovascular risk factors among young Brazilian adults. . 2012;		2004	Scientific literature
High systolic blood pressure	Lima-Costa MF, Firmo JOA, Uchoa E. Cohort profile: the Bambui (Brazil) Cohort Study of Ageing. . 2011; 40(4): 862□.		1997	Scientific literature
High systolic blood pressure	Rigo JC, Vieira JL, Dalacorte RR, Reichert CL. Prevalence of metabolic syndrome in an elderly community: comparison between three diagnostic methods. .		2005	Scientific literature
High systolic blood pressure	Passos VM de A, Barreto SM, Diniz LM, Lima-Costa MF. Type 2 diabetes: prevalence and associated factors in a Brazilian community--the Bambuí health		1996	Scientific literature
High systolic blood pressure	Krieger JE, Nascimento Neto RM, Chagas ACP, Hearts of Brazil Study and Peripheral Arterial Disease Committee of the Brazilian Society of Cardiology/Funcor. Prevalence and risk factors associated with peripheral arterial disease in the Hearts of Brazil Project. . 2008; 91(6): 370-82.		2005	Scientific literature
High systolic blood pressure	Lima-Costa MF, Mambrini JV, Leite ML, Peixoto SV, Firmo JO, Loyola Filho AI, Gouveia MH, Leal TP, Pereira AC, Macinko J, Tarazona-Santos E. Socioeconomic Position, But Not African Genomic Ancestry, Is Associated With Blood Pressure in the Bambui-Epigen (Brazil) Cohort Study of Aging. . 2016; 67(2): 349-55.		1997	Scientific literature
High systolic blood pressure	Gonçalves GC, Soler JMP, Andrade M de, Lorenzi-Filho G, Vallada H, Taporoski TP, Pedrazzoli M, Azambuja AP, Oliveira CM de, Alvim RO, Krieger JE, Pereira AC. Cohort profile: the Baependi Heart Study□ family-based, highly admixed cohort study in a rural Brazilian town. . 2016; 6(10): 011598.		2005	Scientific literature
High systolic blood pressure	Marcopito LF, Rodrigues SSF, Pacheco MA, Shirassu MM, Goldfeder AJ, de Moraes MAOL. Prevalence of a set of risk factors for chronic diseases in the city		2002	Scientific literature
High systolic blood pressure	Lessa I, Magalhães L, Araújo MJ, de Almeida Filho N, Aquino E, Oliveira MM. Arterial hypertension in the adult population of Salvador (BA)--Brazil. . 2006;		1999	Scientific literature
High systolic blood pressure	Schaan BD, Harzheim E, Gus I. [Cardiac risk profile in diabetes mellitus and impaired fasting glucose]. . 2004; 38(4): 529-36.		1999	Scientific literature
High systolic blood pressure	Public Health Department, Federal University of Santa Catarina. Brazil - Florianopolis Epidemiological Study on the Health Status of the Adult	Santa Catarina, Santa Catarina	2009	Survey
High systolic blood pressure	Freitas MPD, Loyola Filho AI de, Lima-Costa MF. Birth cohort differences in cardiovascular risk factors in a Brazilian population of older elderly: the Bambuí Cohort Study of Aging (1997 and 2008). . 2011; S409-417.		1997-2008	Scientific literature
High systolic blood pressure	Castro RA, Moncau JE, Marcopito LF. Hypertension prevalence in the city of Formiga, MG, Brazil. . 2007; 88(3): 334-9.		2004	Scientific literature
High systolic blood pressure	Almeida RC, Dias DJL, Deguchi KTP, Spesia CH, Coelho OR. Prevalence and treatment of hypertension in urban and riverside areas in Porto Velho, the		2013	Scientific literature
High systolic blood pressure	Fattori A, Santimaria MR, Alves RMA, Guariento ME, Neri AL. Influence of blood pressure profile on frailty phenotype in community-dwelling elders in Brazil -		2008	Scientific literature
High systolic blood pressure	Silva DA, Petroski EL, Peres MA. Prehypertension and hypertension among adults in a metropolitan area in Southern Brazil: population-based study. . 2012;		2009	Scientific literature
High systolic blood pressure	J, Vinueza R, Schargrodsky H, Champagne B, Pramparo P, Wilson E, CARMELA Study Investigators. Hypertension in seven Latin American cities: the Cardiovascular Risk Factor Multiple Evaluation in Latin America (CARMELA) study. . 2010; 28(1): 24□34.		2004	Scientific literature
High systolic blood pressure	de Lolio CA. Prevalência da hipertensão arterial em Araraquara. . 1990; 55(3):		1987	Scientific literature
High systolic blood pressure	Pereira JC, Barreto SM, Passos VM de A. [Cardiovascular risk profile and health self-evaluation in Brazil: a population-based study]. . 2009; 25(6): 491-8.		2003	Scientific literature

High systolic blood pressure	CR, Santos FC, Bilton T, Ebel SJ, Macedo MB, Almada CM, Nasri F, Miranda RD, Gonçalves M, Santos AL, Fraietta R, Vivacqua I, Alves ML, Tudisco ES. Two-year follow-up study of elderly residents in S. Paulo, Brazil: methodology and preliminary results. . 1998; 32(5): 397-407.		1992	Scientific literature
High systolic blood pressure	Moraes RS, Fuchs FD, Moreira LB, Wiehe M, Pereira GM, Fuchs SC. Risk factors for cardiovascular disease in a Brazilian population-based cohort study. . 2003;		1995	Scientific literature
High systolic blood pressure	Hartmann M, Dias-da-Costa JS, Anselmo Olinto MT, Pattussi MP, Tramontini A. Prevalência de hipertensão arterial sistêmica e fatores associados: um estudo de base populacional em mulheres no Sul do Brasil. . 2007; 23(8): 1857-66.		2003	Scientific literature
High systolic blood pressure	R, Marafiotti Gonçalves R, Arenales de Lima S, Bulgarelli Bestetti R. Prevalence of hypertension in the urban population of Catanduva, in the State of São Paulo, Brazil. . 2001; 77(1): 9-21.		1998	Scientific literature
High systolic blood pressure	Fornes NS, Martins IS, Velasquez-Melendez G, Latorre Mdo R. Escores de consumo alimentar e níveis lipêmicos em população de São Paulo, Brasil. .		1991	Scientific literature
High body-mass index	Marcellino C, Henn RL, Olinto MT, Bressan AW, Paniz VM, Pattussi MP. Physical inactivity and associated factors among women from a municipality in southern		2006	Scientific literature
High body-mass index	Ministry of Health (Brazil), Secretariat of Health Surveillance, Ministry of Health (Brazil). Brazil Surveillance System of Risk Factors for Chronic Diseases by	Country	2010	Survey
High body-mass index	Ministry of Health (Brazil), Secretariat of Health Surveillance, Ministry of Health (Brazil). Brazil Surveillance System of Risk Factors for Chronic Diseases by	Country	2012	Survey
High body-mass index	Mintem GC, Horta BL, Domingues MR, Gigante DP. Body size dissatisfaction among young adults from the 1982 Pelotas birth cohort. . 2015; 69(1): 5561.		2004	Scientific literature
High body-mass index	Ministry of Health (Brazil), Secretariat of Health Surveillance, Ministry of Health (Brazil). Brazil Surveillance System of Risk Factors for Chronic Diseases by	Country	2011	Survey
High body-mass index	Amaral C de A, Portela MC, Muniz PT, Farias E dos S, Araujo TS de, Souza OF de. Association of handgrip strength with self-reported diseases in adults in Rio Branco, Acre State, Brazil: a population-based study. . 2015; 31(6): 131325.		2007	Scientific literature
High body-mass index	Brazil - Hearts of Brazil Survey 2004	Country	2005	Survey
High body-mass index	Guarita-Souza LC, Olandoski M, Faria-Neto JR. Elevated blood pressure and obesity in childhood: a cross-sectional evaluation of 4,609 schoolchildren. . 2014; 103(3): 23844.		2006	Scientific literature
High body-mass index	Ministry of Health (Brazil), Secretariat of Health Surveillance, Ministry of Health (Brazil), University of São Paulo. Brazil Surveillance System of Risk Factors for Chronic Diseases by Telephone Interviews 2006.	Country	2006	Survey
High body-mass index	Leal DB, de Assis MAA, Gonzalez-Chica DA, da Costa FF. Trends in adiposity in Brazilian 7-10-year-old schoolchildren evidence for increasing overweight but not obesity between 2002 and 2007. . 2014; 41(3): 25562.		2002-2007	Scientific literature
High body-mass index	Ministry of Health (Brazil), Secretariat of Health Surveillance, Ministry of Health (Brazil). Brazil Surveillance System of Risk Factors for Chronic Diseases by	Country	2008	Survey
High body-mass index	Reigota RB, Pedro AO, de Souza Santos Machado V, Costa-Paiva L, Pinto-Neto AM. Prevalence of urinary incontinence and its association with multimorbidity in women aged 50 years or older: A population-based study. . 2016; 35(1): 628.		2011	Scientific literature
High body-mass index	FAO Supply Utilization Accounts 1961-2013	Global	1990-2015	Administrative record
High body-mass index	Brazilian Society for Family Welfare (BEMFAM), Macro International, Inc. Brazil Demographic and Health Survey 1996. Calverton, United States: Macro	Country	1996	Survey
High body-mass index	Ministry of Health (Brazil), Secretariat of Health Surveillance, Ministry of Health (Brazil). Brazil Surveillance System of Risk Factors for Chronic Diseases by	Country	2007	Survey
High body-mass index	Brazilian Society for Family Welfare (BEMFAM), Westinghouse; Institute for Resource Development. Brazil Demographic and Health Survey 1986. Columbia, United States: Westinghouse; Institute for Resource Development.	Country	1986	Survey

High body-mass index	Brazil Risk Factor Morbidity Noncommunicable Disease Survey 2002-2005	Amazonas, Ceará, Distrito Federal, Espírito Santo, Mato Grosso Do Sul, Minas Gerais, Paraná, Paraíba, Pará, Pernambuco, Rio Grande do orte, Rio Grande do Sul, Rio de Janeiro, Santa Catarina, Sergipe, São Paulo, Amazonas, Ceará, Distrito Federal, Espírito Santo, Mato Grosso Do Sul, Minas Gerais, Paraná, Paraíba, Pará, Pernambuco, Rio Grande do orte, Rio Grande do Sul, Rio de Janeiro, Santa	2003	Survey
High body-mass index	Ministry of Health (Brazil), Secretariat of Health Surveillance, Ministry of Health (Brazil). Brazil Surveillance System of Risk Factors for Chronic Diseases by	Country	2009	Survey
High body-mass index	Pimenta AM, Kac G, Gazzinelli A, Corrêa-Oliveira R, Velásquez-Meléndez G. Association between central obesity, triglycerides and hypertension in a rural		2001	Scientific literature
High body-mass index	Reichert FF, Azevedo MR, Breier A, Gerage AM. Physical activity and prevalence of hypertension in a population-based sample of Brazilian adults and elderly. .		2003	Scientific literature
High body-mass index	Bustos P, da Silva AAM, Amigo H, Bettiol H, Barbieri MA. Metabolic syndrome in young adults from two socioeconomic Latin American settings. . 2007; 17(8):		2003	Scientific literature
High body-mass index	Brazil Consumer Expenditure Survey 2002-2003	Country	2002	Survey
High body-mass index	Garcez MR, Pereira JL, Fontanelli M de M, Marchioni DML, Fisberg RM. Prevalence of dyslipidemia according to the nutritional status in a		2008	Scientific literature
High body-mass index	Brazil World Health Survey 2003	Country	2003	Survey
High body-mass index	Brazil - São Paulo Survey on Health, Well-Being, and Aging in Latin America and the Caribbean 1999-2000	São Paulo	1999	Survey
High body-mass index	Brazilian Institute of Geography and Statistics (IBGE). Brazil National Household Sample Survey 2006. Rio de Janeiro, Brazil: Brazilian Institute of Geography and	Country	2006	Survey
High body-mass index	de Freitas SN, Caiaffa WT, César CC, Faria VA, do Nascimento RM, Coelho GL. Nutritional Risk in the Urban Population of Ouro Preto, Southeastern Region of Brazil: The Ouro Preto Heart Study. . 2007; 88(2): 191-9.		2001	Scientific literature
High body-mass index	Santos Silva DA, Petroski EL, Peres MA. Is high body fat estimated by body mass index and waist circumference a predictor of hypertension in adults? A		2009	Scientific literature
High body-mass index	Machado EC, Silveira MF da, Silveira VMF da. Prevalence of weight-loss strategies and use of substances for weight-loss among adults: a population		2010	Scientific literature
High body-mass index	Group, Public Health Institute, Centre for Addiction and Mental Health (Canada), Centre for Alcohol Policy Research, Turning Point Alcohol and Drug Centre (Australia), Kettil Bruun Society for Social and Epidemiological Research on Alcohol, University of North Dakota. Brazil - Botucatu Gender, Alcohol and Culture: An International Study (GENACIS) 2001-2002.	São Paulo, São Paulo, São Paulo	2001	Survey
High body-mass index	Jaime PC, Duran AC, Sarti FM, Lock K. Investigating environmental determinants of diet, physical activity, and overweight among adults in Sao Paulo, Brazil. .		2003	Scientific literature
High body-mass index	Ramos de Marins VM, Varnier Almeida RM, Pereira RA, Barros MB. Factors associated with overweight and central body fat in the city of Rio de Janeiro: results of a two-stage random sampling survey. . 2001; 115(3): 236-42.		1995	Scientific literature
High body-mass index	Abrantes MM, Lamounier JA, Colosimo EA. Prevalência de sobrepeso e obesidade nas regiões Nordeste e Sudeste do Brasil. . 2003; 49(2): 162-6.		1997	Scientific literature
High body-mass index	Dalla Vecchia CF, Susin C, Rösing CK, Oppermann RV, Albandar JM. Overweight and obesity as risk indicators for periodontitis in adults. . 2005; 76(10): 1721-8.		2001	Scientific literature
High body-mass index	De Assis MAA, Rolland-Cachera MF, de Vasconcelos FAG, Bellisle F, Conde W, Calvo MCM, Luna MEP, Ireton MJ, Grosseman S. Central adiposity in Brazilian schoolchildren aged 7-10 years. . 2007; 97(4): 799-805.		2002	Scientific literature
High body-mass index	MEP, Calvo MCM, Barros MVG, Pires MMS, Bellisle F. Obesity, overweight and thinness in schoolchildren of the city of Florianópolis, Southern Brazil. . 2005; 59(9): 1015-21.		2002	Scientific literature
High body-mass index	Guimarães JMN, de Souza Lopes C, Baima J, Sichieri R. Depression symptoms and hypothyroidism in a population-based study of middle-aged Brazilian		2004	Scientific literature

High body-mass index	Oliveira AM, Oliveira AC, Almeida MS, Oliveira N, Adan L. Influence of the family nucleus on obesity in children from northeastern Brazil: a cross-sectional study.		2005	Scientific literature
High body-mass index	De Andrade FB, de França Caldas A Jr, Kitoko PM. Relationship between oral health, nutrient intake and nutritional status in a sample of Brazilian elderly		2006	Scientific literature
High body-mass index	PF, Cunha MLMN, Stefanello II JVL, Brum LM, Oliveira LA, Silva CR, Ribeiro ALD. Diabetes mellitus and impaired glucose tolerance in urban adult population. . 2014; 60(2): 118-24.	Country	2010	Scientific literature
High body-mass index	Brazil Living Standards Measurement Survey 1996-1997	Ceará, Espírito Santo, Maranhão, Minas Gerais, Paraíba, Pernambuco, Piaui, Rio Grande do orte, Rio de Janeiro, Sergipe, São Paulo, Alagoas, Bahia, Ceará, Espírito Santo, Maranhão, Minas Gerais,	1996	Survey
Low bone mineral density	Zerbini CA, Latorre MR, Jaime PC, Tanaka T, Pippa MG. Bone mineral density in Brazilian men 50 years and older. . 2000; 33(12): 1429-35.		1997	Scientific literature
Low bone mineral density	Szejnfeld VL, Atra E, Baracat EC, Aldrighi JM, Civitelli R. Bone density in white Brazilian women: Rapid loss at the time around the menopause. . 1995; 56(3):		1992	Scientific literature
Low bone mineral density	Camargo MBR, Cendoroglo MS, Ramos LR, de Oliveira Latorre M do RD, Saraiva GL, Lage A, Carvalhaes Neto N, Araújo LMQ, Vieira JGH, Lazaretti-Castro M. Bone mineral density and osteoporosis among a predominantly Caucasian elderly population in the city of São Paulo, Brazil. . 2005; 16(11): 1451-60.		1998-1999	Scientific literature
Low bone mineral density	quantitative ultrasound are risk factors for new osteoporotic fracture and total and cardiovascular mortality: a 5-year population-based study of Brazilian elderly women. . 2006; 61(2): 196-203.		1997-2002	Scientific literature
Impaired kidney function	D, Griep RH, Vidigal PG, Ribeiro AL, Lotufo PA, Mill JG. Chronic kidney disease among adult participants of the ELSA-Brasil cohort: association with race and socioeconomic position. . 2016; 70(4): 380-9.		2008-2010	Scientific literature
Impaired kidney function	Pereira ER, Pereira Ade C, Andrade GB, Naghettini AV, Pinto FK, Batista SR, Marques SM. Prevalence of chronic renal disease in adults attended by the		2011-2013	Scientific literature

Appendix Table 4. Guidelines for Accurate and Transparent Health Estimates Reporting (GATHER) checklist of information that should be included in reports of global health estimates and description of compliance and location of information for "Burden of disease in Brazil, 1990 to 2016: a systematic review for the Global Burden of Disease (GBD) Study 2016."			
**#**	**GATHER checklist item**	**Description of compliance**	**Reference**
**Objectives and funding**			
1	Define the indicators, populations, and time periods for which estimates were made.	Narrative in the main text and in the methods appendix describing indicators, definitions, and populations	Main text (Methods) and methods appendix
2	List the funding sources for the work.	Funding sources listed in summary section	Summary (Funding)
**Data Inputs**			
For all data inputs from multiple sources that are synthesized as part of the study:			
3	Describe how the data were identified and how the data were accessed.	Narrative of data seeking methods detailed in the GBD 2016 publications* and methods appendices**	GBD 2016 Mortality appendix (pgs 6-9, 21-25, 53, 58-60), Cause of death appendix (pgs 9-13) Years lived with disability (YLDs) appendix (pgs 6-17)
4	Specify the inclusion and exclusion criteria. Identify all ad-hoc exclusions.	Narrative of inclusion and exclusion criteria by data type detailed in the GBD 2016 publications* and methods appendices**	GBD Mortality appendix (pgs 6-9, 21-25, 53, 58-60) Cause of death appendix (by cause pgs 39-278), YLDs appendix (by cause pgs 34-716)
5	Provide information on all included data sources and their main characteristics. For each data source used, report reference information or contact name/institution, population represented, data collection method, year(s) of data collection, sex and age range, diagnostic criteria or measurement method, and sample size, as relevant.	An interactive, online data source tool providing metadata for data sources by component, geography, cause, risk, or impairment is available	Online data citation tools http://ghdx.healthdata.org/<br><br>Methods appendix tables 1-4
6	Identify and describe any categories of input data that have potentially important biases (e.g., based on characteristics listed in item 5).	A summary of known biases by cause is detailed in the GBD 2016 publications* and methods appendices **	Main Text, Limitations section in GBD Mortality, Cause of death, and YLD papers<br><br>Cause of death appendix (by cause pgs 39-278), YLDs appendix (by cause pgs 34-716)
For data inputs that contribute to the analysis but were not synthesized as part of the study:			
7	Describe and give sources for any other data inputs.	Sources are included in the online data source tool	Online data citation tools http://ghdx.healthdata.org/

For all data inputs:			
8	Provide all data inputs in a file format from which data can be efficiently extracted (e.g., a spreadsheet as opposed to a PDF), including all relevant meta-data listed in item 5. For any data inputs that cannot be shared due to ethical or legal reasons, such as third-party ownership, provide a contact name or the name of the institution that retains the right to the data.	Input data available for download through online tools, including data visualization and data query tools. Input data not available in tools will be made available upon request	Online data visualization tools, data query tools, and the Global Health Data Exchange www.healthdata.org/results/data-visualizations; http://ghdx.healthdata.org/gb-results-tool; http://ghdx.healthdata.org/
**Data analysis**			
9	Provide a conceptual overview of the data analysis method. A diagram may be helpful.	Flow diagrams of the overall methodological processes, as well as cause-specific modelling processes, detailed in the GBD 2016 publications* and methods appendices**	GBD Mortality appendix figure 1, Cause of death appendix figures 1-3, YLDs appendix figures 1-2
10	Provide a detailed description of all steps of the analysis, including mathematical formulae. This description should cover, as relevant, data cleaning, data pre-processing, data adjustments and weighting of data sources, and mathematical or statistical model(s).	Flow diagrams and corresponding methodological write- ups for each cause, as well as the demographics and causes of death databases and modelling processes, detailed in the GBD 2016 publications* and methods appendices**	GBD Mortality appendix figure 1, Cause of death appendix figures 1-3, YLDs appendix figures 1-2<br><br>GBD Cause of death appendix (by cause pgs 39-278) YLDs appendix (by cause pgs 34-716)
11	Describe how candidate models were evaluated and how the final model(s) were selected.	Detailed in the methodological write- ups in the GBD 2016 publications* and methods appendices **	GBD Cause of death appendix (by cause pgs 39-278) YLDs appendix (by cause pgs 34-716)
12	Provide the results of an evaluation of model performance, if done, as well as the results of any relevant sensitivity analysis.	Detailed in the methodological write- up in the GBD 2016 publications* and methods appendices **	GBD Cause of death appendix (by cause pgs 39-278) YLDs appendix (by cause pgs 34-716)

13	Describe methods for calculating uncertainty of the estimates. State which sources of uncertainty were, and were not, accounted for in the uncertainty analysis.	Narrative in the main paper<br><br>Further information detailed in the GBD 2016 publications* and methods appendices**	Main text (Methods)<br><br>GBD Cause of death appendix (pgs 9, 33-35, YLD appendix (pgs 10, 28-29)
14	State how analytic or statistical source code used to generate estimates can be accessed.	Access statement Included in the main paper	Main text (Methods)<br><br>Code is provided in an online repository https://github.com/ihmeuw/ihme-modeling
**Results and Discussion**			
15	Provide published estimates in a file format from which data can be efficiently extracted.	GBD 2016 results are available through online data visualization tools, the Global Health Data Exchange, and the online data query tool	Main text and online data tools (data visualization tools, data query tools, and the Global Health Data Exchange) www.healthdata.org/results/data-visualizations; http://ghdx.healthdata.org/gb-results-tool; http://ghdx.healthdata.org/
16	Report a quantitative measure of the uncertainty of the estimates (e.g. uncertainty intervals).	Uncertainty intervals provided with all results	Main text (Methods, results), methods appendix and online data tools (data visualization tools, data query tools, and The Global Health Data Exchange) www.healthdata.org/results/data-visualizations; http://ghdx.healthdata.org/gb-results-tool; http://ghdx.healthdata.org/
17	Interpret results in light of existing evidence. If updating a previous set of estimates, describe the reasons for changes in estimates.	Discussion of methodological changes between GBD rounds in the research in context section of the main paper	Research in context
18	Discuss limitations of the estimates. Include a discussion of any modelling assumptions or data limitations that affect interpretation of the estimates.	Discussion of limitations in the narrative of the main paper	Main text (Discussion, limitations)

**Gakidou E, Afshin A, Abajobir AA, et al. Global, regional, and national comparative risk assessment of 84 behavioural, environmental and occupational, and metabolic risks or clusters of risks, 1990–2016: a systematic analysis for the Global Burden of Disease Study 2016. The Lancet 2017; 390: 1345–422.*

***Supplementary appendix 1*

**Hay SI, Abajobir AA, Abate KH, et al. Global, regional, and national disability-adjusted life-years (DALYs) for 333 diseases and injuries and healthy life expectancy (HALE) for 195 countries and territories, 1990–2016: a systematic analysis for the Global Burden of Disease Study 2016. The Lancet 2017; 390: 1260–344.*

*** Supplementary appendix 1*
**Naghavi M, Abajobir AA, Abbafati C, et al. Global, regional, and national age-sex specific mortality for 264 causes of death, 1980–2016: a systematic analysis for the Global Burden of Disease Study 2016. The Lancet 2017; 390: 1151–210.*
*** Supplementary appendix 1*
**Vos T, Abajobir AA, Abate KH, et al. Global, regional, and national incidence, prevalence, and years lived with disability for 328 diseases and injuries for 195 countries, 1990–2016: a systematic analysis for the Global Burden of Disease Study 2016. The Lancet 2017; 390: 1211–59.*
*** Supplementary appendix 1*
**Wang H, Abajobir AA, Abate KH, et al. Global, regional, and national under-5 mortality, adult mortality, age-specific mortality, and life expectancy, 1970–2016: a systematic analysis for the Global Burden of Disease Study 2016. The Lancet 2017; 390: 1084–150.*
*** Supplementary appendix 1*

Appendix Figure 1

Age-standardized DALY rates (per 100,000) of top GBD level 3 causes in Brazil, its 26 states, and the Federal District, both sexes, 1990*

	Ischemic heart disease	Interpersonal violence	Low back & neck pain	Stroke	Road injuries	Sense organ diseases	Diabetes	Lower respiratory infect	Skin diseases	Congenital defects	Alzheimer disease	Migraine	COPD	Depressive disorders	Neonatal preterm birth	Anxiety disorders	Falls	Other musculoskeletal	Chronic kidney disease	Alcohol use disorders	Diarrheal diseases	Neonatal encephalopathy
Brazil	3715.5	1478.2	1334.2	2730.7	1600.4	1023.8	966.4	2034.4	832.4	1284.5	650.7	662.2	1080.4	677.2	1670.2	521.5	526.8	457.6	530.4	384.0	2012.4	612.6
Acre	2796.9	1468.6	1323.9	2021.3	1379.1	1071.2	787.0	2304.6	786.9	1380.7	653.8	668.8	1171.3	587.9	2257.0	522.8	461.9	341.4	624.4	380.1	2508.6	851.8
Amapa	2326.7	1086.0	1336.1	1706.9	1120.9	1056.2	762.8	1167.2	814.4	1084.8	646.0	673.2	687.8	591.6	1722.5	526.0	356.9	338.8	576.1	243.0	1113.0	663.3
Amazonas	2431.3	1277.4	1332.2	1959.4	1199.4	1039.9	780.9	1477.0	818.4	1257.4	635.7	673.0	728.1	601.3	1937.4	525.4	427.9	387.1	484.2	272.7	2304.1	598.0
Para	2572.8	1190.1	1333.8	2128.8	1353.2	1073.3	627.6	1971.5	815.4	1121.7	621.7	671.3	739.5	562.4	1945.0	525.2	441.2	357.7	524.1	226.8	2928.5	566.3
Rondonia	3801.2	2269.9	1321.6	2827.2	2220.7	1045.9	952.3	1678.7	820.5	1135.7	668.8	660.5	1216.7	668.5	1631.3	517.1	565.9	345.8	596.5	310.1	1958.2	668.4
Roraima	2857.8	1824.9	1312.7	1985.4	2205.9	1035.9	1255.9	2156.7	844.8	1097.8	660.2	640.3	701.8	733.7	1996.9	504.8	444.8	315.1	680.4	297.4	2081.6	831.8
Tocantins	2395.7	938.8	1324.7	1990.0	1643.7	1104.7	682.5	1758.6	817.8	1507.2	631.2	667.9	691.5	648.1	1835.6	522.4	406.3	321.6	403.9	313.4	1886.5	823.5
Alagoas	3196.6	1883.3	1339.3	3393.2	2049.2	1086.6	1267.7	4090.8	804.9	1618.8	638.4	682.4	1019.6	667.2	2108.7	531.8	483.9	380.5	591.0	315.4	5944.8	986.0
Bahia	2673.6	905.2	1415.5	2169.7	1282.1	1068.6	1009.2	2083.6	878.4	875.0	622.5	681.0	753.2	557.7	2332.8	531.1	491.5	340.9	508.7	374.4	3766.7	646.4
Ceara	1881.7	956.7	1345.7	1715.8	1381.8	1077.8	573.4	2983.6	818.9	2033.0	605.5	688.3	560.3	659.3	2211.1	535.2	460.4	337.0	309.5	372.5	4723.5	715.0
Maranhao	2222.3	1248.3	1331.7	2220.5	1587.2	1104.3	843.5	2561.1	807.1	1538.3	610.9	678.0	586.1	623.1	1969.1	528.9	408.5	332.3	520.5	294.4	3838.8	1306.3
Paraiba	2713.6	1225.1	1347.8	2075.6	1324.0	1075.4	995.8	2280.7	819.0	1809.7	643.4	690.3	733.7	632.1	1345.3	536.3	349.2	379.9	559.9	338.7	2493.3	743.5
Pernambuco	3623.8	2372.1	1343.2	2964.0	1544.7	1055.9	1214.1	3036.6	820.9	1875.6	642.2	687.5	993.6	705.6	2090.4	534.2	411.2	332.3	538.1	394.9	4540.8	643.9
Piaui	2098.1	674.6	1342.2	2077.0	1264.6	1093.6	615.3	1751.4	798.0	1282.2	617.2	685.3	516.9	630.2	1935.2	533.3	331.8	362.1	326.0	272.2	3067.8	687.1
Rio Grande do Norte	2326.4	875.1	1344.8	1567.5	1373.2	1064.4	900.9	2369.2	834.2	1610.7	636.7	686.6	404.9	683.5	2010.6	533.9	324.1	345.5	355.5	321.9	3179.3	633.5
Sergipe	2512.1	1412.1	1336.7	2500.2	1789.9	1060.2	1363.2	1861.3	820.9	1417.1	650.7	683.5	775.1	669.4	2195.7	532.3	494.3	351.0	561.4	501.4	3769.1	862.8
Distrito Federal	3228.8	1449.3	1350.4	2314.3	1869.2	937.4	961.7	1074.0	842.3	1247.1	663.1	688.4	863.2	642.5	1132.4	535.2	610.3	389.3	516.0	399.3	595.7	552.0
Goias	3526.6	1532.2	1331.0	2905.8	2291.6	1037.6	797.9	1393.2	821.2	1212.0	651.3	673.8	1626.6	732.4	1362.1	525.5	591.9	339.5	552.5	374.2	886.0	453.6
Mato Grosso	3567.4	1373.6	1326.9	2000.1	1937.2	1033.8	769.5	1489.0	825.0	1086.0	650.2	662.7	932.5	613.0	1663.3	519.1	540.1	326.5	528.4	294.6	1263.3	429.3
Mato Grosso do Sul	3869.3	1438.8	1334.5	2714.0	1745.2	1023.9	761.5	1419.1	820.1	1068.1	654.2	672.5	995.5	696.9	1316.4	525.3	566.9	355.6	531.0	377.9	944.1	417.1
Espirito Santo	3590.7	1892.4	1334.5	3490.7	1925.0	1006.8	859.3	1183.4	839.5	1496.6	648.3	677.7	967.3	632.5	1468.6	528.8	669.3	335.7	526.4	437.6	1025.0	568.2
Minas Gerais	3828.2	964.0	1333.1	2977.4	1406.5	1025.6	951.3	1628.7	815.6	1265.0	646.0	648.4	1334.2	679.5	1852.2	529.2	510.2	366.3	624.7	501.1	979.3	526.4
Rio de Janeiro	5149.6	2919.5	1344.3	3649.0	1730.7	978.7	1411.6	2118.4	859.8	1194.2	647.8	687.5	1264.7	672.1	1579.0	534.8	582.8	368.3	616.7	358.3	806.2	460.3
Sao Paulo	4343.5	1687.3	1335.8	2614.7	1666.4	983.1	972.4	2014.5	828.3	1143.2	695.8	608.5	1062.0	701.2	1287.2	490.7	657.2	805.9	548.2	431.1	670.2	563.5
Parana	4197.0	1069.9	1338.2	3491.8	1778.7	1017.6	841.4	1236.0	799.6	1169.2	653.2	678.0	1418.9	700.0	1119.5	528.8	536.4	312.7	471.4	394.0	988.0	491.4
Rio Grande do Sul	4195.0	1131.5	1206.6	2743.5	1231.9	1001.1	759.2	1238.1	859.7	952.0	655.4	697.2	1562.1	777.0	933.4	531.4	437.2	366.9	468.0	303.7	436.8	384.2
Santa Catarina	3882.0	806.3	1338.1	2963.3	2084.7	1021.3	853.0	1355.2	825.4	1286.5	647.9	677.7	1595.9	782.7	1090.7	528.8	416.3	349.8	457.2	296.7	851.2	495.4

Significantly lower than national mean
Indistinguishable from national mean
Significantly higher than national mean

*Diarrheal diseases and neonatal encephalopathy are also included, where they were top causes of DALYs in 1990 but were not a leading cause in 2016.
DALYs: Disability-adjusted life-years; GBD: Global burden of disease; COPD: Chronic obstructive pulmonary disease

Appendix Figure 2

Age-standardized DALY rates (per 100,000) of top GBD level 3 causes in Brazil, its 26 states, and the Federal District, both sexes, 2016*

	Ischemic heart disease	Interpersonal violence	Low back & neck pain	Stroke	Road injuries	Sense organ diseases	Diabetes	Lower respiratory infect	Skin diseases	Congenital defects	Alzheimer disease	Migraine	COPD	Depressive disorders	Neonatal preterm birth	Anxiety disorders	Falls	Other musculoskeletal	Chronic kidney disease	Alcohol use disorders	Diarrheal diseases	Neonatal encephalopathy
Brazil	1927.1	1488.7	1275.8	1169.9	1151.5	973.4	882.1	869.8	865.0	790.7	671.3	657.9	633.1	620.2	580.6	572.2	511.7	475.3	459.7	413.0	319.9	300.9
Acre	1718.1	1491.5	1270.5	1284.8	1036.3	997.9	946.0	1123.0	821.6	953.8	664.5	668.7	970.4	582.9	918.9	577.8	565.9	342.1	672.4	428.4	493.5	471.3
Amapa	1615.3	1763.1	1274.6	1293.1	1018.1	985.3	947.0	911.6	862.6	786.1	665.3	669.1	569.0	594.6	751.1	578.0	414.3	344.3	658.3	299.3	273.4	468.8
Amazonas	1542.7	1670.7	1276.8	1260.8	790.9	979.4	997.7	937.4	858.3	798.4	654.7	670.5	564.7	556.4	582.4	579.4	455.4	416.7	576.8	330.2	408.9	331.6
Para	1857.3	1915.6	1271.7	1407.1	1167.4	1006.9	1039.8	1038.7	853.3	623.2	637.8	668.0	634.6	507.6	559.7	577.3	376.2	390.2	537.5	270.9	495.1	288.9
Rondonia	2033.8	1516.9	1272.7	1196.3	1466.6	978.0	977.5	731.0	850.0	700.0	672.9	668.2	773.0	613.1	520.3	576.6	493.0	343.1	579.9	313.0	322.1	309.3
Roraima	1708.6	1493.4	1269.2	1068.5	1574.3	980.3	1295.2	1271.9	885.7	761.1	678.3	665.1	486.6	717.1	756.4	574.6	447.8	316.3	653.7	317.2	389.1	388.4
Tocantins	1987.5	1277.7	1266.5	1369.4	1716.7	1036.3	1022.4	632.9	848.8	880.9	646.6	667.1	519.4	642.1	622.8	576.1	527.0	315.4	514.4	447.3	320.0	416.5
Alagoas	2247.1	2573.6	1279.4	1662.0	1294.5	1009.2	1513.7	886.2	843.3	660.9	644.2	679.1	635.0	691.5	507.8	585.9	422.4	390.2	560.8	436.1	515.2	263.9
Bahia	1720.8	1887.9	1390.3	1196.6	1090.6	998.0	1099.4	760.8	909.8	863.6	628.2	673.5	580.4	544.8	704.8	581.7	477.4	349.0	510.9	517.0	471.3	355.4
Ceara	1865.2	1894.9	1279.9	1262.3	1507.6	1003.2	857.2	892.9	865.2	933.0	647.7	676.9	477.5	644.9	674.4	584.2	458.1	362.9	396.4	631.5	446.0	326.3
Maranhao	2148.0	1567.4	1272.2	1602.9	1401.2	1014.4	1283.4	860.2	845.1	925.4	624.3	674.3	426.3	601.0	613.0	582.5	415.4	348.4	535.0	369.4	578.8	484.9
Paraiba	2253.5	1782.8	1280.6	1254.2	1279.6	1003.7	1259.3	828.1	853.6	837.3	647.9	680.0	537.3	615.7	441.1	586.4	370.7	379.1	534.9	415.9	414.7	245.6
Pernambuco	2375.8	2054.2	1278.9	1328.3	1214.9	994.4	1211.3	801.4	855.3	923.9	649.1	679.4	885.4	692.4	552.5	586.0	357.8	348.3	454.0	475.9	467.0	350.0
Piaui	2145.8	1065.8	1274.3	1567.3	1670.3	1016.5	1134.5	720.4	833.0	895.5	646.8	677.6	425.6	592.1	691.3	583.9	443.9	368.4	462.3	461.5	399.4	496.0
Rio Grande do Norte	1965.7	1715.7	1279.6	942.8	1101.9	990.7	1202.3	718.1	868.1	749.5	639.8	675.4	360.8	651.5	594.7	582.6	408.1	350.5	430.1	446.6	460.4	261.7
Sergipe	1937.2	1956.3	1278.6	1429.6	1448.8	986.3	1387.0	806.5	872.2	911.6	650.3	676.7	549.8	615.6	729.2	584.1	503.6	352.5	544.1	677.4	369.7	496.6
Distrito Federal	1351.6	1387.2	1291.7	888.3	931.6	895.4	671.4	502.6	872.3	872.5	640.7	688.6	433.2	612.0	533.2	592.3	541.8	378.4	415.2	435.6	207.1	263.1
Goias	2086.7	1895.6	1271.2	1140.7	1586.2	975.1	822.8	844.9	852.7	850.0	651.8	671.0	882.4	650.8	627.9	578.8	641.9	326.6	517.5	452.9	277.9	293.7
Mato Grosso	2031.9	1691.6	1270.7	1222.0	1692.9	969.3	1015.0	794.4	858.4	667.2	658.5	665.8	783.4	593.1	470.7	574.9	532.0	344.9	550.9	411.2	296.2	226.6
Mato Grosso do Sul	2213.4	1338.5	1276.9	1247.6	1354.2	965.7	815.8	795.7	856.5	662.0	659.2	671.3	643.6	637.0	403.6	579.1	613.8	339.6	499.8	424.6	276.4	208.4
Espirito Santo	1735.5	2038.4	1273.9	1187.0	1360.9	955.7	806.6	609.9	870.7	948.8	650.4	671.7	479.2	618.7	476.8	579.7	611.6	336.2	467.6	492.1	246.7	316.8
Minas Gerais	1671.9	1265.1	1272.3	1110.6	1129.5	975.5	750.2	798.7	847.4	790.9	646.9	640.4	586.5	623.5	707.5	580.1	484.6	363.2	444.4	554.6	255.1	275.6
Rio de Janeiro	2348.6	1786.2	1289.7	1281.0	1023.2	944.4	1063.6	1116.8	896.3	745.6	655.8	678.3	638.0	580.8	524.3	585.0	469.2	390.2	580.8	315.0	213.3	300.4
Sao Paulo	1908.0	965.4	1275.3	1022.0	874.9	963.0	643.4	933.1	857.5	688.9	743.2	606.0	597.8	612.4	531.6	537.7	599.6	876.6	392.2	310.4	198.8	230.8
Parana	1892.0	1468.5	1275.4	1193.0	1395.1	958.7	900.4	641.0	835.1	793.3	655.9	674.5	782.3	626.8	490.3	581.3	628.0	303.5	417.5	460.7	250.6	261.5
Rio Grande do Sul	1787.2	1217.6	1122.8	1112.2	923.2	957.2	743.2	652.0	890.9	749.3	659.3	707.3	810.4	700.7	475.5	581.9	494.6	352.0	398.2	361.3	192.7	232.3
Santa Catarina	1736.8	802.0	1275.7	962.3	1380.8	959.7	748.8	685.0	854.7	895.3	649.2	670.7	740.0	757.6	537.2	578.9	433.8	339.7	371.4	347.5	237.8	262.3

Significantly lower than national mean

Indistinguishable from national mean

Significantly higher than national mean

*Diarrheal diseases and neonatal encephalopathy are also included, where they were top causes of DALYs in 1990 but were not a leading cause in 2016.

DALYs: Disability-adjusted life-years; GBD: Global burden of disease; COPD: Chronic obstructive pulmonary disease

Appendix Figure 3

Ranking of top GBD level 3 causes of age-standardized DALYs in Brazil and comparator countries, both sexes, 2016

	Brazil	Russia	India	China	South Africa	Mexico	Argentina	Colombia	Australia	Canada	England
Ischemic heart disease	1927.1	5240.0	4111.3	2002.1	1835.3	1700.7	1870.3	1650.5	983.1	1152.0	1139.0
Interpersonal violence	1488.7	747.1	217.8	108.3	1871.6	830.8	343.1	1745.7	94.2	124.6	54.2
Low back and neck pain	1275.8	1382.1	909.0	986.6	1104.6	909.7	1570.5	1009.7	1493.7	1372.5	1819.9
Stroke	1169.9	2939.5	1694.1	2523.2	1413.2	616.3	934.5	608.7	469.2	503.9	569.9
Road injuries	1151.5	1078.5	1017.9	1039.3	2088.9	839.4	736.1	729.0	431.4	463.3	281.9
Sense organ diseases	973.4	899.8	1315.1	873.2	1118.8	1009.1	723.5	1067.0	563.4	562.7	666.7
Diabetes mellitus	882.1	390.3	1059.4	513.7	2325.8	2213.0	696.9	694.6	407.9	547.1	288.9
Lower respiratory infections	869.8	742.5	1868.3	375.2	2536.5	560.9	941.8	456.8	134.9	178.4	338.6
Skin and subcutaneous diseases	865.0	737.5	674.3	703.0	913.2	744.8	906.3	802.0	911.8	1076.0	1067.9
Congenital birth defects	790.7	521.3	684.7	565.9	507.6	811.0	636.5	669.2	292.7	355.1	349.9
Alzheimer disease and other dementias	671.3	325.1	304.0	529.6	386.8	492.7	322.9	474.4	366.1	333.1	440.0
Migraine	657.9	704.4	722.6	380.6	504.8	638.7	670.7	660.2	739.8	685.0	719.1
Chronic obstructive pulmonary disease	633.1	341.4	2432.1	1063.9	931.4	482.3	547.7	559.2	371.7	336.8	507.4
Depressive disorders	620.2	625.7	627.3	539.5	688.2	489.1	588.2	394.4	775.9	589.0	664.1
Neonatal preterm birth complications	580.6	238.9	1308.0	486.5	837.4	500.2	574.5	559.7	187.2	296.9	349.4
Anxiety disorders	572.2	268.1	310.9	291.4	358.2	282.1	568.2	242.0	570.4	481.9	435.4
Falls	511.7	903.7	839.4	365.4	192.9	382.3	306.9	317.5	364.0	414.0	449.5
Other musculoskeletal disorders	475.3	96.1	625.6	312.1	304.3	418.7	643.4	423.9	648.1	596.3	352.4
Chronic kidney disease	459.7	228.3	727.9	322.5	791.3	1563.3	501.6	496.7	185.5	203.6	123.1
Alcohol use disorders	413.0	1119.2	199.2	149.7	197.1	263.0	215.8	178.4	170.6	212.4	170.5
Oral disorders	366.9	322.7	296.5	184.5	282.7	355.7	284.8	345.5	317.4	296.4	354.9
Iron-deficiency anemia	361.9	88.1	1231.4	85.0	275.0	115.3	124.4	84.7	31.2	38.6	84.3
Tracheal, bronchus, and lung cancer	334.8	581.2	171.2	799.3	446.6	179.5	534.8	233.7	488.9	738.6	633.1
HIV/AIDS	322.1	605.0	362.0	88.0	16894.2	197.2	200.5	223.9	22.4	52.7	24.5
Diarrheal diseases	319.9	91.2	2162.5	61.0	1274.3	207.4	95.7	180.9	24.0	48.4	49.8

Rank

1	2-3	4-6	7-10	11-17	18+

GBD: Global burden of disease; DALYs: Disability-adjusted life-years; HIV/AIDS: Human immunodeficiency virus/ Acquired immune deficiency syndrome

Appendix Figure 4

Ratio of observed and expected (O:E) age-standardized disability-adjusted life-year (DALY) rates in Brazil and comparator countries, both sexes, 2016

		Ischemic heart disease	Interpersonal violence	Low back and neck pain	Stroke	Road injuries	Sense organ diseases	Diabetes mellitus	Lower respiratory infections	Skin and subcutaneous diseases	Congenital birth defects	Alzheimer disease and other dementias	Migraine	Chronic obstructive pulmonary disease	Depressive disorders	Neonatal preterm birth complications	Anxiety disorders	Falls	Other musculoskeletal disorders	Chronic kidney disease	Alcohol use disorders
Brazil	1990	0.87	2.58	1.16	0.94	1.05	0.88	0.72	0.90	1.12	1.28	1.26	1.11	0.77	1.09	1.19	1.41	1.20	1.03	0.68	1.47
	2016	0.53	4.28	0.95	0.60	1.03	1.04	1.04	1.10	1.05	1.02	1.32	1.01	0.73	1.01	0.80	1.43	1.05	1.30	1.04	1.70
Russia	1990	1.68	3.44	1.05	2.37	1.50	1.11	0.58	1.32	0.82	1.60	0.66	1.04	0.89	1.06	1.43	0.66	1.82	0.33	0.66	4.35
	2016	1.78	3.82	0.92	2.20	1.33	1.20	0.67	1.87	0.85	1.01	0.70	1.03	0.65	1.03	0.66	0.65	1.69	0.33	0.83	5.15
India	1990	0.94	0.61	0.87	0.69	0.65	1.06	0.62	0.90	0.86	0.90	0.55	1.26	2.27	1.01	1.22	0.95	1.69	1.67	0.92	0.98
	2016	1.00	0.40	0.77	0.63	0.71	1.18	0.86	1.07	0.89	0.70	0.59	1.19	1.90	1.01	1.05	0.82	1.92	1.43	1.05	0.76
China	1990	0.42	0.47	1.00	1.41	0.80	0.75	0.36	0.97	0.93	1.14	1.02	0.65	2.45	0.99	1.10	0.83	0.89	0.78	0.55	0.55
	2016	0.56	0.34	0.72	1.36	0.97	0.96	0.64	0.52	0.85	0.77	1.05	0.58	1.31	0.88	0.72	0.72	0.74	0.88	0.78	0.62
South Africa	1990	0.46	4.96	0.93	0.60	1.89	1.05	1.31	2.44	1.20	0.74	0.73	0.84	0.84	1.15	1.01	0.95	0.47	0.67	1.02	1.02
	2016	0.52	6.09	0.80	0.78	2.00	1.25	2.98	3.73	1.10	0.71	0.77	0.76	1.19	1.12	1.32	0.89	0.39	0.87	1.99	0.83
Mexico	1990	0.46	1.99	0.81	0.33	0.97	0.94	1.78	0.83	0.96	1.03	1.01	1.07	0.44	0.75	0.68	0.71	1.30	0.79	1.13	1.76
	2016	0.48	2.70	0.66	0.34	0.80	1.13	2.84	0.83	0.89	1.13	0.98	0.97	0.62	0.79	0.79	0.70	0.76	1.20	3.92	1.11
Argentina	1990	0.88	0.77	1.26	0.88	0.58	0.77	0.65	0.75	1.06	1.15	0.65	1.08	0.54	0.95	1.42	1.47	0.77	1.53	1.09	1.12
	2016	0.55	1.26	1.11	0.56	0.75	0.85	0.97	1.60	1.07	0.97	0.65	1.00	0.77	0.96	1.05	1.39	0.60	1.94	1.39	0.93
Colombia	1990	0.73	6.37	0.88	0.51	0.86	1.04	0.65	0.52	1.04	0.77	0.99	1.10	0.50	0.68	0.84	0.71	1.03	0.88	0.93	0.58
	2016	0.45	4.90	0.75	0.31	0.65	1.13	0.80	0.56	0.98	0.85	0.93	1.02	0.64	0.64	0.75	0.61	0.66	1.15	1.10	0.73
Australia	1990	1.02	0.70	0.98	0.70	1.18	0.74	0.72	0.43	1.01	0.97	0.85	1.09	1.10	1.27	1.06	1.39	0.64	2.67	0.71	0.75
	2016	0.40	0.63	0.97	0.44	0.63	0.83	0.81	0.48	1.04	0.69	0.87	1.07	0.93	1.31	0.72	1.37	0.68	2.41	0.84	0.83
Canada	1990	1.00	0.86	0.95	0.63	1.21	0.72	1.02	0.79	1.19	1.11	0.77	1.07	0.87	1.02	1.13	1.27	0.75	1.99	0.81	0.97
	2016	0.50	0.90	0.89	0.50	0.71	0.86	1.14	0.72	1.23	0.88	0.82	0.99	0.93	1.00	1.26	1.15	0.77	2.28	1.00	1.05
England	1990	1.08	0.28	1.28	0.73	0.65	0.77	0.56	0.95	1.21	0.92	0.98	1.08	0.97	1.16	0.95	1.05	0.84	1.06	0.43	0.59
	2016	0.43	0.32	1.19	0.48	0.38	0.94	0.54	1.04	1.22	0.76	1.00	1.04	1.13	1.11	1.17	1.05	0.84	1.27	0.51	0.81

O:E < 0.5
O:E 0.5 - 0.99
O:E 1 - 1.99
O:E 2 - 4.99
O:E ≥ 5

Methods

The Global Burden of Disease (GBD) 2016 study organises causes of mortality and morbidity within a four-level classification hierarchy to produce estimates that are mutually exclusive and collectively exhaustive. The full GBD cause hierarchy, including corresponding International Classification of Diseases (ICD)-9 and ICD-10 codes, is detailed in the respective GBD 2016 publications. Risk factors are likewise organized in a four-tier hierarchy. The GBD 2016 included substantial methodological improvements from 2015, as described in detail in the GBD mortality, cause of death, years lived with disability (YLDs), disability-adjusted life years (DALYs) and healthy life expectancy (HALE), and risk factor publications (see "research in context" sections).[1]

1. Data

Data for all-cause mortality models were derived from vital registration systems, survey data, census data, birth histories, and sibling histories where complete vital registration information was not available. Cause-specific data included a comprehensive set of sources that met quality inclusion criteria, such as vital registration, disease registries, and verbal autopsy from 1980 to 2016. A complete list of data sources used in Brazil cause-specific mortality estimates is provided in Appendix Table 1, categorized by type of data source (vital registration, verbal autopsy, surveillance, sibling history, survey/census, cancer registry, or police records). Data on nonfatal outcomes come from published studies, survey data, ministry of health and central statistical office webpages, epidemiological surveillance data, and hospital inpatient data. A complete list of data sources used in Brazil morbidity estimates by cause is provided in Appendix Table 2, including the coverage level, year, and type of data source. Data on risks come primarily from published studies, surveys, censuses, and satellite data. A complete list of data sources used in Brazil risk factor estimates by risk is provided in Appendix Table 3, including the coverage level, year, and type of data source.

Inclusion and exclusion criteria for all-cause mortality and cause-specific mortality and morbidity data is available in the GBD mortality appendix, cause of death appendix, and YLDs appendix. [2]

Detailed information about deaths in Brazil was mainly obtained from the Mortality Information System (Sistema de Informações sobre Mortalidade, SIM) database.[3] Population censuses and intercensal estimates were provided by the Brazilian Institute of Geography and Statistics (Instituto Brasileiro de Geografia e Estatística, IBGE).[4]

---

[1] Wang H, Abajobir AA, Abate KH, et al. Global, regional, and national under-5 mortality, adult mortality, age-specific mortality, and life expectancy, 1970–2016: a systematic analysis for the Global Burden of Disease Study 2016. The Lancet 2017; 390: 1084–150.
Naghavi M, Abajobir AA, Abbafati C, et al. Global, regional, and national age-sex specific mortality for 264 causes of death, 1980–2016: a systematic analysis for the Global Burden of Disease Study 2016. The Lancet 2017; 390: 1151–210.
Vos T, Abajobir AA, Abate KH, et al. Global, regional, and national incidence, prevalence, and years lived with disability for 328 diseases and injuries for 195 countries, 1990–2016: a systematic analysis for the Global Burden of Disease Study 2016. The Lancet 2017; 390: 1211–59.
Gakidou E, Afshin A, Abajobir AA, et al. Global, regional, and national comparative risk assessment of 84 behavioural, environmental and occupational, and metabolic risks or clusters of risks, 1990–2016: a systematic analysis for the Global Burden of Disease Study 2016. The Lancet 2017; 390: 1345–422.
Hay SI, Abajobir AA, Abate KH, et al. Global, regional, and national disability-adjusted life-years (DALYs) for 333 diseases and injuries and healthy life expectancy (HALE) for 195 countries and territories, 1990–2016: a systematic analysis for the Global Burden of Disease Study 2016. The Lancet 2017; 390: 1260–344.

[2] Ibid

[3] Ministério da Saúde. Sistema de Informação sobre Mortalidade. 2014. http://svs.aids.gov.br/cgiae/sim/ (accessed June 20, 2017).

[4] Censo Demográfico 2010. Instituto Brasileiro de Geografia e Estatística. 2015. http://censo2010.ibge.gov.br/ (accessed June 20, 2017).

To evaluate the completeness of death registration, for every intercensal period we applied three death distribution methods: synthetic extinct generations; generalized growth balance; and a hybrid of the first two methods, as described in detail elsewhere.[5] We use the entire time series of these death distribution estimates of completeness and the completeness of child death registration to generate our time series of registration completeness.[5]

An assessment by Campos de Lima and colleagues estimated that the completeness of death counts increased from about 80% in 1980–1991 to over 95% in 2000–2010, and the percentage of ill-defined causes of death decreased about 53%.[6] Despite these improvements, the GBD found that in 2015, 18% of deaths in Brazil were coded to garbage codes (GC), meaning that the deaths were assigned to ill-defined diagnoses or to conditions that cannot be causes or underlying causes of death. The garbage codes were reassigned to specific ICD codes by age, sex, location, and year using proportional, negative correlation, multiple cause of death, and fixed proportion redistribution methods.

2. Mortality

We report data on cause-specific mortality and cause-specific years of life lost (YLLs) due to premature mortality for 264 causes of mortality. For GBD 2016, 68 causes were considered causes of disability but not death, while 5 were considered causes of death but not disability. Cause-specific mortality estimates for each age, sex, and location-year were most commonly generated using the GBD Cause of Death Ensemble model (CODEm) and the CodCorrect process. In CODEm, a variety of models were developed. For each individual model, out-of-sample predictive validity was assessed and models were ranked for use in ensemble modelling. The ensemble with the highest out-of-sample predictive validity was selected from differently weighted combinations of individual models. For causes where there was evidence that children and adults had different relationships between their covariates and death rates, separate models were run for different age ranges. Separate models were also developed for countries with complete, representative, and extensive VR by cause, including Brazil, to ensure that uncertainty reflects the more complete data in those locations. The CoDCorrect process ensures that there is internal consistency between cause-specific and all-cause mortality estimates by rescaling causes up the GBD hierarchy using a core algorithm described in more detail in the GBD cause-specific mortality paper appendix.[6]

3. Morbidity

Estimates of morbidity were produced for 328 causes and 2982 sequela for each age, sex, and year. For GBD 2016, 68 causes were considered causes of disability but not death, while 5 were considered causes of death but not disability. To estimate YLDs, the Bayesian meta-regression tool DisMod-MR 2.1 estimated prevalence and incidence for most causes of disease and injury and their non-fatal outcomes, calculated the product of incidence and a specific disability weight for each sequelae, adjusted for comorbidity, and aggregated to cause-level, ensuring consistency for each condition. Several causes were estimated using custom models; details of these causes and their

---

[5] Naghavi M, Abajobir AA, Abbafati C, et al. Global, regional, and national age-sex specific mortality for 264 causes of death, 1980–2016: a systematic analysis for the Global Burden of Disease Study 2016. The Lancet 2017; 390: 1151–210.

[6] Lima, E. E. C. D., & Queiroz, B. L. (2014). Evolution of the deaths registry system in Brazil: associations with changes in the mortality profile, under-registration of death counts, and ill-defined causes of death. Cadernos de Saúde Pública 2014; 30(8), 1721-1730. http://dx.doi.org/10.1590/0102-311X00131113

modelling strategy can be found in the GBD YLDs publication. YLDs were estimated for all mutually exclusive sequelae by multiplying prevalence by a disability weight, then correcting for comorbidity and aggregating to cause level.

4. Disability-adjusted life-years

DALYs are a combined measure of mortality and morbidity. They are calculated by summing YLLs and YLDs for each age, sex, location, and year.

5. Risk factors

Deaths, YLLs, YLDs, and DALYs attributable to 84 risk factors or clusters of risk factors were assessed in GBD 2016. For each risk-outcome pair, relative risks of mortality and morbidity were estimated on the basis of meta-analyses of the literature. Second, exposure to each risk factor in each country and Brazilian state by age, sex, and year was estimated on the basis of published and unpublished data with primarily Bayesian estimation methods. Finally, attributable deaths or DALYs were estimated by comparing the present distribution of exposure to a theoretical minimum risk distribution of exposure selected for each risk factor. Each risk, exposure estimate, and theoretical minimum risk distribution and uncertainty in the background outcome rates have been propagated into the final estimates.

6. Socio-demographic Index

The Socio-demographic-Index (SDI) is a summary indicator based on average lag-dependent income per capita, total fertility rate in the population, and years of education attained in the population over 15 years of age, calculated as the geometric mean of the rescaled (0-1) values of the three components for each location-year. The 2016 SDI score for Brazil was 0.71. GBD 2016 grouped locations into low, low-middle, middle, high-middle, and high SDI for analysis.

Expected values calculation

Gaussian process regression was used to estimate the relationship between SDI and each age-sex-cause death rate. These relationships were used to estimate expected YLLs based on SDI alone for each age, sex, location, and year.

Scale of SDI

For each component of the SDI, 0 represents a theoretical minimum level of development for the selected health outcomes and 1 represents a theoretical maximum level of development for the selected health outcomes. Thresholds were set based on the relationship between each component with under 5 mortality rates and life expectancy at birth and identified points of limiting returns if they occurred prior to theoretical limits.

7. Uncertainty levels

Uncertainty levels were propagated at multiple stages throughout the GBD modelling process. Uncertainty for mortality and YLLs reflected uncertainty in the levels of all-cause mortality and uncertainty in the estimation of each mortality cause, in each age group, sex, and year. Uncertainty in the disability weight for each sequela was propagated into the estimates of YLDs for each disease and injury. A sample of 1,000 draws was taken from the posterior distribution of each estimation step; aggregation of uncertainty across age, sex, and location was performed on each draw,

assuming independence of uncertainty. The lower and upper uncertainty intervals (UI) represent the ordinal 25th and 975th draws of each quantity and attempt to describe modelling as well as sampling error.
